# Developing Machine-Learning Models to Predict Bacteremia in Febrile Adults Presenting to the Emergency Department: A Retrospective Cohort Study from a Large Center

**DOI:** 10.5811/westjem.35866

**Published:** 2025-05-30

**Authors:** Chia-Ming Fu, Ike Ngo, Pak Sheung Lau, Yaroslav Ivanchuk, Fan-Ya Chou, Chih-Hung Wang, Chien-Yu Lin, Chu-Lin Tsai, Shey-Ying Chen, Tsung-Chien Lu, Hung-Yu Wei

**Affiliations:** * National Taiwan University Hospital, Department of Emergency Medicine, Taipei City, Taiwan; † Min-Sheng General Hospital, Department of Emergency Medicine, Taoyuan City, Taiwan; ‡ National Taiwan University, Department of Electrical Engineering, Taipei City, Taiwan; § National Taiwan University, College of Medicine, Department of Emergency Medicine, Taipei City, Taiwan; || En Chu Kong Hospital, Department of Nephrology, New Taipei City, Taiwan; # Fu Jen Catholic University, School of Medicine, New Taipei City, Taiwan

## Abstract

**Introduction:**

Bacteremia, a common disease but difficult to diagnose early, may result in significant morbidity and mortality without prompt treatment. We aimed to develop machine-learning (ML) algorithms to predict patients with bacteremia from febrile patients presenting to the emergency department (ED) using data that is readily available at the triage.

**Methods:**

We included all adult patients (≥18 years of age) who presented to the emergency department (ED) of National Taiwan University Hospital (NTUH), a tertiary teaching hospital in Taiwan, with the chief complaint of fever or measured body temperature more than 38°C, and who received at least one blood culture during the ED encounter. We extracted data from the Integrated Medical Database of NTUH from 2009–2018.The dataset included patient demographics, triage details, symptoms, and medical history. The positive blood culture result of at least one potential pathogen was defined as bacteremia and used as the binary classification label. We split the dataset into training/validation and testing sets (60-to-40 ratio) and trained five supervised ML models using K-fold cross-validation. The model performance was evaluated using the area under the receiver operating characteristic curve (AUC) in the testing set.

**Results:**

We included 80,201 cases in this study. Of them, 48120 cases were assigned to the training/validation set and 32,081 to the testing set. Bacteremia was identified in 5,831 (12.1%) and 3,824 (11.9%) cases of the training/validation set and test set, respectively. All ML models performed well, with CatBoost achieving the highest AUC (.844, 95% confidence interval [CI] .837–.850), followed by extreme gradient boosting (.843, 95% CI .836–.849), gradient boosting (.842, 95% CI .836–.849), light gradient boosting machine (.841, 95% CI .834–.847), and random forest (.828, 95% CI .821–.834).

**Conclusion:**

Our machine-learning model has shown excellent discriminatory performance to predict bacteremia based only on clinical features at ED triage. It has the potential to improve care quality and save more lives if successfully implemented in the ED.

## INTRODUCTION

Bacteremia, defined as the existence of bacteria in the bloodstream, is a serious and potentially life-threatening condition associated with mortality rates ranging from 13–35%.^1^–^3^ The timely and accurate diagnosis of bacteremia is crucial for appropriate treatment and management of patients who present with fever to the emergency department (ED). Emergency physicians often order blood culture tests for patients suspected of bacterial infections based on the presence of fever. However, excessive and unnecessary blood culture tests can lead to extended hospital length of stay, increased antibiotic use, additional laboratory testing, and even higher rates of side effects related to blood collection and antibiotic administration.^4^

Despite the advancements in modern medical technology, blood culture remains the gold standard for diagnosing bacteremia and determining antimicrobial susceptibility. However, it often takes more than 24 hours to achieve a detectable colony size, preventing emergency physicians from obtaining real-time blood culture results. This delay can lead to the administration of ineffective antibiotics or the premature discharge of patients with bacteremia, which may become evident later on. While detailed evaluations by emergency physicians may not always lead to irreparable consequences, bacteremia caused by specific bacteria can indeed result in unfavorable outcomes.^5^–^7^

Previous studies have identified various variables, including vital signs, clinical symptoms, comorbid conditions, and laboratory values, which are independently associated with bacteremia.^8^–^10^ Several prediction models have also been developed, with area under the receiver operating characteristic curve (AUC) values ranging from .71–.854.^11^–^15^ In recent years, machine-learning (ML) techniques have been applied to predict bacteremia, but most studies have primarily focused on hospitalized patients,^16^–^19^ with limited research involving ED patients.^20^,^21^ Furthermore, many of these studies rely on laboratory results that are not immediately available in the ED. In the ED setting, physicians typically rely solely on demographics, ED triage data, symptoms, and past medical history to make early predictions of bacteremia, emphasizing the critical role of ordering necessary blood cultures.^21^,^22^

In light of these challenges, we sought to create a predictive model that can assist emergency physicians in making timely and informed decisions regarding the need for blood culture tests and appropriate treatment strategies. By harnessing the power of ML and incorporating a wide range of clinical, demographic, and other accessible variables during the ED encounter collected from electronic medical records (EMR), our study aimed to develop the ML algorithms that can predict bacteremia in febrile patients presenting to the ED using data readily available during the triage and history-taking stages. Additionally, we discuss the potential implications of our findings, acknowledge the study’s limitations, and outline future directions for research in this critical field of emergency medicine.

**Population Health Research Capsule**

What do we already know about this issue?

*Early diagnosis of bacteremia is challenging, yet delayed treatment increases morbidity and mortality in febrile emergency department (ED) patients.*

What was the research question?

*Can machine-learning methods predict bacteremia in febrile ED patients using triage data?*

What was the major finding of the study?

*CatBoost achieved the highest AUC (.844, 95% CI .837–.850) for bacteremia prediction at ED triage.*

How does this improve population health?

*Early bacteremia prediction by machine-learning at triage has the potential to enable timely treatment, improving outcomes and reducing mortality in ED patients.*

## METHODS

### Study Design and Setting

We conducted this retrospective cohort study using EMR data extracted from the integrated Medical Database (iMD) of National Taiwan University Hospital (NTUH), a 2,500-bed, university-affiliated teaching hospital providing primary, secondary, and tertiary care in northern Taiwan. The hospital has various departments covering all major specialties, including transplantation and oncology. The ED of this hospital sees an annual average of 110,000 patient visits, with approximately 91,600 visits when excluding pediatric (<18 years of age) cases. This study was approved by the Institutional Review Board of NTUH (202104109RINC) with a waiver of informed consent. This study protocol followed the guidelines of “Minimum information about clinical artificial intelligence modeling: the MI-CLAIM checklist”.^23^

### Study Population

We included all adult patients (≥18 years of age) who presented to the ED of NTUH over a 10-year period (January 2009–December 2018) with the chief complaint of fever or measured body temperature of more than 38° Celsius at triage, and received at least one blood culture during the ED encounter. Repeat ED visits by the same patient within a 30-day period were considered as the same index visit, and duplicate visits were eliminated from the analysis.

### Collection of Variables (Features)

From the iMD, we obtained patient demographics, including age, sex, height, and body mass index (BMI), that are readily available during triage. We also collected ED triage data, which encompassed the five-level Taiwan Triage and Acuity Scale (TTAS), details of emergency medical services (EMS) transport, and transfer status.^24^ The initial ED presenting vital signs, including the Glasgow Coma Scale (GCS) score, body temperature, pulse rate, respiratory rate, oxygen saturation (SpO2), pain index, and acute changes in consciousness, were also retrieved. The GCS scores were further categorized into clear consciousness (GCS 15), minor coma (GCS 13–14), moderate coma (GCS 9–12), severe coma (GCS 3–8), and other (intubated, tracheostomy, or aphasia). Furthermore, we extracted clinical symptoms from the TTAS documentation, which encompassed a total of 179 structured chief complaints (CC) recorded during patient encounters by the triage nurse. Incorporating past medical histories (PMH) as input features for this study involved two different sources of information. First, PMHs were obtained through direct patient reporting during their current ED encounters as part of the interview conducted by the triage nurse while enquiring their CCs. The second source of PMH data was derived from patients’ prior chart records, spanning outpatient, inpatient, or previous ED admissions, and subsequently coded using the *International Classification of Diseases*, *10th Rev* (ICD-10) codes.^25^

### Outcome Measures

The primary outcome of this study —the presence of true bacteremia—was also employed as the classification label for our binary classification task. Patients were labeled as having true bacteremia when either a single blood culture yielded pathogenic bacteria or when two or more sets of blood cultures collected from distinct sites revealed the same bacterial species. Contaminants, including coagulase-negative staphylococci, *Corynebacterium* species, *Bacillus* species (except for *B anthracis*), *Propionibacterium* species, *Micrococcus* species, and *Clostridium perfringens*, were identified and excluded based on prior studies, except in cases where a significant indwelling catheter was present.^26^

### Data Analysis and Feature Selection

We used Microsoft Excel 2016 (Microsoft Corporation, Redmond, WA) for data entry and processing. The data was further analyzed with SPSS Statistics for Windows v24.0 (IBM Corp, Armonk, NY). The variables with missing values were imputed by replacing them with the mean, median, or mode of their respective class if the missing rate was less than 20% for that variable. We presented the results as percentages for categorical variables, standard deviations for continuous variables, and medians with interquartile ranges (IQR) for time variables. Our feature selection approach involved univariate analyses, where we assessed outcome differences between groups using statistical tests such as Student *t*-test, chi-squared test, Fisher exact test, or Mann-Whitney U test, depending on the distribution of the data. We selected variables with a significance level of *P*<.05 in the training cohort as the input features for constructing the ML models.

### Machine-Learning Model Construction

We employed supervised ML algorithms using random forest (RF), gradient boosting (GB), CatBoost (CB), light gradient boosting machine (LGBM) and extreme gradient boosting (XGB) to construct the prediction models. The dataset was randomly split into the training/validation and testing cohorts at a ratio of 60:40. To train our model, we employed K-fold cross-validation, with the value of K ranging from 7–10. The model’s performances were evaluated through the area under the receiver operating characteristic curve (AUC) on the test set. We selected the K value that resulted in the highest AUC performance as our final choice. In addition to AUC, we also reported the classification performances on the testing cohort using F1-score, precision (or positive predictive value [PPV]), recall (sensitivity), specificity, negative predictive value (NPV), and area under the precision-recall curve (AUPRC) for each model. Furthermore, we incorporated SHAP (Shapley additive explanations) value analysis alongside feature importance to enhance the interpretability and transparency of the ML models we developed.^27^ All ML analyses were performed using Python 3.8 programming language (Python Software Foundation, Wilmington, DE) with package scikit-learn 0.23.1 installed.

## RESULTS

**Figure 1:**
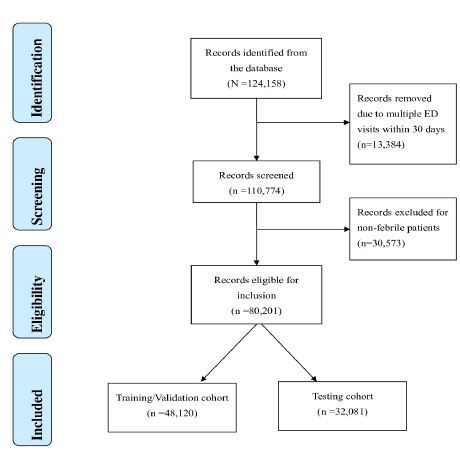
The case inclusion and exclusion flow chart. *ED*, emergency department.

During the study period, a total of 124,158 adult ED visits with at least one blood culture performed were retrieved from the iMD. After excluding records of repeat ED visits within 30 days and patients without fever, we included 80,201 records for analysis. The flow chart of case inclusion and exclusion process is shown in Figure 1. We assigned 48,120 cases to the training/validation cohort and 32,081 to the testing cohort. True bacteremia was identified in 5,831 (12.1%) and 3,824 (11.9%) cases of the training/validation cohort and testing cohort, respectively.

The characteristics of the study population, including patient demographics and ED triage data for both the training/validation and testing cohorts, are presented in Table 1. A comprehensive breakdown of population characteristics, including past medical histories (PMH) and ED presenting symptoms across groups, can be found in Supplementary Table S1. Univariate analyses of features distinguishing patients with and without bacteremia are partially summarized in Table 2 for the training/validation and testing sets. Full details are provided in Supplementary Table S2.

**Figure 2:**
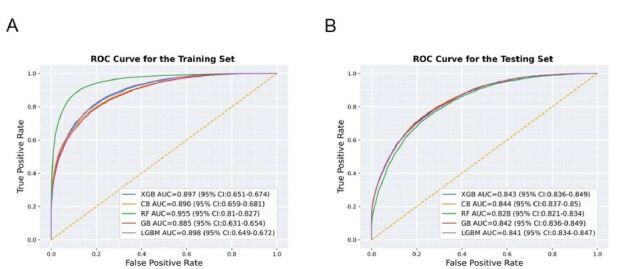
Results and the comparisons of the five machjine-learning models on the training/validation (A), and Testing (B) cohorts, based on the performances of area under the receiver operating characteristic (ROC) curves (AUC). *RF*, random forest; *GB*, gradient boosting; *CB*, CatBoost; *LGBM*, light gradient boosting machine; *XGB*, extreme gradient boosting; *ROC*, receiver operating characteristic; *AUC*, area under the curve.

A total of 395 clinical features were selected by setting the *P*-value <0.05 from the training/validation cohort. These features comprised six demographic factors, 10 triage-related variables, and 25 symptoms. Additionally, there were 354 PMHs sourced from two distinct origins: 98 features were gathered through direct patient interviews conducted by triage nurses, while 256 features were extracted from EMR-based ICD-10 codes. Five ML models, including RF, LGBM, XGB, CB, and GB, were constructed for predicting bacteremia. By selecting 9-fold cross validation, the constructed models showed excellent discrimination ability on the training/validation cohort and maintained their discriminatory performances on the testing cohort in terms of AUC (Figure 2).

As illustrated in Table 3, the ML model constructed using 9-fold cross-validation in the testing cohort achieved the better area under the curve (AUC) with CB at 0.844 (95% CI 0.837–0.850), closely followed by XGB at 0.843 (95% CI 0.836–0.839), GB at 0.842 (95% CI 0.836–0.849), LGBM at 0.841 (95% CI 0.834–0.847), and RF at 0.828 (95% CI 0.821–0.834). When considering the balance between precision and recall by calculating the AUPRC, CB exhibited performance at 0.540 (95% CI 0.525–0.555), followed by XGB at 0.537 (95% CI 0.522–0.552), GB at 0.536 (95% CI 0.521–0.550), LGBM at 0.533 (95% CI 0.518–0.548), and RF at 0.473 (95% CI 0.458–0.489). Except for RF, the differences in AUC and AUPRC among the ML models were not statistically significant. Additional performance metrics, including sensitivity (or recall), specificity, negative predictive value (NPV), and positive predictive value (PPV, or precision), are shown in Table 3.

**Figure 3:**
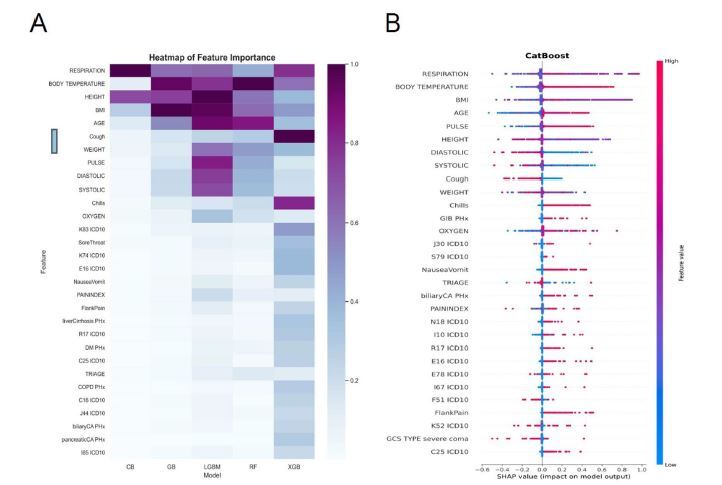
A heat map of the computed top 30 features ordered by median normalized importance across all models of the constructed five different machine-learning (ML) models (A). The Shapley additive explanations of the top 30 important features as a way to explain the output of the constructed ML models by selecting 9-fold cross validation using CB classifiers (B). *PHx*, past history; *ICD-10, International Classification of Diseases* 10th Rev; *DM*, diabetes mellitus; *COPD*, chronic obstructive pulmonary disease; *BMI*, body mass index; CB, CatBoost; *GIB*, gastrointestinal bleeding; *GCS*, Glasgow Coma Scale.

A heat map of the computed top 30 features ordered by median normalized importance across all models of the constructed five different ML models are shown in Figure 3A. The top 100 important features visualized as a heat map are available in Supplementary Figure S1. Of them, the eight most important features selected for constructing the ML models are respiratory rate, body temperature, height, BMI, age, cough, weight, and pulse rate. Using the SHAP values approach, we present the outcomes of our analysis conducted with a 9-fold cross-validation in Figure 3B. This figure visually represents the top 30 importance scores assigned to each feature in predicting outcomes within the CB model we constructed. For the results pertaining to the other four models, please refer to Supplementary Figure S2.

## DISCUSSION

In this study, we used ML methods to predict the presence of bacteremia in febrile patients presenting to the ED. Using 395 clinical features that are readily available at the ED triage, all five ML models we constructed showed excellent discriminatory performance in predicting bacteremia, with AUC ranging from 0.828 to 0.844.

Bacteremia is a serious and potentially life-threatening condition that can lead to severe complications and increased mortality. The diagnosis of bacteremia relies on a high index of suspicion by the emergency physician to order for blood culture tests based on the patient’s demographics, triage data, CCs, PMH, and physical examination. Predicting bacteremia has been studied for decades. However, previous studies have found that the accuracy of clinician impressions was poor,^28^ leading to subsequent studies to include laboratory testing results as predicting variables. However, laboratory results are not readily available at the initial triage and history-taking stage. Even in studies that adopted laboratory values as predicting features, these prediction models still exhibit suboptimal performance in predicting bacteremia with small sample sizes and often lack a validation group for confirmation.^11^–^14^

In the era of big data and ML, Choi et al developed and validated ML models to predict bacteremia in the ED during triage stages.^20^ The best performing model, the “Triage XGB model,” demonstrated only acceptable discrimination performance with an AUC of 0.718 by using demographics, triage data, and CCs as predicting variables. However, comorbidity is also an important reference for emergency physicians to assess patients’ prognosis and whether they have bacteremia.^29^ Our study included 80,201 ED visits during a 10-year period, including demographics, triage data, CCs, and PMH obtained through patient interviews by the triage nurse and retrieved from the patients’ EMRs. The AUC of our five ML models all demonstrated excellent results, with the CB model achieving the best performance (0.844).

Furthermore, by using SHAP analysis, our study offers additional evidence highlighting the importance of triage vital signs such as respiratory rate, body temperature, and pulse rate in predicting bacteremia, as depicted in both Figure 3B and Supplementary Figure S2. Notably, our findings reveal that advanced patient age and the presence of chills are also associated with positive predictors of bacteremia. These observations emphasize the importance of vigilance among emergency physicians when encountering such patients, warranting prompt action, including the ordering of blood culture tests.^13^ In contrast, the symptom of cough is a negative predictor of bacteremia possibly from patients experiencing upper respiratory infection due to viral infection.^30^

In addition to the AUC, we also evaluated the performance of our models using a range of available performance metrics, including sensitivity, specificity, PPV, and NPV. Prediction models from previous studies always showed high sensitivity and NPV but low specificity and PPV; thus, they can only be used clinically to rule out the possibility of bacteremia. The low specificity and PPV may lead to many false positive results, resulting in antibiotic overuse, prolonged length of stay in the ED, and even higher hospitalization rates. While the models we developed exhibit low F1 and recall scores, indicating challenges in identifying true positive cases, they can still serve valuable roles when carefully contextualized. For instance, the models can act as screening tools for bacteremia or provide supporting diagnostic insights. Additionally, our models demonstrate excellent specificity and NPV while also achieving good PPV and AUPRC, which suggests robust overall diagnostic performance, particularly in their ability to balance ruling out negative cases and identifying positive cases effectively.

## LIMITATIONS

There are several limitations in this study. Firstly, this was a retrospective database analysis relying on data collected exclusively from patients who underwent blood culture examinations. We could have missed those patients who might have had bacteremia but did not receive blood culture examination, potentially introducing selection bias. Secondly, the acquisition of patients’ PMHs was reliant on interviews conducted by triage nurses and the EMR. In cases where patients had no prior hospital visits, the PMH was solely obtained through interviews with triage nurses, which might have introduced recall bias.

Thirdly, our models achieved low F1 score and recall, indicating the potential of missed diagnoses. Further strategies—such as addressing class imbalance with techniques like oversampling or undersampling, or optimizing decision thresholds—could be implemented to enhance these metrics and make the models even more reliable for diagnostic tasks. Fourthly, it’s important to note that the dataset used in this study was derived from a single, tertiary teaching hospital’s ED, and external validation from other healthcare settings is lacking. Our future works will expand our study to a regional, multicenter cohort to validate our model’s performance and improve its generalizability.

## CONCLUSION

We have developed ML models to predict bacteremia, achieving excellent discrimination performance by using clinical features readily accessible during ED triage. These models demonstrate potential for identifying low-risk patients, which could help reduce unnecessary healthcare costs. Furthermore, they may assist emergency physicians in making more informed decisions regarding blood cultures orders and antibiotics administration for high-risk patients, potentially improving patient safety. However, as this study lacks external validation, the generalizability of our findings to other settings remains uncertain. Future work should focus on validating these models with external datasets to confirm their robustness and applicability across diverse clinical environments.

## Supplementary Information









## Figures and Tables

**Table 1:** Patient demographics, ED triage data of the training/validation cohort and testing cohort.

Variables (features)	Total (N=80,201)		Training cohort (n=48,120)		Testing cohort (n=32,081)		*P-value*
Sex							0.87
Female	40,220	(50.1)	24,143	(50.2)	16,077	(50.1)	
Male	39,981	(49.9)	23,977	(49.8)	16,004	(49.9)	
Age, Mean (SD)	59.3	(20.0)	59.3	(20)	59.4	(20.1)	0.36
Temperature, Mean (SD)	38.4	(1.4)	38.4	(1.3)	38.4	(1.5)	0.25
Acute change							0.47
No	71,876	(89.6)	43,142	(89.7)	28,734	(89.6)	
Yes	2,570	(3.2)	1,561	(3.2)	1,009	(3.1)	
NA	5,755	(7.2)	3,417	(7.1)	2,338	(7.3)	
EMS transfer							0.12
No	64,486	(80.4)	38,686	(80.4)	25,800	(80.4)	
Yes	68	(0.1)	47	(0.1)	21	(0.1)	
NA	15,647	(19.5)	9,387	(19.5)	6,260	(19.5)	
Triage							0.83
1	3,477	(4.3)	2,118	(4.4)	1,359	(4.2)	
2	20,770	(25.9)	12,448	(25.9)	8,322	(25.9)	
3	54,769	(68.3)	32,851	(68.3)	21,918	(68.3)	
4	1,127	(1.4)	669	(1.4)	458	(1.4)	
5	58	(0.1)	34	(0.1)	24	(0.1)	
Systolic BP, Mean (SD)	132.5	(27.1)	132.5	(27)	132.6	(27.2)	0.88
DiastolicC BP, Mean (SD)	75.7	(15.1)	75.7	(15.1)	75.7	(15.2)	0.97
Pulse, Mean (SD)	106.2	(19.7)	106.4	(19.7)	105.9	(19.7)	0.003
Oxygen, Miean (SD)	95.9	(3.6)	95.9	(3.5)	95.9	(3.7)	0.97
Respiration rate, Mean (SD)	19.6	(5.5)	19.6	(4.8)	19.6	(6.4)	0.77
Pain Index							0.19
0	58,424	(72.8)	35,085	(72.9)	23,339	(72.8)	
1	49	(0.1)	22	(0.0)	27	(0.1)	
2	314	(0.4)	187	(0.4)	127	(0.4)	
3	847	(1.1)	510	(1.1)	337	(1.1)	
4	2,430	(3.0)	1,444	(3)	986	(3.1)	
5	5,315	(6.6)	3,182	(6.6)	2,133	(6.6)	
6	2,749	(3.4)	1,659	(3.4)	1,090	(3.4)	
7	3,867	(4.8)	2,387	(5)	1,480	(4.6)	
8	2,549	(3.2)	1,488	(3.1)	1,061	(3.3)	
9	494	(0.6)	289	(0.6)	205	(0.6)	
10	596	(0.7)	351	(0.7)	245	(0.8)	
NA	2,567	(3.2)	1516	(3.2)	1051	(3.3)	
Height, Mean (SD)	161.8	(10.4)	161.8	(10.5)	161.8	(10.2)	0.88
Weight, Mean (SD)	61.1	(19.4)	61.1	(22.5)	61.1	(13.5)	0.73
BMI, Mean (SD)	23.2	(4.4)	23.2	(4.4)	23.2	(4.4)	0.79
GCS TYPE							0.32
Clear consciousness	65,917	(82.2)	39,514	(82.1)	26,403	(82.3)	
Minor coma	1,240	(1.5)	766	(1.6)	474	(1.5)	
Moderate coma	3,553	(4.4)	2,155	(4.5)	1,398	(4.4)	
Severe coma	1,747	(2.2)	1,047	(2.2)	700	(2.2)	
others	1,686	(2.1)	1,043	(2.2)	643	(2.0)	
NA	6,058	(7.6)	3,595	(7.5)	2,463	(7.7)	

*ED*, emergency department; *EMS*, emergency medical service; *BP*, blood pressure; *BMI*, body mass index; *GCS*, Glasgow Coma Scale; *NA*, not available

**Table 2:** Univariate analyses of features between patients with or without bacteremia on the on the training/validation and testing cohorts.

Cohort	Training/validation					Testing cohort				
		
Variables (features)	Bacteremia (−) (n=42,298)		Bacteremia (+) (n=5,822)		*P-value*	Bacteremia (−) (n=28,248)		Bacteremia (+) (n=3,833)		*P-value*
Sex					0.04					0.04
Female	21,147	(50.0)	2,996	(51.5)		14,097	(49.9)	1,980	(51.7)	
Male	21,151	(50.0)	2,826	(48.5)		14,151	(50.1)	1,853	(48.3)	
Age, Mean (SD)	58.3	(20.3)	66.4	(16.3)	<.001	58.4	(20.3)	66.6	(16.4)	<0.001
Body temperature, Mean (SD)	38.4	(1.3)	38.8	(1.4)	<.001	38.4	(1.5)	38.8	(1.3)	<0.001
Acute change					<.001					<0.001
No	38,090	(90.1)	5,052	(86.8)		25,425	(90.0)	3,309	(86.3)	
Yes	1,229	(2.9)	332	(5.7)		780	(2.8)	229	(6.0)	
NA	2,979	(7.0)	438	(7.5)		2,043	(7.2)	295	(7.7)	
EMS					0.005					0.72
No	33,974	(80.3)	4,712	(80.9)		22,687	(80.3)	3,113	(81.2)	
Yes	35	(0.1)	12	(0.2)		19	(0.1)	2	(0.1)	
NA	8289	(19.6)	1098	(18.9)		5542	(19.6)	718	(18.7)	
Triage					<0.001					<0.001
1	1,616	(3.8)	502	(8.6)		1,038	(3.7)	321	(8.4)	
2	10,618	(25.1)	1,830	(31.4)		7,133	(25.3)	1,189	(31.0)	
3	29,392	(69.5)	3,459	(59.4)		19,619	(69.5)	2,299	(60.0)	
4	639	(1.5)	30	(0.5)		434	(1.5)	24	(0.6)	
5	33	(0.1)	1	(0.0)		24	(0.1)	0	(0.0)	
Systolic BP, Mean (SD)	132.7	(26.4)	131.2	(30.8)	<0.001	132.8	(26.7)	130.7	(30.6)	<0.001
Diastolic BP, Mean (SD)	76.1	(14.9)	72.8	(16.2)	<0.001	76.1	(14.9)	72.4	(16.4)	<0.001
Pulse, Mean (SD)	105.7	(19.3)	111.1	(21.5)	<0.001	105.3	(19.4)	110.5	(21.6)	<0.001
Oxygen, Mean (SD)	96	(3.5)	95.6	(4.1)	<.001	96	(3.6)	95.7	(4.2)	<0.001
Respiration, Mean (SD)	19.5	(4.7)	20.1	(5.2)	<.001	19.5	(6.7)	20	(3.8)	<0.001
Pain index					<.001					<0.001
0	30,657	(72.5)	4,428	(76.1)		20,397	(72.2)	2942	(76.8)	
1	21	(0.0)	1	(0.0)		25	(0.1)	2	(0.1)	
2	179	(0.4)	8	(0.1)		114	(0.4)	13	(0.3)	
3	460	(1.1)	50	(0.9)		315	(1.1)	22	(0.6)	
4	1,303	(3.1)	141	(2.4)		897	(3.2)	89	(2.3)	
5	2,879	(6.8)	303	(5.2)		1,939	(6.9)	194	(5.1)	
6	1,494	(3.5)	165	(2.8)		974	(3.4)	116	(3.0)	
7	2,112	(5.0)	275	(4.7)		1,333	(4.7)	147	(3.8)	
8	1,323	(3.1)	165	(2.8)		940	(3.3)	121	(3.2)	
9	246	(0.6)	43	(0.7)		184	(0.7)	21	(0.5)	
10	311	(0.7)	40	(0.7)		203	(0.7)	42	(1.1)	
NA	1,313	(3.1)	203	(3.5)		927	(3.3)	124	(3.2)	
Height, Mean (SD)	162	(10.4)	160.4	(11.5)	<.001	162.1	(10.2)	160.3	(10.6)	<0.001
Weight, Mean (SD)	61.2	(23.5)	60.3	(13.3)	0.01	61.1	(13.6)	60.4	(12.9)	0.001
BMI, Mean (SD)	23.2	(4.4)	23.4	(4.6)	0.003	23.2	(4.4)	23.3	(4.4)	0.04
GCS type					<.001					<0.001
NA	3,134	(7.4)	461	(7.9)		2,157	(7.6)	306	(8.0)	
Clear consciousness	34,958	(82.6)	4,556	(78.3)		23,390	(82.8)	3013	(78.6)	
Minor coma	634	(1.5)	132	(2.3)		384	(1.4)	90	(2.3)	
Moderate coma	1,768	(4.2)	387	(6.6)		1,147	(4.1)	251	(6.5)	
Other	929	(2.2)	114	(2.0)		575	(2.0)	68	(1.8)	
Severe coma	875	(2.1)	172	(3.0)		595	(2.1)	105	(2.7)	

*EMS*, emergency medical service; *BP*, blood pressure; *BMI*, body mass index; *GCS*, Glasgow Coma Scale; *NA*, not available

**Table 3:** Comparison between model performances on the training/validation and testing cohorts.

Model	AUC (95% CI)	AUPRC (95% CI)	F1	Kappa	Sensitivity (Recall)	Specificity	PPV (Precision)	NPV
**Training/validation cohort**								
CatBoost	0.890 (0.886–0.895)	0.670 (0.659–0.681)	0.462	0.428	0.307	0.997	0.932	0.913
XGBoost	0.897 (0.893–0.901)	0.663 (0.651–0.674)	0.439	0.404	0.289	0.996	0.909	0.911
Gradient boosting	0.885 (0.880–0.889)	0.643 (0.631–0.654)	0.440	0.404	0.292	0.995	0.891	0.911
Light GBM	0.898 (0.894–0.903)	0.661 (0.649–0.672)	0.412	0.379	0.266	0.997	0.914	0.908
Random forest	0.955 (0.952–0.957)	0.818 (0.810–0.827)	0.699	0.649	0.839	0.923	0.599	0.977
**Testing cohort**								
CatBoost	0.844 (0.837–0.850)	0.540 (0.525–0.555)	0.378	0.339	0.249	0.990	0.779	0.906
XGBoost	0.843 (0.836–0.849)	0.537 (0.522–0.552)	0.372	0.334	0.244	0.991	0.785	0.906
Gradient boosting	0.842 (0.836–0.849)	0.536 (0.521–0.550)	0.380	0.341	0.252	0.990	0.773	0.907
LightGBM	0.841 (0.834–0.847)	0.533 (0.518–0.548)	0.361	0.325	0.233	0.992	0.803	0.905
Random forest	0.828 (0.821–0.834)	0.473 (0.458–0.489)	0.455	0.370	0.518	0.897	0.406	0.932

Note: The best parameters for CatBoost are {‘depth’: 4, ‘learning_rate’: 0.12, ‘random_seed’: 0, ‘loss_function’: ‘Logloss’, ‘iterations’: 700}; for XGBoost are {‘learning_rate’: 0.14, ‘max_depth’: 4, ‘n_estimators’: 300}; for Gradient boosting are {‘max_depth’: 3, ‘learning_rate’: 0.1, ‘n_estimators’: 600, ‘random_state’: 0}: for Light GBM are {‘learning_rate’: 0.1, ‘n_estimators’: 100, ‘max_depth’: 30, ‘random_state’: 0}: for Random forest are {‘max_depth’: 35, ‘n_estimators’: 1000, ‘random_state’: 0, ‘max_leaf_nodes’: 1000, ‘class_weight’: ‘balanced’}.

*AUC*, area under the receiver operating characteristic curve; *AUPRC*, area under the precision recall curve; *CI*, confidence interval; *PPV*, positive predictive value; *NPV*, negative predictive value; *GBM*, gradient boosting machine; *XGBoost*, eXtreme Gradient Booting.
