# Supplementary material for: Developing Machine-Learning Models to Predict Bacteremia in Febrile Adults Presenting to the Emergency Department: A Retrospective Cohort Study from a Large Center
Source: West J Emerg Med. 2025 May 30;26(3):617–26. doi: 10.5811/westjem.35866 (PMC12208070; doi:10.5811/westjem.35866)
Supplement: Supplementary file 1 [file wjem-26-617-s001.pdf]

Supplementary Table 1. Characteristics of the study population (complete list).

Variables (Features)	Total (n=80201)		Training/validation cohort (n=48120)		Testing cohort (n=32081)		P value
GENDER							0.87
Female	40220	(50.1)	24143	(50.2)	16077	(50.1)	
Male	39981	(49.9)	23977	(49.8)	16004	(49.9)	
AGE, Mean(SD)	59.3	(20.0)	59.3	(20.0)	59.4	(20.1)	0.36
BODY TEMPERATURE, Mean(SD)	38.4	(1.4)	38.4	(1.3)	38.4	(1.5)	0.25
ACUTE CHANGE							0.47
No	71876	(89.6)	43142	(89.7)	28734	(89.6)	
Yes	2570	(3.2)	1561	(3.2)	1009	(3.1)	
NA	5755	(7.2)	3417	(7.1)	2338	(7.3)	
EMS Transfer							0.12
No	64486	(80.4)	38686	(80.4)	25800	(80.4)	
Yes	68	(0.1)	47	(0.1)	21	(0.1)	
NA	15647	(19.5)	9387	(19.5)	6260	(19.5)	
TRIAGE							0.83
1	3477	(4.3)	2118	(4.4)	1359	(4.2)	
2	20770	(25.9)	12448	(25.9)	8322	(25.9)	
3	54769	(68.3)	32851	(68.3)	21918	(68.3)	
4	1127	(1.4)	669	(1.4)	458	(1.4)	
5	58	(0.1)	34	(0.1)	24	(0.1)	
SYSTOLIC BP, Mean(SD)	132.5	(27.1)	132.5	(27.0)	132.6	(27.2)	0.88
DIASTOLIC BP, Mean(SD)	75.7	(15.1)	75.7	(15.1)	75.7	(15.2)	0.97
PULSE, Mean(SD)	106.2	(19.7)	106.4	(19.7)	105.9	(19.7)	0.003
OXYGEN, Mean(SD)	95.9	(3.6)	95.9	(3.5)	95.9	(3.7)	0.97
RESPIRATION, Mean(SD)	19.6	(5.5)	19.6	(4.8)	19.6	(6.4)	0.77
PAININDEX							0.19
0	58424	(72.8)	35085	(72.9)	23339	(72.8)	
1	49	(0.1)	22	(0.0)	27	(0.1)	
2	314	(0.4)	187	(0.4)	127	(0.4)	
3	847	(1.1)	510	(1.1)	337	(1.1)	
4	2430	(3.0)	1444	(3.0)	986	(3.1)	
5	5315	(6.6)	3182	(6.6)	2133	(6.6)	
6	2749	(3.4)	1659	(3.4)	1090	(3.4)	
7	3867	(4.8)	2387	(5.0)	1480	(4.6)	
8	2549	(3.2)	1488	(3.1)	1061	(3.3)	
9	494	(0.6)	289	(0.6)	205	(0.6)	
10	596	(0.7)	351	(0.7)	245	(0.8)	
NA	2567	(3.2)	1516	(3.2)	1051	(3.3)	
HEIGHT, Mean(SD)	161.8	(10.4)	161.8	(10.5)	161.8	(10.2)	0.88
WEIGHT, Mean(SD)	61.1	(19.4)	61.1	(22.5)	61.1	(13.5)	0.73
BMI, Mean(SD)	23.2	(4.4)	23.2	(4.4)	23.2	(4.4)	0.79
GCS_TYPE							0.32
NA	6058	(7.6)	3595	(7.5)	2463	(7.7)	

clear_consciousness	65917	(82.2)	39514	(82.1)	26403	(82.3)	
minor_coma	1240	(1.5)	766	(1.6)	474	(1.5)	
moderate_coma	3553	(4.4)	2155	(4.5)	1398	(4.4)	
others	1686	(2.1)	1043	(2.2)	643	(2.0)	
severe_coma	1747	(2.2)	1047	(2.2)	700	(2.2)	
A00_ICD10	5	(0.0)	5	(0.0)	0	(0.0)	0.07
A01_ICD10	2	(0.0)	1	(0.0)	1	(0.0)	0.77
A02_ICD10	62	(0.1)	30	(0.1)	32	(0.1)	0.06
A03_ICD10	3	(0.0)	3	(0.0)	0	(0.0)	0.16
A04_ICD10	121	(0.2)	68	(0.1)	53	(0.2)	0.39
A05_ICD10	7	(0.0)	5	(0.0)	2	(0.0)	0.54
A06_ICD10	30	(0.0)	16	(0.0)	14	(0.0)	0.46
A07_ICD10	14	(0.0)	10	(0.0)	4	(0.0)	0.38
A08_ICD10	25	(0.0)	15	(0.0)	10	(0.0)	1
A09_ICD10	469	(0.6)	280	(0.6)	189	(0.6)	0.89
A15_ICD10	1248	(1.6)	746	(1.6)	502	(1.6)	0.87
A17_ICD10	41	(0.1)	31	(0.1)	10	(0.0)	0.04
A18_ICD10	152	(0.2)	91	(0.2)	61	(0.2)	0.97
A19_ICD10	54	(0.1)	32	(0.1)	22	(0.1)	0.91
A24_ICD10	4	(0.0)	2	(0.0)	2	(0.0)	0.68
A27_ICD10	95	(0.1)	63	(0.1)	32	(0.1)	0.21
A28_ICD10	8	(0.0)	6	(0.0)	2	(0.0)	0.39
A31_ICD10	288	(0.4)	177	(0.4)	111	(0.3)	0.61
A37_ICD10	6	(0.0)	5	(0.0)	1	(0.0)	0.24
A38_ICD10	1	(0.0)	1	(0.0)	0	(0.0)	0.41
A39_ICD10	3	(0.0)	2	(0.0)	1	(0.0)	0.81
A40_ICD10	23	(0.0)	14	(0.0)	9	(0.0)	0.93
A41_ICD10	873	(1.1)	525	(1.1)	348	(1.1)	0.93
A42_ICD10	6	(0.0)	2	(0.0)	4	(0.0)	0.18
A43_ICD10	3	(0.0)	2	(0.0)	1	(0.0)	0.81
A46_ICD10	32	(0.0)	17	(0.0)	15	(0.0)	0.43
A48_ICD10	52	(0.1)	30	(0.1)	22	(0.1)	0.73
A49_ICD10	37	(0.0)	21	(0.0)	16	(0.0)	0.69
A50_ICD10	1	(0.0)	1	(0.0)	0	(0.0)	0.41
A51_ICD10	27	(0.0)	16	(0.0)	11	(0.0)	0.94
A52_ICD10	28	(0.0)	12	(0.0)	16	(0.0)	0.06
A53_ICD10	244	(0.3)	131	(0.3)	113	(0.4)	0.04
A54_ICD10	25	(0.0)	17	(0.0)	8	(0.0)	0.41
A55_ICD10	5	(0.0)	2	(0.0)	3	(0.0)	0.36
A59_ICD10	10	(0.0)	6	(0.0)	4	(0.0)	1
A60_ICD10	66	(0.1)	35	(0.1)	31	(0.1)	0.25
A63_ICD10	60	(0.1)	37	(0.1)	23	(0.1)	0.79
A64_ICD10	11	(0.0)	6	(0.0)	5	(0.0)	0.71
A66_ICD10	11	(0.0)	8	(0.0)	3	(0.0)	0.39
A69_ICD10	15	(0.0)	12	(0.0)	3	(0.0)	0.11
A71_ICD10	25	(0.0)	15	(0.0)	10	(0.0)	1
A74_ICD10	7	(0.0)	3	(0.0)	4	(0.0)	0.35
A75_ICD10	125	(0.2)	82	(0.2)	43	(0.1)	0.2

A77_ICD10	1	(0.0)	1	(0.0)	0	(0.0)	0.41
A78_ICD10	60	(0.1)	38	(0.1)	22	(0.1)	0.6
A79_ICD10	8	(0.0)	7	(0.0)	1	(0.0)	0.11
A80_ICD10	9	(0.0)	6	(0.0)	3	(0.0)	0.68
A81_ICD10	14	(0.0)	10	(0.0)	4	(0.0)	0.38
A83_ICD10	21	(0.0)	13	(0.0)	8	(0.0)	0.86
A85_ICD10	2	(0.0)	1	(0.0)	1	(0.0)	0.77
A87_ICD10	5	(0.0)	4	(0.0)	1	(0.0)	0.36
A88_ICD10	6	(0.0)	4	(0.0)	2	(0.0)	0.74
A90_ICD10	22	(0.0)	15	(0.0)	7	(0.0)	0.43
A92_ICD10	4	(0.0)	3	(0.0)	1	(0.0)	0.54
A95_ICD10	1	(0.0)	1	(0.0)	0	(0.0)	0.41
A98_ICD10	24	(0.0)	17	(0.0)	7	(0.0)	0.28
B00_ICD10	879	(1.1)	511	(1.1)	368	(1.1)	0.26
B01_ICD10	35	(0.0)	27	(0.1)	8	(0.0)	0.04
B02_ICD10	2484	(3.1)	1502	(3.1)	982	(3.1)	0.63
B05_ICD10	4	(0.0)	4	(0.0)	0	(0.0)	0.1
B06_ICD10	3	(0.0)	3	(0.0)	0	(0.0)	0.16
B07_ICD10	838	(1.0)	502	(1.0)	336	(1.0)	0.96
B08_ICD10	36	(0.0)	18	(0.0)	18	(0.1)	0.22
B09_ICD10	13	(0.0)	9	(0.0)	4	(0.0)	0.5
B15_ICD10	19	(0.0)	10	(0.0)	9	(0.0)	0.51
B16_ICD10	969	(1.2)	591	(1.2)	378	(1.2)	0.53
B17_ICD10	799	(1.0)	464	(1.0)	335	(1.0)	0.26
B18_ICD10	4037	(5.0)	2440	(5.1)	1597	(5.0)	0.56
B19_ICD10	203	(0.3)	124	(0.3)	79	(0.2)	0.75
B20_ICD10	529	(0.7)	323	(0.7)	206	(0.6)	0.62
B25_ICD10	111	(0.1)	63	(0.1)	48	(0.1)	0.49
B26_ICD10	9	(0.0)	6	(0.0)	3	(0.0)	0.68
B27_ICD10	12	(0.0)	6	(0.0)	6	(0.0)	0.48
B30_ICD10	56	(0.1)	38	(0.1)	18	(0.1)	0.23
B33_ICD10	8	(0.0)	4	(0.0)	4	(0.0)	0.56
B34_ICD10	1	(0.0)	1	(0.0)	0	(0.0)	0.41
B35_ICD10	3672	(4.6)	2225	(4.6)	1447	(4.5)	0.45
B36_ICD10	252	(0.3)	147	(0.3)	105	(0.3)	0.59
B37_ICD10	1342	(1.7)	819	(1.7)	523	(1.6)	0.44
B38_ICD10	2	(0.0)	1	(0.0)	1	(0.0)	0.77
B39_ICD10	1	(0.0)	1	(0.0)	0	(0.0)	0.41
B44_ICD10	195	(0.2)	121	(0.3)	74	(0.2)	0.56
B45_ICD10	60	(0.1)	38	(0.1)	22	(0.1)	0.6
B46_ICD10	1	(0.0)	1	(0.0)	0	(0.0)	0.41
B48_ICD10	123	(0.2)	75	(0.2)	48	(0.1)	0.82
B49_ICD10	15	(0.0)	4	(0.0)	11	(0.0)	0.008
B54_ICD10	2	(0.0)	0	(0.0)	2	(0.0)	0.08
B57_ICD10	2	(0.0)	0	(0.0)	2	(0.0)	0.08
B58_ICD10	21	(0.0)	11	(0.0)	10	(0.0)	0.48
B59_ICD10	141	(0.2)	85	(0.2)	56	(0.2)	0.94
B60_ICD10	1	(0.0)	0	(0.0)	1	(0.0)	0.22

B65_ICD10	2	(0.0)	2	(0.0)	0	(0.0)	0.25
B66_ICD10	2	(0.0)	2	(0.0)	0	(0.0)	0.25
B68_ICD10	1	(0.0)	1	(0.0)	0	(0.0)	0.41
B75_ICD10	6	(0.0)	3	(0.0)	3	(0.0)	0.62
B79_ICD10	1	(0.0)	1	(0.0)	0	(0.0)	0.41
B83_ICD10	6	(0.0)	3	(0.0)	3	(0.0)	0.62
B85_ICD10	2	(0.0)	0	(0.0)	2	(0.0)	0.08
B86_ICD10	194	(0.2)	114	(0.2)	80	(0.2)	0.72
B89_ICD10	19	(0.0)	12	(0.0)	7	(0.0)	0.78
B90_ICD10	629	(0.8)	372	(0.8)	257	(0.8)	0.66
B91_ICD10	28	(0.0)	19	(0.0)	9	(0.0)	0.4
B94_ICD10	1	(0.0)	1	(0.0)	0	(0.0)	0.41
B95_ICD10	34	(0.0)	15	(0.0)	19	(0.1)	0.06
B96_ICD10	693	(0.9)	426	(0.9)	267	(0.8)	0.43
B97_ICD10	32	(0.0)	19	(0.0)	13	(0.0)	0.94
C00_ICD10	15	(0.0)	12	(0.0)	3	(0.0)	0.11
C01_ICD10	93	(0.1)	59	(0.1)	34	(0.1)	0.5
C02_ICD10	219	(0.3)	134	(0.3)	85	(0.3)	0.72
C03_ICD10	68	(0.1)	47	(0.1)	21	(0.1)	0.12
C04_ICD10	16	(0.0)	9	(0.0)	7	(0.0)	0.76
C05_ICD10	40	(0.0)	27	(0.1)	13	(0.0)	0.33
C06_ICD10	370	(0.5)	232	(0.5)	138	(0.4)	0.29
C07_ICD10	34	(0.0)	22	(0.0)	12	(0.0)	0.58
C08_ICD10	19	(0.0)	11	(0.0)	8	(0.0)	0.85
C09_ICD10	110	(0.1)	70	(0.1)	40	(0.1)	0.44
C10_ICD10	158	(0.2)	91	(0.2)	67	(0.2)	0.54
C11_ICD10	676	(0.8)	400	(0.8)	276	(0.9)	0.66
C12_ICD10	18	(0.0)	13	(0.0)	5	(0.0)	0.29
C13_ICD10	186	(0.2)	124	(0.3)	62	(0.2)	0.06
C14_ICD10	59	(0.1)	39	(0.1)	20	(0.1)	0.34
C15_ICD10	235	(0.3)	151	(0.3)	84	(0.3)	0.18
C16_ICD10	412	(0.5)	246	(0.5)	166	(0.5)	0.9
C17_ICD10	74	(0.1)	46	(0.1)	28	(0.1)	0.7
C18_ICD10	996	(1.2)	591	(1.2)	405	(1.3)	0.67
C19_ICD10	319	(0.4)	200	(0.4)	119	(0.4)	0.32
C20_ICD10	474	(0.6)	282	(0.6)	192	(0.6)	0.82
C21_ICD10	112	(0.1)	71	(0.1)	41	(0.1)	0.46
C22_ICD10	1695	(2.1)	1005	(2.1)	690	(2.2)	0.55
C23_ICD10	123	(0.2)	62	(0.1)	61	(0.2)	0.03
C24_ICD10	292	(0.4)	175	(0.4)	117	(0.4)	0.98
C25_ICD10	546	(0.7)	326	(0.7)	220	(0.7)	0.89
C26_ICD10	31	(0.0)	19	(0.0)	12	(0.0)	0.88
C30_ICD10	49	(0.1)	27	(0.1)	22	(0.1)	0.48
C31_ICD10	64	(0.1)	35	(0.1)	29	(0.1)	0.39
C32_ICD10	129	(0.2)	79	(0.2)	50	(0.2)	0.77
C33_ICD10	460	(0.6)	275	(0.6)	185	(0.6)	0.92
C34_ICD10	1531	(1.9)	926	(1.9)	605	(1.9)	0.7
C37_ICD10	68	(0.1)	53	(0.1)	15	(0.0)	0.003

C38_ICD10	54	(0.1)	35	(0.1)	19	(0.1)	0.47
C39_ICD10	12	(0.0)	7	(0.0)	5	(0.0)	0.91
C40_ICD10	18	(0.0)	12	(0.0)	6	(0.0)	0.56
C41_ICD10	75	(0.1)	39	(0.1)	36	(0.1)	0.16
C43_ICD10	51	(0.1)	34	(0.1)	17	(0.1)	0.33
C44_ICD10	36	(0.0)	21	(0.0)	15	(0.0)	0.84
C46_ICD10	39	(0.0)	22	(0.0)	17	(0.1)	0.65
C47_ICD10	11	(0.0)	5	(0.0)	6	(0.0)	0.32
C48_ICD10	68	(0.1)	40	(0.1)	28	(0.1)	0.84
C49_ICD10	185	(0.2)	113	(0.2)	72	(0.2)	0.76
C4A_ICD10	1	(0.0)	1	(0.0)	0	(0.0)	0.41
C50_ICD10	1399	(1.7)	848	(1.8)	551	(1.7)	0.64
C51_ICD10	17	(0.0)	12	(0.0)	5	(0.0)	0.37
C52_ICD10	20	(0.0)	11	(0.0)	9	(0.0)	0.65
C53_ICD10	337	(0.4)	199	(0.4)	138	(0.4)	0.72
C54_ICD10	224	(0.3)	132	(0.3)	92	(0.3)	0.74
C55_ICD10	70	(0.1)	43	(0.1)	27	(0.1)	0.81
C56_ICD10	262	(0.3)	159	(0.3)	103	(0.3)	0.82
C57_ICD10	19	(0.0)	11	(0.0)	8	(0.0)	0.85
C58_ICD10	1	(0.0)	1	(0.0)	0	(0.0)	0.41
C60_ICD10	2	(0.0)	1	(0.0)	1	(0.0)	0.77
C61_ICD10	657	(0.8)	411	(0.9)	246	(0.8)	0.18
C62_ICD10	14	(0.0)	13	(0.0)	1	(0.0)	0.01
C63_ICD10	1	(0.0)	1	(0.0)	0	(0.0)	0.41
C64_ICD10	299	(0.4)	158	(0.3)	141	(0.4)	0.01
C65_ICD10	110	(0.1)	57	(0.1)	53	(0.2)	0.08
C66_ICD10	136	(0.2)	75	(0.2)	61	(0.2)	0.25
C67_ICD10	435	(0.5)	258	(0.5)	177	(0.6)	0.77
C68_ICD10	60	(0.1)	31	(0.1)	29	(0.1)	0.19
C69_ICD10	29	(0.0)	21	(0.0)	8	(0.0)	0.17
C70_ICD10	6	(0.0)	5	(0.0)	1	(0.0)	0.24
C71_ICD10	144	(0.2)	86	(0.2)	58	(0.2)	0.95
C72_ICD10	14	(0.0)	10	(0.0)	4	(0.0)	0.38
C73_ICD10	194	(0.2)	125	(0.3)	69	(0.2)	0.21
C74_ICD10	13	(0.0)	7	(0.0)	6	(0.0)	0.65
C75_ICD10	33	(0.0)	17	(0.0)	16	(0.0)	0.32
C76_ICD10	29	(0.0)	21	(0.0)	8	(0.0)	0.17
C77_ICD10	40	(0.0)	28	(0.1)	12	(0.0)	0.2
C78_ICD10	268	(0.3)	164	(0.3)	104	(0.3)	0.69
C79_ICD10	740	(0.9)	456	(0.9)	284	(0.9)	0.37
C7A_ICD10	122	(0.2)	76	(0.2)	46	(0.1)	0.6
C7B_ICD10	4	(0.0)	2	(0.0)	2	(0.0)	0.68
C80_ICD10	7201	(9.0)	4310	(9.0)	2891	(9.0)	0.79
C81_ICD10	74	(0.1)	43	(0.1)	31	(0.1)	0.74
C82_ICD10	461	(0.6)	282	(0.6)	179	(0.6)	0.61
C83_ICD10	223	(0.3)	143	(0.3)	80	(0.2)	0.21
C84_ICD10	56	(0.1)	38	(0.1)	18	(0.1)	0.23
C85_ICD10	1159	(1.4)	722	(1.5)	437	(1.4)	0.11

C88_ICD10	18	(0.0)	10	(0.0)	8	(0.0)	0.7
C90_ICD10	374	(0.5)	214	(0.4)	160	(0.5)	0.27
C91_ICD10	413	(0.5)	253	(0.5)	160	(0.5)	0.6
C92_ICD10	831	(1.0)	489	(1.0)	342	(1.1)	0.49
C93_ICD10	25	(0.0)	17	(0.0)	8	(0.0)	0.41
C94_ICD10	21	(0.0)	11	(0.0)	10	(0.0)	0.48
C95_ICD10	343	(0.4)	207	(0.4)	136	(0.4)	0.89
C96_ICD10	90	(0.1)	57	(0.1)	33	(0.1)	0.52
D00_ICD10	2	(0.0)	1	(0.0)	1	(0.0)	0.77
D01_ICD10	4	(0.0)	3	(0.0)	1	(0.0)	0.54
D02_ICD10	9	(0.0)	6	(0.0)	3	(0.0)	0.68
D04_ICD10	48	(0.1)	27	(0.1)	21	(0.1)	0.6
D05_ICD10	121	(0.2)	78	(0.2)	43	(0.1)	0.32
D06_ICD10	156	(0.2)	80	(0.2)	76	(0.2)	0.03
D07_ICD10	3	(0.0)	1	(0.0)	2	(0.0)	0.35
D09_ICD10	6	(0.0)	3	(0.0)	3	(0.0)	0.62
D10_ICD10	66	(0.1)	40	(0.1)	26	(0.1)	0.92
D11_ICD10	7	(0.0)	5	(0.0)	2	(0.0)	0.54
D12_ICD10	1113	(1.4)	680	(1.4)	433	(1.3)	0.45
D13_ICD10	192	(0.2)	113	(0.2)	79	(0.2)	0.75
D14_ICD10	50	(0.1)	33	(0.1)	17	(0.1)	0.39
D15_ICD10	21	(0.0)	12	(0.0)	9	(0.0)	0.79
D16_ICD10	24	(0.0)	14	(0.0)	10	(0.0)	0.87
D17_ICD10	557	(0.7)	319	(0.7)	238	(0.7)	0.19
D18_ICD10	307	(0.4)	190	(0.4)	117	(0.4)	0.5
D19_ICD10	2	(0.0)	1	(0.0)	1	(0.0)	0.77
D20_ICD10	1	(0.0)	0	(0.0)	1	(0.0)	0.22
D21_ICD10	119	(0.1)	69	(0.1)	50	(0.2)	0.65
D22_ICD10	24	(0.0)	17	(0.0)	7	(0.0)	0.28
D23_ICD10	672	(0.8)	432	(0.9)	240	(0.7)	0.02
D24_ICD10	551	(0.7)	323	(0.7)	228	(0.7)	0.51
D25_ICD10	909	(1.1)	559	(1.2)	350	(1.1)	0.35
D26_ICD10	4	(0.0)	3	(0.0)	1	(0.0)	0.54
D27_ICD10	7	(0.0)	4	(0.0)	3	(0.0)	0.88
D28_ICD10	1	(0.0)	1	(0.0)	0	(0.0)	0.41
D29_ICD10	29	(0.0)	20	(0.0)	9	(0.0)	0.32
D30_ICD10	45	(0.1)	31	(0.1)	14	(0.0)	0.22
D31_ICD10	1	(0.0)	1	(0.0)	0	(0.0)	0.41
D32_ICD10	134	(0.2)	74	(0.2)	60	(0.2)	0.26
D33_ICD10	38	(0.0)	24	(0.0)	14	(0.0)	0.69
D34_ICD10	3	(0.0)	2	(0.0)	1	(0.0)	0.81
D35_ICD10	79	(0.1)	43	(0.1)	36	(0.1)	0.31
D36_ICD10	14	(0.0)	8	(0.0)	6	(0.0)	0.83
D37_ICD10	62	(0.1)	33	(0.1)	29	(0.1)	0.28
D38_ICD10	62	(0.1)	34	(0.1)	28	(0.1)	0.41
D3A_ICD10	3	(0.0)	2	(0.0)	1	(0.0)	0.81
D40_ICD10	1	(0.0)	1	(0.0)	0	(0.0)	0.41
D41_ICD10	7	(0.0)	2	(0.0)	5	(0.0)	0.09

D43_ICD10	5	(0.0)	4	(0.0)	1	(0.0)	0.36
D44_ICD10	2	(0.0)	1	(0.0)	1	(0.0)	0.77
D45_ICD10	30	(0.0)	19	(0.0)	11	(0.0)	0.71
D46_ICD10	114	(0.1)	65	(0.1)	49	(0.2)	0.52
D47_ICD10	81	(0.1)	50	(0.1)	31	(0.1)	0.75
D48_ICD10	51	(0.1)	25	(0.1)	26	(0.1)	0.11
D49_ICD10	10445	(13.0)	6227	(12.9)	4218	(13.1)	0.39
D50_ICD10	1203	(1.5)	719	(1.5)	484	(1.5)	0.87
D51_ICD10	145	(0.2)	85	(0.2)	60	(0.2)	0.73
D52_ICD10	32	(0.0)	16	(0.0)	16	(0.0)	0.25
D53_ICD10	39	(0.0)	27	(0.1)	12	(0.0)	0.24
D55_ICD10	6	(0.0)	1	(0.0)	5	(0.0)	0.03
D56_ICD10	64	(0.1)	39	(0.1)	25	(0.1)	0.88
D58_ICD10	80	(0.1)	52	(0.1)	28	(0.1)	0.36
D59_ICD10	133	(0.2)	78	(0.2)	55	(0.2)	0.75
D60_ICD10	3	(0.0)	1	(0.0)	2	(0.0)	0.35
D61_ICD10	287	(0.4)	160	(0.3)	127	(0.4)	0.14
D62_ICD10	6	(0.0)	4	(0.0)	2	(0.0)	0.74
D63_ICD10	357	(0.4)	208	(0.4)	149	(0.5)	0.5
D64_ICD10	4249	(5.3)	2579	(5.4)	1670	(5.2)	0.34
D65_ICD10	7	(0.0)	5	(0.0)	2	(0.0)	0.54
D66_ICD10	11	(0.0)	5	(0.0)	6	(0.0)	0.32
D67_ICD10	9	(0.0)	4	(0.0)	5	(0.0)	0.34
D68_ICD10	295	(0.4)	173	(0.4)	122	(0.4)	0.63
D69_ICD10	621	(0.8)	369	(0.8)	252	(0.8)	0.77
D70_ICD10	144	(0.2)	90	(0.2)	54	(0.2)	0.54
D71_ICD10	2	(0.0)	1	(0.0)	1	(0.0)	0.77
D72_ICD10	393	(0.5)	234	(0.5)	159	(0.5)	0.85
D73_ICD10	46	(0.1)	25	(0.1)	21	(0.1)	0.43
D75_ICD10	93	(0.1)	58	(0.1)	35	(0.1)	0.64
D76_ICD10	4	(0.0)	3	(0.0)	1	(0.0)	0.54
D78_ICD10	23	(0.0)	17	(0.0)	6	(0.0)	0.17
D80_ICD10	31	(0.0)	20	(0.0)	11	(0.0)	0.61
D82_ICD10	2	(0.0)	2	(0.0)	0	(0.0)	0.25
D83_ICD10	5	(0.0)	1	(0.0)	4	(0.0)	0.07
D84_ICD10	4	(0.0)	1	(0.0)	3	(0.0)	0.15
D86_ICD10	31	(0.0)	18	(0.0)	13	(0.0)	0.83
D89_ICD10	61	(0.1)	29	(0.1)	32	(0.1)	0.05
E00_ICD10	14	(0.0)	12	(0.0)	2	(0.0)	0.05
E01_ICD10	981	(1.2)	603	(1.3)	378	(1.2)	0.34
E03_ICD10	836	(1.0)	498	(1.0)	338	(1.1)	0.8
E04_ICD10	938	(1.2)	563	(1.2)	375	(1.2)	0.99
E05_ICD10	762	(1.0)	454	(0.9)	308	(1.0)	0.81
E06_ICD10	354	(0.4)	208	(0.4)	146	(0.5)	0.63
E07_ICD10	26	(0.0)	18	(0.0)	8	(0.0)	0.34
E08_ICD10	9130	(11.4)	5446	(11.3)	3684	(11.5)	0.47
E09_ICD10	7	(0.0)	4	(0.0)	3	(0.0)	0.88
E10_ICD10	97	(0.1)	54	(0.1)	43	(0.1)	0.38

E11_ICD10	8130	(10.1)	4883	(10.1)	3247	(10.1)	0.9
E13_ICD10	18	(0.0)	11	(0.0)	7	(0.0)	0.92
E15_ICD10	17	(0.0)	11	(0.0)	6	(0.0)	0.69
E16_ICD10	664	(0.8)	395	(0.8)	269	(0.8)	0.79
E20_ICD10	55	(0.1)	33	(0.1)	22	(0.1)	1
E21_ICD10	87	(0.1)	55	(0.1)	32	(0.1)	0.54
E22_ICD10	82	(0.1)	44	(0.1)	38	(0.1)	0.24
E23_ICD10	156	(0.2)	92	(0.2)	64	(0.2)	0.79
E24_ICD10	45	(0.1)	24	(0.0)	21	(0.1)	0.36
E25_ICD10	80	(0.1)	47	(0.1)	33	(0.1)	0.82
E26_ICD10	18	(0.0)	13	(0.0)	5	(0.0)	0.29
E27_ICD10	56	(0.1)	38	(0.1)	18	(0.1)	0.23
E28_ICD10	261	(0.3)	163	(0.3)	98	(0.3)	0.42
E29_ICD10	25	(0.0)	21	(0.0)	4	(0.0)	0.01
E30_ICD10	3	(0.0)	2	(0.0)	1	(0.0)	0.81
E31_ICD10	2	(0.0)	2	(0.0)	0	(0.0)	0.25
E34_ICD10	10	(0.0)	6	(0.0)	4	(0.0)	1
E41_ICD10	12	(0.0)	8	(0.0)	4	(0.0)	0.64
E43_ICD10	1	(0.0)	1	(0.0)	0	(0.0)	0.41
E44_ICD10	8	(0.0)	4	(0.0)	4	(0.0)	0.56
E45_ICD10	2	(0.0)	2	(0.0)	0	(0.0)	0.25
E46_ICD10	73	(0.1)	41	(0.1)	32	(0.1)	0.5
E50_ICD10	1	(0.0)	1	(0.0)	0	(0.0)	0.41
E53_ICD10	24	(0.0)	12	(0.0)	12	(0.0)	0.32
E55_ICD10	1	(0.0)	0	(0.0)	1	(0.0)	0.22
E56_ICD10	4	(0.0)	2	(0.0)	2	(0.0)	0.68
E63_ICD10	5	(0.0)	1	(0.0)	4	(0.0)	0.07
E66_ICD10	480	(0.6)	282	(0.6)	198	(0.6)	0.58
E67_ICD10	1	(0.0)	1	(0.0)	0	(0.0)	0.41
E70_ICD10	2	(0.0)	1	(0.0)	1	(0.0)	0.77
E72_ICD10	99	(0.1)	60	(0.1)	39	(0.1)	0.9
E74_ICD10	7	(0.0)	5	(0.0)	2	(0.0)	0.54
E75_ICD10	4	(0.0)	4	(0.0)	0	(0.0)	0.1
E76_ICD10	1	(0.0)	1	(0.0)	0	(0.0)	0.41
E78_ICD10	7507	(9.4)	4497	(9.3)	3010	(9.4)	0.86
E80_ICD10	8	(0.0)	4	(0.0)	4	(0.0)	0.56
E83_ICD10	234	(0.3)	132	(0.3)	102	(0.3)	0.26
E84_ICD10	1	(0.0)	0	(0.0)	1	(0.0)	0.22
E85_ICD10	13	(0.0)	10	(0.0)	3	(0.0)	0.21
E86_ICD10	5	(0.0)	1	(0.0)	4	(0.0)	0.07
E87_ICD10	1205	(1.5)	735	(1.5)	470	(1.5)	0.48
E88_ICD10	3	(0.0)	2	(0.0)	1	(0.0)	0.81
E89_ICD10	15	(0.0)	10	(0.0)	5	(0.0)	0.6
F01_ICD10	1231	(1.5)	750	(1.6)	481	(1.5)	0.5
F02_ICD10	1215	(1.5)	723	(1.5)	492	(1.5)	0.72
F03_ICD10	1678	(2.1)	994	(2.1)	684	(2.1)	0.52
F04_ICD10	18	(0.0)	9	(0.0)	9	(0.0)	0.39
F05_ICD10	476	(0.6)	286	(0.6)	190	(0.6)	0.97

F06_ICD10	309	(0.4)	181	(0.4)	128	(0.4)	0.61
F07_ICD10	169	(0.2)	102	(0.2)	67	(0.2)	0.92
F09_ICD10	25	(0.0)	16	(0.0)	9	(0.0)	0.68
F10_ICD10	138	(0.2)	91	(0.2)	47	(0.1)	0.15
F11_ICD10	6	(0.0)	4	(0.0)	2	(0.0)	0.74
F13_ICD10	18	(0.0)	11	(0.0)	7	(0.0)	0.92
F15_ICD10	22	(0.0)	10	(0.0)	12	(0.0)	0.16
F17_ICD10	136	(0.2)	90	(0.2)	46	(0.1)	0.14
F19_ICD10	26	(0.0)	17	(0.0)	9	(0.0)	0.58
F20_ICD10	192	(0.2)	114	(0.2)	78	(0.2)	0.86
F21_ICD10	2	(0.0)	2	(0.0)	0	(0.0)	0.25
F22_ICD10	50	(0.1)	27	(0.1)	23	(0.1)	0.39
F23_ICD10	56	(0.1)	34	(0.1)	22	(0.1)	0.91
F25_ICD10	20	(0.0)	12	(0.0)	8	(0.0)	1
F28_ICD10	1	(0.0)	1	(0.0)	0	(0.0)	0.41
F29_ICD10	60	(0.1)	33	(0.1)	27	(0.1)	0.43
F30_ICD10	97	(0.1)	49	(0.1)	48	(0.1)	0.06
F31_ICD10	194	(0.2)	98	(0.2)	96	(0.3)	0.007
F32_ICD10	1399	(1.7)	842	(1.7)	557	(1.7)	0.89
F33_ICD10	293	(0.4)	165	(0.3)	128	(0.4)	0.2
F34_ICD10	845	(1.1)	468	(1.0)	377	(1.2)	0.006
F39_ICD10	79	(0.1)	40	(0.1)	39	(0.1)	0.09
F40_ICD10	38	(0.0)	25	(0.1)	13	(0.0)	0.47
F41_ICD10	2066	(2.6)	1258	(2.6)	808	(2.5)	0.4
F42_ICD10	35	(0.0)	18	(0.0)	17	(0.1)	0.3
F43_ICD10	916	(1.1)	536	(1.1)	380	(1.2)	0.36
F44_ICD10	8	(0.0)	7	(0.0)	1	(0.0)	0.11
F45_ICD10	408	(0.5)	228	(0.5)	180	(0.6)	0.09
F48_ICD10	200	(0.2)	119	(0.2)	81	(0.3)	0.89
F50_ICD10	20	(0.0)	12	(0.0)	8	(0.0)	1
F51_ICD10	5068	(6.3)	3074	(6.4)	1994	(6.2)	0.32
F52_ICD10	16	(0.0)	14	(0.0)	2	(0.0)	0.02
F54_ICD10	2	(0.0)	2	(0.0)	0	(0.0)	0.25
F60_ICD10	39	(0.0)	26	(0.1)	13	(0.0)	0.4
F63_ICD10	6	(0.0)	6	(0.0)	0	(0.0)	0.05
F64_ICD10	1	(0.0)	0	(0.0)	1	(0.0)	0.22
F68_ICD10	1	(0.0)	1	(0.0)	0	(0.0)	0.41
F69_ICD10	7	(0.0)	2	(0.0)	5	(0.0)	0.09
F70_ICD10	7	(0.0)	2	(0.0)	5	(0.0)	0.09
F71_ICD10	5	(0.0)	1	(0.0)	4	(0.0)	0.07
F72_ICD10	4	(0.0)	2	(0.0)	2	(0.0)	0.68
F73_ICD10	2	(0.0)	1	(0.0)	1	(0.0)	0.77
F79_ICD10	51	(0.1)	35	(0.1)	16	(0.0)	0.21
F80_ICD10	13	(0.0)	10	(0.0)	3	(0.0)	0.21
F81_ICD10	1	(0.0)	0	(0.0)	1	(0.0)	0.22
F84_ICD10	19	(0.0)	10	(0.0)	9	(0.0)	0.51
F88_ICD10	2	(0.0)	1	(0.0)	1	(0.0)	0.77
F90_ICD10	29	(0.0)	22	(0.0)	7	(0.0)	0.08

F91_ICD10	8	(0.0)	6	(0.0)	2	(0.0)	0.39
F93_ICD10	23	(0.0)	16	(0.0)	7	(0.0)	0.35
F95_ICD10	4	(0.0)	1	(0.0)	3	(0.0)	0.15
F98_ICD10	24	(0.0)	17	(0.0)	7	(0.0)	0.28
G00_ICD10	24	(0.0)	20	(0.0)	4	(0.0)	0.02
G02_ICD10	1	(0.0)	0	(0.0)	1	(0.0)	0.22
G03_ICD10	122	(0.2)	69	(0.1)	53	(0.2)	0.44
G04_ICD10	105	(0.1)	64	(0.1)	41	(0.1)	0.84
G05_ICD10	2	(0.0)	2	(0.0)	0	(0.0)	0.25
G06_ICD10	44	(0.1)	30	(0.1)	14	(0.0)	0.27
G08_ICD10	18	(0.0)	13	(0.0)	5	(0.0)	0.29
G09_ICD10	1	(0.0)	0	(0.0)	1	(0.0)	0.22
G10_ICD10	6	(0.0)	5	(0.0)	1	(0.0)	0.24
G11_ICD10	60	(0.1)	33	(0.1)	27	(0.1)	0.43
G12_ICD10	52	(0.1)	29	(0.1)	23	(0.1)	0.53
G14_ICD10	2	(0.0)	1	(0.0)	1	(0.0)	0.77
G20_ICD10	1614	(2.0)	953	(2.0)	661	(2.1)	0.43
G21_ICD10	443	(0.6)	266	(0.6)	177	(0.6)	0.98
G23_ICD10	130	(0.2)	83	(0.2)	47	(0.1)	0.37
G24_ICD10	163	(0.2)	100	(0.2)	63	(0.2)	0.72
G25_ICD10	438	(0.5)	247	(0.5)	191	(0.6)	0.12
G30_ICD10	137	(0.2)	78	(0.2)	59	(0.2)	0.46
G31_ICD10	171	(0.2)	97	(0.2)	74	(0.2)	0.38
G32_ICD10	52	(0.1)	36	(0.1)	16	(0.0)	0.17
G35_ICD10	72	(0.1)	45	(0.1)	27	(0.1)	0.66
G36_ICD10	13	(0.0)	8	(0.0)	5	(0.0)	0.91
G37_ICD10	14	(0.0)	11	(0.0)	3	(0.0)	0.16
G40_ICD10	673	(0.8)	417	(0.9)	256	(0.8)	0.3
G43_ICD10	280	(0.3)	163	(0.3)	117	(0.4)	0.54
G44_ICD10	2050	(2.6)	1265	(2.6)	785	(2.4)	0.11
G45_ICD10	732	(0.9)	422	(0.9)	310	(1.0)	0.19
G47_ICD10	2845	(3.5)	1727	(3.6)	1118	(3.5)	0.44
G50_ICD10	151	(0.2)	88	(0.2)	63	(0.2)	0.67
G51_ICD10	308	(0.4)	191	(0.4)	117	(0.4)	0.47
G52_ICD10	28	(0.0)	17	(0.0)	11	(0.0)	0.94
G54_ICD10	303	(0.4)	184	(0.4)	119	(0.4)	0.8
G56_ICD10	705	(0.9)	444	(0.9)	261	(0.8)	0.1
G57_ICD10	68	(0.1)	53	(0.1)	15	(0.0)	0.003
G58_ICD10	604	(0.8)	366	(0.8)	238	(0.7)	0.76
G60_ICD10	629	(0.8)	385	(0.8)	244	(0.8)	0.53
G61_ICD10	35	(0.0)	18	(0.0)	17	(0.1)	0.3
G62_ICD10	30	(0.0)	15	(0.0)	15	(0.0)	0.26
G63_ICD10	51	(0.1)	32	(0.1)	19	(0.1)	0.69
G70_ICD10	16	(0.0)	7	(0.0)	9	(0.0)	0.18
G71_ICD10	14	(0.0)	8	(0.0)	6	(0.0)	0.83
G72_ICD10	79	(0.1)	51	(0.1)	28	(0.1)	0.41
G73_ICD10	9	(0.0)	4	(0.0)	5	(0.0)	0.34
G80_ICD10	70	(0.1)	45	(0.1)	25	(0.1)	0.46

G81_ICD10	109	(0.1)	68	(0.1)	41	(0.1)	0.61
G82_ICD10	36	(0.0)	19	(0.0)	17	(0.1)	0.38
G83_ICD10	133	(0.2)	73	(0.2)	60	(0.2)	0.23
G89_ICD10	7	(0.0)	4	(0.0)	3	(0.0)	0.88
G90_ICD10	37	(0.0)	21	(0.0)	16	(0.0)	0.69
G91_ICD10	343	(0.4)	209	(0.4)	134	(0.4)	0.72
G93_ICD10	809	(1.0)	499	(1.0)	310	(1.0)	0.33
G95_ICD10	103	(0.1)	61	(0.1)	42	(0.1)	0.87
G96_ICD10	10	(0.0)	7	(0.0)	3	(0.0)	0.52
G97_ICD10	1	(0.0)	0	(0.0)	1	(0.0)	0.22
G99_ICD10	64	(0.1)	35	(0.1)	29	(0.1)	0.39
H00_ICD10	242	(0.3)	137	(0.3)	105	(0.3)	0.28
H01_ICD10	96	(0.1)	53	(0.1)	43	(0.1)	0.34
H02_ICD10	877	(1.1)	542	(1.1)	335	(1.0)	0.27
H04_ICD10	2638	(3.3)	1592	(3.3)	1046	(3.3)	0.71
H05_ICD10	42	(0.1)	28	(0.1)	14	(0.0)	0.38
H10_ICD10	6875	(8.6)	4103	(8.5)	2772	(8.6)	0.57
H11_ICD10	660	(0.8)	378	(0.8)	282	(0.9)	0.15
H15_ICD10	40	(0.0)	27	(0.1)	13	(0.0)	0.33
H16_ICD10	274	(0.3)	163	(0.3)	111	(0.3)	0.86
H17_ICD10	164	(0.2)	103	(0.2)	61	(0.2)	0.46
H18_ICD10	928	(1.2)	552	(1.1)	376	(1.2)	0.75
H20_ICD10	57	(0.1)	36	(0.1)	21	(0.1)	0.63
H21_ICD10	14	(0.0)	8	(0.0)	6	(0.0)	0.83
H25_ICD10	1260	(1.6)	768	(1.6)	492	(1.5)	0.49
H26_ICD10	5088	(6.3)	3051	(6.3)	2037	(6.3)	0.96
H27_ICD10	52	(0.1)	33	(0.1)	19	(0.1)	0.61
H28_ICD10	1	(0.0)	0	(0.0)	1	(0.0)	0.22
H30_ICD10	24	(0.0)	13	(0.0)	11	(0.0)	0.56
H31_ICD10	24	(0.0)	14	(0.0)	10	(0.0)	0.87
H33_ICD10	308	(0.4)	186	(0.4)	122	(0.4)	0.89
H34_ICD10	201	(0.3)	119	(0.2)	82	(0.3)	0.82
H35_ICD10	1992	(2.5)	1194	(2.5)	798	(2.5)	0.96
H36_ICD10	2	(0.0)	1	(0.0)	1	(0.0)	0.77
H40_ICD10	1933	(2.4)	1143	(2.4)	790	(2.5)	0.43
H43_ICD10	975	(1.2)	604	(1.3)	371	(1.2)	0.21
H44_ICD10	252	(0.3)	154	(0.3)	98	(0.3)	0.72
H46_ICD10	48	(0.1)	34	(0.1)	14	(0.0)	0.13
H47_ICD10	344	(0.4)	220	(0.5)	124	(0.4)	0.13
H49_ICD10	112	(0.1)	66	(0.1)	46	(0.1)	0.82
H50_ICD10	144	(0.2)	99	(0.2)	45	(0.1)	0.03
H51_ICD10	16	(0.0)	10	(0.0)	6	(0.0)	0.84
H52_ICD10	1329	(1.7)	810	(1.7)	519	(1.6)	0.48
H53_ICD10	3193	(4.0)	1958	(4.1)	1235	(3.8)	0.12
H54_ICD10	14	(0.0)	10	(0.0)	4	(0.0)	0.38
H55_ICD10	9	(0.0)	5	(0.0)	4	(0.0)	0.79
H57_ICD10	23	(0.0)	16	(0.0)	7	(0.0)	0.35
H60_ICD10	701	(0.9)	417	(0.9)	284	(0.9)	0.78

H61_ICD10	596	(0.7)	335	(0.7)	261	(0.8)	0.06
H62_ICD10	10	(0.0)	8	(0.0)	2	(0.0)	0.2
H65_ICD10	420	(0.5)	246	(0.5)	174	(0.5)	0.55
H66_ICD10	691	(0.9)	433	(0.9)	258	(0.8)	0.15
H69_ICD10	121	(0.2)	69	(0.1)	52	(0.2)	0.5
H70_ICD10	18	(0.0)	13	(0.0)	5	(0.0)	0.29
H71_ICD10	30	(0.0)	14	(0.0)	16	(0.0)	0.14
H72_ICD10	2	(0.0)	2	(0.0)	0	(0.0)	0.25
H73_ICD10	13	(0.0)	8	(0.0)	5	(0.0)	0.91
H80_ICD10	15	(0.0)	11	(0.0)	4	(0.0)	0.29
H81_ICD10	597	(0.7)	353	(0.7)	244	(0.8)	0.66
H83_ICD10	2537	(3.2)	1515	(3.1)	1022	(3.2)	0.77
H90_ICD10	120	(0.1)	74	(0.2)	46	(0.1)	0.71
H91_ICD10	1494	(1.9)	917	(1.9)	577	(1.8)	0.27
H92_ICD10	32	(0.0)	17	(0.0)	15	(0.0)	0.43
H93_ICD10	825	(1.0)	499	(1.0)	326	(1.0)	0.77
H95_ICD10	1	(0.0)	1	(0.0)	0	(0.0)	0.41
I00_ICD10	2	(0.0)	2	(0.0)	0	(0.0)	0.25
I02_ICD10	1	(0.0)	1	(0.0)	0	(0.0)	0.41
I05_ICD10	193	(0.2)	119	(0.2)	74	(0.2)	0.64
I06_ICD10	88	(0.1)	48	(0.1)	40	(0.1)	0.3
I07_ICD10	43	(0.1)	28	(0.1)	15	(0.0)	0.49
I08_ICD10	53	(0.1)	37	(0.1)	16	(0.0)	0.14
I09_ICD10	113	(0.1)	67	(0.1)	46	(0.1)	0.88
I10_ICD10	13992	(17.4)	8408	(17.5)	5584	(17.4)	0.81
I11_ICD10	1042	(1.3)	592	(1.2)	450	(1.4)	0.03
I12_ICD10	65	(0.1)	39	(0.1)	26	(0.1)	1
I13_ICD10	24	(0.0)	16	(0.0)	8	(0.0)	0.5
I15_ICD10	63	(0.1)	38	(0.1)	25	(0.1)	0.96
I20_ICD10	561	(0.7)	347	(0.7)	214	(0.7)	0.37
I21_ICD10	667	(0.8)	426	(0.9)	241	(0.8)	0.04
I24_ICD10	2648	(3.3)	1586	(3.3)	1062	(3.3)	0.91
I25_ICD10	5635	(7.0)	3396	(7.1)	2239	(7.0)	0.67
I26_ICD10	165	(0.2)	103	(0.2)	62	(0.2)	0.52
I27_ICD10	203	(0.3)	128	(0.3)	75	(0.2)	0.37
I28_ICD10	5	(0.0)	3	(0.0)	2	(0.0)	1
I30_ICD10	8	(0.0)	6	(0.0)	2	(0.0)	0.39
I31_ICD10	59	(0.1)	34	(0.1)	25	(0.1)	0.71
I32_ICD10	4	(0.0)	2	(0.0)	2	(0.0)	0.68
I33_ICD10	144	(0.2)	97	(0.2)	47	(0.1)	0.07
I34_ICD10	838	(1.0)	490	(1.0)	348	(1.1)	0.36
I35_ICD10	356	(0.4)	216	(0.4)	140	(0.4)	0.79
I36_ICD10	11	(0.0)	6	(0.0)	5	(0.0)	0.71
I37_ICD10	3	(0.0)	2	(0.0)	1	(0.0)	0.81
I38_ICD10	228	(0.3)	135	(0.3)	93	(0.3)	0.81
I39_ICD10	9	(0.0)	3	(0.0)	6	(0.0)	0.1
I40_ICD10	6	(0.0)	3	(0.0)	3	(0.0)	0.62
I42_ICD10	115	(0.1)	83	(0.2)	32	(0.1)	0.008

I43_ICD10	9	(0.0)	5	(0.0)	4	(0.0)	0.79
I44_ICD10	169	(0.2)	102	(0.2)	67	(0.2)	0.92
I45_ICD10	41	(0.1)	27	(0.1)	14	(0.0)	0.44
I46_ICD10	88	(0.1)	59	(0.1)	29	(0.1)	0.18
I47_ICD10	386	(0.5)	234	(0.5)	152	(0.5)	0.8
I48_ICD10	1717	(2.1)	1044	(2.2)	673	(2.1)	0.49
I49_ICD10	3171	(4.0)	1876	(3.9)	1295	(4.0)	0.33
I50_ICD10	3044	(3.8)	1805	(3.8)	1239	(3.9)	0.42
I51_ICD10	3707	(4.6)	2231	(4.6)	1476	(4.6)	0.81
I60_ICD10	130	(0.2)	86	(0.2)	44	(0.1)	0.15
I61_ICD10	550	(0.7)	339	(0.7)	211	(0.7)	0.43
I62_ICD10	462	(0.6)	294	(0.6)	168	(0.5)	0.11
I63_ICD10	3392	(4.2)	2034	(4.2)	1358	(4.2)	0.97
I65_ICD10	319	(0.4)	198	(0.4)	121	(0.4)	0.45
I66_ICD10	269	(0.3)	161	(0.3)	108	(0.3)	0.96
I67_ICD10	5111	(6.4)	3051	(6.3)	2060	(6.4)	0.65
I69_ICD10	1619	(2.0)	993	(2.1)	626	(2.0)	0.27
I70_ICD10	353	(0.4)	213	(0.4)	140	(0.4)	0.9
I71_ICD10	358	(0.4)	217	(0.5)	141	(0.4)	0.81
I72_ICD10	109	(0.1)	63	(0.1)	46	(0.1)	0.64
I73_ICD10	1217	(1.5)	716	(1.5)	501	(1.6)	0.4
I74_ICD10	939	(1.2)	551	(1.1)	388	(1.2)	0.41
I77_ICD10	513	(0.6)	286	(0.6)	227	(0.7)	0.05
I78_ICD10	5	(0.0)	4	(0.0)	1	(0.0)	0.36
I79_ICD10	19	(0.0)	12	(0.0)	7	(0.0)	0.78
I80_ICD10	450	(0.6)	268	(0.6)	182	(0.6)	0.85
I81_ICD10	10	(0.0)	6	(0.0)	4	(0.0)	1
I82_ICD10	65	(0.1)	36	(0.1)	29	(0.1)	0.45
I83_ICD10	625	(0.8)	374	(0.8)	251	(0.8)	0.93
I85_ICD10	381	(0.5)	222	(0.5)	159	(0.5)	0.49
I86_ICD10	131	(0.2)	70	(0.1)	61	(0.2)	0.12
I87_ICD10	20	(0.0)	13	(0.0)	7	(0.0)	0.65
I88_ICD10	150	(0.2)	86	(0.2)	64	(0.2)	0.5
I89_ICD10	152	(0.2)	89	(0.2)	63	(0.2)	0.72
I95_ICD10	268	(0.3)	165	(0.3)	103	(0.3)	0.6
I96_ICD10	62	(0.1)	40	(0.1)	22	(0.1)	0.47
I97_ICD10	130	(0.2)	83	(0.2)	47	(0.1)	0.37
I99_ICD10	1	(0.0)	1	(0.0)	0	(0.0)	0.41
J00_ICD10	1547	(1.9)	919	(1.9)	628	(2.0)	0.63
J01_ICD10	1215	(1.5)	752	(1.6)	463	(1.4)	0.17
J02_ICD10	1439	(1.8)	844	(1.8)	595	(1.9)	0.29
J03_ICD10	645	(0.8)	386	(0.8)	259	(0.8)	0.94
J04_ICD10	29	(0.0)	18	(0.0)	11	(0.0)	0.82
J05_ICD10	39	(0.0)	24	(0.0)	15	(0.0)	0.84
J06_ICD10	9351	(11.7)	5671	(11.8)	3680	(11.5)	0.17
J09_ICD10	7	(0.0)	4	(0.0)	3	(0.0)	0.88
J10_ICD10	504	(0.6)	310	(0.6)	194	(0.6)	0.49
J11_ICD10	436	(0.5)	263	(0.5)	173	(0.5)	0.89

J12_ICD10	8	(0.0)	4	(0.0)	4	(0.0)	0.56
J13_ICD10	49	(0.1)	33	(0.1)	16	(0.0)	0.29
J14_ICD10	2	(0.0)	0	(0.0)	2	(0.0)	0.08
J15_ICD10	697	(0.9)	432	(0.9)	265	(0.8)	0.28
J16_ICD10	24	(0.0)	9	(0.0)	15	(0.0)	0.02
J17_ICD10	14	(0.0)	6	(0.0)	8	(0.0)	0.19
J18_ICD10	7423	(9.3)	4469	(9.3)	2954	(9.2)	0.7
J20_ICD10	1871	(2.3)	1132	(2.4)	739	(2.3)	0.65
J21_ICD10	306	(0.4)	177	(0.4)	129	(0.4)	0.44
J30_ICD10	4122	(5.1)	2512	(5.2)	1610	(5.0)	0.2
J31_ICD10	1232	(1.5)	742	(1.5)	490	(1.5)	0.87
J32_ICD10	1030	(1.3)	611	(1.3)	419	(1.3)	0.65
J33_ICD10	50	(0.1)	29	(0.1)	21	(0.1)	0.77
J34_ICD10	148	(0.2)	93	(0.2)	55	(0.2)	0.48
J35_ICD10	84	(0.1)	52	(0.1)	32	(0.1)	0.72
J36_ICD10	35	(0.0)	22	(0.0)	13	(0.0)	0.73
J37_ICD10	54	(0.1)	38	(0.1)	16	(0.0)	0.12
J38_ICD10	843	(1.1)	511	(1.1)	332	(1.0)	0.71
J39_ICD10	176	(0.2)	108	(0.2)	68	(0.2)	0.71
J40_ICD10	3266	(4.1)	1994	(4.1)	1272	(4.0)	0.21
J41_ICD10	13	(0.0)	9	(0.0)	4	(0.0)	0.5
J42_ICD10	774	(1.0)	445	(0.9)	329	(1.0)	0.15
J43_ICD10	123	(0.2)	74	(0.2)	49	(0.2)	0.97
J44_ICD10	3201	(4.0)	1953	(4.1)	1248	(3.9)	0.23
J45_ICD10	1888	(2.4)	1167	(2.4)	721	(2.2)	0.1
J47_ICD10	843	(1.1)	530	(1.1)	313	(1.0)	0.09
J60_ICD10	41	(0.1)	29	(0.1)	12	(0.0)	0.16
J62_ICD10	3	(0.0)	2	(0.0)	1	(0.0)	0.81
J63_ICD10	2	(0.0)	0	(0.0)	2	(0.0)	0.08
J64_ICD10	46	(0.1)	32	(0.1)	14	(0.0)	0.19
J67_ICD10	5	(0.0)	5	(0.0)	0	(0.0)	0.07
J68_ICD10	6	(0.0)	4	(0.0)	2	(0.0)	0.74
J69_ICD10	676	(0.8)	422	(0.9)	254	(0.8)	0.2
J70_ICD10	4	(0.0)	4	(0.0)	0	(0.0)	0.1
J80_ICD10	31	(0.0)	19	(0.0)	12	(0.0)	0.88
J81_ICD10	55	(0.1)	28	(0.1)	27	(0.1)	0.17
J82_ICD10	2	(0.0)	2	(0.0)	0	(0.0)	0.25
J84_ICD10	406	(0.5)	247	(0.5)	159	(0.5)	0.73
J85_ICD10	115	(0.1)	66	(0.1)	49	(0.2)	0.57
J86_ICD10	175	(0.2)	104	(0.2)	71	(0.2)	0.88
J90_ICD10	131	(0.2)	81	(0.2)	50	(0.2)	0.67
J91_ICD10	623	(0.8)	351	(0.7)	272	(0.8)	0.06
J93_ICD10	36	(0.0)	21	(0.0)	15	(0.0)	0.84
J94_ICD10	4	(0.0)	2	(0.0)	2	(0.0)	0.68
J95_ICD10	76	(0.1)	50	(0.1)	26	(0.1)	0.3
J96_ICD10	630	(0.8)	382	(0.8)	248	(0.8)	0.74
J98_ICD10	151	(0.2)	90	(0.2)	61	(0.2)	0.92
J99_ICD10	2	(0.0)	0	(0.0)	2	(0.0)	0.08

K00_ICD10	471	(0.6)	297	(0.6)	174	(0.5)	0.17
K01_ICD10	49	(0.1)	29	(0.1)	20	(0.1)	0.91
K02_ICD10	377	(0.5)	249	(0.5)	128	(0.4)	0.02
K03_ICD10	103	(0.1)	67	(0.1)	36	(0.1)	0.3
K04_ICD10	2079	(2.6)	1241	(2.6)	838	(2.6)	0.77
K05_ICD10	679	(0.8)	430	(0.9)	249	(0.8)	0.08
K06_ICD10	1	(0.0)	0	(0.0)	1	(0.0)	0.22
K08_ICD10	1923	(2.4)	1124	(2.3)	799	(2.5)	0.16
K09_ICD10	19	(0.0)	10	(0.0)	9	(0.0)	0.51
K11_ICD10	483	(0.6)	308	(0.6)	175	(0.5)	0.09
K12_ICD10	978	(1.2)	568	(1.2)	410	(1.3)	0.22
K13_ICD10	2124	(2.6)	1284	(2.7)	840	(2.6)	0.67
K14_ICD10	309	(0.4)	196	(0.4)	113	(0.4)	0.22
K20_ICD10	347	(0.4)	209	(0.4)	138	(0.4)	0.93
K21_ICD10	5222	(6.5)	3134	(6.5)	2088	(6.5)	0.98
K22_ICD10	96	(0.1)	58	(0.1)	38	(0.1)	0.93
K25_ICD10	2378	(3.0)	1451	(3.0)	927	(2.9)	0.3
K26_ICD10	1255	(1.6)	736	(1.5)	519	(1.6)	0.32
K27_ICD10	2133	(2.7)	1320	(2.7)	813	(2.5)	0.07
K28_ICD10	10	(0.0)	9	(0.0)	1	(0.0)	0.05
K29_ICD10	7066	(8.8)	4253	(8.8)	2813	(8.8)	0.73
K30_ICD10	1409	(1.8)	844	(1.8)	565	(1.8)	0.94
K31_ICD10	4095	(5.1)	2507	(5.2)	1588	(4.9)	0.1
K35_ICD10	263	(0.3)	181	(0.4)	82	(0.3)	0.003
K36_ICD10	12	(0.0)	11	(0.0)	1	(0.0)	0.03
K37_ICD10	117	(0.1)	75	(0.2)	42	(0.1)	0.36
K38_ICD10	1	(0.0)	0	(0.0)	1	(0.0)	0.22
K40_ICD10	647	(0.8)	384	(0.8)	263	(0.8)	0.74
K41_ICD10	4	(0.0)	3	(0.0)	1	(0.0)	0.54
K42_ICD10	32	(0.0)	18	(0.0)	14	(0.0)	0.67
K43_ICD10	135	(0.2)	78	(0.2)	57	(0.2)	0.6
K44_ICD10	73	(0.1)	42	(0.1)	31	(0.1)	0.67
K45_ICD10	15	(0.0)	8	(0.0)	7	(0.0)	0.6
K46_ICD10	233	(0.3)	144	(0.3)	89	(0.3)	0.57
K50_ICD10	94	(0.1)	53	(0.1)	41	(0.1)	0.47
K51_ICD10	116	(0.1)	68	(0.1)	48	(0.1)	0.76
K52_ICD10	3311	(4.1)	2057	(4.3)	1254	(3.9)	0.01
K55_ICD10	54	(0.1)	32	(0.1)	22	(0.1)	0.91
K56_ICD10	1054	(1.3)	642	(1.3)	412	(1.3)	0.54
K57_ICD10	212	(0.3)	131	(0.3)	81	(0.3)	0.59
K58_ICD10	541	(0.7)	333	(0.7)	208	(0.6)	0.46
K59_ICD10	1313	(1.6)	793	(1.6)	520	(1.6)	0.77
K60_ICD10	193	(0.2)	114	(0.2)	79	(0.2)	0.79
K61_ICD10	213	(0.3)	115	(0.2)	98	(0.3)	0.07
K62_ICD10	259	(0.3)	152	(0.3)	107	(0.3)	0.67
K63_ICD10	739	(0.9)	446	(0.9)	293	(0.9)	0.84
K64_ICD10	3071	(3.8)	1849	(3.8)	1222	(3.8)	0.81
K65_ICD10	225	(0.3)	126	(0.3)	99	(0.3)	0.22

K66_ICD10	9	(0.0)	8	(0.0)	1	(0.0)	0.08
K67_ICD10	45	(0.1)	30	(0.1)	15	(0.0)	0.36
K68_ICD10	203	(0.3)	128	(0.3)	75	(0.2)	0.37
K70_ICD10	241	(0.3)	153	(0.3)	88	(0.3)	0.27
K71_ICD10	360	(0.4)	208	(0.4)	152	(0.5)	0.39
K72_ICD10	354	(0.4)	220	(0.5)	134	(0.4)	0.41
K73_ICD10	1269	(1.6)	757	(1.6)	512	(1.6)	0.8
K74_ICD10	2080	(2.6)	1231	(2.6)	849	(2.6)	0.44
K75_ICD10	495	(0.6)	281	(0.6)	214	(0.7)	0.14
K76_ICD10	1416	(1.8)	885	(1.8)	531	(1.7)	0.05
K77_ICD10	2	(0.0)	0	(0.0)	2	(0.0)	0.08
K80_ICD10	2391	(3.0)	1437	(3.0)	954	(3.0)	0.92
K81_ICD10	728	(0.9)	428	(0.9)	300	(0.9)	0.5
K82_ICD10	306	(0.4)	181	(0.4)	125	(0.4)	0.76
K83_ICD10	1559	(1.9)	921	(1.9)	638	(2.0)	0.45
K85_ICD10	464	(0.6)	286	(0.6)	178	(0.6)	0.47
K86_ICD10	653	(0.8)	380	(0.8)	273	(0.9)	0.34
K90_ICD10	46	(0.1)	26	(0.1)	20	(0.1)	0.63
K91_ICD10	56	(0.1)	31	(0.1)	25	(0.1)	0.48
K92_ICD10	4146	(5.2)	2524	(5.2)	1622	(5.1)	0.24
K94_ICD10	72	(0.1)	43	(0.1)	29	(0.1)	0.96
L01_ICD10	19	(0.0)	12	(0.0)	7	(0.0)	0.78
L02_ICD10	461	(0.6)	280	(0.6)	181	(0.6)	0.75
L03_ICD10	5191	(6.5)	3080	(6.4)	2111	(6.6)	0.31
L04_ICD10	133	(0.2)	80	(0.2)	53	(0.2)	0.97
L05_ICD10	2	(0.0)	2	(0.0)	0	(0.0)	0.25
L08_ICD10	346	(0.4)	201	(0.4)	145	(0.5)	0.47
L10_ICD10	38	(0.0)	21	(0.0)	17	(0.1)	0.55
L11_ICD10	41	(0.1)	23	(0.0)	18	(0.1)	0.61
L12_ICD10	157	(0.2)	98	(0.2)	59	(0.2)	0.54
L13_ICD10	163	(0.2)	101	(0.2)	62	(0.2)	0.61
L20_ICD10	188	(0.2)	103	(0.2)	85	(0.3)	0.14
L21_ICD10	1034	(1.3)	612	(1.3)	422	(1.3)	0.59
L22_ICD10	75	(0.1)	42	(0.1)	33	(0.1)	0.48
L24_ICD10	9	(0.0)	3	(0.0)	6	(0.0)	0.1
L25_ICD10	6173	(7.7)	3698	(7.7)	2475	(7.7)	0.88
L26_ICD10	337	(0.4)	204	(0.4)	133	(0.4)	0.84
L27_ICD10	557	(0.7)	356	(0.7)	201	(0.6)	0.06
L28_ICD10	845	(1.1)	485	(1.0)	360	(1.1)	0.12
L29_ICD10	2038	(2.5)	1197	(2.5)	841	(2.6)	0.24
L30_ICD10	368	(0.5)	206	(0.4)	162	(0.5)	0.11
L40_ICD10	288	(0.4)	169	(0.4)	119	(0.4)	0.65
L41_ICD10	13	(0.0)	3	(0.0)	10	(0.0)	0.007
L42_ICD10	50	(0.1)	27	(0.1)	23	(0.1)	0.39
L43_ICD10	104	(0.1)	64	(0.1)	40	(0.1)	0.75
L44_ICD10	23	(0.0)	12	(0.0)	11	(0.0)	0.44
L50_ICD10	1148	(1.4)	685	(1.4)	463	(1.4)	0.82
L51_ICD10	11	(0.0)	6	(0.0)	5	(0.0)	0.71

L52_ICD10	36	(0.0)	20	(0.0)	16	(0.0)	0.59
L53_ICD10	352	(0.4)	207	(0.4)	145	(0.5)	0.65
L55_ICD10	5	(0.0)	5	(0.0)	0	(0.0)	0.07
L56_ICD10	35	(0.0)	25	(0.1)	10	(0.0)	0.17
L57_ICD10	71	(0.1)	42	(0.1)	29	(0.1)	0.88
L58_ICD10	19	(0.0)	11	(0.0)	8	(0.0)	0.85
L59_ICD10	1	(0.0)	1	(0.0)	0	(0.0)	0.41
L60_ICD10	287	(0.4)	168	(0.3)	119	(0.4)	0.61
L63_ICD10	46	(0.1)	32	(0.1)	14	(0.0)	0.19
L65_ICD10	243	(0.3)	137	(0.3)	106	(0.3)	0.25
L66_ICD10	1053	(1.3)	628	(1.3)	425	(1.3)	0.81
L67_ICD10	5	(0.0)	3	(0.0)	2	(0.0)	1
L68_ICD10	2	(0.0)	0	(0.0)	2	(0.0)	0.08
L70_ICD10	527	(0.7)	319	(0.7)	208	(0.6)	0.8
L71_ICD10	116	(0.1)	70	(0.1)	46	(0.1)	0.94
L72_ICD10	828	(1.0)	491	(1.0)	337	(1.1)	0.68
L73_ICD10	190	(0.2)	116	(0.2)	74	(0.2)	0.77
L74_ICD10	80	(0.1)	52	(0.1)	28	(0.1)	0.36
L75_ICD10	2	(0.0)	2	(0.0)	0	(0.0)	0.25
L76_ICD10	6	(0.0)	3	(0.0)	3	(0.0)	0.62
L80_ICD10	93	(0.1)	57	(0.1)	36	(0.1)	0.8
L81_ICD10	380	(0.5)	219	(0.5)	161	(0.5)	0.34
L82_ICD10	682	(0.9)	418	(0.9)	264	(0.8)	0.49
L83_ICD10	10	(0.0)	6	(0.0)	4	(0.0)	1
L84_ICD10	562	(0.7)	327	(0.7)	235	(0.7)	0.38
L85_ICD10	1631	(2.0)	986	(2.0)	645	(2.0)	0.71
L87_ICD10	1	(0.0)	1	(0.0)	0	(0.0)	0.41
L88_ICD10	14	(0.0)	8	(0.0)	6	(0.0)	0.83
L89_ICD10	141	(0.2)	94	(0.2)	47	(0.1)	0.11
L90_ICD10	87	(0.1)	52	(0.1)	35	(0.1)	0.97
L91_ICD10	211	(0.3)	134	(0.3)	77	(0.2)	0.3
L92_ICD10	49	(0.1)	26	(0.1)	23	(0.1)	0.32
L93_ICD10	58	(0.1)	33	(0.1)	25	(0.1)	0.63
L94_ICD10	3	(0.0)	1	(0.0)	2	(0.0)	0.35
L95_ICD10	7	(0.0)	3	(0.0)	4	(0.0)	0.35
L97_ICD10	216	(0.3)	117	(0.2)	99	(0.3)	0.08
L98_ICD10	811	(1.0)	486	(1.0)	325	(1.0)	0.97
M00_ICD10	136	(0.2)	80	(0.2)	56	(0.2)	0.78
M01_ICD10	8	(0.0)	6	(0.0)	2	(0.0)	0.39
M02_ICD10	5	(0.0)	3	(0.0)	2	(0.0)	1
M06_ICD10	626	(0.8)	389	(0.8)	237	(0.7)	0.27
M08_ICD10	19	(0.0)	12	(0.0)	7	(0.0)	0.78
M10_ICD10	2558	(3.2)	1533	(3.2)	1025	(3.2)	0.94
M11_ICD10	4	(0.0)	2	(0.0)	2	(0.0)	0.68
M12_ICD10	3022	(3.8)	1838	(3.8)	1184	(3.7)	0.35
M13_ICD10	190	(0.2)	113	(0.2)	77	(0.2)	0.88
M14_ICD10	1	(0.0)	0	(0.0)	1	(0.0)	0.22
M15_ICD10	6089	(7.6)	3668	(7.6)	2421	(7.5)	0.69

M16_ICD10	820	(1.0)	502	(1.0)	318	(1.0)	0.47
M17_ICD10	3505	(4.4)	2152	(4.5)	1353	(4.2)	0.08
M18_ICD10	199	(0.2)	116	(0.2)	83	(0.3)	0.62
M19_ICD10	1396	(1.7)	867	(1.8)	529	(1.6)	0.11
M1A_ICD10	4	(0.0)	1	(0.0)	3	(0.0)	0.15
M20_ICD10	128	(0.2)	72	(0.1)	56	(0.2)	0.39
M21_ICD10	115	(0.1)	71	(0.1)	44	(0.1)	0.7
M22_ICD10	30	(0.0)	16	(0.0)	14	(0.0)	0.46
M23_ICD10	108	(0.1)	67	(0.1)	41	(0.1)	0.67
M24_ICD10	218	(0.3)	120	(0.2)	98	(0.3)	0.13
M25_ICD10	2029	(2.5)	1218	(2.5)	811	(2.5)	0.98
M26_ICD10	621	(0.8)	388	(0.8)	233	(0.7)	0.21
M27_ICD10	200	(0.2)	116	(0.2)	84	(0.3)	0.56
M30_ICD10	11	(0.0)	9	(0.0)	2	(0.0)	0.14
M31_ICD10	60	(0.1)	32	(0.1)	28	(0.1)	0.29
M32_ICD10	597	(0.7)	368	(0.8)	229	(0.7)	0.41
M33_ICD10	98	(0.1)	65	(0.1)	33	(0.1)	0.2
M34_ICD10	58	(0.1)	39	(0.1)	19	(0.1)	0.26
M35_ICD10	925	(1.2)	568	(1.2)	357	(1.1)	0.38
M36_ICD10	1	(0.0)	1	(0.0)	0	(0.0)	0.41
M40_ICD10	92	(0.1)	53	(0.1)	39	(0.1)	0.64
M41_ICD10	482	(0.6)	291	(0.6)	191	(0.6)	0.87
M43_ICD10	704	(0.9)	426	(0.9)	278	(0.9)	0.78
M45_ICD10	169	(0.2)	116	(0.2)	53	(0.2)	0.02
M46_ICD10	172	(0.2)	102	(0.2)	70	(0.2)	0.85
M47_ICD10	4896	(6.1)	2979	(6.2)	1917	(6.0)	0.21
M48_ICD10	1126	(1.4)	686	(1.4)	440	(1.4)	0.52
M49_ICD10	8	(0.0)	5	(0.0)	3	(0.0)	0.89
M50_ICD10	68	(0.1)	41	(0.1)	27	(0.1)	0.96
M51_ICD10	866	(1.1)	518	(1.1)	348	(1.1)	0.91
M53_ICD10	123	(0.2)	82	(0.2)	41	(0.1)	0.13
M54_ICD10	5133	(6.4)	3100	(6.4)	2033	(6.3)	0.55
M60_ICD10	1966	(2.5)	1158	(2.4)	808	(2.5)	0.31
M61_ICD10	9	(0.0)	4	(0.0)	5	(0.0)	0.34
M62_ICD10	281	(0.4)	162	(0.3)	119	(0.4)	0.42
M65_ICD10	1398	(1.7)	860	(1.8)	538	(1.7)	0.24
M66_ICD10	13	(0.0)	9	(0.0)	4	(0.0)	0.5
M67_ICD10	236	(0.3)	147	(0.3)	89	(0.3)	0.47
M70_ICD10	33	(0.0)	19	(0.0)	14	(0.0)	0.78
M71_ICD10	180	(0.2)	117	(0.2)	63	(0.2)	0.17
M72_ICD10	403	(0.5)	249	(0.5)	154	(0.5)	0.46
M75_ICD10	1318	(1.6)	799	(1.7)	519	(1.6)	0.64
M76_ICD10	105	(0.1)	68	(0.1)	37	(0.1)	0.32
M77_ICD10	670	(0.8)	417	(0.9)	253	(0.8)	0.23
M79_ICD10	1260	(1.6)	765	(1.6)	495	(1.5)	0.6
M81_ICD10	2249	(2.8)	1369	(2.8)	880	(2.7)	0.39
M83_ICD10	6	(0.0)	4	(0.0)	2	(0.0)	0.74
M84_ICD10	1744	(2.2)	1038	(2.2)	706	(2.2)	0.68

M85_ICD10	9	(0.0)	6	(0.0)	3	(0.0)	0.68
M86_ICD10	336	(0.4)	197	(0.4)	139	(0.4)	0.61
M87_ICD10	163	(0.2)	90	(0.2)	73	(0.2)	0.21
M88_ICD10	1	(0.0)	1	(0.0)	0	(0.0)	0.41
M89_ICD10	594	(0.7)	355	(0.7)	239	(0.7)	0.91
M92_ICD10	8	(0.0)	5	(0.0)	3	(0.0)	0.89
M93_ICD10	2	(0.0)	1	(0.0)	1	(0.0)	0.77
M94_ICD10	6	(0.0)	2	(0.0)	4	(0.0)	0.18
M95_ICD10	7	(0.0)	6	(0.0)	1	(0.0)	0.16
M96_ICD10	45	(0.1)	25	(0.1)	20	(0.1)	0.54
N00_ICD10	9	(0.0)	6	(0.0)	3	(0.0)	0.68
N01_ICD10	2	(0.0)	2	(0.0)	0	(0.0)	0.25
N02_ICD10	3	(0.0)	2	(0.0)	1	(0.0)	0.81
N03_ICD10	423	(0.5)	252	(0.5)	171	(0.5)	0.86
N04_ICD10	222	(0.3)	135	(0.3)	87	(0.3)	0.8
N05_ICD10	305	(0.4)	195	(0.4)	110	(0.3)	0.16
N08_ICD10	39	(0.0)	22	(0.0)	17	(0.1)	0.65
N10_ICD10	766	(1.0)	464	(1.0)	302	(0.9)	0.74
N11_ICD10	6	(0.0)	4	(0.0)	2	(0.0)	0.74
N12_ICD10	143	(0.2)	94	(0.2)	49	(0.2)	0.16
N13_ICD10	1262	(1.6)	763	(1.6)	499	(1.6)	0.74
N15_ICD10	85	(0.1)	54	(0.1)	31	(0.1)	0.51
N16_ICD10	3	(0.0)	2	(0.0)	1	(0.0)	0.81
N17_ICD10	637	(0.8)	392	(0.8)	245	(0.8)	0.43
N18_ICD10	3673	(4.6)	2153	(4.5)	1520	(4.7)	0.08
N19_ICD10	672	(0.8)	400	(0.8)	272	(0.8)	0.8
N20_ICD10	2167	(2.7)	1340	(2.8)	827	(2.6)	0.08
N21_ICD10	170	(0.2)	104	(0.2)	66	(0.2)	0.75
N23_ICD10	24	(0.0)	14	(0.0)	10	(0.0)	0.87
N25_ICD10	48	(0.1)	20	(0.0)	28	(0.1)	0.01
N26_ICD10	30	(0.0)	24	(0.0)	6	(0.0)	0.03
N27_ICD10	8	(0.0)	4	(0.0)	4	(0.0)	0.56
N28_ICD10	3087	(3.8)	1826	(3.8)	1261	(3.9)	0.33
N30_ICD10	486	(0.6)	317	(0.7)	169	(0.5)	0.02
N31_ICD10	1643	(2.0)	952	(2.0)	691	(2.2)	0.09
N32_ICD10	795	(1.0)	487	(1.0)	308	(1.0)	0.47
N34_ICD10	56	(0.1)	29	(0.1)	27	(0.1)	0.21
N35_ICD10	107	(0.1)	71	(0.1)	36	(0.1)	0.18
N36_ICD10	50	(0.1)	33	(0.1)	17	(0.1)	0.39
N37_ICD10	1	(0.0)	1	(0.0)	0	(0.0)	0.41
N39_ICD10	8905	(11.1)	5386	(11.2)	3519	(11.0)	0.32
N40_ICD10	1473	(1.8)	913	(1.9)	560	(1.7)	0.12
N41_ICD10	361	(0.5)	214	(0.4)	147	(0.5)	0.78
N42_ICD10	7	(0.0)	3	(0.0)	4	(0.0)	0.35
N43_ICD10	85	(0.1)	57	(0.1)	28	(0.1)	0.18
N44_ICD10	21	(0.0)	13	(0.0)	8	(0.0)	0.86
N45_ICD10	205	(0.3)	129	(0.3)	76	(0.2)	0.39
N46_ICD10	90	(0.1)	56	(0.1)	34	(0.1)	0.67

N47_ICD10	185	(0.2)	101	(0.2)	84	(0.3)	0.13
N48_ICD10	33	(0.0)	25	(0.1)	8	(0.0)	0.06
N49_ICD10	19	(0.0)	11	(0.0)	8	(0.0)	0.85
N50_ICD10	97	(0.1)	64	(0.1)	33	(0.1)	0.23
N52_ICD10	278	(0.3)	173	(0.4)	105	(0.3)	0.45
N60_ICD10	168	(0.2)	100	(0.2)	68	(0.2)	0.9
N61_ICD10	98	(0.1)	65	(0.1)	33	(0.1)	0.2
N62_ICD10	89	(0.1)	51	(0.1)	38	(0.1)	0.6
N63_ICD10	1246	(1.6)	760	(1.6)	486	(1.5)	0.47
N64_ICD10	147	(0.2)	83	(0.2)	64	(0.2)	0.38
N70_ICD10	32	(0.0)	22	(0.0)	10	(0.0)	0.31
N71_ICD10	25	(0.0)	13	(0.0)	12	(0.0)	0.41
N72_ICD10	16	(0.0)	11	(0.0)	5	(0.0)	0.47
N73_ICD10	282	(0.4)	178	(0.4)	104	(0.3)	0.28
N75_ICD10	48	(0.1)	31	(0.1)	17	(0.1)	0.52
N76_ICD10	1508	(1.9)	936	(1.9)	572	(1.8)	0.1
N77_ICD10	9	(0.0)	4	(0.0)	5	(0.0)	0.34
N80_ICD10	463	(0.6)	294	(0.6)	169	(0.5)	0.12
N81_ICD10	150	(0.2)	86	(0.2)	64	(0.2)	0.5
N82_ICD10	13	(0.0)	8	(0.0)	5	(0.0)	0.91
N83_ICD10	359	(0.4)	219	(0.5)	140	(0.4)	0.7
N84_ICD10	263	(0.3)	167	(0.3)	96	(0.3)	0.25
N85_ICD10	318	(0.4)	188	(0.4)	130	(0.4)	0.75
N86_ICD10	61	(0.1)	33	(0.1)	28	(0.1)	0.35
N87_ICD10	13	(0.0)	11	(0.0)	2	(0.0)	0.07
N88_ICD10	2	(0.0)	2	(0.0)	0	(0.0)	0.25
N89_ICD10	49	(0.1)	25	(0.1)	24	(0.1)	0.2
N90_ICD10	25	(0.0)	17	(0.0)	8	(0.0)	0.41
N91_ICD10	153	(0.2)	102	(0.2)	51	(0.2)	0.09
N92_ICD10	813	(1.0)	487	(1.0)	326	(1.0)	0.95
N93_ICD10	70	(0.1)	41	(0.1)	29	(0.1)	0.81
N94_ICD10	161	(0.2)	99	(0.2)	62	(0.2)	0.7
N95_ICD10	2008	(2.5)	1209	(2.5)	799	(2.5)	0.85
N97_ICD10	358	(0.4)	210	(0.4)	148	(0.5)	0.6
N99_ICD10	5	(0.0)	3	(0.0)	2	(0.0)	1
NoD_ICD10	8	(0.0)	5	(0.0)	3	(0.0)	0.89
O00_ICD10	1	(0.0)	0	(0.0)	1	(0.0)	0.22
O01_ICD10	1	(0.0)	1	(0.0)	0	(0.0)	0.41
O02_ICD10	14	(0.0)	9	(0.0)	5	(0.0)	0.74
O03_ICD10	18	(0.0)	10	(0.0)	8	(0.0)	0.7
O04_ICD10	1	(0.0)	1	(0.0)	0	(0.0)	0.41
O08_ICD10	1	(0.0)	0	(0.0)	1	(0.0)	0.22
O10_ICD10	4	(0.0)	4	(0.0)	0	(0.0)	0.1
O13_ICD10	19	(0.0)	9	(0.0)	10	(0.0)	0.26
O14_ICD10	26	(0.0)	18	(0.0)	8	(0.0)	0.34
O20_ICD10	249	(0.3)	145	(0.3)	104	(0.3)	0.57
O21_ICD10	5	(0.0)	4	(0.0)	1	(0.0)	0.36
O22_ICD10	1	(0.0)	1	(0.0)	0	(0.0)	0.41

O23_ICD10	2	(0.0)	1	(0.0)	1	(0.0)	0.77
O24_ICD10	15	(0.0)	11	(0.0)	4	(0.0)	0.29
O26_ICD10	12	(0.0)	9	(0.0)	3	(0.0)	0.29
O30_ICD10	12	(0.0)	9	(0.0)	3	(0.0)	0.29
O31_ICD10	1	(0.0)	1	(0.0)	0	(0.0)	0.41
O32_ICD10	51	(0.1)	37	(0.1)	14	(0.0)	0.07
O33_ICD10	7	(0.0)	2	(0.0)	5	(0.0)	0.09
O34_ICD10	95	(0.1)	62	(0.1)	33	(0.1)	0.29
O35_ICD10	31	(0.0)	19	(0.0)	12	(0.0)	0.88
O36_ICD10	19	(0.0)	11	(0.0)	8	(0.0)	0.85
O41_ICD10	2	(0.0)	1	(0.0)	1	(0.0)	0.77
O42_ICD10	6	(0.0)	4	(0.0)	2	(0.0)	0.74
O43_ICD10	2	(0.0)	1	(0.0)	1	(0.0)	0.77
O44_ICD10	9	(0.0)	3	(0.0)	6	(0.0)	0.1
O45_ICD10	1	(0.0)	0	(0.0)	1	(0.0)	0.22
O46_ICD10	7	(0.0)	3	(0.0)	4	(0.0)	0.35
O47_ICD10	2	(0.0)	2	(0.0)	0	(0.0)	0.25
O48_ICD10	6	(0.0)	1	(0.0)	5	(0.0)	0.03
O60_ICD10	46	(0.1)	25	(0.1)	21	(0.1)	0.43
O61_ICD10	2	(0.0)	2	(0.0)	0	(0.0)	0.25
O62_ICD10	11	(0.0)	8	(0.0)	3	(0.0)	0.39
O63_ICD10	31	(0.0)	19	(0.0)	12	(0.0)	0.88
O66_ICD10	1	(0.0)	0	(0.0)	1	(0.0)	0.22
O68_ICD10	21	(0.0)	10	(0.0)	11	(0.0)	0.25
O70_ICD10	243	(0.3)	146	(0.3)	97	(0.3)	0.98
O72_ICD10	1	(0.0)	1	(0.0)	0	(0.0)	0.41
O75_ICD10	35	(0.0)	27	(0.1)	8	(0.0)	0.04
O80_ICD10	3	(0.0)	2	(0.0)	1	(0.0)	0.81
O82_ICD10	18	(0.0)	9	(0.0)	9	(0.0)	0.39
O86_ICD10	1	(0.0)	1	(0.0)	0	(0.0)	0.41
O87_ICD10	1	(0.0)	1	(0.0)	0	(0.0)	0.41
O91_ICD10	12	(0.0)	7	(0.0)	5	(0.0)	0.91
O92_ICD10	1	(0.0)	1	(0.0)	0	(0.0)	0.41
O98_ICD10	3	(0.0)	2	(0.0)	1	(0.0)	0.81
O99_ICD10	30	(0.0)	21	(0.0)	9	(0.0)	0.26
P00_ICD10	2	(0.0)	1	(0.0)	1	(0.0)	0.77
P02_ICD10	1	(0.0)	1	(0.0)	0	(0.0)	0.41
P07_ICD10	2	(0.0)	2	(0.0)	0	(0.0)	0.25
P10_ICD10	20	(0.0)	13	(0.0)	7	(0.0)	0.65
P11_ICD10	4	(0.0)	3	(0.0)	1	(0.0)	0.54
P13_ICD10	1	(0.0)	1	(0.0)	0	(0.0)	0.41
P23_ICD10	3	(0.0)	1	(0.0)	2	(0.0)	0.35
P25_ICD10	3	(0.0)	0	(0.0)	3	(0.0)	0.03
P26_ICD10	4	(0.0)	3	(0.0)	1	(0.0)	0.54
P28_ICD10	12	(0.0)	4	(0.0)	8	(0.0)	0.06
P35_ICD10	3	(0.0)	2	(0.0)	1	(0.0)	0.81
P38_ICD10	6	(0.0)	5	(0.0)	1	(0.0)	0.24
P54_ICD10	2	(0.0)	1	(0.0)	1	(0.0)	0.77

P55_ICD10	8	(0.0)	6	(0.0)	2	(0.0)	0.39
P58_ICD10	1	(0.0)	0	(0.0)	1	(0.0)	0.22
P59_ICD10	3	(0.0)	2	(0.0)	1	(0.0)	0.81
P70_ICD10	3	(0.0)	2	(0.0)	1	(0.0)	0.81
P71_ICD10	1	(0.0)	1	(0.0)	0	(0.0)	0.41
P78_ICD10	1	(0.0)	1	(0.0)	0	(0.0)	0.41
P80_ICD10	6	(0.0)	3	(0.0)	3	(0.0)	0.62
P91_ICD10	3	(0.0)	2	(0.0)	1	(0.0)	0.81
P95_ICD10	1	(0.0)	1	(0.0)	0	(0.0)	0.41
Q00_ICD10	2	(0.0)	0	(0.0)	2	(0.0)	0.08
Q01_ICD10	2	(0.0)	1	(0.0)	1	(0.0)	0.77
Q03_ICD10	11	(0.0)	8	(0.0)	3	(0.0)	0.39
Q04_ICD10	15	(0.0)	5	(0.0)	10	(0.0)	0.04
Q05_ICD10	5	(0.0)	4	(0.0)	1	(0.0)	0.36
Q06_ICD10	14	(0.0)	9	(0.0)	5	(0.0)	0.74
Q07_ICD10	4	(0.0)	3	(0.0)	1	(0.0)	0.54
Q10_ICD10	10	(0.0)	8	(0.0)	2	(0.0)	0.2
Q11_ICD10	2	(0.0)	1	(0.0)	1	(0.0)	0.77
Q12_ICD10	5	(0.0)	3	(0.0)	2	(0.0)	1
Q13_ICD10	1	(0.0)	1	(0.0)	0	(0.0)	0.41
Q14_ICD10	9	(0.0)	7	(0.0)	2	(0.0)	0.28
Q15_ICD10	8	(0.0)	5	(0.0)	3	(0.0)	0.89
Q16_ICD10	125	(0.2)	75	(0.2)	50	(0.2)	1
Q18_ICD10	12	(0.0)	6	(0.0)	6	(0.0)	0.48
Q20_ICD10	56	(0.1)	35	(0.1)	21	(0.1)	0.7
Q21_ICD10	114	(0.1)	66	(0.1)	48	(0.1)	0.65
Q22_ICD10	6	(0.0)	5	(0.0)	1	(0.0)	0.24
Q23_ICD10	28	(0.0)	19	(0.0)	9	(0.0)	0.4
Q24_ICD10	13	(0.0)	8	(0.0)	5	(0.0)	0.91
Q25_ICD10	41	(0.1)	27	(0.1)	14	(0.0)	0.44
Q26_ICD10	3	(0.0)	1	(0.0)	2	(0.0)	0.35
Q27_ICD10	15	(0.0)	10	(0.0)	5	(0.0)	0.6
Q28_ICD10	66	(0.1)	43	(0.1)	23	(0.1)	0.39
Q31_ICD10	2	(0.0)	2	(0.0)	0	(0.0)	0.25
Q33_ICD10	9	(0.0)	7	(0.0)	2	(0.0)	0.28
Q35_ICD10	5	(0.0)	2	(0.0)	3	(0.0)	0.36
Q38_ICD10	3	(0.0)	1	(0.0)	2	(0.0)	0.35
Q39_ICD10	8	(0.0)	6	(0.0)	2	(0.0)	0.39
Q40_ICD10	10	(0.0)	4	(0.0)	6	(0.0)	0.2
Q41_ICD10	9	(0.0)	3	(0.0)	6	(0.0)	0.1
Q42_ICD10	16	(0.0)	9	(0.0)	7	(0.0)	0.76
Q43_ICD10	32	(0.0)	21	(0.0)	11	(0.0)	0.52
Q44_ICD10	122	(0.2)	75	(0.2)	47	(0.1)	0.74
Q45_ICD10	37	(0.0)	26	(0.1)	11	(0.0)	0.2
Q51_ICD10	12	(0.0)	9	(0.0)	3	(0.0)	0.29
Q53_ICD10	4	(0.0)	0	(0.0)	4	(0.0)	0.01
Q54_ICD10	9	(0.0)	6	(0.0)	3	(0.0)	0.68
Q55_ICD10	8	(0.0)	3	(0.0)	5	(0.0)	0.19

Q60_ICD10	12	(0.0)	7	(0.0)	5	(0.0)	0.91
Q61_ICD10	199	(0.2)	116	(0.2)	83	(0.3)	0.62
Q62_ICD10	39	(0.0)	25	(0.1)	14	(0.0)	0.6
Q63_ICD10	16	(0.0)	8	(0.0)	8	(0.0)	0.41
Q64_ICD10	8	(0.0)	2	(0.0)	6	(0.0)	0.04
Q65_ICD10	3	(0.0)	1	(0.0)	2	(0.0)	0.35
Q66_ICD10	72	(0.1)	42	(0.1)	30	(0.1)	0.77
Q67_ICD10	15	(0.0)	10	(0.0)	5	(0.0)	0.6
Q68_ICD10	2	(0.0)	2	(0.0)	0	(0.0)	0.25
Q74_ICD10	26	(0.0)	18	(0.0)	8	(0.0)	0.34
Q75_ICD10	3	(0.0)	1	(0.0)	2	(0.0)	0.35
Q76_ICD10	107	(0.1)	66	(0.1)	41	(0.1)	0.72
Q78_ICD10	21	(0.0)	13	(0.0)	8	(0.0)	0.86
Q79_ICD10	12	(0.0)	7	(0.0)	5	(0.0)	0.91
Q80_ICD10	27	(0.0)	15	(0.0)	12	(0.0)	0.64
Q81_ICD10	34	(0.0)	17	(0.0)	17	(0.1)	0.23
Q82_ICD10	3	(0.0)	1	(0.0)	2	(0.0)	0.35
Q85_ICD10	57	(0.1)	37	(0.1)	20	(0.1)	0.45
Q87_ICD10	13	(0.0)	10	(0.0)	3	(0.0)	0.21
Q89_ICD10	7	(0.0)	5	(0.0)	2	(0.0)	0.54
Q90_ICD10	5	(0.0)	3	(0.0)	2	(0.0)	1
Q92_ICD10	7	(0.0)	5	(0.0)	2	(0.0)	0.54
Q96_ICD10	2	(0.0)	2	(0.0)	0	(0.0)	0.25
Q97_ICD10	1	(0.0)	1	(0.0)	0	(0.0)	0.41
Q98_ICD10	1	(0.0)	1	(0.0)	0	(0.0)	0.41
Q99_ICD10	14	(0.0)	6	(0.0)	8	(0.0)	0.19
R00_ICD10	1106	(1.4)	642	(1.3)	464	(1.4)	0.18
R01_ICD10	148	(0.2)	86	(0.2)	62	(0.2)	0.64
R03_ICD10	118	(0.1)	77	(0.2)	41	(0.1)	0.24
R04_ICD10	760	(0.9)	443	(0.9)	317	(1.0)	0.33
R05_ICD10	5142	(6.4)	3114	(6.5)	2028	(6.3)	0.4
R06_ICD10	3047	(3.8)	1805	(3.8)	1242	(3.9)	0.38
R07_ICD10	3824	(4.8)	2301	(4.8)	1523	(4.7)	0.82
R09_ICD10	38	(0.0)	24	(0.0)	14	(0.0)	0.69
R10_ICD10	6940	(8.7)	4186	(8.7)	2754	(8.6)	0.57
R11_ICD10	958	(1.2)	592	(1.2)	366	(1.1)	0.25
R12_ICD10	5	(0.0)	5	(0.0)	0	(0.0)	0.07
R13_ICD10	234	(0.3)	136	(0.3)	98	(0.3)	0.56
R14_ICD10	189	(0.2)	116	(0.2)	73	(0.2)	0.7
R15_ICD10	6	(0.0)	2	(0.0)	4	(0.0)	0.18
R16_ICD10	291	(0.4)	178	(0.4)	113	(0.4)	0.68
R17_ICD10	613	(0.8)	358	(0.7)	255	(0.8)	0.42
R18_ICD10	77	(0.1)	44	(0.1)	33	(0.1)	0.61
R19_ICD10	961	(1.2)	576	(1.2)	385	(1.2)	0.97
R20_ICD10	512	(0.6)	319	(0.7)	193	(0.6)	0.29
R21_ICD10	196	(0.2)	117	(0.2)	79	(0.2)	0.93
R22_ICD10	1396	(1.7)	830	(1.7)	566	(1.8)	0.68
R23_ICD10	70	(0.1)	41	(0.1)	29	(0.1)	0.81

R25_ICD10	579	(0.7)	335	(0.7)	244	(0.8)	0.29
R26_ICD10	89	(0.1)	60	(0.1)	29	(0.1)	0.15
R27_ICD10	5	(0.0)	3	(0.0)	2	(0.0)	1
R29_ICD10	133	(0.2)	94	(0.2)	39	(0.1)	0.01
R30_ICD10	341	(0.4)	207	(0.4)	134	(0.4)	0.79
R31_ICD10	391	(0.5)	260	(0.5)	131	(0.4)	0.009
R32_ICD10	424	(0.5)	252	(0.5)	172	(0.5)	0.81
R33_ICD10	949	(1.2)	566	(1.2)	383	(1.2)	0.82
R34_ICD10	30	(0.0)	21	(0.0)	9	(0.0)	0.26
R35_ICD10	1108	(1.4)	674	(1.4)	434	(1.4)	0.57
R36_ICD10	5	(0.0)	5	(0.0)	0	(0.0)	0.07
R39_ICD10	34	(0.0)	27	(0.1)	7	(0.0)	0.02
R40_ICD10	108	(0.1)	55	(0.1)	53	(0.2)	0.05
R41_ICD10	41	(0.1)	28	(0.1)	13	(0.0)	0.28
R42_ICD10	3789	(4.7)	2264	(4.7)	1525	(4.8)	0.75
R43_ICD10	50	(0.1)	31	(0.1)	19	(0.1)	0.77
R44_ICD10	15	(0.0)	10	(0.0)	5	(0.0)	0.6
R45_ICD10	5	(0.0)	3	(0.0)	2	(0.0)	1
R47_ICD10	142	(0.2)	86	(0.2)	56	(0.2)	0.89
R48_ICD10	5	(0.0)	3	(0.0)	2	(0.0)	1
R49_ICD10	323	(0.4)	194	(0.4)	129	(0.4)	0.98
R50_ICD10	2271	(2.8)	1369	(2.8)	902	(2.8)	0.78
R51_ICD10	279	(0.3)	179	(0.4)	100	(0.3)	0.16
R52_ICD10	10	(0.0)	7	(0.0)	3	(0.0)	0.52
R53_ICD10	228	(0.3)	127	(0.3)	101	(0.3)	0.18
R55_ICD10	801	(1.0)	453	(0.9)	348	(1.1)	0.05
R56_ICD10	782	(1.0)	474	(1.0)	308	(1.0)	0.72
R57_ICD10	707	(0.9)	402	(0.8)	305	(1.0)	0.09
R58_ICD10	8	(0.0)	4	(0.0)	4	(0.0)	0.56
R59_ICD10	347	(0.4)	202	(0.4)	145	(0.5)	0.5
R60_ICD10	2416	(3.0)	1466	(3.0)	950	(3.0)	0.49
R61_ICD10	19	(0.0)	12	(0.0)	7	(0.0)	0.78
R62_ICD10	23	(0.0)	13	(0.0)	10	(0.0)	0.73
R63_ICD10	1280	(1.6)	753	(1.6)	527	(1.6)	0.39
R64_ICD10	394	(0.5)	226	(0.5)	168	(0.5)	0.28
R65_ICD10	67	(0.1)	39	(0.1)	28	(0.1)	0.76
R68_ICD10	434	(0.5)	260	(0.5)	174	(0.5)	0.97
R69_ICD10	1	(0.0)	1	(0.0)	0	(0.0)	0.41
R73_ICD10	107	(0.1)	68	(0.1)	39	(0.1)	0.45
R74_ICD10	5	(0.0)	4	(0.0)	1	(0.0)	0.36
R75_ICD10	1	(0.0)	1	(0.0)	0	(0.0)	0.41
R76_ICD10	119	(0.1)	70	(0.1)	49	(0.2)	0.79
R78_ICD10	2809	(3.5)	1641	(3.4)	1168	(3.6)	0.08
R79_ICD10	13	(0.0)	8	(0.0)	5	(0.0)	0.91
R80_ICD10	694	(0.9)	420	(0.9)	274	(0.9)	0.78
R81_ICD10	3	(0.0)	3	(0.0)	0	(0.0)	0.16
R82_ICD10	94	(0.1)	61	(0.1)	33	(0.1)	0.33
R83_ICD10	1	(0.0)	1	(0.0)	0	(0.0)	0.41

R85_ICD10	5	(0.0)	3	(0.0)	2	(0.0)	1
R87_ICD10	8	(0.0)	5	(0.0)	3	(0.0)	0.89
R88_ICD10	1	(0.0)	0	(0.0)	1	(0.0)	0.22
R91_ICD10	10	(0.0)	3	(0.0)	7	(0.0)	0.05
R93_ICD10	160	(0.2)	113	(0.2)	47	(0.1)	0.006
R94_ICD10	1489	(1.9)	939	(2.0)	550	(1.7)	0.01
R97_ICD10	767	(1.0)	468	(1.0)	299	(0.9)	0.56
R99_ICD10	6	(0.0)	4	(0.0)	2	(0.0)	0.74
S00_ICD10	327	(0.4)	203	(0.4)	124	(0.4)	0.44
S01_ICD10	265	(0.3)	177	(0.4)	88	(0.3)	0.02
S02_ICD10	658	(0.8)	410	(0.9)	248	(0.8)	0.22
S03_ICD10	11	(0.0)	6	(0.0)	5	(0.0)	0.71
S04_ICD10	9	(0.0)	7	(0.0)	2	(0.0)	0.28
S05_ICD10	61	(0.1)	42	(0.1)	19	(0.1)	0.16
S06_ICD10	496	(0.6)	293	(0.6)	203	(0.6)	0.67
S07_ICD10	2	(0.0)	1	(0.0)	1	(0.0)	0.77
S09_ICD10	1544	(1.9)	968	(2.0)	576	(1.8)	0.03
S11_ICD10	4	(0.0)	2	(0.0)	2	(0.0)	0.68
S12_ICD10	429	(0.5)	255	(0.5)	174	(0.5)	0.81
S13_ICD10	215	(0.3)	139	(0.3)	76	(0.2)	0.16
S14_ICD10	153	(0.2)	91	(0.2)	62	(0.2)	0.9
S15_ICD10	2	(0.0)	2	(0.0)	0	(0.0)	0.25
S19_ICD10	2	(0.0)	1	(0.0)	1	(0.0)	0.77
S20_ICD10	356	(0.4)	209	(0.4)	147	(0.5)	0.62
S21_ICD10	62	(0.1)	41	(0.1)	21	(0.1)	0.32
S22_ICD10	295	(0.4)	170	(0.4)	125	(0.4)	0.4
S23_ICD10	534	(0.7)	310	(0.6)	224	(0.7)	0.36
S24_ICD10	11	(0.0)	9	(0.0)	2	(0.0)	0.14
S25_ICD10	1	(0.0)	1	(0.0)	0	(0.0)	0.41
S26_ICD10	1	(0.0)	1	(0.0)	0	(0.0)	0.41
S27_ICD10	16	(0.0)	10	(0.0)	6	(0.0)	0.84
S30_ICD10	198	(0.2)	124	(0.3)	74	(0.2)	0.45
S31_ICD10	1307	(1.6)	774	(1.6)	533	(1.7)	0.56
S32_ICD10	373	(0.5)	216	(0.4)	157	(0.5)	0.41
S33_ICD10	86	(0.1)	53	(0.1)	33	(0.1)	0.76
S34_ICD10	25	(0.0)	20	(0.0)	5	(0.0)	0.04
S35_ICD10	12	(0.0)	5	(0.0)	7	(0.0)	0.19
S36_ICD10	14	(0.0)	10	(0.0)	4	(0.0)	0.38
S37_ICD10	23	(0.0)	9	(0.0)	14	(0.0)	0.04
S40_ICD10	167	(0.2)	99	(0.2)	68	(0.2)	0.85
S41_ICD10	69	(0.1)	34	(0.1)	35	(0.1)	0.07
S42_ICD10	500	(0.6)	305	(0.6)	195	(0.6)	0.65
S43_ICD10	580	(0.7)	371	(0.8)	209	(0.7)	0.05
S44_ICD10	6	(0.0)	3	(0.0)	3	(0.0)	0.62
S46_ICD10	2	(0.0)	2	(0.0)	0	(0.0)	0.25
S47_ICD10	1	(0.0)	1	(0.0)	0	(0.0)	0.41
S48_ICD10	3	(0.0)	2	(0.0)	1	(0.0)	0.81
S49_ICD10	60	(0.1)	31	(0.1)	29	(0.1)	0.19

S50_ICD10	101	(0.1)	62	(0.1)	39	(0.1)	0.78
S51_ICD10	41	(0.1)	22	(0.0)	19	(0.1)	0.41
S52_ICD10	480	(0.6)	277	(0.6)	203	(0.6)	0.3
S53_ICD10	38	(0.0)	22	(0.0)	16	(0.0)	0.79
S57_ICD10	1	(0.0)	1	(0.0)	0	(0.0)	0.41
S58_ICD10	1	(0.0)	1	(0.0)	0	(0.0)	0.41
S59_ICD10	19	(0.0)	7	(0.0)	12	(0.0)	0.04
S60_ICD10	177	(0.2)	111	(0.2)	66	(0.2)	0.46
S61_ICD10	272	(0.3)	169	(0.4)	103	(0.3)	0.47
S62_ICD10	160	(0.2)	102	(0.2)	58	(0.2)	0.33
S63_ICD10	169	(0.2)	95	(0.2)	74	(0.2)	0.31
S64_ICD10	1	(0.0)	1	(0.0)	0	(0.0)	0.41
S66_ICD10	1	(0.0)	0	(0.0)	1	(0.0)	0.22
S67_ICD10	7	(0.0)	6	(0.0)	1	(0.0)	0.16
S68_ICD10	9	(0.0)	6	(0.0)	3	(0.0)	0.68
S69_ICD10	177	(0.2)	107	(0.2)	70	(0.2)	0.9
S70_ICD10	379	(0.5)	223	(0.5)	156	(0.5)	0.64
S71_ICD10	9	(0.0)	5	(0.0)	4	(0.0)	0.79
S72_ICD10	1273	(1.6)	764	(1.6)	509	(1.6)	0.99
S73_ICD10	240	(0.3)	148	(0.3)	92	(0.3)	0.6
S75_ICD10	2	(0.0)	2	(0.0)	0	(0.0)	0.25
S76_ICD10	1	(0.0)	0	(0.0)	1	(0.0)	0.22
S78_ICD10	39	(0.0)	22	(0.0)	17	(0.1)	0.65
S79_ICD10	107	(0.1)	66	(0.1)	41	(0.1)	0.72
S80_ICD10	286	(0.4)	164	(0.3)	122	(0.4)	0.36
S81_ICD10	521	(0.6)	321	(0.7)	200	(0.6)	0.45
S82_ICD10	414	(0.5)	242	(0.5)	172	(0.5)	0.52
S83_ICD10	437	(0.5)	278	(0.6)	159	(0.5)	0.12
S86_ICD10	10	(0.0)	8	(0.0)	2	(0.0)	0.2
S87_ICD10	3	(0.0)	0	(0.0)	3	(0.0)	0.03
S88_ICD10	22	(0.0)	15	(0.0)	7	(0.0)	0.43
S89_ICD10	151	(0.2)	90	(0.2)	61	(0.2)	0.92
S90_ICD10	157	(0.2)	83	(0.2)	74	(0.2)	0.07
S91_ICD10	337	(0.4)	189	(0.4)	148	(0.5)	0.14
S92_ICD10	170	(0.2)	98	(0.2)	72	(0.2)	0.53
S93_ICD10	386	(0.5)	220	(0.5)	166	(0.5)	0.23
S97_ICD10	14	(0.0)	9	(0.0)	5	(0.0)	0.74
S98_ICD10	20	(0.0)	13	(0.0)	7	(0.0)	0.65
T07_ICD10	431	(0.5)	274	(0.6)	157	(0.5)	0.13
T14_ICD10	260	(0.3)	149	(0.3)	111	(0.3)	0.37
T15_ICD10	80	(0.1)	44	(0.1)	36	(0.1)	0.36
T16_ICD10	11	(0.0)	5	(0.0)	6	(0.0)	0.32
T17_ICD10	66	(0.1)	44	(0.1)	22	(0.1)	0.27
T18_ICD10	60	(0.1)	46	(0.1)	14	(0.0)	0.008
T19_ICD10	1	(0.0)	1	(0.0)	0	(0.0)	0.41
T20_ICD10	25	(0.0)	16	(0.0)	9	(0.0)	0.68
T21_ICD10	32	(0.0)	16	(0.0)	16	(0.0)	0.25
T22_ICD10	24	(0.0)	8	(0.0)	16	(0.0)	0.008

T23_ICD10	22	(0.0)	12	(0.0)	10	(0.0)	0.6
T24_ICD10	48	(0.1)	28	(0.1)	20	(0.1)	0.81
T25_ICD10	7	(0.0)	4	(0.0)	3	(0.0)	0.88
T26_ICD10	6	(0.0)	3	(0.0)	3	(0.0)	0.62
T28_ICD10	2	(0.0)	2	(0.0)	0	(0.0)	0.25
T30_ICD10	199	(0.2)	116	(0.2)	83	(0.3)	0.62
T31_ICD10	28	(0.0)	13	(0.0)	15	(0.0)	0.14
T33_ICD10	1	(0.0)	1	(0.0)	0	(0.0)	0.41
T42_ICD10	8	(0.0)	5	(0.0)	3	(0.0)	0.89
T43_ICD10	2	(0.0)	1	(0.0)	1	(0.0)	0.77
T45_ICD10	2	(0.0)	1	(0.0)	1	(0.0)	0.77
T46_ICD10	3	(0.0)	3	(0.0)	0	(0.0)	0.16
T48_ICD10	5	(0.0)	3	(0.0)	2	(0.0)	1
T50_ICD10	26	(0.0)	15	(0.0)	11	(0.0)	0.81
T56_ICD10	9	(0.0)	6	(0.0)	3	(0.0)	0.68
T57_ICD10	3	(0.0)	1	(0.0)	2	(0.0)	0.35
T58_ICD10	3	(0.0)	2	(0.0)	1	(0.0)	0.81
T60_ICD10	1	(0.0)	1	(0.0)	0	(0.0)	0.41
T63_ICD10	1	(0.0)	0	(0.0)	1	(0.0)	0.22
T65_ICD10	4	(0.0)	3	(0.0)	1	(0.0)	0.54
T66_ICD10	1	(0.0)	0	(0.0)	1	(0.0)	0.22
T67_ICD10	1	(0.0)	0	(0.0)	1	(0.0)	0.22
T68_ICD10	11	(0.0)	6	(0.0)	5	(0.0)	0.71
T69_ICD10	7	(0.0)	4	(0.0)	3	(0.0)	0.88
T70_ICD10	3	(0.0)	2	(0.0)	1	(0.0)	0.81
T75_ICD10	3	(0.0)	3	(0.0)	0	(0.0)	0.16
T78_ICD10	307	(0.4)	198	(0.4)	109	(0.3)	0.11
T79_ICD10	71	(0.1)	48	(0.1)	23	(0.1)	0.19
T80_ICD10	7	(0.0)	5	(0.0)	2	(0.0)	0.54
T81_ICD10	94	(0.1)	61	(0.1)	33	(0.1)	0.33
T82_ICD10	127	(0.2)	86	(0.2)	41	(0.1)	0.08
T83_ICD10	13	(0.0)	8	(0.0)	5	(0.0)	0.91
T84_ICD10	40	(0.0)	25	(0.1)	15	(0.0)	0.75
T85_ICD10	153	(0.2)	99	(0.2)	54	(0.2)	0.23
T86_ICD10	192	(0.2)	115	(0.2)	77	(0.2)	0.98
T87_ICD10	1	(0.0)	1	(0.0)	0	(0.0)	0.41
V81_ICD10	2	(0.0)	1	(0.0)	1	(0.0)	0.77
V90_ICD10	4	(0.0)	3	(0.0)	1	(0.0)	0.54
V91_ICD10	7	(0.0)	5	(0.0)	2	(0.0)	0.54
V93_ICD10	63	(0.1)	39	(0.1)	24	(0.1)	0.76
W06_ICD10	1	(0.0)	0	(0.0)	1	(0.0)	0.22
W07_ICD10	1	(0.0)	1	(0.0)	0	(0.0)	0.41
W18_ICD10	3	(0.0)	3	(0.0)	0	(0.0)	0.16
W19_ICD10	14	(0.0)	9	(0.0)	5	(0.0)	0.74
W46_ICD10	1	(0.0)	1	(0.0)	0	(0.0)	0.41
W55_ICD10	2	(0.0)	2	(0.0)	0	(0.0)	0.25
W57_ICD10	1	(0.0)	0	(0.0)	1	(0.0)	0.22
X58_ICD10	11	(0.0)	5	(0.0)	6	(0.0)	0.32

Y83_ICD10	1	(0.0)	1	(0.0)	0	(0.0)	0.41
Y84_ICD10	1	(0.0)	1	(0.0)	0	(0.0)	0.41
Y93_ICD10	6	(0.0)	6	(0.0)	0	(0.0)	0.05
Z00_ICD10	10283	(12.8)	6244	(13.0)	4039	(12.6)	0.11
Z01_ICD10	770	(1.0)	473	(1.0)	297	(0.9)	0.42
Z02_ICD10	1179	(1.5)	722	(1.5)	457	(1.4)	0.38
Z03_ICD10	4	(0.0)	0	(0.0)	4	(0.0)	0.01
Z04_ICD10	29	(0.0)	17	(0.0)	12	(0.0)	0.88
Z08_ICD10	5	(0.0)	4	(0.0)	1	(0.0)	0.36
Z09_ICD10	3316	(4.1)	1993	(4.1)	1323	(4.1)	0.9
Z11_ICD10	3	(0.0)	1	(0.0)	2	(0.0)	0.35
Z12_ICD10	3775	(4.7)	2316	(4.8)	1459	(4.5)	0.08
Z13_ICD10	3	(0.0)	2	(0.0)	1	(0.0)	0.81
Z16_ICD10	6	(0.0)	3	(0.0)	3	(0.0)	0.62
Z18_ICD10	4	(0.0)	4	(0.0)	0	(0.0)	0.1
Z20_ICD10	226	(0.3)	134	(0.3)	92	(0.3)	0.83
Z21_ICD10	376	(0.5)	220	(0.5)	156	(0.5)	0.55
Z22_ICD10	1715	(2.1)	1051	(2.2)	664	(2.1)	0.27
Z23_ICD10	1773	(2.2)	1097	(2.3)	676	(2.1)	0.1
Z30_ICD10	22	(0.0)	13	(0.0)	9	(0.0)	0.93
Z31_ICD10	22	(0.0)	9	(0.0)	13	(0.0)	0.07
Z32_ICD10	41	(0.1)	26	(0.1)	15	(0.0)	0.66
Z33_ICD10	34	(0.0)	21	(0.0)	13	(0.0)	0.83
Z34_ICD10	668	(0.8)	397	(0.8)	271	(0.8)	0.76
Z36_ICD10	124	(0.2)	83	(0.2)	41	(0.1)	0.11
Z39_ICD10	420	(0.5)	252	(0.5)	168	(0.5)	1
Z41_ICD10	594	(0.7)	357	(0.7)	237	(0.7)	0.96
Z43_ICD10	66	(0.1)	33	(0.1)	33	(0.1)	0.1
Z44_ICD10	2	(0.0)	0	(0.0)	2	(0.0)	0.08
Z45_ICD10	23	(0.0)	15	(0.0)	8	(0.0)	0.61
Z46_ICD10	425	(0.5)	268	(0.6)	157	(0.5)	0.2
Z48_ICD10	147	(0.2)	92	(0.2)	55	(0.2)	0.52
Z49_ICD10	42	(0.1)	29	(0.1)	13	(0.0)	0.23
Z51_ICD10	86	(0.1)	43	(0.1)	43	(0.1)	0.06
Z52_ICD10	61	(0.1)	31	(0.1)	30	(0.1)	0.14
Z55_ICD10	1	(0.0)	1	(0.0)	0	(0.0)	0.41
Z57_ICD10	1	(0.0)	1	(0.0)	0	(0.0)	0.41
Z62_ICD10	3	(0.0)	2	(0.0)	1	(0.0)	0.81
Z63_ICD10	12	(0.0)	10	(0.0)	2	(0.0)	0.1
Z64_ICD10	20	(0.0)	9	(0.0)	11	(0.0)	0.17
Z65_ICD10	19	(0.0)	14	(0.0)	5	(0.0)	0.22
Z71_ICD10	5407	(6.7)	3301	(6.9)	2106	(6.6)	0.1
Z72_ICD10	9	(0.0)	7	(0.0)	2	(0.0)	0.28
Z76_ICD10	2	(0.0)	0	(0.0)	2	(0.0)	0.08
Z77_ICD10	1	(0.0)	0	(0.0)	1	(0.0)	0.22
Z78_ICD10	2	(0.0)	2	(0.0)	0	(0.0)	0.25
Z79_ICD10	1	(0.0)	0	(0.0)	1	(0.0)	0.22
Z80_ICD10	75	(0.1)	52	(0.1)	23	(0.1)	0.1

Z82_ICD10	15	(0.0)	6	(0.0)	9	(0.0)	0.11
Z83_ICD10	26	(0.0)	19	(0.0)	7	(0.0)	0.17
Z84_ICD10	3	(0.0)	2	(0.0)	1	(0.0)	0.81
Z85_ICD10	29	(0.0)	21	(0.0)	8	(0.0)	0.17
Z86_ICD10	37	(0.0)	23	(0.0)	14	(0.0)	0.79
Z87_ICD10	30	(0.0)	18	(0.0)	12	(0.0)	1
Z88_ICD10	4	(0.0)	2	(0.0)	2	(0.0)	0.68
Z89_ICD10	16	(0.0)	5	(0.0)	11	(0.0)	0.02
Z90_ICD10	18	(0.0)	14	(0.0)	4	(0.0)	0.12
Z91_ICD10	2	(0.0)	1	(0.0)	1	(0.0)	0.77
Z92_ICD10	1	(0.0)	1	(0.0)	0	(0.0)	0.41
Z93_ICD10	41	(0.1)	27	(0.1)	14	(0.0)	0.44
Z94_ICD10	2268	(2.8)	1345	(2.8)	923	(2.9)	0.49
Z95_ICD10	326	(0.4)	202	(0.4)	124	(0.4)	0.47
Z96_ICD10	2749	(3.4)	1640	(3.4)	1109	(3.5)	0.71
Z97_ICD10	13	(0.0)	9	(0.0)	4	(0.0)	0.5
Z98_ICD10	212	(0.3)	121	(0.3)	91	(0.3)	0.38
Z99_ICD10	139	(0.2)	84	(0.2)	55	(0.2)	0.92
Chills	8190	(10.2)	4903	(10.2)	3287	(10.2)	0.79
Headache	3307	(4.1)	1983	(4.1)	1324	(4.1)	0.97
Myalgia	979	(1.2)	584	(1.2)	395	(1.2)	0.82
Arthralgia	85	(0.1)	49	(0.1)	36	(0.1)	0.66
Pain	18128	(22.6)	10916	(22.7)	7212	(22.5)	0.5
Weakness	6533	(8.1)	3866	(8.0)	2667	(8.3)	0.16
AMS	2155	(2.7)	1290	(2.7)	865	(2.7)	0.89
Syncope	252	(0.3)	156	(0.3)	96	(0.3)	0.54
Dizziness	2709	(3.4)	1623	(3.4)	1086	(3.4)	0.92
Seizures	338	(0.4)	207	(0.4)	131	(0.4)	0.64
Motor	185	(0.2)	106	(0.2)	79	(0.2)	0.45
Bulbar	95	(0.1)	60	(0.1)	35	(0.1)	0.53
Rhinorrhea	1021	(1.3)	615	(1.3)	406	(1.3)	0.88
Cough	11451	(14.3)	6895	(14.3)	4556	(14.2)	0.61
sputum	3479	(4.3)	2095	(4.4)	1384	(4.3)	0.79
SOB	9573	(11.9)	5815	(12.1)	3758	(11.7)	0.11
ChestPain	1039	(1.3)	617	(1.3)	422	(1.3)	0.68
Hoarseness	49	(0.1)	33	(0.1)	16	(0.0)	0.29
SoreThroat	2609	(3.3)	1610	(3.3)	999	(3.1)	0.07
Hemoptysis	244	(0.3)	137	(0.3)	107	(0.3)	0.22
Palpitations	351	(0.4)	217	(0.5)	134	(0.4)	0.48
Diaphoresis	226	(0.3)	139	(0.3)	87	(0.3)	0.64
Bradycardia	10	(0.0)	2	(0.0)	8	(0.0)	0.01
Choke	112	(0.1)	66	(0.1)	46	(0.1)	0.82
Cyanosis	26	(0.0)	18	(0.0)	8	(0.0)	0.34
Desat	65	(0.1)	36	(0.1)	29	(0.1)	0.45
Supp_O2	579	(0.7)	351	(0.7)	228	(0.7)	0.76
PPV	201	(0.3)	128	(0.3)	73	(0.2)	0.29
Erythema	2212	(2.8)	1300	(2.7)	912	(2.8)	0.23
Swelling	2208	(2.8)	1347	(2.8)	861	(2.7)	0.33

Discharge	251	(0.3)	148	(0.3)	103	(0.3)	0.74
Rash	933	(1.2)	547	(1.1)	386	(1.2)	0.39
LUTS	2676	(3.3)	1607	(3.3)	1069	(3.3)	0.95
FlankPain	1822	(2.3)	1123	(2.3)	699	(2.2)	0.15
Oliguria	104	(0.1)	62	(0.1)	42	(0.1)	0.94
anorexia	724	(0.9)	440	(0.9)	284	(0.9)	0.67
NauseaVomit	7435	(9.3)	4435	(9.2)	3000	(9.4)	0.52
AbdomenDistention	289	(0.4)	169	(0.4)	120	(0.4)	0.6
AbdominalPain	7368	(9.2)	4397	(9.1)	2971	(9.3)	0.55
Diarrhea	4268	(5.3)	2573	(5.3)	1695	(5.3)	0.69
GIB	723	(0.9)	437	(0.9)	286	(0.9)	0.81
Constipation	340	(0.4)	210	(0.4)	130	(0.4)	0.51
Hematemesis	236	(0.3)	144	(0.3)	92	(0.3)	0.75
Jaundice	293	(0.4)	178	(0.4)	115	(0.4)	0.79
Hiccup	31	(0.0)	14	(0.0)	17	(0.1)	0.09
Hyperglycemia	78	(0.1)	53	(0.1)	25	(0.1)	0.15
Hypoglycemia	9	(0.0)	4	(0.0)	5	(0.0)	0.34
HTN	75	(0.1)	41	(0.1)	34	(0.1)	0.35
hoTN	117	(0.1)	66	(0.1)	51	(0.2)	0.43
Fall	635	(0.8)	387	(0.8)	248	(0.8)	0.63
HTN_PHx	16756	(20.9)	9993	(20.8)	6763	(21.1)	0.28
DM_PHx	10871	(13.6)	6477	(13.5)	4394	(13.7)	0.34
CAD_PHx	3237	(4.0)	1963	(4.1)	1274	(4.0)	0.45
CVA_PHx	2764	(3.4)	1689	(3.5)	1075	(3.4)	0.23
BPH_PHx	2867	(3.6)	1710	(3.6)	1157	(3.6)	0.69
UTI_PHx	2450	(3.1)	1477	(3.1)	973	(3.0)	0.77
CKD_PHx	1881	(2.3)	1099	(2.3)	782	(2.4)	0.16
heartDz_PHx	2123	(2.6)	1275	(2.6)	848	(2.6)	0.96
HBV_PHx	2207	(2.8)	1361	(2.8)	846	(2.6)	0.1
COPD_PHx	1552	(1.9)	929	(1.9)	623	(1.9)	0.91
dementia_PHx	1734	(2.2)	1054	(2.2)	680	(2.1)	0.5
asthma_PHx	1607	(2.0)	966	(2.0)	641	(2.0)	0.93
HLD_PHx	1863	(2.3)	1149	(2.4)	714	(2.2)	0.14
pneumonia_PHx	1898	(2.4)	1132	(2.4)	766	(2.4)	0.75
lungCA_PHx	1916	(2.4)	1149	(2.4)	767	(2.4)	0.98
gout_PHx	1518	(1.9)	909	(1.9)	609	(1.9)	0.92
anemia_PHx	1797	(2.2)	1068	(2.2)	729	(2.3)	0.62
CHF_PHx	1202	(1.5)	702	(1.5)	500	(1.6)	0.25
liverCirrhosis_PHx	1373	(1.7)	819	(1.7)	554	(1.7)	0.79
Parkinsonism_PHx	1286	(1.6)	780	(1.6)	506	(1.6)	0.63
GU_PHx	1404	(1.8)	874	(1.8)	530	(1.7)	0.08
HCC_PHx	1412	(1.8)	833	(1.7)	579	(1.8)	0.44
HCV_PHx	1156	(1.4)	686	(1.4)	470	(1.5)	0.65
arrhythmia_PHx	993	(1.2)	585	(1.2)	408	(1.3)	0.48
lymphoma_PHx	1377	(1.7)	825	(1.7)	552	(1.7)	0.95
gallstone_PHx	1018	(1.3)	587	(1.2)	431	(1.3)	0.13
OA_PHx	828	(1.0)	494	(1.0)	334	(1.0)	0.84
CVD_PHx	1022	(1.3)	630	(1.3)	392	(1.2)	0.28

breastCA_PHx	1901	(2.4)	1158	(2.4)	743	(2.3)	0.41
Afib_PHx	720	(0.9)	411	(0.9)	309	(1.0)	0.11
colonCA_PHx	1100	(1.4)	645	(1.3)	455	(1.4)	0.35
depression_PHx	682	(0.9)	387	(0.8)	295	(0.9)	0.08
cellulitis_PHx	620	(0.8)	378	(0.8)	242	(0.8)	0.62
nephrolithiasis_PHx	791	(1.0)	468	(1.0)	323	(1.0)	0.63
SLE_PHx	681	(0.8)	391	(0.8)	290	(0.9)	0.17
constipation_PHx	444	(0.6)	261	(0.5)	183	(0.6)	0.6
insomnia_PHx	662	(0.8)	383	(0.8)	279	(0.9)	0.26
GERD_PHx	590	(0.7)	364	(0.8)	226	(0.7)	0.4
NPC_PHx	747	(0.9)	452	(0.9)	295	(0.9)	0.78
AML_PHx	729	(0.9)	420	(0.9)	309	(1.0)	0.19
ESRD_PHx	1495	(1.9)	865	(1.8)	630	(2.0)	0.09
epilepsy_PHx	499	(0.6)	311	(0.6)	188	(0.6)	0.29
prostateCA_PHx	543	(0.7)	330	(0.7)	213	(0.7)	0.71
TB_PHx	729	(0.9)	424	(0.9)	305	(1.0)	0.31
kidneyTx_PHx	869	(1.1)	523	(1.1)	346	(1.1)	0.91
cataract_PHx	572	(0.7)	344	(0.7)	228	(0.7)	0.95
DU_PHx	458	(0.6)	266	(0.6)	192	(0.6)	0.4
gastritis_PHx	429	(0.5)	245	(0.5)	184	(0.6)	0.22
anxiety_PHx	454	(0.6)	280	(0.6)	174	(0.5)	0.47
hemorrhoid_PHx	491	(0.6)	288	(0.6)	203	(0.6)	0.54
HIV_PHx	424	(0.5)	264	(0.5)	160	(0.5)	0.34
RA_PHx	362	(0.5)	236	(0.5)	126	(0.4)	0.04
hepatitis_PHx	2245	(2.8)	1396	(2.9)	849	(2.6)	0.03
bronchitis_PHx	416	(0.5)	240	(0.5)	176	(0.5)	0.34
liverTumor_PHx	416	(0.5)	246	(0.5)	170	(0.5)	0.72
pancreaticCA_PHx	513	(0.6)	308	(0.6)	205	(0.6)	0.99
refluxEsophagitis_PHx	310	(0.4)	180	(0.4)	130	(0.4)	0.49
gastricCA_PHx	520	(0.6)	298	(0.6)	222	(0.7)	0.21
URI_PHx	330	(0.4)	196	(0.4)	134	(0.4)	0.82
AMI_PHx	467	(0.6)	268	(0.6)	199	(0.6)	0.25
bladderCA_PHx	411	(0.5)	234	(0.5)	177	(0.6)	0.2
hypothyroidism_PHx	286	(0.4)	165	(0.3)	121	(0.4)	0.42
osteoporosis_PHx	285	(0.4)	185	(0.4)	100	(0.3)	0.09
PAOD_PHx	280	(0.3)	163	(0.3)	117	(0.4)	0.54
neurogenicBladder_PHx	281	(0.4)	166	(0.3)	115	(0.4)	0.75
appendicitis_PHx	285	(0.4)	181	(0.4)	104	(0.3)	0.23
bronchiectasis_PHx	277	(0.3)	165	(0.3)	112	(0.3)	0.88
HZV_PHx	332	(0.4)	189	(0.4)	143	(0.4)	0.25
ICH_PHx	391	(0.5)	250	(0.5)	141	(0.4)	0.11
GIB_PHx	236	(0.3)	137	(0.3)	99	(0.3)	0.54
MM_PHx	329	(0.4)	190	(0.4)	139	(0.4)	0.4
lumbarSpondylosis_PHx	271	(0.3)	181	(0.4)	90	(0.3)	0.02
lungTumor_PHx	286	(0.4)	177	(0.4)	109	(0.3)	0.51
hydronephrosis_PHx	338	(0.4)	210	(0.4)	128	(0.4)	0.42
liverTx_PHx	393	(0.5)	227	(0.5)	166	(0.5)	0.36
APN_PHx	338	(0.4)	213	(0.4)	125	(0.4)	0.26

rectalCA_PHx	356	(0.4)	214	(0.4)	142	(0.4)	0.97
hyperthyroidism_PHx	314	(0.4)	194	(0.4)	120	(0.4)	0.52
chemoRx_PHx	3364	(4.2)	2046	(4.3)	1318	(4.1)	0.32
BTI_PHx	307	(0.4)	187	(0.4)	120	(0.4)	0.74
goiter_PHx	295	(0.4)	178	(0.4)	117	(0.4)	0.91
glaucoma_PHx	302	(0.4)	183	(0.4)	119	(0.4)	0.83
MDS_PHx	312	(0.4)	177	(0.4)	135	(0.4)	0.24
hernia_PHx	376	(0.5)	229	(0.5)	147	(0.5)	0.72
urolithiasis_PHx	217	(0.3)	127	(0.3)	90	(0.3)	0.66
MVP_PHx	325	(0.4)	171	(0.4)	154	(0.5)	0.006
EV_PHx	200	(0.2)	118	(0.2)	82	(0.3)	0.77
uterineMyoma_PHx	359	(0.4)	204	(0.4)	155	(0.5)	0.22
pancreatitis_PHx	337	(0.4)	190	(0.4)	147	(0.5)	0.17
DVT_PHx	235	(0.3)	147	(0.3)	88	(0.3)	0.42
seizure_PHx	132	(0.2)	72	(0.1)	60	(0.2)	0.2
hyperuricemia_PHx	216	(0.3)	126	(0.3)	90	(0.3)	0.62
cervicalCA_PHx	303	(0.4)	176	(0.4)	127	(0.4)	0.5
CesareanSection_PHx	155	(0.2)	86	(0.2)	69	(0.2)	0.25
ileus_PHx	188	(0.2)	107	(0.2)	81	(0.3)	0.39
boneMetastasis_PHx	356	(0.4)	214	(0.4)	142	(0.4)	0.97
jaundice_PHx	194	(0.2)	137	(0.3)	57	(0.2)	0.003
cholecystitis_PHx	219	(0.3)	124	(0.3)	95	(0.3)	0.31
hematuria_PHx	165	(0.2)	93	(0.2)	72	(0.2)	0.34
compressionFx_PHx	181	(0.2)	108	(0.2)	73	(0.2)	0.93
PLE_PHx	199	(0.2)	125	(0.3)	74	(0.2)	0.42
esophCA_PHx	280	(0.3)	172	(0.4)	108	(0.3)	0.62
thrombocytopenia_PHx	213	(0.3)	133	(0.3)	80	(0.2)	0.47
chronicPancreatitis_PHx	161	(0.2)	95	(0.2)	66	(0.2)	0.8
arthritis_PHx	1287	(1.6)	788	(1.6)	499	(1.6)	0.36
leukemia_PHx	1078	(1.3)	646	(1.3)	432	(1.3)	0.96
chronicPeriodontitis_PHx	184	(0.2)	105	(0.2)	79	(0.2)	0.42
Sjogren_PHx	190	(0.2)	108	(0.2)	82	(0.3)	0.37
allergicRhinitis_PHx	174	(0.2)	110	(0.2)	64	(0.2)	0.39
thalassemia_PHx	208	(0.3)	123	(0.3)	85	(0.3)	0.8
chronicConjunctivitis_PHx	158	(0.2)	94	(0.2)	64	(0.2)	0.9
organicBrain_PHx	152	(0.2)	94	(0.2)	58	(0.2)	0.64
biliaryCA_PHx	158	(0.2)	93	(0.2)	65	(0.2)	0.77
colonPolyp_PHx	157	(0.2)	97	(0.2)	60	(0.2)	0.65
kidneyCA_PHx	229	(0.3)	133	(0.3)	96	(0.3)	0.55
AA_PHx	201	(0.3)	111	(0.2)	90	(0.3)	0.17
oralCA_PHx	220	(0.3)	119	(0.2)	101	(0.3)	0.07
choledocholithiasis_PHx	176	(0.2)	111	(0.2)	65	(0.2)	0.41
respFailure_PHx	141	(0.2)	87	(0.2)	54	(0.2)	0.68
boneMarrowTx_PHx	381	(0.5)	217	(0.5)	164	(0.5)	0.22
dizziness_PHx	101	(0.1)	64	(0.1)	37	(0.1)	0.49
Sicca_PHx	149	(0.2)	88	(0.2)	61	(0.2)	0.81
Psy_PHx	87	(0.1)	47	(0.1)	40	(0.1)	0.25
UGIB_PHx	94	(0.1)	64	(0.1)	30	(0.1)	0.11

PVD_PHx	142	(0.2)	86	(0.2)	56	(0.2)	0.89
pancreaticTumor_PHx	132	(0.2)	80	(0.2)	52	(0.2)	0.89
bronchopneumonia_PHx	115	(0.1)	66	(0.1)	49	(0.2)	0.57
VHD_PHx	233	(0.3)	147	(0.3)	86	(0.3)	0.33
tongueCA_PHx	222	(0.3)	136	(0.3)	86	(0.3)	0.7
lungFibrosis_PHx	129	(0.2)	78	(0.2)	51	(0.2)	0.91
hoTN_PHx	126	(0.2)	80	(0.2)	46	(0.1)	0.42
brainTumor_PHx	262	(0.3)	150	(0.3)	112	(0.3)	0.36
HIVD_PHx	162	(0.2)	97	(0.2)	65	(0.2)	0.97
cardiomegaly_PHx	102	(0.1)	54	(0.1)	48	(0.1)	0.15
functionalGI_PHx	124	(0.2)	73	(0.2)	51	(0.2)	0.8
cholangioCA_PHx	202	(0.3)	130	(0.3)	72	(0.2)	0.21
pressureUlcer_PHx	136	(0.2)	83	(0.2)	53	(0.2)	0.81
brainMetastasis_PHx	205	(0.3)	126	(0.3)	79	(0.2)	0.67
buccalCA_PHx	153	(0.2)	80	(0.2)	73	(0.2)	0.05
vertigo_PHx	194	(0.2)	128	(0.3)	66	(0.2)	0.09
sinusitis_PHx	180	(0.2)	101	(0.2)	79	(0.2)	0.29
femoralFx_PHx	140	(0.2)	85	(0.2)	55	(0.2)	0.86
polyneuropathy_PHx	117	(0.1)	76	(0.2)	41	(0.1)	0.27
eczema_PHx	119	(0.1)	71	(0.1)	48	(0.1)	0.94
schizophrenia_PHx	181	(0.2)	111	(0.2)	70	(0.2)	0.72
fattyLiver_PHx	134	(0.2)	81	(0.2)	53	(0.2)	0.92
MDD_PHx	83	(0.1)	44	(0.1)	39	(0.1)	0.19
ascites_PHx	115	(0.1)	72	(0.1)	43	(0.1)	0.57
liverAbscess_PHx	136	(0.2)	82	(0.2)	54	(0.2)	0.94
AKI_PHx	87	(0.1)	52	(0.1)	35	(0.1)	0.97
vasculitis_PHx	137	(0.2)	78	(0.2)	59	(0.2)	0.46
TIA_PHx	98	(0.1)	58	(0.1)	40	(0.1)	0.87
SSS_PHx	139	(0.2)	80	(0.2)	59	(0.2)	0.56
ovarianCA_PHx	258	(0.3)	158	(0.3)	100	(0.3)	0.68
thyroidCA_PHx	110	(0.1)	69	(0.1)	41	(0.1)	0.56
MR_PHx	110	(0.1)	59	(0.1)	51	(0.2)	0.17
cervicalSpondylosis_PHx	103	(0.1)	69	(0.1)	34	(0.1)	0.15
cachexia_PHx	77	(0.1)	46	(0.1)	31	(0.1)	0.96
SDH_PHx	112	(0.1)	71	(0.1)	41	(0.1)	0.46
dermatitis_PHx	153	(0.2)	89	(0.2)	64	(0.2)	0.64
pacemaker_PHx	300	(0.4)	170	(0.4)	130	(0.4)	0.24
CML_PHx	113	(0.1)	73	(0.2)	40	(0.1)	0.32
autoimmune_PHx	130	(0.2)	73	(0.2)	57	(0.2)	0.37
aspirationPNA_PHx	100	(0.1)	56	(0.1)	44	(0.1)	0.41
radiculopathy_PHx	69	(0.1)	40	(0.1)	29	(0.1)	0.73
heartTx_PHx	119	(0.1)	65	(0.1)	54	(0.2)	0.23
pseudophakia_PHx	81	(0.1)	51	(0.1)	30	(0.1)	0.59
renalDz_PHx	107	(0.1)	64	(0.1)	43	(0.1)	0.97
Alzheimer_PHx	117	(0.1)	67	(0.1)	50	(0.2)	0.55
abdominalPain_PHx	68	(0.1)	45	(0.1)	23	(0.1)	0.3
hypopharynxCA_PHx	155	(0.2)	98	(0.2)	57	(0.2)	0.41
breastTumor_PHx	139	(0.2)	79	(0.2)	60	(0.2)	0.45

dysphagia_PHx	90	(0.1)	56	(0.1)	34	(0.1)	0.67
myofascialPain_PHx	100	(0.1)	65	(0.1)	35	(0.1)	0.31
MG_PHx	80	(0.1)	42	(0.1)	38	(0.1)	0.17
AFL_PHx	80	(0.1)	49	(0.1)	31	(0.1)	0.82
edema_PHx	52	(0.1)	34	(0.1)	18	(0.1)	0.43
spineOP_PHx	79	(0.1)	49	(0.1)	30	(0.1)	0.71
oropharynxCA_PHx	86	(0.1)	57	(0.1)	29	(0.1)	0.23
osteomyelitis_PHx	60	(0.1)	35	(0.1)	25	(0.1)	0.79
sleep_PHx	82	(0.1)	41	(0.1)	41	(0.1)	0.06
SNHL_PHx	80	(0.1)	47	(0.1)	33	(0.1)	0.82
pulmEdema_PHx	63	(0.1)	37	(0.1)	26	(0.1)	0.84
urticaria_PHx	99	(0.1)	49	(0.1)	50	(0.2)	0.03
radioRx_PHx	551	(0.7)	313	(0.7)	238	(0.7)	0.12
tinea_PHx	96	(0.1)	52	(0.1)	44	(0.1)	0.24
abnlLFTs_PHx	102	(0.1)	62	(0.1)	40	(0.1)	0.87
endometrialCA_PHx	151	(0.2)	81	(0.2)	70	(0.2)	0.11
liverMetastasis_PHx	213	(0.3)	125	(0.3)	88	(0.3)	0.7
labyrintheInsufficiency_PHx	85	(0.1)	50	(0.1)	35	(0.1)	0.82
pancytopenia_PHx	83	(0.1)	44	(0.1)	39	(0.1)	0.19
neckMass_PHx	72	(0.1)	46	(0.1)	26	(0.1)	0.5
hyperparathyroidism_PHx	42	(0.1)	22	(0.0)	20	(0.1)	0.31
hypoK_PHx	53	(0.1)	32	(0.1)	21	(0.1)	0.96
AoS_PHx	70	(0.1)	48	(0.1)	22	(0.1)	0.14
oralUlcer_PHx	63	(0.1)	40	(0.1)	23	(0.1)	0.57
bipolar_PHx	95	(0.1)	56	(0.1)	39	(0.1)	0.83
hydrocephalus_PHx	122	(0.2)	81	(0.2)	41	(0.1)	0.15
renalTumor_PHx	90	(0.1)	60	(0.1)	30	(0.1)	0.2
G6PD_PHx	90	(0.1)	56	(0.1)	34	(0.1)	0.67
adrenalInsufficiency_PHx	74	(0.1)	42	(0.1)	32	(0.1)	0.57
cholecystectomy_PHx	101	(0.1)	56	(0.1)	45	(0.1)	0.35
Fever_PHx	79	(0.1)	55	(0.1)	24	(0.1)	0.08
emphysema_PHx	57	(0.1)	29	(0.1)	28	(0.1)	0.16
urineRetention_PHx	55	(0.1)	32	(0.1)	23	(0.1)	0.78
delirium_PHx	76	(0.1)	44	(0.1)	32	(0.1)	0.71
liverCyst_PHx	73	(0.1)	50	(0.1)	23	(0.1)	0.14
CP_PHx	57	(0.1)	37	(0.1)	20	(0.1)	0.45
chronicGN_PHx	76	(0.1)	51	(0.1)	25	(0.1)	0.21
spinalStenosis_PHx	39	(0.0)	25	(0.1)	14	(0.0)	0.6
trachealCA_PHx	98	(0.1)	67	(0.1)	31	(0.1)	0.09
gallbladderCA_PHx	71	(0.1)	35	(0.1)	36	(0.1)	0.07
HSV_PHx	86	(0.1)	45	(0.1)	41	(0.1)	0.15
alcoholicLiverCirrhosis_PHx	59	(0.1)	37	(0.1)	22	(0.1)	0.67
carotidStenosis_PHx	72	(0.1)	40	(0.1)	32	(0.1)	0.44
pulmHTN_PHx	65	(0.1)	37	(0.1)	28	(0.1)	0.61
nephroticSyndrome_PHx	73	(0.1)	45	(0.1)	28	(0.1)	0.77
PSA_PHx	69	(0.1)	47	(0.1)	22	(0.1)	0.17
backPain_PHx	62	(0.1)	43	(0.1)	19	(0.1)	0.13
UC_PHx	74	(0.1)	45	(0.1)	29	(0.1)	0.89

AoA_PHx	80	(0.1)	47	(0.1)	33	(0.1)	0.82
Fx_PHx	641	(0.8)	384	(0.8)	257	(0.8)	0.96
PSVT_PHx	82	(0.1)	48	(0.1)	34	(0.1)	0.79
psoriasis_PHx	74	(0.1)	37	(0.1)	37	(0.1)	0.08
chestPain_PHx	38	(0.0)	26	(0.1)	12	(0.0)	0.29
headache_PHx	49	(0.1)	33	(0.1)	16	(0.0)	0.29
larynxCA_PHx	79	(0.1)	44	(0.1)	35	(0.1)	0.43
splenomegaly_PHx	60	(0.1)	35	(0.1)	25	(0.1)	0.79
colonTumor_PHx	73	(0.1)	43	(0.1)	30	(0.1)	0.85
hyperK_PHx	57	(0.1)	41	(0.1)	16	(0.0)	0.07
abscess_PHx	380	(0.5)	222	(0.5)	158	(0.5)	0.53
PE_PHx	51	(0.1)	32	(0.1)	19	(0.1)	0.69
AGE_PHx	53	(0.1)	34	(0.1)	19	(0.1)	0.54
myeloproliferativeDz_PHx	50	(0.1)	28	(0.1)	22	(0.1)	0.56
palpitations_PHx	45	(0.1)	26	(0.1)	19	(0.1)	0.76
PID_PHx	1924	(2.4)	1188	(2.5)	736	(2.3)	0.11
aneurysm_PHx	152	(0.2)	91	(0.2)	61	(0.2)	0.97
hysterectomy_PHx	65	(0.1)	42	(0.1)	23	(0.1)	0.45
Graves_PHx	90	(0.1)	58	(0.1)	32	(0.1)	0.39
dentalCaries_PHx	49	(0.1)	30	(0.1)	19	(0.1)	0.86
AoD_PHx	85	(0.1)	47	(0.1)	38	(0.1)	0.38
proteinuria_PHx	46	(0.1)	29	(0.1)	17	(0.1)	0.67
hepaticComa_PHx	35	(0.0)	20	(0.0)	15	(0.0)	0.73
hemoptysis_PHx	37	(0.0)	22	(0.0)	15	(0.0)	0.95
AS_PHx	54	(0.1)	38	(0.1)	16	(0.0)	0.12
biliaryAtresia_PHx	41	(0.1)	26	(0.1)	15	(0.0)	0.66
tonsillitis_PHx	82	(0.1)	49	(0.1)	33	(0.1)	0.96
biliaryCirrhosis_PHx	52	(0.1)	31	(0.1)	21	(0.1)	0.95
hypoNa_PHx	59	(0.1)	40	(0.1)	19	(0.1)	0.22
TKR_PHx	130	(0.2)	70	(0.1)	60	(0.2)	0.15
NTM_PHx	54	(0.1)	33	(0.1)	21	(0.1)	0.87
Crohn_PHx	70	(0.1)	41	(0.1)	29	(0.1)	0.81
dryEye_PHx	59	(0.1)	32	(0.1)	27	(0.1)	0.37
bacteremia_PHx	42	(0.1)	30	(0.1)	12	(0.0)	0.13
transplant_PHx	1720	(2.1)	1003	(2.1)	717	(2.2)	0.15
hemolyticAnemia_PHx	85	(0.1)	40	(0.1)	45	(0.1)	0.01
neuropathy_PHx	216	(0.3)	130	(0.3)	86	(0.3)	0.96
AVshunt_PHx	753	(0.9)	428	(0.9)	325	(1.0)	0.08
meningitis_PHx	82	(0.1)	48	(0.1)	34	(0.1)	0.79
thyroidTumor_PHx	58	(0.1)	37	(0.1)	21	(0.1)	0.56
sepsis_PHx	86	(0.1)	49	(0.1)	37	(0.1)	0.57
MS_PHx	44	(0.1)	26	(0.1)	18	(0.1)	0.9
diarrhea_PHx	52	(0.1)	26	(0.1)	26	(0.1)	0.14
AVB_PHx	48	(0.1)	31	(0.1)	17	(0.1)	0.52
PCKD_PHx	77	(0.1)	52	(0.1)	25	(0.1)	0.18
PTX_PHx	91	(0.1)	56	(0.1)	35	(0.1)	0.76
conjunctivitis_PHx	213	(0.3)	121	(0.3)	92	(0.3)	0.34
adenomyosis_PHx	58	(0.1)	33	(0.1)	25	(0.1)	0.63

ureterCA_PHx	54	(0.1)	27	(0.1)	27	(0.1)	0.13
pelvicTumor_PHx	48	(0.1)	32	(0.1)	16	(0.0)	0.35
chronicGingivitis_PHx	39	(0.0)	21	(0.0)	18	(0.1)	0.43
ITP_PHx	72	(0.1)	40	(0.1)	32	(0.1)	0.44
MSA_PHx	54	(0.1)	40	(0.1)	14	(0.0)	0.03
CervicalCIN_PHx	25	(0.0)	10	(0.0)	15	(0.0)	0.04
thyroiditis_PHx	62	(0.1)	40	(0.1)	22	(0.1)	0.47
pneumoconiosis_PHx	46	(0.1)	32	(0.1)	14	(0.0)	0.19
tonsillarCA_PHx	71	(0.1)	48	(0.1)	23	(0.1)	0.19
dermatomyositis_PHx	47	(0.1)	33	(0.1)	14	(0.0)	0.15
macularLesion_PHx	69	(0.1)	39	(0.1)	30	(0.1)	0.56
ovarianTumor_PHx	83	(0.1)	54	(0.1)	29	(0.1)	0.35
GvHD_PHx	47	(0.1)	28	(0.1)	19	(0.1)	0.95
leukopenia_PHx	35	(0.0)	25	(0.1)	10	(0.0)	0.17
ovarianCyst_PHx	51	(0.1)	32	(0.1)	19	(0.1)	0.69
LAP_PHx	46	(0.1)	28	(0.1)	18	(0.1)	0.9
ventilator_PHx	56	(0.1)	38	(0.1)	18	(0.1)	0.23
bladderTumor_PHx	57	(0.1)	28	(0.1)	29	(0.1)	0.09
paronychia_PHx	36	(0.0)	22	(0.0)	14	(0.0)	0.89
folliculitis_PHx	37	(0.0)	24	(0.0)	13	(0.0)	0.55
meningioma_PHx	38	(0.0)	24	(0.0)	14	(0.0)	0.69
AIDS_PHx	72	(0.1)	34	(0.1)	38	(0.1)	0.03
esophagitis_PHx	347	(0.4)	206	(0.4)	141	(0.4)	0.81
panic_PHx	49	(0.1)	25	(0.1)	24	(0.1)	0.2
carpelTunnel_PHx	30	(0.0)	17	(0.0)	13	(0.0)	0.71
retainedDentRoot_PHx	29	(0.0)	17	(0.0)	12	(0.0)	0.88
leukocytosis_PHx	27	(0.0)	16	(0.0)	11	(0.0)	0.94
spondylolisthesis_PHx	39	(0.0)	23	(0.0)	16	(0.0)	0.9
tracheostomy_PHx	207	(0.3)	133	(0.3)	74	(0.2)	0.21
gingivaCA_PHx	38	(0.0)	20	(0.0)	18	(0.1)	0.35
VBI_PHx	31	(0.0)	14	(0.0)	17	(0.1)	0.09
OAB_PHx	39	(0.0)	22	(0.0)	17	(0.1)	0.65
adrenalTumor_PHx	42	(0.1)	26	(0.1)	16	(0.0)	0.8
perianalAbscess_PHx	30	(0.0)	12	(0.0)	18	(0.1)	0.03
Flu_PHx	46	(0.1)	27	(0.1)	19	(0.1)	0.86
BWL_PHx	32	(0.0)	22	(0.0)	10	(0.0)	0.31
cough_PHx	34	(0.0)	18	(0.0)	16	(0.0)	0.4
neuralgia_PHx	55	(0.1)	36	(0.1)	19	(0.1)	0.41
gallbladderPolyp_PHx	45	(0.1)	28	(0.1)	17	(0.1)	0.76
vaginitis_PHx	51	(0.1)	28	(0.1)	23	(0.1)	0.46
spondylosis_PHx	388	(0.5)	258	(0.5)	130	(0.4)	0.009
pruritus_PHx	38	(0.0)	23	(0.0)	15	(0.0)	0.95
encephalopathy_PHx	65	(0.1)	30	(0.1)	35	(0.1)	0.02
hemangioma_PHx	62	(0.1)	36	(0.1)	26	(0.1)	0.76
acuteBronchiolitis_PHx	22	(0.0)	17	(0.0)	5	(0.0)	0.1
syphilis_PHx	40	(0.0)	23	(0.0)	17	(0.1)	0.75
softTissueTumor_PHx	26	(0.0)	20	(0.0)	6	(0.0)	0.08
boneTumor_PHx	28	(0.0)	18	(0.0)	10	(0.0)	0.64

ILD_PHx	25	(0.0)	16	(0.0)	9	(0.0)	0.68
HPylori_PHx	33	(0.0)	15	(0.0)	18	(0.1)	0.09
hyperCa_PHx	18	(0.0)	9	(0.0)	9	(0.0)	0.39
ureteralStricture_PHx	29	(0.0)	22	(0.0)	7	(0.0)	0.08
SCI_PHx	62	(0.1)	41	(0.1)	21	(0.1)	0.32
seronegativeArthritis_PHx	27	(0.0)	15	(0.0)	12	(0.0)	0.64
menopause_PHx	43	(0.1)	25	(0.1)	18	(0.1)	0.8
vocalCordPalsy_PHx	18	(0.0)	8	(0.0)	10	(0.0)	0.18
eyeStrain_PHx	24	(0.0)	16	(0.0)	8	(0.0)	0.5
epididymitis_PHx	25	(0.0)	13	(0.0)	12	(0.0)	0.41
chronicPharyngitis_PHx	29	(0.0)	18	(0.0)	11	(0.0)	0.82
headInjury_PHx	30	(0.0)	16	(0.0)	14	(0.0)	0.46
dyspnea_PHx	21	(0.0)	14	(0.0)	7	(0.0)	0.53
osteopenia_PHx	23	(0.0)	12	(0.0)	11	(0.0)	0.44
polymyositis_PHx	26	(0.0)	16	(0.0)	10	(0.0)	0.87
pituitaryTumor_PHx	35	(0.0)	21	(0.0)	14	(0.0)	1
lungAbscess_PHx	33	(0.0)	23	(0.0)	10	(0.0)	0.26
DNI_PHx	19	(0.0)	11	(0.0)	8	(0.0)	0.85
VH_PHx	23	(0.0)	16	(0.0)	7	(0.0)	0.35
majorTrauma_PHx	36	(0.0)	24	(0.0)	12	(0.0)	0.41
oralCandidiasis_PHx	25	(0.0)	12	(0.0)	13	(0.0)	0.22
obesity_PHx	36	(0.0)	23	(0.0)	13	(0.0)	0.63
renalCyst_PHx	42	(0.1)	27	(0.1)	15	(0.0)	0.57
THR_PHx	18	(0.0)	11	(0.0)	7	(0.0)	0.92
thymicCA_PHx	29	(0.0)	20	(0.0)	9	(0.0)	0.32
humerusFx_PHx	24	(0.0)	18	(0.0)	6	(0.0)	0.13
hypopituitarism_PHx	31	(0.0)	17	(0.0)	14	(0.0)	0.56
essentialTremor_PHx	19	(0.0)	9	(0.0)	10	(0.0)	0.26
peritonitis_PHx	80	(0.1)	48	(0.1)	32	(0.1)	1
PBSCT_PHx	41	(0.1)	24	(0.0)	17	(0.1)	0.85
scoliosis_PHx	27	(0.0)	20	(0.0)	7	(0.0)	0.14
PPU_PHx	84	(0.1)	52	(0.1)	32	(0.1)	0.72
DCM_PHx	32	(0.0)	22	(0.0)	10	(0.0)	0.31
scabies_PHx	24	(0.0)	11	(0.0)	13	(0.0)	0.16
AR_PHx	38	(0.0)	24	(0.0)	14	(0.0)	0.69
cornealErosion_PHx	17	(0.0)	10	(0.0)	7	(0.0)	0.92
pemphigoid_PHx	26	(0.0)	14	(0.0)	12	(0.0)	0.52
gastricTumor_PHx	29	(0.0)	14	(0.0)	15	(0.0)	0.2
OSA_PHx	22	(0.0)	9	(0.0)	13	(0.0)	0.07
candidiasis_PHx	79	(0.1)	46	(0.1)	33	(0.1)	0.75
GV_PHx	32	(0.0)	22	(0.0)	10	(0.0)	0.31
NF_PHx	18	(0.0)	11	(0.0)	7	(0.0)	0.92
macrocyticAnemia_PHx	26	(0.0)	15	(0.0)	11	(0.0)	0.81
septicArthritis_PHx	17	(0.0)	13	(0.0)	4	(0.0)	0.17
flankPain_PHx	13	(0.0)	6	(0.0)	7	(0.0)	0.31
CLL_PHx	37	(0.0)	22	(0.0)	15	(0.0)	0.95
migraine_PHx	28	(0.0)	16	(0.0)	12	(0.0)	0.76
infertility_PHx	26	(0.0)	16	(0.0)	10	(0.0)	0.87

hemodialysis_PHx	1429	(1.8)	821	(1.7)	608	(1.9)	0.05
brainCA_PHx	18	(0.0)	12	(0.0)	6	(0.0)	0.56
empyema_PHx	24	(0.0)	13	(0.0)	11	(0.0)	0.56
polio_PHx	26	(0.0)	17	(0.0)	9	(0.0)	0.58
vulvitis_PHx	24	(0.0)	13	(0.0)	11	(0.0)	0.56
MUP_PHx	17	(0.0)	12	(0.0)	5	(0.0)	0.37
IE_PHx	34	(0.0)	22	(0.0)	12	(0.0)	0.58
skinTumor_PHx	15	(0.0)	10	(0.0)	5	(0.0)	0.6
lungMetastasis_PHx	159	(0.2)	90	(0.2)	69	(0.2)	0.38
thymicTumor_PHx	38	(0.0)	24	(0.0)	14	(0.0)	0.69
incontinence_PHx	55	(0.1)	33	(0.1)	22	(0.1)	1
IBS_PHx	27	(0.0)	16	(0.0)	11	(0.0)	0.94
urineFrequency_PHx	11	(0.0)	8	(0.0)	3	(0.0)	0.39
dermatophytosis_PHx	17	(0.0)	13	(0.0)	4	(0.0)	0.17
analFistula_PHx	50	(0.1)	36	(0.1)	14	(0.0)	0.08
SAH_PHx	52	(0.1)	36	(0.1)	16	(0.0)	0.17
CMV_PHx	25	(0.0)	15	(0.0)	10	(0.0)	1
VSD_PHx	27	(0.0)	13	(0.0)	14	(0.0)	0.21
prostateTumor_PHx	21	(0.0)	8	(0.0)	13	(0.0)	0.04
floater_PHx	19	(0.0)	12	(0.0)	7	(0.0)	0.78
frozenShoulder_PHx	16	(0.0)	11	(0.0)	5	(0.0)	0.47
bedRidden_PHx	42	(0.1)	24	(0.0)	18	(0.1)	0.71
liverDz_PHx	45	(0.1)	25	(0.1)	20	(0.1)	0.54
fibromyalgia_PHx	16	(0.0)	11	(0.0)	5	(0.0)	0.47
GIST_PHx	27	(0.0)	18	(0.0)	9	(0.0)	0.48
otitis_PHx	51	(0.1)	33	(0.1)	18	(0.1)	0.49
parotidTumor_PHx	15	(0.0)	12	(0.0)	3	(0.0)	0.11
UterineCA_PHx	22	(0.0)	15	(0.0)	7	(0.0)	0.43
spineFx_PHx	17	(0.0)	10	(0.0)	7	(0.0)	0.92
coagulopathy_PHx	13	(0.0)	7	(0.0)	6	(0.0)	0.65
duodenumCA_PHx	24	(0.0)	12	(0.0)	12	(0.0)	0.32
hemochromatosis_PHx	10	(0.0)	6	(0.0)	4	(0.0)	1
PD_PHx	309	(0.4)	181	(0.4)	128	(0.4)	0.61
endometriosis_PHx	41	(0.1)	25	(0.1)	16	(0.0)	0.9
neutropenia_PHx	16	(0.0)	8	(0.0)	8	(0.0)	0.41
ocularHTN_PHx	17	(0.0)	10	(0.0)	7	(0.0)	0.92
alcoholism_PHx	115	(0.1)	77	(0.2)	38	(0.1)	0.13
ALL_PHx	23	(0.0)	14	(0.0)	9	(0.0)	0.93
cystocele_PHx	18	(0.0)	10	(0.0)	8	(0.0)	0.7
PVC_PHx	15	(0.0)	11	(0.0)	4	(0.0)	0.29
renalAbscess_PHx	21	(0.0)	12	(0.0)	9	(0.0)	0.79
dystonia_PHx	18	(0.0)	14	(0.0)	4	(0.0)	0.12
enteritis_PHx	100	(0.1)	66	(0.1)	34	(0.1)	0.22
ALS_PHx	18	(0.0)	11	(0.0)	7	(0.0)	0.92
thrombocytosis_PHx	16	(0.0)	12	(0.0)	4	(0.0)	0.22
syncope_PHx	21	(0.0)	11	(0.0)	10	(0.0)	0.48
pleurisy_PHx	14	(0.0)	8	(0.0)	6	(0.0)	0.83
adjustmentDz_PHx	18	(0.0)	9	(0.0)	9	(0.0)	0.39

tinnitus_PHx	20	(0.0)	11	(0.0)	9	(0.0)	0.65
pregnancy_PHx	48	(0.1)	28	(0.1)	20	(0.1)	0.81
aphasia_PHx	13	(0.0)	11	(0.0)	2	(0.0)	0.07
ankleFx_PHx	17	(0.0)	12	(0.0)	5	(0.0)	0.37
hyperglycemia_PHx	17	(0.0)	9	(0.0)	8	(0.0)	0.55
CCRT_PHx	146	(0.2)	94	(0.2)	52	(0.2)	0.28

Abbreviations: ICD-10, International Statistical Classification of Diseases and Related Health Problems 10th Revision; PHx, past history; EMS, Emergency medical service; BP, Blood pressure; BMI, Body Mass Index; GCS, Glasgow Coma Scale; AMS, Altered mental status; SOB, Short of breath; Desat. Desaturation; Supp_O2; Oxygen supplement; PPV, Positive pressure ventilation; GIB, Gastrointestinal bleeding; HTN, Hypertension; HoTN, Hypotension; DM, Diabetes mellitus; CAD, Coronary artery disease; CVA, Cerebrovascular disease; BPH, Benign prostatic hyperplasia; UTI, Urinary tract infection; CKD, Chronic kidney disease; HeartDz, Heaert Disease; HBV, Hepatitis B virus; COPD, Chronic Obstruction Pulmonary Disease; HLD, Hyperlipidemia; LungCA, Lung cancer; GU, Gastric ulcer; HCC, Hepatocellular carcinoma; HCV, Hepatitis C virus; OA, Osteoarthritis; CVD, Cardiovascular disease; breastCA, Breast cancer; Afib, Atrial fibrilattion; ColonCA, Colon cancer; SLE, Systemic Lupus Erythematosus; GERD, Gastroesophageal reflux disease; NPC, Nasopharyngeal carcinoma; AML, Acute myeloid leukemia; ESRD, End-Stage Renal Disease; prostateCA, Prostate cancer; TB, Tuberculosis; kidneyTx, Kidney transplant; DU, Duodenal ulcer; HIV, Human Immunodeficiency Virus; RA, Rheumatoid arthritis; PancreaticCA, Pancreatic cancer; gastricCA, Gastric cancer; URI, Upper respiratory tract infection; AMI, Acute myocardial infarction; bladderCA, Bladder cancer; PAOD, Peripheral Arterial Occlusion Disease; HZV, Herpes zoster virus; ICH, Intracranial hemorrhage; GIB, Gastrointestinal bleeding; MM, Multiple myeloma; liverTx, Liver transplant; APN, Acute pyelonephritis; rectalCA, Rectal cancer; chemoRx, chemotherapy; BTI, Biliary tract infection; MDS, Myelodysplastic Syndromes; MVP, Mitral valve prolapse; EV, Esophageal varices; DVT, Deep vein thrombosis; cervicalCA, Cervical cancer; compressionFx, Compression fracture; PLE, pleural effusion; esophCA, Esophageal cancer; biliaryCA, Billiary cancer; KidneyCA, Kidney cancer; AA, Aplastic anemia; respFailure, Respiratory failure; boneMarrowTx, Bone marrow transplant; Psy, Psychiatric disease; UGIB, Upper gastrointestinal bleeding; PVD, Peripheral vascular disease; VHD, Valvular heart disease; tongueCA, Tongue cancer; HIVD, Herniation of intervertebral disc; functionalGI, Functional gastrointestinal disorders; cholangioCA, Cholangiocarcinoma; buccalCA, Buccal cancer; femoralFx, Femoral fracture; MDD, Major depressive disorder; AKI, Acute kidney injury; TIA, Transient ischemic attack; SSS, Sick sinus syndrome; ovarianCA, Ovarian cancer; thyroidCA, Thyroid cancer; MR, Mitral regurgitation; SDH, Subdural hematoma; CML, chronic myeloid leukemia; Autoimmune, Autoimmune disease; aspirationPNA, Aspiration pneumonia; heartTx, Heaert Transplant; renalDz, Renal disease; hypopharynxCA, Hypopharyngeal carcinoma; MG, Myasthenia Gravis; AFL, Atrial flutter; spineOP, Spine operation; oropharynxCA, Oropharyngeal cancer; SNHL, Sensorineural hearing loss; pulmEdema, Pulmonary edema; radioRx, Radiotherapy; abnlLFTs, Abnormal liver function; endometrialCA, Endometrial cancer; hypoK, Hypokalemia; AoS, Aortic stenosis; CP, Cerebral palsy; Chronic GN, Chronic Glomerulonephritis; trachealCA, Tracheal cancer; gallbladderCA, Gallbladder cancer; HSV, herpes simplex virus; pulmHTN, Pulmonary hypertension; PSA, Elevated prostate specific antigen; UC, Ulcerative colitis; AoA, Aortic aneurysm; Fx, Fracture; PSVT, Paroxysmal supraventricular tachycardia; larynxCA, Laryngeal cancer; HyperK, Hyperkalemia; PE, Pulmoanry embolism; AGE, Acute gastroenteritis; myeloproliferativeDz, Myeloproliferative disease; PID, Pelvic inflammatory disease; AoD, Dissection of aorta; AsS, Aoric stenosis; hypoNa, Hyponatremia; TKR, Total knee replacement;

NTM, Non-tuberculosis mycobacterium; AVshunt, Arteriovenous shunt; MS, Multiple sclerosis; AVB, Atrioventricular block; PCKD, Polycystic kidney disease; PTX, Pneumothorax; ureterCA, Ureter Cancer; ITP, Immune Thrombocytopenia Purpura; MSA , multiple system atrophy; CervicalCIN, Cervical intraepithelial neoplasia; tonsillarCA, Tonsillar cancer; GVHD, Graft-versus-host disease; LAP, Lymphadenopathy; AIDS, Acquired Immunodeficiency Syndrome; gingivaCA,Gingival cancer; VBI, Vertebrobasilar insufficiency; OAB, Overactive bladder; Flu, Influenza; BWL, Body weight loss; ILD, Interstitial lung disease; Hpylori, Helicobacter pylori; hyperCa, Hypercalcemia; SCI, Spinal cord injury; DNI, Deep neck infection; VH, Vitreous hemorrhage; THR, Total hip replacement; thymicCA, Thymic cancer; humerusFx, Humerus fracture; PBSCT, Peripheral Blood Stem Cell Transplantation; PPU, Perforated peptic ulcer; DCM, Dilated cardiomyopathy; AR, Aortic regurgitation; OSA, Obstructive sleep apnea; GV, Gastric varices; NF, Necrotizing fasciitis; CLL, Chronic lymphocytic leukemia; brainCA, Brain cancer; MUP, Metastatic cancer of unknown primary; IE, Infective endocarditis; IBS, Irritable bowel syndrome; SAH, Subarachnoid hemorrhage; CMV, Cytomegalovirus; VSD, Ventricular Septal Defect; GIST, Gastrointestinal stromal tumor; UterineCA, Uterine cancer; spineFx, Spine fracture; duodenumCA, Duodenal cancer; PD, Peritoneal dialysis; ocularHTN, Ocular Hypertension; ALL, Acute lymphoblastic leukemia; PVC, Premature ventricular complex; ALS, Amyotrophic lateral sclerosis; adjustmentDz, Adjustment disorder; ankleFx, Ankle fracture; CCRT, Concurrent chemoradiotherapy
