# Supplementary material for: Developing Machine-Learning Models to Predict Bacteremia in Febrile Adults Presenting to the Emergency Department: A Retrospective Cohort Study from a Large Center
Source: West J Emerg Med. 2025 May 30;26(3):617–26. doi: 10.5811/westjem.35866 (PMC12208070; doi:10.5811/westjem.35866)
Supplement: Supplementary file 2 [file wjem-26-617-s002.pdf]

Supplementary Table 2. Characteristics and univariate analyses of variables (features) between patients with or without bacteremia on the training/validation and testing cohorts (complete list).

Cohort	Training/Validation					Testing Cohort				
Variables (Features)	BACTEREMIA (-) (n=42298)		BACTEREMIA (+) (n=5822)		P value	BACTEREMIA (-) (n=28248)		BACTEREMIA (+) (n=3833)		P value
GENDER					0.04					0.04
Female	21147	(50.0)	2996	(51.5)		14097	(49.9)	1980	(51.7)	
Male	21151	(50.0)	2826	(48.5)		14151	(50.1)	1853	(48.3)	
AGE, Mean(SD)	58.3	(20.3)	66.4	(16.3)	<0.001	58.4	(20.3)	66.6	(16.4)	<0.001
BODY_TEMPERATURE, Mean(SD)	38.4	(1.3)	38.8	(1.4)	<0.001	38.4	(1.5)	38.8	(1.3)	<0.001
ACUTE CHANGE					<0.001					<0.001
No	38090	(90.1)	5052	(86.8)		25425	(90.0)	3309	(86.3)	
Yes	1229	(2.9)	332	(5.7)		780	(2.8)	229	(6.0)	
NA	2979	(7.0)	438	(7.5)		2043	(7.2)	295	(7.7)	
EMS					0.005					0.72
No	33974	(80.3)	4712	(80.9)		22687	(80.3)	3113	(81.2)	
Yes	35	(0.1)	12	(0.2)		19	(0.1)	2	(0.1)	
NA	8289	(19.6)	1098	(18.9)		5542	(19.6)	718	(18.7)	
TRIAGE					<0.001					<0.001
1	1616	(3.8)	502	(8.6)		1038	(3.7)	321	(8.4)	
2	10618	(25.1)	1830	(31.4)		7133	(25.3)	1189	(31.0)	
3	29392	(69.5)	3459	(59.4)		19619	(69.5)	2299	(60.0)	
4	639	(1.5)	30	(0.5)		434	(1.5)	24	(0.6)	
5	33	(0.1)	1	(0.0)		24	(0.1)	0	(0.0)	
SYSTOLIC, Mean(SD)	132.7	(26.4)	131.2	(30.8)	<0.001	132.8	(26.7)	130.7	(30.6)	<0.001
DIASTOLIC, Mean(SD)	76.1	(14.9)	72.8	(16.2)	<0.001	76.1	(14.9)	72.4	(16.4)	<0.001
PULSE, Mean(SD)	105.7	(19.3)	111.1	(21.5)	<0.001	105.3	(19.4)	110.5	(21.6)	<0.001
OXYGEN, Mean(SD)	96	(3.5)	95.6	(4.1)	<0.001	96	(3.6)	95.7	(4.2)	<0.001
RESPIRATION, Mean(SD)	19.5	(4.7)	20.1	(5.2)	<0.001	19.5	(6.7)	20	(3.8)	<0.001

PAININDEX					<0.001					<0.001
0	30657	(72.5)	4428	(76.1)		20397	(72.2)	2942	(76.8)	
1	21	(0.0)	1	(0.0)		25	(0.1)	2	(0.1)	
2	179	(0.4)	8	(0.1)		114	(0.4)	13	(0.3)	
3	460	(1.1)	50	(0.9)		315	(1.1)	22	(0.6)	
4	1303	(3.1)	141	(2.4)		897	(3.2)	89	(2.3)	
5	2879	(6.8)	303	(5.2)		1939	(6.9)	194	(5.1)	
6	1494	(3.5)	165	(2.8)		974	(3.4)	116	(3.0)	
7	2112	(5.0)	275	(4.7)		1333	(4.7)	147	(3.8)	
8	1323	(3.1)	165	(2.8)		940	(3.3)	121	(3.2)	
9	246	(0.6)	43	(0.7)		184	(0.7)	21	(0.5)	
10	311	(0.7)	40	(0.7)		203	(0.7)	42	(1.1)	
NA	1313	(3.1)	203	(3.5)		927	(3.3)	124	(3.2)	
HEIGHT, Mean(SD)	162	(10.4)	160.4	(11.5)	<0.001	162.1	(10.2)	160.3	(10.6)	<0.001
WEIGHT, Mean(SD)	61.2	(23.5)	60.3	(13.3)	0.01	61.1	(13.6)	60.4	(12.9)	0.001
BMI, Mean(SD)	23.2	(4.4)	23.4	(4.6)	0.003	23.2	(4.4)	23.3	(4.4)	0.04
GCS_TYPE					<0.001					<0.001
NA	3134	(7.4)	461	(7.9)		2157	(7.6)	306	(8.0)	
clear_consciousness	34958	(82.6)	4556	(78.3)		23390	(82.8)	3013	(78.6)	
minor_coma	634	(1.5)	132	(2.3)		384	(1.4)	90	(2.3)	
moderate_coma	1768	(4.2)	387	(6.6)		1147	(4.1)	251	(6.5)	
others	929	(2.2)	114	(2.0)		575	(2.0)	68	(1.8)	
severe_coma	875	(2.1)	172	(3.0)		595	(2.1)	105	(2.7)	
A00_ICD10	3	(0.0)	2	(0.0)	0.06	0	(0.0)	0	(0.0)	1
A01_ICD10	1	(0.0)	0	(0.0)	0.71	0	(0.0)	1	(0.0)	0.007
A02_ICD10	25	(0.1)	5	(0.1)	0.44	29	(0.1)	3	(0.1)	0.65
A03_ICD10	3	(0.0)	0	(0.0)	0.52	0	(0.0)	0	(0.0)	1
A04_ICD10	59	(0.1)	9	(0.2)	0.77	40	(0.1)	13	(0.3)	0.005
A05_ICD10	5	(0.0)	0	(0.0)	0.41	0	(0.0)	2	(0.1)	<0.001
A06_ICD10	15	(0.0)	1	(0.0)	0.47	14	(0.0)	0	(0.0)	0.17
A07_ICD10	9	(0.0)	1	(0.0)	0.84	4	(0.0)	0	(0.0)	0.46
A08_ICD10	15	(0.0)	0	(0.0)	0.15	8	(0.0)	2	(0.1)	0.43

A09_ICD10	242	(0.6)	38	(0.7)	0.45	163	(0.6)	26	(0.7)	0.44
A15_ICD10	670	(1.6)	76	(1.3)	0.11	458	(1.6)	44	(1.1)	0.03
A17_ICD10	29	(0.1)	2	(0.0)	0.33	9	(0.0)	1	(0.0)	0.85
A18_ICD10	78	(0.2)	13	(0.2)	0.52	53	(0.2)	8	(0.2)	0.78
A19_ICD10	26	(0.1)	6	(0.1)	0.25	21	(0.1)	1	(0.0)	0.28
A24_ICD10	2	(0.0)	0	(0.0)	0.6	1	(0.0)	1	(0.0)	0.1
A27_ICD10	58	(0.1)	5	(0.1)	0.31	29	(0.1)	3	(0.1)	0.65
A28_ICD10	5	(0.0)	1	(0.0)	0.73	2	(0.0)	0	(0.0)	0.6
A31_ICD10	163	(0.4)	14	(0.2)	0.09	103	(0.4)	8	(0.2)	0.12
A37_ICD10	4	(0.0)	1	(0.0)	0.59	1	(0.0)	0	(0.0)	0.71
A38_ICD10	1	(0.0)	0	(0.0)	0.71	0	(0.0)	0	(0.0)	1
A39_ICD10	1	(0.0)	1	(0.0)	0.1	1	(0.0)	0	(0.0)	0.71
A40_ICD10	12	(0.0)	2	(0.0)	0.8	5	(0.0)	4	(0.1)	0.003
A41_ICD10	425	(1.0)	100	(1.7)	<0.001	271	(1.0)	77	(2.0)	<0.001
A42_ICD10	2	(0.0)	0	(0.0)	0.6	4	(0.0)	0	(0.0)	0.46
A43_ICD10	2	(0.0)	0	(0.0)	0.6	1	(0.0)	0	(0.0)	0.71
A46_ICD10	17	(0.0)	0	(0.0)	0.13	12	(0.0)	3	(0.1)	0.34
A48_ICD10	27	(0.1)	3	(0.1)	0.72	21	(0.1)	1	(0.0)	0.28
A49_ICD10	16	(0.0)	5	(0.1)	0.1	14	(0.0)	2	(0.1)	0.95
A50_ICD10	1	(0.0)	0	(0.0)	0.71	0	(0.0)	0	(0.0)	1
A51_ICD10	13	(0.0)	3	(0.1)	0.41	10	(0.0)	1	(0.0)	0.77
A52_ICD10	9	(0.0)	3	(0.1)	0.17	14	(0.0)	2	(0.1)	0.95
A53_ICD10	119	(0.3)	12	(0.2)	0.3	108	(0.4)	5	(0.1)	0.01
A54_ICD10	15	(0.0)	2	(0.0)	0.97	8	(0.0)	0	(0.0)	0.3
A55_ICD10	1	(0.0)	1	(0.0)	0.1	2	(0.0)	1	(0.0)	0.25
A59_ICD10	6	(0.0)	0	(0.0)	0.36	4	(0.0)	0	(0.0)	0.46
A60_ICD10	34	(0.1)	1	(0.0)	0.09	26	(0.1)	5	(0.1)	0.47
A63_ICD10	33	(0.1)	4	(0.1)	0.81	19	(0.1)	4	(0.1)	0.42
A64_ICD10	4	(0.0)	2	(0.0)	0.11	5	(0.0)	0	(0.0)	0.41
A66_ICD10	8	(0.0)	0	(0.0)	0.29	3	(0.0)	0	(0.0)	0.52
A69_ICD10	11	(0.0)	1	(0.0)	0.69	2	(0.0)	1	(0.0)	0.25
A71_ICD10	13	(0.0)	2	(0.0)	0.88	9	(0.0)	1	(0.0)	0.85

A74_ICD10	3	(0.0)	0	(0.0)	0.52	4	(0.0)	0	(0.0)	0.46
A75_ICD10	75	(0.2)	7	(0.1)	0.32	37	(0.1)	6	(0.2)	0.68
A77_ICD10	1	(0.0)	0	(0.0)	0.71	0	(0.0)	0	(0.0)	1
A78_ICD10	36	(0.1)	2	(0.0)	0.2	20	(0.1)	2	(0.1)	0.68
A79_ICD10	5	(0.0)	2	(0.0)	0.18	0	(0.0)	1	(0.0)	0.007
A80_ICD10	6	(0.0)	0	(0.0)	0.36	2	(0.0)	1	(0.0)	0.25
A81_ICD10	8	(0.0)	2	(0.0)	0.44	4	(0.0)	0	(0.0)	0.46
A83_ICD10	13	(0.0)	0	(0.0)	0.18	8	(0.0)	0	(0.0)	0.3
A85_ICD10	1	(0.0)	0	(0.0)	0.71	1	(0.0)	0	(0.0)	0.71
A87_ICD10	4	(0.0)	0	(0.0)	0.46	1	(0.0)	0	(0.0)	0.71
A88_ICD10	4	(0.0)	0	(0.0)	0.46	2	(0.0)	0	(0.0)	0.6
A90_ICD10	15	(0.0)	0	(0.0)	0.15	7	(0.0)	0	(0.0)	0.33
A92_ICD10	3	(0.0)	0	(0.0)	0.52	1	(0.0)	0	(0.0)	0.71
A95_ICD10	1	(0.0)	0	(0.0)	0.71	0	(0.0)	0	(0.0)	1
A98_ICD10	15	(0.0)	2	(0.0)	0.97	5	(0.0)	2	(0.1)	0.18
B00_ICD10	435	(1.0)	76	(1.3)	0.05	325	(1.2)	43	(1.1)	0.88
B01_ICD10	26	(0.1)	1	(0.0)	0.18	7	(0.0)	1	(0.0)	0.96
B02_ICD10	1294	(3.1)	208	(3.6)	0.03	843	(3.0)	139	(3.6)	0.03
B05_ICD10	4	(0.0)	0	(0.0)	0.46	0	(0.0)	0	(0.0)	1
B06_ICD10	3	(0.0)	0	(0.0)	0.52	0	(0.0)	0	(0.0)	1
B07_ICD10	442	(1.0)	60	(1.0)	0.92	276	(1.0)	60	(1.6)	<0.001
B08_ICD10	18	(0.0)	0	(0.0)	0.12	17	(0.1)	1	(0.0)	0.4
B09_ICD10	9	(0.0)	0	(0.0)	0.27	4	(0.0)	0	(0.0)	0.46
B15_ICD10	10	(0.0)	0	(0.0)	0.24	9	(0.0)	0	(0.0)	0.27
B16_ICD10	496	(1.2)	95	(1.6)	0.003	313	(1.1)	65	(1.7)	0.002
B17_ICD10	375	(0.9)	89	(1.5)	<0.001	277	(1.0)	58	(1.5)	0.002
B18_ICD10	2067	(4.9)	373	(6.4)	<0.001	1344	(4.8)	253	(6.6)	<0.001
B19_ICD10	109	(0.3)	15	(0.3)	1	64	(0.2)	15	(0.4)	0.05
B20_ICD10	292	(0.7)	31	(0.5)	0.17	191	(0.7)	15	(0.4)	0.04
B25_ICD10	55	(0.1)	8	(0.1)	0.88	39	(0.1)	9	(0.2)	0.15
B26_ICD10	4	(0.0)	2	(0.0)	0.11	2	(0.0)	1	(0.0)	0.25
B27_ICD10	6	(0.0)	0	(0.0)	0.36	5	(0.0)	1	(0.0)	0.72

B30_ICD10	33	(0.1)	5	(0.1)	0.84	16	(0.1)	2	(0.1)	0.91
B33_ICD10	4	(0.0)	0	(0.0)	0.46	4	(0.0)	0	(0.0)	0.46
B34_ICD10	1	(0.0)	0	(0.0)	0.71	0	(0.0)	0	(0.0)	1
B35_ICD10	1958	(4.6)	267	(4.6)	0.88	1255	(4.4)	192	(5.0)	0.11
B36_ICD10	133	(0.3)	14	(0.2)	0.34	96	(0.3)	9	(0.2)	0.29
B37_ICD10	719	(1.7)	100	(1.7)	0.92	463	(1.6)	60	(1.6)	0.74
B38_ICD10	1	(0.0)	0	(0.0)	0.71	1	(0.0)	0	(0.0)	0.71
B39_ICD10	1	(0.0)	0	(0.0)	0.71	0	(0.0)	0	(0.0)	1
B44_ICD10	95	(0.2)	26	(0.4)	0.002	64	(0.2)	10	(0.3)	0.68
B45_ICD10	30	(0.1)	8	(0.1)	0.09	19	(0.1)	3	(0.1)	0.81
B46_ICD10	0	(0.0)	1	(0.0)	0.007	0	(0.0)	0	(0.0)	1
B48_ICD10	69	(0.2)	6	(0.1)	0.28	48	(0.2)	0	(0.0)	0.01
B49_ICD10	4	(0.0)	0	(0.0)	0.46	11	(0.0)	0	(0.0)	0.22
B54_ICD10	0	(0.0)	0	(0.0)	1	2	(0.0)	0	(0.0)	0.6
B57_ICD10	0	(0.0)	0	(0.0)	1	2	(0.0)	0	(0.0)	0.6
B58_ICD10	11	(0.0)	0	(0.0)	0.22	9	(0.0)	1	(0.0)	0.85
B59_ICD10	75	(0.2)	10	(0.2)	0.92	54	(0.2)	2	(0.1)	0.05
B60_ICD10	0	(0.0)	0	(0.0)	1	1	(0.0)	0	(0.0)	0.71
B65_ICD10	2	(0.0)	0	(0.0)	0.6	0	(0.0)	0	(0.0)	1
B66_ICD10	2	(0.0)	0	(0.0)	0.6	0	(0.0)	0	(0.0)	1
B68_ICD10	1	(0.0)	0	(0.0)	0.71	0	(0.0)	0	(0.0)	1
B75_ICD10	3	(0.0)	0	(0.0)	0.52	3	(0.0)	0	(0.0)	0.52
B79_ICD10	1	(0.0)	0	(0.0)	0.71	0	(0.0)	0	(0.0)	1
B83_ICD10	2	(0.0)	1	(0.0)	0.26	2	(0.0)	1	(0.0)	0.25
B85_ICD10	0	(0.0)	0	(0.0)	1	2	(0.0)	0	(0.0)	0.6
B86_ICD10	100	(0.2)	14	(0.2)	0.95	70	(0.2)	10	(0.3)	0.88
B89_ICD10	10	(0.0)	2	(0.0)	0.63	5	(0.0)	2	(0.1)	0.18
B90_ICD10	339	(0.8)	33	(0.6)	0.06	223	(0.8)	34	(0.9)	0.52
B91_ICD10	18	(0.0)	1	(0.0)	0.36	9	(0.0)	0	(0.0)	0.27
B94_ICD10	1	(0.0)	0	(0.0)	0.71	0	(0.0)	0	(0.0)	1
B95_ICD10	11	(0.0)	4	(0.1)	0.08	15	(0.1)	4	(0.1)	0.22
B96_ICD10	370	(0.9)	56	(1.0)	0.51	239	(0.8)	28	(0.7)	0.46

B97_ICD10	17	(0.0)	2	(0.0)	0.83	11	(0.0)	2	(0.1)	0.7
C00_ICD10	11	(0.0)	1	(0.0)	0.69	3	(0.0)	0	(0.0)	0.52
C01_ICD10	53	(0.1)	6	(0.1)	0.65	28	(0.1)	6	(0.2)	0.31
C02_ICD10	111	(0.3)	23	(0.4)	0.07	76	(0.3)	9	(0.2)	0.7
C03_ICD10	39	(0.1)	8	(0.1)	0.3	18	(0.1)	3	(0.1)	0.74
C04_ICD10	8	(0.0)	1	(0.0)	0.93	6	(0.0)	1	(0.0)	0.85
C05_ICD10	24	(0.1)	3	(0.1)	0.87	12	(0.0)	1	(0.0)	0.64
C06_ICD10	200	(0.5)	32	(0.5)	0.43	122	(0.4)	16	(0.4)	0.9
C07_ICD10	19	(0.0)	3	(0.1)	0.82	11	(0.0)	1	(0.0)	0.7
C08_ICD10	10	(0.0)	1	(0.0)	0.76	8	(0.0)	0	(0.0)	0.3
C09_ICD10	62	(0.1)	8	(0.1)	0.86	36	(0.1)	4	(0.1)	0.7
C10_ICD10	75	(0.2)	16	(0.3)	0.11	58	(0.2)	9	(0.2)	0.71
C11_ICD10	352	(0.8)	48	(0.8)	0.95	246	(0.9)	30	(0.8)	0.58
C12_ICD10	9	(0.0)	4	(0.1)	0.04	5	(0.0)	0	(0.0)	0.41
C13_ICD10	112	(0.3)	12	(0.2)	0.41	56	(0.2)	6	(0.2)	0.58
C14_ICD10	33	(0.1)	6	(0.1)	0.53	18	(0.1)	2	(0.1)	0.79
C15_ICD10	135	(0.3)	16	(0.3)	0.57	76	(0.3)	8	(0.2)	0.49
C16_ICD10	184	(0.4)	62	(1.1)	<0.001	131	(0.5)	35	(0.9)	<0.001
C17_ICD10	35	(0.1)	11	(0.2)	0.01	16	(0.1)	12	(0.3)	<0.001
C18_ICD10	482	(1.1)	109	(1.9)	<0.001	323	(1.1)	82	(2.1)	<0.001
C19_ICD10	165	(0.4)	35	(0.6)	0.02	102	(0.4)	17	(0.4)	0.43
C20_ICD10	219	(0.5)	63	(1.1)	<0.001	161	(0.6)	31	(0.8)	0.07
C21_ICD10	59	(0.1)	12	(0.2)	0.21	39	(0.1)	2	(0.1)	0.16
C22_ICD10	774	(1.8)	231	(4.0)	<0.001	546	(1.9)	144	(3.8)	<0.001
C23_ICD10	39	(0.1)	23	(0.4)	<0.001	41	(0.1)	20	(0.5)	<0.001
C24_ICD10	119	(0.3)	56	(1.0)	<0.001	81	(0.3)	36	(0.9)	<0.001
C25_ICD10	237	(0.6)	89	(1.5)	<0.001	162	(0.6)	58	(1.5)	<0.001
C26_ICD10	15	(0.0)	4	(0.1)	0.23	11	(0.0)	1	(0.0)	0.7
C30_ICD10	24	(0.1)	3	(0.1)	0.87	20	(0.1)	2	(0.1)	0.68
C31_ICD10	34	(0.1)	1	(0.0)	0.09	25	(0.1)	4	(0.1)	0.76
C32_ICD10	71	(0.2)	8	(0.1)	0.59	46	(0.2)	4	(0.1)	0.39
C33_ICD10	240	(0.6)	35	(0.6)	0.75	164	(0.6)	21	(0.5)	0.8

C34_ICD10	827	(2.0)	99	(1.7)	0.18	540	(1.9)	65	(1.7)	0.36
C37_ICD10	51	(0.1)	2	(0.0)	0.06	11	(0.0)	4	(0.1)	0.08
C38_ICD10	33	(0.1)	2	(0.0)	0.25	18	(0.1)	1	(0.0)	0.37
C39_ICD10	7	(0.0)	0	(0.0)	0.33	5	(0.0)	0	(0.0)	0.41
C40_ICD10	10	(0.0)	2	(0.0)	0.63	6	(0.0)	0	(0.0)	0.37
C41_ICD10	34	(0.1)	5	(0.1)	0.89	32	(0.1)	4	(0.1)	0.88
C43_ICD10	29	(0.1)	5	(0.1)	0.64	14	(0.0)	3	(0.1)	0.47
C44_ICD10	18	(0.0)	3	(0.1)	0.76	14	(0.0)	1	(0.0)	0.53
C46_ICD10	17	(0.0)	5	(0.1)	0.13	14	(0.0)	3	(0.1)	0.47
C47_ICD10	3	(0.0)	2	(0.0)	0.06	6	(0.0)	0	(0.0)	0.37
C48_ICD10	36	(0.1)	4	(0.1)	0.68	24	(0.1)	4	(0.1)	0.7
C49_ICD10	93	(0.2)	20	(0.3)	0.07	63	(0.2)	9	(0.2)	0.89
C4A_ICD10	1	(0.0)	0	(0.0)	0.71	0	(0.0)	0	(0.0)	1
C50_ICD10	731	(1.7)	117	(2.0)	0.13	491	(1.7)	60	(1.6)	0.44
C51_ICD10	10	(0.0)	2	(0.0)	0.63	4	(0.0)	1	(0.0)	0.58
C52_ICD10	8	(0.0)	3	(0.1)	0.12	8	(0.0)	1	(0.0)	0.94
C53_ICD10	154	(0.4)	45	(0.8)	<0.001	100	(0.4)	38	(1.0)	<0.001
C54_ICD10	111	(0.3)	21	(0.4)	0.18	75	(0.3)	17	(0.4)	0.05
C55_ICD10	38	(0.1)	5	(0.1)	0.92	21	(0.1)	6	(0.2)	0.1
C56_ICD10	133	(0.3)	26	(0.4)	0.1	85	(0.3)	18	(0.5)	0.08
C57_ICD10	9	(0.0)	2	(0.0)	0.54	7	(0.0)	1	(0.0)	0.96
C58_ICD10	1	(0.0)	0	(0.0)	0.71	0	(0.0)	0	(0.0)	1
C60_ICD10	1	(0.0)	0	(0.0)	0.71	1	(0.0)	0	(0.0)	0.71
C61_ICD10	338	(0.8)	73	(1.3)	<0.001	204	(0.7)	42	(1.1)	0.01
C62_ICD10	12	(0.0)	1	(0.0)	0.63	1	(0.0)	0	(0.0)	0.71
C63_ICD10	1	(0.0)	0	(0.0)	0.71	0	(0.0)	0	(0.0)	1
C64_ICD10	131	(0.3)	27	(0.5)	0.05	119	(0.4)	22	(0.6)	0.18
C65_ICD10	45	(0.1)	12	(0.2)	0.04	43	(0.2)	10	(0.3)	0.12
C66_ICD10	65	(0.2)	10	(0.2)	0.74	52	(0.2)	9	(0.2)	0.5
C67_ICD10	217	(0.5)	41	(0.7)	0.06	135	(0.5)	42	(1.1)	<0.001
C68_ICD10	28	(0.1)	3	(0.1)	0.68	25	(0.1)	4	(0.1)	0.76
C69_ICD10	19	(0.0)	2	(0.0)	0.72	7	(0.0)	1	(0.0)	0.96

C70_ICD10	4	(0.0)	1	(0.0)	0.59	1	(0.0)	0	(0.0)	0.71
C71_ICD10	75	(0.2)	11	(0.2)	0.84	54	(0.2)	4	(0.1)	0.24
C72_ICD10	9	(0.0)	1	(0.0)	0.84	4	(0.0)	0	(0.0)	0.46
C73_ICD10	112	(0.3)	13	(0.2)	0.56	56	(0.2)	13	(0.3)	0.08
C74_ICD10	5	(0.0)	2	(0.0)	0.18	5	(0.0)	1	(0.0)	0.72
C75_ICD10	11	(0.0)	6	(0.1)	0.003	12	(0.0)	4	(0.1)	0.11
C76_ICD10	17	(0.0)	4	(0.1)	0.33	7	(0.0)	1	(0.0)	0.96
C77_ICD10	18	(0.0)	10	(0.2)	<0.001	11	(0.0)	1	(0.0)	0.7
C78_ICD10	135	(0.3)	29	(0.5)	0.03	82	(0.3)	22	(0.6)	0.004
C79_ICD10	394	(0.9)	62	(1.1)	0.32	239	(0.8)	45	(1.2)	0.04
C7A_ICD10	66	(0.2)	10	(0.2)	0.78	35	(0.1)	11	(0.3)	0.01
C7B_ICD10	2	(0.0)	0	(0.0)	0.6	2	(0.0)	0	(0.0)	0.6
C80_ICD10	3727	(8.8)	583	(10.0)	0.003	2471	(8.7)	420	(11.0)	<0.001
C81_ICD10	42	(0.1)	1	(0.0)	0.05	29	(0.1)	2	(0.1)	0.35
C82_ICD10	243	(0.6)	39	(0.7)	0.37	157	(0.6)	22	(0.6)	0.89
C83_ICD10	126	(0.3)	17	(0.3)	0.94	68	(0.2)	12	(0.3)	0.4
C84_ICD10	30	(0.1)	8	(0.1)	0.09	13	(0.0)	5	(0.1)	0.04
C85_ICD10	621	(1.5)	101	(1.7)	0.12	392	(1.4)	45	(1.2)	0.28
C88_ICD10	9	(0.0)	1	(0.0)	0.84	7	(0.0)	1	(0.0)	0.96
C90_ICD10	181	(0.4)	33	(0.6)	0.14	133	(0.5)	27	(0.7)	0.05
C91_ICD10	205	(0.5)	48	(0.8)	<0.001	146	(0.5)	14	(0.4)	0.21
C92_ICD10	429	(1.0)	60	(1.0)	0.91	307	(1.1)	35	(0.9)	0.33
C93_ICD10	16	(0.0)	1	(0.0)	0.43	8	(0.0)	0	(0.0)	0.3
C94_ICD10	11	(0.0)	0	(0.0)	0.22	9	(0.0)	1	(0.0)	0.85
C95_ICD10	170	(0.4)	37	(0.6)	0.01	119	(0.4)	17	(0.4)	0.84
C96_ICD10	48	(0.1)	9	(0.2)	0.39	30	(0.1)	3	(0.1)	0.61
D00_ICD10	0	(0.0)	1	(0.0)	0.007	1	(0.0)	0	(0.0)	0.71
D01_ICD10	3	(0.0)	0	(0.0)	0.52	0	(0.0)	1	(0.0)	0.007
D02_ICD10	6	(0.0)	0	(0.0)	0.36	2	(0.0)	1	(0.0)	0.25
D04_ICD10	22	(0.1)	5	(0.1)	0.31	20	(0.1)	1	(0.0)	0.31
D05_ICD10	69	(0.2)	9	(0.2)	0.88	38	(0.1)	5	(0.1)	0.95
D06_ICD10	68	(0.2)	12	(0.2)	0.43	63	(0.2)	13	(0.3)	0.17

D07_ICD10	1	(0.0)	0	(0.0)	0.71	1	(0.0)	1	(0.0)	0.1
D09_ICD10	3	(0.0)	0	(0.0)	0.52	0	(0.0)	3	(0.1)	<0.001
D10_ICD10	36	(0.1)	4	(0.1)	0.68	23	(0.1)	3	(0.1)	0.95
D11_ICD10	4	(0.0)	1	(0.0)	0.59	1	(0.0)	1	(0.0)	0.1
D12_ICD10	588	(1.4)	92	(1.6)	0.25	378	(1.3)	55	(1.4)	0.63
D13_ICD10	86	(0.2)	27	(0.5)	<0.001	64	(0.2)	15	(0.4)	0.05
D14_ICD10	28	(0.1)	5	(0.1)	0.59	13	(0.0)	4	(0.1)	0.14
D15_ICD10	10	(0.0)	2	(0.0)	0.63	8	(0.0)	1	(0.0)	0.94
D16_ICD10	13	(0.0)	1	(0.0)	0.57	10	(0.0)	0	(0.0)	0.24
D17_ICD10	290	(0.7)	29	(0.5)	0.1	210	(0.7)	28	(0.7)	0.93
D18_ICD10	166	(0.4)	24	(0.4)	0.82	104	(0.4)	13	(0.3)	0.78
D19_ICD10	1	(0.0)	0	(0.0)	0.71	1	(0.0)	0	(0.0)	0.71
D20_ICD10	0	(0.0)	0	(0.0)	1	1	(0.0)	0	(0.0)	0.71
D21_ICD10	59	(0.1)	10	(0.2)	0.54	44	(0.2)	6	(0.2)	0.99
D22_ICD10	14	(0.0)	3	(0.1)	0.48	7	(0.0)	0	(0.0)	0.33
D23_ICD10	396	(0.9)	36	(0.6)	0.02	216	(0.8)	24	(0.6)	0.35
D24_ICD10	290	(0.7)	33	(0.6)	0.3	210	(0.7)	18	(0.5)	0.06
D25_ICD10	504	(1.2)	55	(0.9)	0.1	314	(1.1)	36	(0.9)	0.34
D26_ICD10	3	(0.0)	0	(0.0)	0.52	1	(0.0)	0	(0.0)	0.71
D27_ICD10	4	(0.0)	0	(0.0)	0.46	3	(0.0)	0	(0.0)	0.52
D28_ICD10	1	(0.0)	0	(0.0)	0.71	0	(0.0)	0	(0.0)	1
D29_ICD10	17	(0.0)	3	(0.1)	0.69	9	(0.0)	0	(0.0)	0.27
D30_ICD10	27	(0.1)	4	(0.1)	0.89	9	(0.0)	5	(0.1)	0.006
D31_ICD10	1	(0.0)	0	(0.0)	0.71	0	(0.0)	0	(0.0)	1
D32_ICD10	62	(0.1)	12	(0.2)	0.28	52	(0.2)	8	(0.2)	0.74
D33_ICD10	23	(0.1)	1	(0.0)	0.23	13	(0.0)	1	(0.0)	0.58
D34_ICD10	2	(0.0)	0	(0.0)	0.6	1	(0.0)	0	(0.0)	0.71
D35_ICD10	35	(0.1)	8	(0.1)	0.19	35	(0.1)	1	(0.0)	0.09
D36_ICD10	7	(0.0)	1	(0.0)	0.97	5	(0.0)	1	(0.0)	0.72
D37_ICD10	26	(0.1)	7	(0.1)	0.11	25	(0.1)	4	(0.1)	0.76
D38_ICD10	29	(0.1)	5	(0.1)	0.64	25	(0.1)	3	(0.1)	0.84
D3A_ICD10	1	(0.0)	1	(0.0)	0.1	1	(0.0)	0	(0.0)	0.71

D40_ICD10	0	(0.0)	1	(0.0)	0.007	0	(0.0)	0	(0.0)	1
D41_ICD10	2	(0.0)	0	(0.0)	0.6	4	(0.0)	1	(0.0)	0.58
D43_ICD10	4	(0.0)	0	(0.0)	0.46	1	(0.0)	0	(0.0)	0.71
D44_ICD10	1	(0.0)	0	(0.0)	0.71	1	(0.0)	0	(0.0)	0.71
D45_ICD10	16	(0.0)	3	(0.1)	0.62	11	(0.0)	0	(0.0)	0.22
D46_ICD10	56	(0.1)	9	(0.2)	0.67	44	(0.2)	5	(0.1)	0.71
D47_ICD10	42	(0.1)	8	(0.1)	0.4	31	(0.1)	0	(0.0)	0.04
D48_ICD10	22	(0.1)	3	(0.1)	0.99	20	(0.1)	6	(0.2)	0.08
D49_ICD10	5352	(12.7)	875	(15.0)	<0.001	3649	(12.9)	569	(14.8)	<0.001
D50_ICD10	601	(1.4)	118	(2.0)	<0.001	410	(1.5)	74	(1.9)	0.02
D51_ICD10	73	(0.2)	12	(0.2)	0.57	49	(0.2)	11	(0.3)	0.13
D52_ICD10	12	(0.0)	4	(0.1)	0.11	14	(0.0)	2	(0.1)	0.95
D53_ICD10	21	(0.0)	6	(0.1)	0.11	10	(0.0)	2	(0.1)	0.61
D55_ICD10	1	(0.0)	0	(0.0)	0.71	5	(0.0)	0	(0.0)	0.41
D56_ICD10	31	(0.1)	8	(0.1)	0.11	21	(0.1)	4	(0.1)	0.53
D58_ICD10	45	(0.1)	7	(0.1)	0.76	25	(0.1)	3	(0.1)	0.84
D59_ICD10	67	(0.2)	11	(0.2)	0.59	50	(0.2)	5	(0.1)	0.51
D60_ICD10	1	(0.0)	0	(0.0)	0.71	1	(0.0)	1	(0.0)	0.1
D61_ICD10	126	(0.3)	34	(0.6)	<0.001	112	(0.4)	15	(0.4)	0.96
D62_ICD10	4	(0.0)	0	(0.0)	0.46	2	(0.0)	0	(0.0)	0.6
D63_ICD10	166	(0.4)	42	(0.7)	<0.001	112	(0.4)	37	(1.0)	<0.001
D64_ICD10	2151	(5.1)	428	(7.4)	<0.001	1387	(4.9)	283	(7.4)	<0.001
D65_ICD10	4	(0.0)	1	(0.0)	0.59	2	(0.0)	0	(0.0)	0.6
D66_ICD10	5	(0.0)	0	(0.0)	0.41	5	(0.0)	1	(0.0)	0.72
D67_ICD10	4	(0.0)	0	(0.0)	0.46	2	(0.0)	3	(0.1)	<0.001
D68_ICD10	147	(0.3)	26	(0.4)	0.24	104	(0.4)	18	(0.5)	0.34
D69_ICD10	299	(0.7)	70	(1.2)	<0.001	213	(0.8)	39	(1.0)	0.08
D70_ICD10	75	(0.2)	15	(0.3)	0.18	45	(0.2)	9	(0.2)	0.28
D71_ICD10	1	(0.0)	0	(0.0)	0.71	1	(0.0)	0	(0.0)	0.71
D72_ICD10	206	(0.5)	28	(0.5)	0.95	131	(0.5)	28	(0.7)	0.03
D73_ICD10	23	(0.1)	2	(0.0)	0.53	20	(0.1)	1	(0.0)	0.31
D75_ICD10	50	(0.1)	8	(0.1)	0.69	30	(0.1)	5	(0.1)	0.67

D76_ICD10	3	(0.0)	0	(0.0)	0.52	1	(0.0)	0	(0.0)	0.71
D78_ICD10	14	(0.0)	3	(0.1)	0.48	6	(0.0)	0	(0.0)	0.37
D80_ICD10	17	(0.0)	3	(0.1)	0.69	10	(0.0)	1	(0.0)	0.77
D82_ICD10	1	(0.0)	1	(0.0)	0.1	0	(0.0)	0	(0.0)	1
D83_ICD10	1	(0.0)	0	(0.0)	0.71	4	(0.0)	0	(0.0)	0.46
D84_ICD10	1	(0.0)	0	(0.0)	0.71	3	(0.0)	0	(0.0)	0.52
D86_ICD10	14	(0.0)	4	(0.1)	0.19	11	(0.0)	2	(0.1)	0.7
D89_ICD10	24	(0.1)	5	(0.1)	0.4	30	(0.1)	2	(0.1)	0.32
E00_ICD10	12	(0.0)	0	(0.0)	0.2	2	(0.0)	0	(0.0)	0.6
E01_ICD10	523	(1.2)	80	(1.4)	0.38	345	(1.2)	33	(0.9)	0.05
E03_ICD10	420	(1.0)	78	(1.3)	0.01	287	(1.0)	51	(1.3)	0.07
E04_ICD10	479	(1.1)	84	(1.4)	0.04	331	(1.2)	44	(1.1)	0.9
E05_ICD10	403	(1.0)	51	(0.9)	0.57	278	(1.0)	30	(0.8)	0.23
E06_ICD10	185	(0.4)	23	(0.4)	0.64	127	(0.4)	19	(0.5)	0.69
E07_ICD10	18	(0.0)	0	(0.0)	0.12	7	(0.0)	1	(0.0)	0.96
E08_ICD10	4525	(10.7)	921	(15.8)	<0.001	3037	(10.8)	647	(16.9)	<0.001
E09_ICD10	3	(0.0)	1	(0.0)	0.43	3	(0.0)	0	(0.0)	0.52
E10_ICD10	50	(0.1)	4	(0.1)	0.29	36	(0.1)	7	(0.2)	0.38
E11_ICD10	4048	(9.6)	835	(14.3)	<0.001	2705	(9.6)	542	(14.1)	<0.001
E13_ICD10	10	(0.0)	1	(0.0)	0.76	3	(0.0)	4	(0.1)	<0.001
E15_ICD10	8	(0.0)	3	(0.1)	0.12	4	(0.0)	2	(0.1)	0.11
E16_ICD10	294	(0.7)	101	(1.7)	<0.001	203	(0.7)	66	(1.7)	<0.001
E20_ICD10	30	(0.1)	3	(0.1)	0.6	17	(0.1)	5	(0.1)	0.12
E21_ICD10	48	(0.1)	7	(0.1)	0.89	25	(0.1)	7	(0.2)	0.08
E22_ICD10	38	(0.1)	6	(0.1)	0.75	37	(0.1)	1	(0.0)	0.08
E23_ICD10	79	(0.2)	13	(0.2)	0.55	57	(0.2)	7	(0.2)	0.8
E24_ICD10	20	(0.0)	4	(0.1)	0.49	21	(0.1)	0	(0.0)	0.09
E25_ICD10	41	(0.1)	6	(0.1)	0.89	29	(0.1)	4	(0.1)	0.98
E26_ICD10	9	(0.0)	4	(0.1)	0.04	4	(0.0)	1	(0.0)	0.58
E27_ICD10	33	(0.1)	5	(0.1)	0.84	16	(0.1)	2	(0.1)	0.91
E28_ICD10	156	(0.4)	7	(0.1)	0.002	92	(0.3)	6	(0.2)	0.07
E29_ICD10	20	(0.0)	1	(0.0)	0.3	2	(0.0)	2	(0.1)	0.02

E30_ICD10	2	(0.0)	0	(0.0)	0.6	1	(0.0)	0	(0.0)	0.71
E31_ICD10	2	(0.0)	0	(0.0)	0.6	0	(0.0)	0	(0.0)	1
E34_ICD10	5	(0.0)	1	(0.0)	0.73	4	(0.0)	0	(0.0)	0.46
E41_ICD10	6	(0.0)	2	(0.0)	0.26	4	(0.0)	0	(0.0)	0.46
E43_ICD10	0	(0.0)	1	(0.0)	0.007	0	(0.0)	0	(0.0)	1
E44_ICD10	4	(0.0)	0	(0.0)	0.46	3	(0.0)	1	(0.0)	0.42
E45_ICD10	2	(0.0)	0	(0.0)	0.6	0	(0.0)	0	(0.0)	1
E46_ICD10	34	(0.1)	7	(0.1)	0.33	27	(0.1)	5	(0.1)	0.52
E50_ICD10	1	(0.0)	0	(0.0)	0.71	0	(0.0)	0	(0.0)	1
E53_ICD10	12	(0.0)	0	(0.0)	0.2	11	(0.0)	1	(0.0)	0.7
E55_ICD10	0	(0.0)	0	(0.0)	1	0	(0.0)	1	(0.0)	0.007
E56_ICD10	2	(0.0)	0	(0.0)	0.6	1	(0.0)	1	(0.0)	0.1
E63_ICD10	1	(0.0)	0	(0.0)	0.71	4	(0.0)	0	(0.0)	0.46
E66_ICD10	246	(0.6)	36	(0.6)	0.73	176	(0.6)	22	(0.6)	0.72
E67_ICD10	1	(0.0)	0	(0.0)	0.71	0	(0.0)	0	(0.0)	1
E70_ICD10	1	(0.0)	0	(0.0)	0.71	1	(0.0)	0	(0.0)	0.71
E72_ICD10	46	(0.1)	14	(0.2)	0.008	31	(0.1)	8	(0.2)	0.1
E74_ICD10	5	(0.0)	0	(0.0)	0.41	2	(0.0)	0	(0.0)	0.6
E75_ICD10	4	(0.0)	0	(0.0)	0.46	0	(0.0)	0	(0.0)	1
E76_ICD10	1	(0.0)	0	(0.0)	0.71	0	(0.0)	0	(0.0)	1
E78_ICD10	3796	(9.0)	701	(12.0)	<0.001	2550	(9.0)	460	(12.0)	<0.001
E80_ICD10	3	(0.0)	1	(0.0)	0.43	3	(0.0)	1	(0.0)	0.42
E83_ICD10	109	(0.3)	23	(0.4)	0.06	88	(0.3)	14	(0.4)	0.58
E84_ICD10	0	(0.0)	0	(0.0)	1	1	(0.0)	0	(0.0)	0.71
E85_ICD10	8	(0.0)	2	(0.0)	0.44	1	(0.0)	2	(0.1)	0.003
E86_ICD10	1	(0.0)	0	(0.0)	0.71	1	(0.0)	3	(0.1)	<0.001
E87_ICD10	609	(1.4)	126	(2.2)	<0.001	393	(1.4)	77	(2.0)	0.003
E88_ICD10	2	(0.0)	0	(0.0)	0.6	1	(0.0)	0	(0.0)	0.71
E89_ICD10	10	(0.0)	0	(0.0)	0.24	4	(0.0)	1	(0.0)	0.58
F01_ICD10	656	(1.6)	94	(1.6)	0.71	413	(1.5)	68	(1.8)	0.14
F02_ICD10	631	(1.5)	92	(1.6)	0.6	417	(1.5)	75	(2.0)	0.02
F03_ICD10	849	(2.0)	145	(2.5)	0.02	592	(2.1)	92	(2.4)	0.22

F04_ICD10	6	(0.0)	3	(0.1)	0.05	8	(0.0)	1	(0.0)	0.94
F05_ICD10	244	(0.6)	42	(0.7)	0.18	151	(0.5)	39	(1.0)	<0.001
F06_ICD10	152	(0.4)	29	(0.5)	0.1	109	(0.4)	19	(0.5)	0.31
F07_ICD10	91	(0.2)	11	(0.2)	0.68	56	(0.2)	11	(0.3)	0.26
F09_ICD10	14	(0.0)	2	(0.0)	0.96	8	(0.0)	1	(0.0)	0.94
F10_ICD10	81	(0.2)	10	(0.2)	0.75	38	(0.1)	9	(0.2)	0.13
F11_ICD10	3	(0.0)	1	(0.0)	0.43	2	(0.0)	0	(0.0)	0.6
F13_ICD10	9	(0.0)	2	(0.0)	0.54	6	(0.0)	1	(0.0)	0.85
F15_ICD10	10	(0.0)	0	(0.0)	0.24	12	(0.0)	0	(0.0)	0.2
F17_ICD10	86	(0.2)	4	(0.1)	0.03	43	(0.2)	3	(0.1)	0.26
F19_ICD10	15	(0.0)	2	(0.0)	0.97	9	(0.0)	0	(0.0)	0.27
F20_ICD10	101	(0.2)	13	(0.2)	0.82	71	(0.3)	7	(0.2)	0.42
F21_ICD10	2	(0.0)	0	(0.0)	0.6	0	(0.0)	0	(0.0)	1
F22_ICD10	23	(0.1)	4	(0.1)	0.67	22	(0.1)	1	(0.0)	0.26
F23_ICD10	32	(0.1)	2	(0.0)	0.27	21	(0.1)	1	(0.0)	0.28
F25_ICD10	12	(0.0)	0	(0.0)	0.2	8	(0.0)	0	(0.0)	0.3
F28_ICD10	1	(0.0)	0	(0.0)	0.71	0	(0.0)	0	(0.0)	1
F29_ICD10	29	(0.1)	4	(0.1)	1	24	(0.1)	3	(0.1)	0.89
F30_ICD10	46	(0.1)	3	(0.1)	0.2	43	(0.2)	5	(0.1)	0.74
F31_ICD10	92	(0.2)	6	(0.1)	0.07	85	(0.3)	11	(0.3)	0.88
F32_ICD10	737	(1.7)	105	(1.8)	0.74	475	(1.7)	82	(2.1)	0.04
F33_ICD10	152	(0.4)	13	(0.2)	0.1	110	(0.4)	18	(0.5)	0.46
F34_ICD10	405	(1.0)	63	(1.1)	0.36	339	(1.2)	38	(1.0)	0.26
F39_ICD10	36	(0.1)	4	(0.1)	0.68	35	(0.1)	4	(0.1)	0.74
F40_ICD10	21	(0.0)	4	(0.1)	0.55	12	(0.0)	1	(0.0)	0.64
F41_ICD10	1109	(2.6)	149	(2.6)	0.78	725	(2.6)	83	(2.2)	0.14
F42_ICD10	16	(0.0)	2	(0.0)	0.9	16	(0.1)	1	(0.0)	0.44
F43_ICD10	449	(1.1)	87	(1.5)	0.003	328	(1.2)	52	(1.4)	0.29
F44_ICD10	7	(0.0)	0	(0.0)	0.33	1	(0.0)	0	(0.0)	0.71
F45_ICD10	211	(0.5)	17	(0.3)	0.03	160	(0.6)	20	(0.5)	0.73
F48_ICD10	103	(0.2)	16	(0.3)	0.65	70	(0.2)	11	(0.3)	0.65
F50_ICD10	9	(0.0)	3	(0.1)	0.17	6	(0.0)	2	(0.1)	0.25

F51_ICD10	2654	(6.3)	420	(7.2)	0.006	1720	(6.1)	274	(7.1)	0.01
F52_ICD10	13	(0.0)	1	(0.0)	0.57	2	(0.0)	0	(0.0)	0.6
F54_ICD10	2	(0.0)	0	(0.0)	0.6	0	(0.0)	0	(0.0)	1
F60_ICD10	23	(0.1)	3	(0.1)	0.93	12	(0.0)	1	(0.0)	0.64
F63_ICD10	5	(0.0)	1	(0.0)	0.73	0	(0.0)	0	(0.0)	1
F64_ICD10	0	(0.0)	0	(0.0)	1	1	(0.0)	0	(0.0)	0.71
F68_ICD10	1	(0.0)	0	(0.0)	0.71	0	(0.0)	0	(0.0)	1
F69_ICD10	2	(0.0)	0	(0.0)	0.6	5	(0.0)	0	(0.0)	0.41
F70_ICD10	1	(0.0)	1	(0.0)	0.1	5	(0.0)	0	(0.0)	0.41
F71_ICD10	1	(0.0)	0	(0.0)	0.71	4	(0.0)	0	(0.0)	0.46
F72_ICD10	2	(0.0)	0	(0.0)	0.6	2	(0.0)	0	(0.0)	0.6
F73_ICD10	1	(0.0)	0	(0.0)	0.71	1	(0.0)	0	(0.0)	0.71
F79_ICD10	30	(0.1)	5	(0.1)	0.69	15	(0.1)	1	(0.0)	0.48
F80_ICD10	9	(0.0)	1	(0.0)	0.84	2	(0.0)	1	(0.0)	0.25
F81_ICD10	0	(0.0)	0	(0.0)	1	1	(0.0)	0	(0.0)	0.71
F84_ICD10	9	(0.0)	1	(0.0)	0.84	9	(0.0)	0	(0.0)	0.27
F88_ICD10	1	(0.0)	0	(0.0)	0.71	1	(0.0)	0	(0.0)	0.71
F90_ICD10	21	(0.0)	1	(0.0)	0.28	7	(0.0)	0	(0.0)	0.33
F91_ICD10	6	(0.0)	0	(0.0)	0.36	2	(0.0)	0	(0.0)	0.6
F93_ICD10	13	(0.0)	3	(0.1)	0.41	7	(0.0)	0	(0.0)	0.33
F95_ICD10	1	(0.0)	0	(0.0)	0.71	3	(0.0)	0	(0.0)	0.52
F98_ICD10	16	(0.0)	1	(0.0)	0.43	7	(0.0)	0	(0.0)	0.33
G00_ICD10	17	(0.0)	3	(0.1)	0.69	3	(0.0)	1	(0.0)	0.42
G02_ICD10	0	(0.0)	0	(0.0)	1	1	(0.0)	0	(0.0)	0.71
G03_ICD10	61	(0.1)	8	(0.1)	0.9	50	(0.2)	3	(0.1)	0.16
G04_ICD10	53	(0.1)	11	(0.2)	0.21	37	(0.1)	4	(0.1)	0.67
G05_ICD10	2	(0.0)	0	(0.0)	0.6	0	(0.0)	0	(0.0)	1
G06_ICD10	27	(0.1)	3	(0.1)	0.72	11	(0.0)	3	(0.1)	0.27
G08_ICD10	11	(0.0)	2	(0.0)	0.72	5	(0.0)	0	(0.0)	0.41
G09_ICD10	0	(0.0)	0	(0.0)	1	1	(0.0)	0	(0.0)	0.71
G10_ICD10	4	(0.0)	1	(0.0)	0.59	1	(0.0)	0	(0.0)	0.71
G11_ICD10	25	(0.1)	8	(0.1)	0.03	23	(0.1)	4	(0.1)	0.65

G12_ICD10	28	(0.1)	1	(0.0)	0.15	23	(0.1)	0	(0.0)	0.08
G14_ICD10	1	(0.0)	0	(0.0)	0.71	1	(0.0)	0	(0.0)	0.71
G20_ICD10	829	(2.0)	124	(2.1)	0.38	585	(2.1)	76	(2.0)	0.72
G21_ICD10	224	(0.5)	42	(0.7)	0.06	158	(0.6)	19	(0.5)	0.62
G23_ICD10	76	(0.2)	7	(0.1)	0.31	41	(0.1)	6	(0.2)	0.86
G24_ICD10	91	(0.2)	9	(0.2)	0.34	52	(0.2)	11	(0.3)	0.18
G25_ICD10	207	(0.5)	40	(0.7)	0.05	169	(0.6)	22	(0.6)	0.85
G30_ICD10	64	(0.2)	14	(0.2)	0.11	54	(0.2)	5	(0.1)	0.41
G31_ICD10	76	(0.2)	21	(0.4)	0.004	60	(0.2)	14	(0.4)	0.06
G32_ICD10	29	(0.1)	7	(0.1)	0.18	15	(0.1)	1	(0.0)	0.48
G35_ICD10	36	(0.1)	9	(0.2)	0.1	25	(0.1)	2	(0.1)	0.47
G36_ICD10	6	(0.0)	2	(0.0)	0.26	4	(0.0)	1	(0.0)	0.58
G37_ICD10	9	(0.0)	2	(0.0)	0.54	3	(0.0)	0	(0.0)	0.52
G40_ICD10	372	(0.9)	45	(0.8)	0.41	228	(0.8)	28	(0.7)	0.62
G43_ICD10	149	(0.4)	14	(0.2)	0.17	106	(0.4)	11	(0.3)	0.39
G44_ICD10	1112	(2.6)	153	(2.6)	1	686	(2.4)	99	(2.6)	0.56
G45_ICD10	364	(0.9)	58	(1.0)	0.3	269	(1.0)	41	(1.1)	0.49
G47_ICD10	1481	(3.5)	246	(4.2)	0.005	975	(3.5)	143	(3.7)	0.38
G50_ICD10	78	(0.2)	10	(0.2)	0.83	57	(0.2)	6	(0.2)	0.55
G51_ICD10	159	(0.4)	32	(0.5)	0.05	102	(0.4)	15	(0.4)	0.77
G52_ICD10	16	(0.0)	1	(0.0)	0.43	10	(0.0)	1	(0.0)	0.77
G54_ICD10	156	(0.4)	28	(0.5)	0.19	104	(0.4)	15	(0.4)	0.82
G56_ICD10	386	(0.9)	58	(1.0)	0.53	234	(0.8)	27	(0.7)	0.42
G57_ICD10	48	(0.1)	5	(0.1)	0.55	14	(0.0)	1	(0.0)	0.53
G58_ICD10	313	(0.7)	53	(0.9)	0.16	205	(0.7)	33	(0.9)	0.36
G60_ICD10	315	(0.7)	70	(1.2)	<0.001	213	(0.8)	31	(0.8)	0.71
G61_ICD10	15	(0.0)	3	(0.1)	0.55	15	(0.1)	2	(0.1)	0.98
G62_ICD10	14	(0.0)	1	(0.0)	0.52	13	(0.0)	2	(0.1)	0.87
G63_ICD10	24	(0.1)	8	(0.1)	0.03	15	(0.1)	4	(0.1)	0.22
G70_ICD10	6	(0.0)	1	(0.0)	0.86	7	(0.0)	2	(0.1)	0.34
G71_ICD10	8	(0.0)	0	(0.0)	0.29	6	(0.0)	0	(0.0)	0.37
G72_ICD10	46	(0.1)	5	(0.1)	0.62	25	(0.1)	3	(0.1)	0.84

G73_ICD10	4	(0.0)	0	(0.0)	0.46	5	(0.0)	0	(0.0)	0.41
G80_ICD10	43	(0.1)	2	(0.0)	0.12	24	(0.1)	1	(0.0)	0.22
G81_ICD10	53	(0.1)	15	(0.3)	0.01	38	(0.1)	3	(0.1)	0.36
G82_ICD10	14	(0.0)	5	(0.1)	0.06	16	(0.1)	1	(0.0)	0.44
G83_ICD10	66	(0.2)	7	(0.1)	0.51	57	(0.2)	3	(0.1)	0.1
G89_ICD10	4	(0.0)	0	(0.0)	0.46	3	(0.0)	0	(0.0)	0.52
G90_ICD10	21	(0.0)	0	(0.0)	0.09	16	(0.1)	0	(0.0)	0.14
G91_ICD10	187	(0.4)	22	(0.4)	0.48	125	(0.4)	9	(0.2)	0.06
G93_ICD10	415	(1.0)	84	(1.4)	0.001	258	(0.9)	52	(1.4)	0.008
G95_ICD10	50	(0.1)	11	(0.2)	0.16	36	(0.1)	6	(0.2)	0.64
G96_ICD10	5	(0.0)	2	(0.0)	0.18	3	(0.0)	0	(0.0)	0.52
G97_ICD10	0	(0.0)	0	(0.0)	1	1	(0.0)	0	(0.0)	0.71
G99_ICD10	32	(0.1)	3	(0.1)	0.52	24	(0.1)	5	(0.1)	0.38
H00_ICD10	124	(0.3)	13	(0.2)	0.35	97	(0.3)	8	(0.2)	0.17
H01_ICD10	44	(0.1)	9	(0.2)	0.28	37	(0.1)	6	(0.2)	0.68
H02_ICD10	471	(1.1)	71	(1.2)	0.47	292	(1.0)	43	(1.1)	0.61
H04_ICD10	1387	(3.3)	205	(3.5)	0.33	900	(3.2)	146	(3.8)	0.04
H05_ICD10	24	(0.1)	4	(0.1)	0.72	12	(0.0)	2	(0.1)	0.79
H10_ICD10	3530	(8.3)	573	(9.8)	<0.001	2371	(8.4)	401	(10.5)	<0.001
H11_ICD10	340	(0.8)	38	(0.7)	0.22	251	(0.9)	31	(0.8)	0.62
H15_ICD10	24	(0.1)	3	(0.1)	0.87	13	(0.0)	0	(0.0)	0.18
H16_ICD10	139	(0.3)	24	(0.4)	0.3	103	(0.4)	8	(0.2)	0.12
H17_ICD10	82	(0.2)	21	(0.4)	0.01	56	(0.2)	5	(0.1)	0.37
H18_ICD10	478	(1.1)	74	(1.3)	0.34	316	(1.1)	60	(1.6)	0.02
H20_ICD10	31	(0.1)	5	(0.1)	0.74	19	(0.1)	2	(0.1)	0.73
H21_ICD10	6	(0.0)	2	(0.0)	0.26	6	(0.0)	0	(0.0)	0.37
H25_ICD10	642	(1.5)	126	(2.2)	<0.001	408	(1.4)	84	(2.2)	<0.001
H26_ICD10	2581	(6.1)	470	(8.1)	<0.001	1715	(6.1)	322	(8.4)	<0.001
H27_ICD10	27	(0.1)	6	(0.1)	0.28	17	(0.1)	2	(0.1)	0.85
H28_ICD10	0	(0.0)	0	(0.0)	1	1	(0.0)	0	(0.0)	0.71
H30_ICD10	12	(0.0)	1	(0.0)	0.63	9	(0.0)	2	(0.1)	0.52
H31_ICD10	12	(0.0)	2	(0.0)	0.8	7	(0.0)	3	(0.1)	0.08

H33_ICD10	161	(0.4)	25	(0.4)	0.57	111	(0.4)	11	(0.3)	0.32
H34_ICD10	95	(0.2)	24	(0.4)	0.007	69	(0.2)	13	(0.3)	0.27
H35_ICD10	1012	(2.4)	182	(3.1)	<0.001	662	(2.3)	136	(3.5)	<0.001
H36_ICD10	1	(0.0)	0	(0.0)	0.71	1	(0.0)	0	(0.0)	0.71
H40_ICD10	988	(2.3)	155	(2.7)	0.13	659	(2.3)	131	(3.4)	<0.001
H43_ICD10	532	(1.3)	72	(1.2)	0.89	322	(1.1)	49	(1.3)	0.45
H44_ICD10	134	(0.3)	20	(0.3)	0.73	88	(0.3)	10	(0.3)	0.59
H46_ICD10	31	(0.1)	3	(0.1)	0.56	14	(0.0)	0	(0.0)	0.17
H47_ICD10	185	(0.4)	35	(0.6)	0.08	103	(0.4)	21	(0.5)	0.09
H49_ICD10	54	(0.1)	12	(0.2)	0.13	39	(0.1)	7	(0.2)	0.49
H50_ICD10	80	(0.2)	19	(0.3)	0.03	35	(0.1)	10	(0.3)	0.03
H51_ICD10	10	(0.0)	0	(0.0)	0.24	3	(0.0)	3	(0.1)	0.004
H52_ICD10	727	(1.7)	83	(1.4)	0.1	463	(1.6)	56	(1.5)	0.41
H53_ICD10	1714	(4.1)	244	(4.2)	0.62	1041	(3.7)	194	(5.1)	<0.001
H54_ICD10	10	(0.0)	0	(0.0)	0.24	4	(0.0)	0	(0.0)	0.46
H55_ICD10	5	(0.0)	0	(0.0)	0.41	3	(0.0)	1	(0.0)	0.42
H57_ICD10	15	(0.0)	1	(0.0)	0.47	7	(0.0)	0	(0.0)	0.33
H60_ICD10	369	(0.9)	48	(0.8)	0.71	248	(0.9)	36	(0.9)	0.7
H61_ICD10	287	(0.7)	48	(0.8)	0.21	224	(0.8)	37	(1.0)	0.27
H62_ICD10	8	(0.0)	0	(0.0)	0.29	1	(0.0)	1	(0.0)	0.1
H65_ICD10	224	(0.5)	22	(0.4)	0.13	147	(0.5)	27	(0.7)	0.15
H66_ICD10	389	(0.9)	44	(0.8)	0.21	231	(0.8)	27	(0.7)	0.46
H69_ICD10	65	(0.2)	4	(0.1)	0.11	47	(0.2)	5	(0.1)	0.6
H70_ICD10	13	(0.0)	0	(0.0)	0.18	5	(0.0)	0	(0.0)	0.41
H71_ICD10	9	(0.0)	5	(0.1)	0.007	16	(0.1)	0	(0.0)	0.14
H72_ICD10	2	(0.0)	0	(0.0)	0.6	0	(0.0)	0	(0.0)	1
H73_ICD10	8	(0.0)	0	(0.0)	0.29	4	(0.0)	1	(0.0)	0.58
H80_ICD10	7	(0.0)	4	(0.1)	0.01	4	(0.0)	0	(0.0)	0.46
H81_ICD10	302	(0.7)	51	(0.9)	0.17	216	(0.8)	28	(0.7)	0.82
H83_ICD10	1309	(3.1)	206	(3.5)	0.07	912	(3.2)	110	(2.9)	0.24
H90_ICD10	60	(0.1)	14	(0.2)	0.07	37	(0.1)	9	(0.2)	0.11
H91_ICD10	783	(1.9)	134	(2.3)	0.02	482	(1.7)	95	(2.5)	<0.001

H92_ICD10	14	(0.0)	3	(0.1)	0.48	12	(0.0)	3	(0.1)	0.34
H93_ICD10	448	(1.1)	51	(0.9)	0.2	287	(1.0)	39	(1.0)	0.99
H95_ICD10	1	(0.0)	0	(0.0)	0.71	0	(0.0)	0	(0.0)	1
I00_ICD10	2	(0.0)	0	(0.0)	0.6	0	(0.0)	0	(0.0)	1
I02_ICD10	1	(0.0)	0	(0.0)	0.71	0	(0.0)	0	(0.0)	1
I05_ICD10	105	(0.2)	14	(0.2)	0.91	63	(0.2)	11	(0.3)	0.44
I06_ICD10	43	(0.1)	5	(0.1)	0.72	31	(0.1)	9	(0.2)	0.04
I07_ICD10	25	(0.1)	3	(0.1)	0.82	14	(0.0)	1	(0.0)	0.53
I08_ICD10	32	(0.1)	5	(0.1)	0.79	13	(0.0)	3	(0.1)	0.4
I09_ICD10	57	(0.1)	10	(0.2)	0.48	37	(0.1)	9	(0.2)	0.11
I10_ICD10	7104	(16.8)	1304	(22.4)	<0.001	4711	(16.7)	873	(22.8)	<0.001
I11_ICD10	502	(1.2)	90	(1.5)	0.02	389	(1.4)	61	(1.6)	0.29
I12_ICD10	31	(0.1)	8	(0.1)	0.11	21	(0.1)	5	(0.1)	0.25
I13_ICD10	9	(0.0)	7	(0.1)	<0.001	7	(0.0)	1	(0.0)	0.96
I15_ICD10	34	(0.1)	4	(0.1)	0.77	21	(0.1)	4	(0.1)	0.53
I20_ICD10	290	(0.7)	57	(1.0)	0.01	191	(0.7)	23	(0.6)	0.59
I21_ICD10	361	(0.9)	65	(1.1)	0.04	206	(0.7)	35	(0.9)	0.22
I24_ICD10	1384	(3.3)	202	(3.5)	0.43	912	(3.2)	150	(3.9)	0.03
I25_ICD10	2898	(6.9)	498	(8.6)	<0.001	1910	(6.8)	329	(8.6)	<0.001
I26_ICD10	88	(0.2)	15	(0.3)	0.44	55	(0.2)	7	(0.2)	0.87
I27_ICD10	115	(0.3)	13	(0.2)	0.5	63	(0.2)	12	(0.3)	0.28
I28_ICD10	3	(0.0)	0	(0.0)	0.52	2	(0.0)	0	(0.0)	0.6
I30_ICD10	5	(0.0)	1	(0.0)	0.73	2	(0.0)	0	(0.0)	0.6
I31_ICD10	28	(0.1)	6	(0.1)	0.32	23	(0.1)	2	(0.1)	0.54
I32_ICD10	2	(0.0)	0	(0.0)	0.6	2	(0.0)	0	(0.0)	0.6
I33_ICD10	83	(0.2)	14	(0.2)	0.48	32	(0.1)	15	(0.4)	<0.001
I34_ICD10	435	(1.0)	55	(0.9)	0.55	313	(1.1)	35	(0.9)	0.27
I35_ICD10	187	(0.4)	29	(0.5)	0.55	111	(0.4)	29	(0.8)	0.001
I36_ICD10	6	(0.0)	0	(0.0)	0.36	5	(0.0)	0	(0.0)	0.41
I37_ICD10	1	(0.0)	1	(0.0)	0.1	1	(0.0)	0	(0.0)	0.71
I38_ICD10	108	(0.3)	27	(0.5)	0.005	78	(0.3)	15	(0.4)	0.21
I39_ICD10	2	(0.0)	1	(0.0)	0.26	5	(0.0)	1	(0.0)	0.72

I40_ICD10	3	(0.0)	0	(0.0)	0.52	2	(0.0)	1	(0.0)	0.25
I42_ICD10	75	(0.2)	8	(0.1)	0.49	27	(0.1)	5	(0.1)	0.52
I43_ICD10	5	(0.0)	0	(0.0)	0.41	4	(0.0)	0	(0.0)	0.46
I44_ICD10	86	(0.2)	16	(0.3)	0.27	57	(0.2)	10	(0.3)	0.45
I45_ICD10	23	(0.1)	4	(0.1)	0.67	11	(0.0)	3	(0.1)	0.27
I46_ICD10	50	(0.1)	9	(0.2)	0.46	28	(0.1)	1	(0.0)	0.16
I47_ICD10	196	(0.5)	38	(0.7)	0.05	135	(0.5)	17	(0.4)	0.77
I48_ICD10	888	(2.1)	156	(2.7)	0.004	561	(2.0)	112	(2.9)	<0.001
I49_ICD10	1631	(3.9)	245	(4.2)	0.19	1125	(4.0)	170	(4.4)	0.18
I50_ICD10	1502	(3.6)	303	(5.2)	<0.001	1055	(3.7)	184	(4.8)	0.001
I51_ICD10	1913	(4.5)	318	(5.5)	0.001	1278	(4.5)	198	(5.2)	0.08
I60_ICD10	75	(0.2)	11	(0.2)	0.84	41	(0.1)	3	(0.1)	0.29
I61_ICD10	301	(0.7)	38	(0.7)	0.61	187	(0.7)	24	(0.6)	0.8
I62_ICD10	250	(0.6)	44	(0.8)	0.13	141	(0.5)	27	(0.7)	0.1
I63_ICD10	1738	(4.1)	296	(5.1)	<0.001	1182	(4.2)	176	(4.6)	0.24
I65_ICD10	165	(0.4)	33	(0.6)	0.05	107	(0.4)	14	(0.4)	0.9
I66_ICD10	124	(0.3)	37	(0.6)	<0.001	95	(0.3)	13	(0.3)	0.98
I67_ICD10	2600	(6.1)	451	(7.7)	<0.001	1765	(6.2)	295	(7.7)	<0.001
I69_ICD10	835	(2.0)	158	(2.7)	<0.001	538	(1.9)	88	(2.3)	0.1
I70_ICD10	172	(0.4)	41	(0.7)	0.001	121	(0.4)	19	(0.5)	0.55
I71_ICD10	173	(0.4)	44	(0.8)	<0.001	118	(0.4)	23	(0.6)	0.11
I72_ICD10	54	(0.1)	9	(0.2)	0.59	36	(0.1)	10	(0.3)	0.04
I73_ICD10	607	(1.4)	109	(1.9)	0.01	412	(1.5)	89	(2.3)	<0.001
I74_ICD10	466	(1.1)	85	(1.5)	0.02	319	(1.1)	69	(1.8)	<0.001
I77_ICD10	245	(0.6)	41	(0.7)	0.24	200	(0.7)	27	(0.7)	0.98
I78_ICD10	4	(0.0)	0	(0.0)	0.46	1	(0.0)	0	(0.0)	0.71
I79_ICD10	10	(0.0)	2	(0.0)	0.63	7	(0.0)	0	(0.0)	0.33
I80_ICD10	210	(0.5)	58	(1.0)	<0.001	160	(0.6)	22	(0.6)	0.95
I81_ICD10	5	(0.0)	1	(0.0)	0.73	3	(0.0)	1	(0.0)	0.42
I82_ICD10	31	(0.1)	5	(0.1)	0.74	27	(0.1)	2	(0.1)	0.4
I83_ICD10	312	(0.7)	62	(1.1)	0.008	206	(0.7)	45	(1.2)	0.003
I85_ICD10	165	(0.4)	57	(1.0)	<0.001	104	(0.4)	55	(1.4)	<0.001

I86_ICD10	55	(0.1)	15	(0.3)	0.02	41	(0.1)	20	(0.5)	<0.001
I87_ICD10	12	(0.0)	1	(0.0)	0.63	6	(0.0)	1	(0.0)	0.85
I88_ICD10	82	(0.2)	4	(0.1)	0.03	60	(0.2)	4	(0.1)	0.16
I89_ICD10	77	(0.2)	12	(0.2)	0.69	51	(0.2)	12	(0.3)	0.08
I95_ICD10	140	(0.3)	25	(0.4)	0.23	89	(0.3)	14	(0.4)	0.61
I96_ICD10	34	(0.1)	6	(0.1)	0.57	20	(0.1)	2	(0.1)	0.68
I97_ICD10	75	(0.2)	8	(0.1)	0.49	38	(0.1)	9	(0.2)	0.13
I99_ICD10	1	(0.0)	0	(0.0)	0.71	0	(0.0)	0	(0.0)	1
J00_ICD10	816	(1.9)	103	(1.8)	0.4	560	(2.0)	68	(1.8)	0.38
J01_ICD10	688	(1.6)	64	(1.1)	0.002	426	(1.5)	37	(1.0)	0.008
J02_ICD10	754	(1.8)	90	(1.5)	0.2	528	(1.9)	67	(1.7)	0.6
J03_ICD10	355	(0.8)	31	(0.5)	0.01	236	(0.8)	23	(0.6)	0.13
J04_ICD10	15	(0.0)	3	(0.1)	0.55	9	(0.0)	2	(0.1)	0.52
J05_ICD10	23	(0.1)	1	(0.0)	0.23	13	(0.0)	2	(0.1)	0.87
J06_ICD10	4983	(11.8)	688	(11.8)	0.94	3183	(11.3)	497	(13.0)	0.002
J09_ICD10	4	(0.0)	0	(0.0)	0.46	2	(0.0)	1	(0.0)	0.25
J10_ICD10	291	(0.7)	19	(0.3)	0.001	177	(0.6)	17	(0.4)	0.17
J11_ICD10	239	(0.6)	24	(0.4)	0.14	155	(0.5)	18	(0.5)	0.53
J12_ICD10	2	(0.0)	2	(0.0)	0.02	4	(0.0)	0	(0.0)	0.46
J13_ICD10	29	(0.1)	4	(0.1)	1	14	(0.0)	2	(0.1)	0.95
J14_ICD10	0	(0.0)	0	(0.0)	1	2	(0.0)	0	(0.0)	0.6
J15_ICD10	397	(0.9)	35	(0.6)	0.01	233	(0.8)	32	(0.8)	0.95
J16_ICD10	8	(0.0)	1	(0.0)	0.93	11	(0.0)	4	(0.1)	0.08
J17_ICD10	5	(0.0)	1	(0.0)	0.73	8	(0.0)	0	(0.0)	0.3
J18_ICD10	3923	(9.3)	546	(9.4)	0.8	2565	(9.1)	389	(10.1)	0.03
J20_ICD10	1005	(2.4)	127	(2.2)	0.36	661	(2.3)	78	(2.0)	0.24
J21_ICD10	158	(0.4)	19	(0.3)	0.58	120	(0.4)	9	(0.2)	0.08
J30_ICD10	2239	(5.3)	273	(4.7)	0.05	1455	(5.2)	155	(4.0)	0.003
J31_ICD10	659	(1.6)	83	(1.4)	0.44	438	(1.6)	52	(1.4)	0.36
J32_ICD10	538	(1.3)	73	(1.3)	0.91	384	(1.4)	35	(0.9)	0.02
J33_ICD10	27	(0.1)	2	(0.0)	0.39	20	(0.1)	1	(0.0)	0.31
J34_ICD10	83	(0.2)	10	(0.2)	0.69	50	(0.2)	5	(0.1)	0.51

J35_ICD10	49	(0.1)	3	(0.1)	0.16	32	(0.1)	0	(0.0)	0.04
J36_ICD10	21	(0.0)	1	(0.0)	0.28	11	(0.0)	2	(0.1)	0.7
J37_ICD10	31	(0.1)	7	(0.1)	0.23	13	(0.0)	3	(0.1)	0.4
J38_ICD10	439	(1.0)	72	(1.2)	0.17	301	(1.1)	31	(0.8)	0.14
J39_ICD10	97	(0.2)	11	(0.2)	0.54	64	(0.2)	4	(0.1)	0.12
J40_ICD10	1780	(4.2)	214	(3.7)	0.06	1126	(4.0)	146	(3.8)	0.6
J41_ICD10	7	(0.0)	2	(0.0)	0.35	4	(0.0)	0	(0.0)	0.46
J42_ICD10	382	(0.9)	63	(1.1)	0.18	288	(1.0)	41	(1.1)	0.77
J43_ICD10	71	(0.2)	3	(0.1)	0.03	44	(0.2)	5	(0.1)	0.71
J44_ICD10	1773	(4.2)	180	(3.1)	<0.001	1133	(4.0)	115	(3.0)	0.002
J45_ICD10	1047	(2.5)	120	(2.1)	0.05	646	(2.3)	75	(2.0)	0.2
J47_ICD10	493	(1.2)	37	(0.6)	<0.001	281	(1.0)	32	(0.8)	0.34
J60_ICD10	26	(0.1)	3	(0.1)	0.77	12	(0.0)	0	(0.0)	0.2
J62_ICD10	2	(0.0)	0	(0.0)	0.6	1	(0.0)	0	(0.0)	0.71
J63_ICD10	0	(0.0)	0	(0.0)	1	2	(0.0)	0	(0.0)	0.6
J64_ICD10	30	(0.1)	2	(0.0)	0.31	13	(0.0)	1	(0.0)	0.58
J67_ICD10	5	(0.0)	0	(0.0)	0.41	0	(0.0)	0	(0.0)	1
J68_ICD10	3	(0.0)	1	(0.0)	0.43	2	(0.0)	0	(0.0)	0.6
J69_ICD10	384	(0.9)	38	(0.7)	0.05	234	(0.8)	20	(0.5)	0.04
J70_ICD10	4	(0.0)	0	(0.0)	0.46	0	(0.0)	0	(0.0)	1
J80_ICD10	16	(0.0)	3	(0.1)	0.62	10	(0.0)	2	(0.1)	0.61
J81_ICD10	20	(0.0)	8	(0.1)	0.008	22	(0.1)	5	(0.1)	0.29
J82_ICD10	2	(0.0)	0	(0.0)	0.6	0	(0.0)	0	(0.0)	1
J84_ICD10	218	(0.5)	29	(0.5)	0.86	147	(0.5)	12	(0.3)	0.09
J85_ICD10	59	(0.1)	7	(0.1)	0.71	48	(0.2)	1	(0.0)	0.03
J86_ICD10	91	(0.2)	13	(0.2)	0.9	59	(0.2)	12	(0.3)	0.2
J90_ICD10	68	(0.2)	13	(0.2)	0.28	39	(0.1)	11	(0.3)	0.03
J91_ICD10	306	(0.7)	45	(0.8)	0.68	235	(0.8)	37	(1.0)	0.4
J93_ICD10	20	(0.0)	1	(0.0)	0.3	14	(0.0)	1	(0.0)	0.53
J94_ICD10	1	(0.0)	1	(0.0)	0.1	2	(0.0)	0	(0.0)	0.6
J95_ICD10	46	(0.1)	4	(0.1)	0.37	25	(0.1)	1	(0.0)	0.2
J96_ICD10	326	(0.8)	56	(1.0)	0.12	216	(0.8)	32	(0.8)	0.64

J98_ICD10	82	(0.2)	8	(0.1)	0.35	57	(0.2)	4	(0.1)	0.19
J99_ICD10	0	(0.0)	0	(0.0)	1	2	(0.0)	0	(0.0)	0.6
K00_ICD10	276	(0.7)	21	(0.4)	0.008	163	(0.6)	11	(0.3)	0.02
K01_ICD10	26	(0.1)	3	(0.1)	0.77	18	(0.1)	2	(0.1)	0.79
K02_ICD10	219	(0.5)	30	(0.5)	0.98	113	(0.4)	15	(0.4)	0.94
K03_ICD10	59	(0.1)	8	(0.1)	0.97	30	(0.1)	6	(0.2)	0.38
K04_ICD10	1100	(2.6)	141	(2.4)	0.42	726	(2.6)	112	(2.9)	0.2
K05_ICD10	368	(0.9)	62	(1.1)	0.14	216	(0.8)	33	(0.9)	0.52
K06_ICD10	0	(0.0)	0	(0.0)	1	1	(0.0)	0	(0.0)	0.71
K08_ICD10	983	(2.3)	141	(2.4)	0.64	672	(2.4)	127	(3.3)	<0.001
K09_ICD10	8	(0.0)	2	(0.0)	0.44	8	(0.0)	1	(0.0)	0.94
K11_ICD10	270	(0.6)	38	(0.7)	0.9	148	(0.5)	27	(0.7)	0.15
K12_ICD10	500	(1.2)	68	(1.2)	0.93	350	(1.2)	60	(1.6)	0.09
K13_ICD10	1120	(2.6)	164	(2.8)	0.45	734	(2.6)	106	(2.8)	0.54
K14_ICD10	166	(0.4)	30	(0.5)	0.17	97	(0.3)	16	(0.4)	0.47
K20_ICD10	184	(0.4)	25	(0.4)	0.95	121	(0.4)	17	(0.4)	0.89
K21_ICD10	2700	(6.4)	434	(7.5)	0.002	1796	(6.4)	292	(7.6)	0.003
K22_ICD10	46	(0.1)	12	(0.2)	0.04	30	(0.1)	8	(0.2)	0.08
K25_ICD10	1210	(2.9)	241	(4.1)	<0.001	774	(2.7)	153	(4.0)	<0.001
K26_ICD10	609	(1.4)	127	(2.2)	<0.001	431	(1.5)	88	(2.3)	<0.001
K27_ICD10	1116	(2.6)	204	(3.5)	<0.001	675	(2.4)	138	(3.6)	<0.001
K28_ICD10	6	(0.0)	3	(0.1)	0.05	0	(0.0)	1	(0.0)	0.007
K29_ICD10	3604	(8.5)	649	(11.1)	<0.001	2398	(8.5)	415	(10.8)	<0.001
K30_ICD10	711	(1.7)	133	(2.3)	0.001	461	(1.6)	104	(2.7)	<0.001
K31_ICD10	2132	(5.0)	375	(6.4)	<0.001	1357	(4.8)	231	(6.0)	0.001
K35_ICD10	164	(0.4)	17	(0.3)	0.26	70	(0.2)	12	(0.3)	0.45
K36_ICD10	11	(0.0)	0	(0.0)	0.22	0	(0.0)	1	(0.0)	0.007
K37_ICD10	68	(0.2)	7	(0.1)	0.46	37	(0.1)	5	(0.1)	0.99
K38_ICD10	0	(0.0)	0	(0.0)	1	1	(0.0)	0	(0.0)	0.71
K40_ICD10	321	(0.8)	63	(1.1)	0.009	220	(0.8)	43	(1.1)	0.03
K41_ICD10	2	(0.0)	1	(0.0)	0.26	1	(0.0)	0	(0.0)	0.71
K42_ICD10	12	(0.0)	6	(0.1)	0.006	12	(0.0)	2	(0.1)	0.79

K43_ICD10	63	(0.1)	15	(0.3)	0.05	47	(0.2)	10	(0.3)	0.19
K44_ICD10	38	(0.1)	4	(0.1)	0.61	27	(0.1)	4	(0.1)	0.87
K45_ICD10	7	(0.0)	1	(0.0)	0.97	5	(0.0)	2	(0.1)	0.18
K46_ICD10	117	(0.3)	27	(0.5)	0.01	76	(0.3)	13	(0.3)	0.44
K50_ICD10	45	(0.1)	8	(0.1)	0.5	37	(0.1)	4	(0.1)	0.67
K51_ICD10	58	(0.1)	10	(0.2)	0.51	41	(0.1)	7	(0.2)	0.57
K52_ICD10	1770	(4.2)	287	(4.9)	0.008	1111	(3.9)	143	(3.7)	0.54
K55_ICD10	28	(0.1)	4	(0.1)	0.94	22	(0.1)	0	(0.0)	0.08
K56_ICD10	542	(1.3)	100	(1.7)	0.007	344	(1.2)	68	(1.8)	0.004
K57_ICD10	106	(0.3)	25	(0.4)	0.01	74	(0.3)	7	(0.2)	0.36
K58_ICD10	289	(0.7)	44	(0.8)	0.53	190	(0.7)	18	(0.5)	0.14
K59_ICD10	672	(1.6)	121	(2.1)	0.006	415	(1.5)	105	(2.7)	<0.001
K60_ICD10	92	(0.2)	22	(0.4)	0.02	69	(0.2)	10	(0.3)	0.85
K61_ICD10	102	(0.2)	13	(0.2)	0.79	83	(0.3)	15	(0.4)	0.3
K62_ICD10	136	(0.3)	16	(0.3)	0.55	92	(0.3)	15	(0.4)	0.51
K63_ICD10	383	(0.9)	63	(1.1)	0.19	244	(0.9)	49	(1.3)	0.01
K64_ICD10	1588	(3.8)	261	(4.5)	0.007	1073	(3.8)	149	(3.9)	0.79
K65_ICD10	93	(0.2)	33	(0.6)	<0.001	86	(0.3)	13	(0.3)	0.72
K66_ICD10	7	(0.0)	1	(0.0)	0.97	0	(0.0)	1	(0.0)	0.007
K67_ICD10	22	(0.1)	8	(0.1)	0.01	12	(0.0)	3	(0.1)	0.34
K68_ICD10	115	(0.3)	13	(0.2)	0.5	67	(0.2)	8	(0.2)	0.73
K70_ICD10	124	(0.3)	29	(0.5)	0.009	67	(0.2)	21	(0.5)	<0.001
K71_ICD10	187	(0.4)	21	(0.4)	0.37	134	(0.5)	18	(0.5)	0.97
K72_ICD10	169	(0.4)	51	(0.9)	<0.001	101	(0.4)	33	(0.9)	<0.001
K73_ICD10	623	(1.5)	134	(2.3)	<0.001	438	(1.6)	74	(1.9)	0.08
K74_ICD10	943	(2.2)	288	(4.9)	<0.001	645	(2.3)	204	(5.3)	<0.001
K75_ICD10	228	(0.5)	53	(0.9)	<0.001	158	(0.6)	56	(1.5)	<0.001
K76_ICD10	759	(1.8)	126	(2.2)	0.05	461	(1.6)	70	(1.8)	0.38
K77_ICD10	0	(0.0)	0	(0.0)	1	2	(0.0)	0	(0.0)	0.6
K80_ICD10	1152	(2.7)	285	(4.9)	<0.001	763	(2.7)	191	(5.0)	<0.001
K81_ICD10	356	(0.8)	72	(1.2)	0.003	232	(0.8)	68	(1.8)	<0.001
K82_ICD10	150	(0.4)	31	(0.5)	0.04	113	(0.4)	12	(0.3)	0.42

K83_ICD10	679	(1.6)	242	(4.2)	<0.001	464	(1.6)	174	(4.5)	<0.001
K85_ICD10	221	(0.5)	65	(1.1)	<0.001	129	(0.5)	49	(1.3)	<0.001
K86_ICD10	295	(0.7)	85	(1.5)	<0.001	218	(0.8)	55	(1.4)	<0.001
K90_ICD10	21	(0.0)	5	(0.1)	0.26	18	(0.1)	2	(0.1)	0.79
K91_ICD10	23	(0.1)	8	(0.1)	0.02	21	(0.1)	4	(0.1)	0.53
K92_ICD10	2096	(5.0)	428	(7.4)	<0.001	1342	(4.8)	280	(7.3)	<0.001
K94_ICD10	37	(0.1)	6	(0.1)	0.71	27	(0.1)	2	(0.1)	0.4
L01_ICD10	8	(0.0)	4	(0.1)	0.02	5	(0.0)	2	(0.1)	0.18
L02_ICD10	244	(0.6)	36	(0.6)	0.7	155	(0.5)	26	(0.7)	0.31
L03_ICD10	2621	(6.2)	459	(7.9)	<0.001	1814	(6.4)	297	(7.7)	0.002
L04_ICD10	69	(0.2)	11	(0.2)	0.65	47	(0.2)	6	(0.2)	0.89
L05_ICD10	2	(0.0)	0	(0.0)	0.6	0	(0.0)	0	(0.0)	1
L08_ICD10	176	(0.4)	25	(0.4)	0.88	125	(0.4)	20	(0.5)	0.49
L10_ICD10	18	(0.0)	3	(0.1)	0.76	13	(0.0)	4	(0.1)	0.14
L11_ICD10	21	(0.0)	2	(0.0)	0.62	16	(0.1)	2	(0.1)	0.91
L12_ICD10	85	(0.2)	13	(0.2)	0.72	51	(0.2)	8	(0.2)	0.7
L13_ICD10	92	(0.2)	9	(0.2)	0.33	55	(0.2)	7	(0.2)	0.87
L20_ICD10	94	(0.2)	9	(0.2)	0.3	79	(0.3)	6	(0.2)	0.16
L21_ICD10	548	(1.3)	64	(1.1)	0.21	370	(1.3)	52	(1.4)	0.81
L22_ICD10	35	(0.1)	7	(0.1)	0.36	28	(0.1)	5	(0.1)	0.57
L24_ICD10	3	(0.0)	0	(0.0)	0.52	5	(0.0)	1	(0.0)	0.72
L25_ICD10	3221	(7.6)	477	(8.2)	0.12	2152	(7.6)	323	(8.4)	0.08
L26_ICD10	182	(0.4)	22	(0.4)	0.56	121	(0.4)	12	(0.3)	0.3
L27_ICD10	311	(0.7)	45	(0.8)	0.75	171	(0.6)	30	(0.8)	0.19
L28_ICD10	435	(1.0)	50	(0.9)	0.22	317	(1.1)	43	(1.1)	1
L29_ICD10	1024	(2.4)	173	(3.0)	0.01	704	(2.5)	137	(3.6)	<0.001
L30_ICD10	185	(0.4)	21	(0.4)	0.4	151	(0.5)	11	(0.3)	0.04
L40_ICD10	152	(0.4)	17	(0.3)	0.42	102	(0.4)	17	(0.4)	0.43
L41_ICD10	3	(0.0)	0	(0.0)	0.52	7	(0.0)	3	(0.1)	0.08
L42_ICD10	26	(0.1)	1	(0.0)	0.18	20	(0.1)	3	(0.1)	0.87
L43_ICD10	55	(0.1)	9	(0.2)	0.63	38	(0.1)	2	(0.1)	0.18
L44_ICD10	11	(0.0)	1	(0.0)	0.69	8	(0.0)	3	(0.1)	0.12

L50_ICD10	598	(1.4)	87	(1.5)	0.63	415	(1.5)	48	(1.3)	0.29
L51_ICD10	5	(0.0)	1	(0.0)	0.73	5	(0.0)	0	(0.0)	0.41
L52_ICD10	20	(0.0)	0	(0.0)	0.1	16	(0.1)	0	(0.0)	0.14
L53_ICD10	182	(0.4)	25	(0.4)	0.99	133	(0.5)	12	(0.3)	0.17
L55_ICD10	5	(0.0)	0	(0.0)	0.41	0	(0.0)	0	(0.0)	1
L56_ICD10	23	(0.1)	2	(0.0)	0.53	8	(0.0)	2	(0.1)	0.43
L57_ICD10	34	(0.1)	8	(0.1)	0.17	24	(0.1)	5	(0.1)	0.38
L58_ICD10	9	(0.0)	2	(0.0)	0.54	7	(0.0)	1	(0.0)	0.96
L59_ICD10	1	(0.0)	0	(0.0)	0.71	0	(0.0)	0	(0.0)	1
L60_ICD10	155	(0.4)	13	(0.2)	0.08	103	(0.4)	16	(0.4)	0.61
L63_ICD10	29	(0.1)	3	(0.1)	0.64	12	(0.0)	2	(0.1)	0.79
L65_ICD10	120	(0.3)	17	(0.3)	0.91	100	(0.4)	6	(0.2)	0.05
L66_ICD10	573	(1.4)	55	(0.9)	0.01	383	(1.4)	42	(1.1)	0.19
L67_ICD10	3	(0.0)	0	(0.0)	0.52	2	(0.0)	0	(0.0)	0.6
L68_ICD10	0	(0.0)	0	(0.0)	1	2	(0.0)	0	(0.0)	0.6
L70_ICD10	300	(0.7)	19	(0.3)	<0.001	196	(0.7)	12	(0.3)	0.006
L71_ICD10	66	(0.2)	4	(0.1)	0.1	40	(0.1)	6	(0.2)	0.82
L72_ICD10	442	(1.0)	49	(0.8)	0.15	296	(1.0)	41	(1.1)	0.9
L73_ICD10	111	(0.3)	5	(0.1)	0.01	72	(0.3)	2	(0.1)	0.01
L74_ICD10	42	(0.1)	10	(0.2)	0.11	26	(0.1)	2	(0.1)	0.43
L75_ICD10	2	(0.0)	0	(0.0)	0.6	0	(0.0)	0	(0.0)	1
L76_ICD10	3	(0.0)	0	(0.0)	0.52	3	(0.0)	0	(0.0)	0.52
L80_ICD10	52	(0.1)	5	(0.1)	0.44	34	(0.1)	2	(0.1)	0.24
L81_ICD10	198	(0.5)	21	(0.4)	0.25	150	(0.5)	11	(0.3)	0.04
L82_ICD10	359	(0.8)	59	(1.0)	0.2	225	(0.8)	39	(1.0)	0.16
L83_ICD10	6	(0.0)	0	(0.0)	0.36	2	(0.0)	2	(0.1)	0.02
L84_ICD10	287	(0.7)	40	(0.7)	0.94	202	(0.7)	33	(0.9)	0.32
L85_ICD10	850	(2.0)	136	(2.3)	0.1	552	(2.0)	93	(2.4)	0.05
L87_ICD10	1	(0.0)	0	(0.0)	0.71	0	(0.0)	0	(0.0)	1
L88_ICD10	8	(0.0)	0	(0.0)	0.29	6	(0.0)	0	(0.0)	0.37
L89_ICD10	81	(0.2)	13	(0.2)	0.61	39	(0.1)	8	(0.2)	0.28
L90_ICD10	43	(0.1)	9	(0.2)	0.25	31	(0.1)	4	(0.1)	0.92

L91_ICD10	120	(0.3)	14	(0.2)	0.56	71	(0.3)	6	(0.2)	0.26
L92_ICD10	20	(0.0)	6	(0.1)	0.09	22	(0.1)	1	(0.0)	0.26
L93_ICD10	31	(0.1)	2	(0.0)	0.29	25	(0.1)	0	(0.0)	0.07
L94_ICD10	1	(0.0)	0	(0.0)	0.71	2	(0.0)	0	(0.0)	0.6
L95_ICD10	2	(0.0)	1	(0.0)	0.26	2	(0.0)	2	(0.1)	0.02
L97_ICD10	102	(0.2)	15	(0.3)	0.81	83	(0.3)	16	(0.4)	0.2
L98_ICD10	431	(1.0)	55	(0.9)	0.6	273	(1.0)	52	(1.4)	0.02
M00_ICD10	71	(0.2)	9	(0.2)	0.82	45	(0.2)	11	(0.3)	0.08
M01_ICD10	5	(0.0)	1	(0.0)	0.73	2	(0.0)	0	(0.0)	0.6
M02_ICD10	2	(0.0)	1	(0.0)	0.26	2	(0.0)	0	(0.0)	0.6
M06_ICD10	347	(0.8)	42	(0.7)	0.43	200	(0.7)	37	(1.0)	0.08
M08_ICD10	11	(0.0)	1	(0.0)	0.69	7	(0.0)	0	(0.0)	0.33
M10_ICD10	1295	(3.1)	238	(4.1)	<0.001	861	(3.0)	164	(4.3)	<0.001
M11_ICD10	2	(0.0)	0	(0.0)	0.6	2	(0.0)	0	(0.0)	0.6
M12_ICD10	1617	(3.8)	221	(3.8)	0.92	1043	(3.7)	141	(3.7)	0.97
M13_ICD10	99	(0.2)	14	(0.2)	0.92	68	(0.2)	9	(0.2)	0.94
M14_ICD10	0	(0.0)	0	(0.0)	1	1	(0.0)	0	(0.0)	0.71
M15_ICD10	3132	(7.4)	536	(9.2)	<0.001	2016	(7.1)	405	(10.6)	<0.001
M16_ICD10	427	(1.0)	75	(1.3)	0.05	277	(1.0)	41	(1.1)	0.6
M17_ICD10	1820	(4.3)	332	(5.7)	<0.001	1123	(4.0)	230	(6.0)	<0.001
M18_ICD10	100	(0.2)	16	(0.3)	0.58	74	(0.3)	9	(0.2)	0.76
M19_ICD10	732	(1.7)	135	(2.3)	0.002	442	(1.6)	87	(2.3)	0.001
M1A_ICD10	1	(0.0)	0	(0.0)	0.71	3	(0.0)	0	(0.0)	0.52
M20_ICD10	58	(0.1)	14	(0.2)	0.06	48	(0.2)	8	(0.2)	0.59
M21_ICD10	65	(0.2)	6	(0.1)	0.35	37	(0.1)	7	(0.2)	0.42
M22_ICD10	16	(0.0)	0	(0.0)	0.14	13	(0.0)	1	(0.0)	0.58
M23_ICD10	63	(0.1)	4	(0.1)	0.12	36	(0.1)	5	(0.1)	0.96
M24_ICD10	108	(0.3)	12	(0.2)	0.48	79	(0.3)	19	(0.5)	0.02
M25_ICD10	1056	(2.5)	162	(2.8)	0.19	711	(2.5)	100	(2.6)	0.73
M26_ICD10	351	(0.8)	37	(0.6)	0.12	212	(0.8)	21	(0.5)	0.17
M27_ICD10	100	(0.2)	16	(0.3)	0.58	70	(0.2)	14	(0.4)	0.18
M30_ICD10	9	(0.0)	0	(0.0)	0.27	2	(0.0)	0	(0.0)	0.6

M31_ICD10	28	(0.1)	4	(0.1)	0.94	23	(0.1)	5	(0.1)	0.33
M32_ICD10	319	(0.8)	49	(0.8)	0.47	198	(0.7)	31	(0.8)	0.46
M33_ICD10	58	(0.1)	7	(0.1)	0.74	28	(0.1)	5	(0.1)	0.57
M34_ICD10	30	(0.1)	9	(0.2)	0.04	18	(0.1)	1	(0.0)	0.37
M35_ICD10	501	(1.2)	67	(1.2)	0.82	310	(1.1)	47	(1.2)	0.48
M36_ICD10	1	(0.0)	0	(0.0)	0.71	0	(0.0)	0	(0.0)	1
M40_ICD10	40	(0.1)	13	(0.2)	0.005	31	(0.1)	8	(0.2)	0.1
M41_ICD10	260	(0.6)	31	(0.5)	0.45	165	(0.6)	26	(0.7)	0.48
M43_ICD10	369	(0.9)	57	(1.0)	0.42	229	(0.8)	49	(1.3)	0.003
M45_ICD10	104	(0.2)	12	(0.2)	0.56	48	(0.2)	5	(0.1)	0.57
M46_ICD10	88	(0.2)	14	(0.2)	0.61	63	(0.2)	7	(0.2)	0.61
M47_ICD10	2531	(6.0)	448	(7.7)	<0.001	1627	(5.8)	290	(7.6)	<0.001
M48_ICD10	572	(1.4)	114	(2.0)	<0.001	364	(1.3)	76	(2.0)	<0.001
M49_ICD10	4	(0.0)	1	(0.0)	0.59	2	(0.0)	1	(0.0)	0.25
M50_ICD10	37	(0.1)	4	(0.1)	0.65	24	(0.1)	3	(0.1)	0.89
M51_ICD10	469	(1.1)	49	(0.8)	0.06	303	(1.1)	45	(1.2)	0.57
M53_ICD10	77	(0.2)	5	(0.1)	0.1	37	(0.1)	4	(0.1)	0.67
M54_ICD10	2707	(6.4)	393	(6.8)	0.31	1735	(6.1)	298	(7.8)	<0.001
M60_ICD10	1003	(2.4)	155	(2.7)	0.17	702	(2.5)	106	(2.8)	0.3
M61_ICD10	3	(0.0)	1	(0.0)	0.43	3	(0.0)	2	(0.1)	0.05
M62_ICD10	139	(0.3)	23	(0.4)	0.41	101	(0.4)	18	(0.5)	0.28
M65_ICD10	741	(1.8)	119	(2.0)	0.11	476	(1.7)	62	(1.6)	0.76
M66_ICD10	7	(0.0)	2	(0.0)	0.35	4	(0.0)	0	(0.0)	0.46
M67_ICD10	135	(0.3)	12	(0.2)	0.14	78	(0.3)	11	(0.3)	0.9
M70_ICD10	18	(0.0)	1	(0.0)	0.36	14	(0.0)	0	(0.0)	0.17
M71_ICD10	104	(0.2)	13	(0.2)	0.74	57	(0.2)	6	(0.2)	0.55
M72_ICD10	221	(0.5)	28	(0.5)	0.68	134	(0.5)	20	(0.5)	0.69
M75_ICD10	696	(1.6)	103	(1.8)	0.49	454	(1.6)	65	(1.7)	0.68
M76_ICD10	60	(0.1)	8	(0.1)	0.93	33	(0.1)	4	(0.1)	0.83
M77_ICD10	376	(0.9)	41	(0.7)	0.15	218	(0.8)	35	(0.9)	0.35
M79_ICD10	648	(1.5)	117	(2.0)	0.006	434	(1.5)	61	(1.6)	0.8
M81_ICD10	1130	(2.7)	239	(4.1)	<0.001	727	(2.6)	153	(4.0)	<0.001

M83_ICD10	4	(0.0)	0	(0.0)	0.46	2	(0.0)	0	(0.0)	0.6
M84_ICD10	837	(2.0)	201	(3.5)	<0.001	582	(2.1)	124	(3.2)	<0.001
M85_ICD10	6	(0.0)	0	(0.0)	0.36	3	(0.0)	0	(0.0)	0.52
M86_ICD10	164	(0.4)	33	(0.6)	0.04	122	(0.4)	17	(0.4)	0.92
M87_ICD10	74	(0.2)	16	(0.3)	0.1	62	(0.2)	11	(0.3)	0.41
M88_ICD10	0	(0.0)	1	(0.0)	0.007	0	(0.0)	0	(0.0)	1
M89_ICD10	300	(0.7)	55	(0.9)	0.05	208	(0.7)	31	(0.8)	0.62
M92_ICD10	5	(0.0)	0	(0.0)	0.41	2	(0.0)	1	(0.0)	0.25
M93_ICD10	1	(0.0)	0	(0.0)	0.71	1	(0.0)	0	(0.0)	0.71
M94_ICD10	2	(0.0)	0	(0.0)	0.6	4	(0.0)	0	(0.0)	0.46
M95_ICD10	6	(0.0)	0	(0.0)	0.36	1	(0.0)	0	(0.0)	0.71
M96_ICD10	23	(0.1)	2	(0.0)	0.53	18	(0.1)	2	(0.1)	0.79
N00_ICD10	6	(0.0)	0	(0.0)	0.36	2	(0.0)	1	(0.0)	0.25
N01_ICD10	2	(0.0)	0	(0.0)	0.6	0	(0.0)	0	(0.0)	1
N02_ICD10	1	(0.0)	1	(0.0)	0.1	1	(0.0)	0	(0.0)	0.71
N03_ICD10	216	(0.5)	36	(0.6)	0.29	125	(0.4)	46	(1.2)	<0.001
N04_ICD10	108	(0.3)	27	(0.5)	0.005	68	(0.2)	19	(0.5)	0.004
N05_ICD10	168	(0.4)	27	(0.5)	0.45	93	(0.3)	17	(0.4)	0.26
N08_ICD10	17	(0.0)	5	(0.1)	0.13	16	(0.1)	1	(0.0)	0.44
N10_ICD10	390	(0.9)	74	(1.3)	0.01	261	(0.9)	41	(1.1)	0.38
N11_ICD10	4	(0.0)	0	(0.0)	0.46	2	(0.0)	0	(0.0)	0.6
N12_ICD10	85	(0.2)	9	(0.2)	0.45	43	(0.2)	6	(0.2)	0.95
N13_ICD10	635	(1.5)	128	(2.2)	<0.001	401	(1.4)	98	(2.6)	<0.001
N15_ICD10	44	(0.1)	10	(0.2)	0.15	26	(0.1)	5	(0.1)	0.47
N16_ICD10	2	(0.0)	0	(0.0)	0.6	1	(0.0)	0	(0.0)	0.71
N17_ICD10	314	(0.7)	78	(1.3)	<0.001	198	(0.7)	47	(1.2)	<0.001
N18_ICD10	1785	(4.2)	368	(6.3)	<0.001	1252	(4.4)	268	(7.0)	<0.001
N19_ICD10	321	(0.8)	79	(1.4)	<0.001	225	(0.8)	47	(1.2)	0.006
N20_ICD10	1111	(2.6)	229	(3.9)	<0.001	704	(2.5)	123	(3.2)	0.009
N21_ICD10	89	(0.2)	15	(0.3)	0.47	57	(0.2)	9	(0.2)	0.67
N23_ICD10	12	(0.0)	2	(0.0)	0.8	9	(0.0)	1	(0.0)	0.85
N25_ICD10	16	(0.0)	4	(0.1)	0.28	24	(0.1)	4	(0.1)	0.7

N26_ICD10	20	(0.0)	4	(0.1)	0.49	4	(0.0)	2	(0.1)	0.11
N27_ICD10	3	(0.0)	1	(0.0)	0.43	4	(0.0)	0	(0.0)	0.46
N28_ICD10	1508	(3.6)	318	(5.5)	<0.001	1025	(3.6)	236	(6.2)	<0.001
N30_ICD10	268	(0.6)	49	(0.8)	0.07	142	(0.5)	27	(0.7)	0.11
N31_ICD10	800	(1.9)	152	(2.6)	<0.001	580	(2.1)	111	(2.9)	<0.001
N32_ICD10	416	(1.0)	71	(1.2)	0.09	265	(0.9)	43	(1.1)	0.27
N34_ICD10	28	(0.1)	1	(0.0)	0.15	24	(0.1)	3	(0.1)	0.89
N35_ICD10	63	(0.1)	8	(0.1)	0.83	32	(0.1)	4	(0.1)	0.88
N36_ICD10	30	(0.1)	3	(0.1)	0.6	14	(0.0)	3	(0.1)	0.47
N37_ICD10	1	(0.0)	0	(0.0)	0.71	0	(0.0)	0	(0.0)	1
N39_ICD10	4570	(10.8)	816	(14.0)	<0.001	2970	(10.5)	549	(14.3)	<0.001
N40_ICD10	771	(1.8)	142	(2.4)	0.001	472	(1.7)	88	(2.3)	0.006
N41_ICD10	192	(0.5)	22	(0.4)	0.41	131	(0.5)	16	(0.4)	0.69
N42_ICD10	3	(0.0)	0	(0.0)	0.52	4	(0.0)	0	(0.0)	0.46
N43_ICD10	51	(0.1)	6	(0.1)	0.72	19	(0.1)	9	(0.2)	<0.001
N44_ICD10	11	(0.0)	2	(0.0)	0.72	7	(0.0)	1	(0.0)	0.96
N45_ICD10	108	(0.3)	21	(0.4)	0.14	64	(0.2)	12	(0.3)	0.3
N46_ICD10	52	(0.1)	4	(0.1)	0.26	30	(0.1)	4	(0.1)	0.97
N47_ICD10	92	(0.2)	9	(0.2)	0.33	78	(0.3)	6	(0.2)	0.17
N48_ICD10	21	(0.0)	4	(0.1)	0.55	8	(0.0)	0	(0.0)	0.3
N49_ICD10	10	(0.0)	1	(0.0)	0.76	6	(0.0)	2	(0.1)	0.25
N50_ICD10	55	(0.1)	9	(0.2)	0.63	30	(0.1)	3	(0.1)	0.61
N52_ICD10	154	(0.4)	19	(0.3)	0.65	86	(0.3)	19	(0.5)	0.05
N60_ICD10	90	(0.2)	10	(0.2)	0.52	62	(0.2)	6	(0.2)	0.43
N61_ICD10	60	(0.1)	5	(0.1)	0.28	29	(0.1)	4	(0.1)	0.98
N62_ICD10	46	(0.1)	5	(0.1)	0.62	33	(0.1)	5	(0.1)	0.82
N63_ICD10	680	(1.6)	80	(1.4)	0.18	446	(1.6)	40	(1.0)	0.01
N64_ICD10	80	(0.2)	3	(0.1)	0.02	59	(0.2)	5	(0.1)	0.31
N70_ICD10	19	(0.0)	3	(0.1)	0.82	9	(0.0)	1	(0.0)	0.85
N71_ICD10	11	(0.0)	2	(0.0)	0.72	12	(0.0)	0	(0.0)	0.2
N72_ICD10	11	(0.0)	0	(0.0)	0.22	5	(0.0)	0	(0.0)	0.41
N73_ICD10	163	(0.4)	15	(0.3)	0.13	98	(0.3)	6	(0.2)	0.05

N75_ICD10	25	(0.1)	6	(0.1)	0.22	15	(0.1)	2	(0.1)	0.98
N76_ICD10	814	(1.9)	122	(2.1)	0.38	512	(1.8)	60	(1.6)	0.28
N77_ICD10	4	(0.0)	0	(0.0)	0.46	5	(0.0)	0	(0.0)	0.41
N80_ICD10	270	(0.6)	24	(0.4)	0.04	158	(0.6)	11	(0.3)	0.03
N81_ICD10	72	(0.2)	14	(0.2)	0.23	50	(0.2)	14	(0.4)	0.01
N82_ICD10	7	(0.0)	1	(0.0)	0.97	3	(0.0)	2	(0.1)	0.05
N83_ICD10	190	(0.4)	29	(0.5)	0.6	126	(0.4)	14	(0.4)	0.48
N84_ICD10	154	(0.4)	13	(0.2)	0.09	90	(0.3)	6	(0.2)	0.08
N85_ICD10	171	(0.4)	17	(0.3)	0.2	123	(0.4)	7	(0.2)	0.02
N86_ICD10	28	(0.1)	5	(0.1)	0.59	25	(0.1)	3	(0.1)	0.84
N87_ICD10	9	(0.0)	2	(0.0)	0.54	2	(0.0)	0	(0.0)	0.6
N88_ICD10	2	(0.0)	0	(0.0)	0.6	0	(0.0)	0	(0.0)	1
N89_ICD10	23	(0.1)	2	(0.0)	0.53	22	(0.1)	2	(0.1)	0.58
N90_ICD10	13	(0.0)	4	(0.1)	0.15	8	(0.0)	0	(0.0)	0.3
N91_ICD10	93	(0.2)	9	(0.2)	0.31	44	(0.2)	7	(0.2)	0.7
N92_ICD10	454	(1.1)	33	(0.6)	<0.001	307	(1.1)	19	(0.5)	<0.001
N93_ICD10	38	(0.1)	3	(0.1)	0.35	26	(0.1)	3	(0.1)	0.79
N94_ICD10	90	(0.2)	9	(0.2)	0.36	60	(0.2)	2	(0.1)	0.03
N95_ICD10	1036	(2.4)	173	(3.0)	0.02	695	(2.5)	104	(2.7)	0.35
N97_ICD10	199	(0.5)	11	(0.2)	0.002	143	(0.5)	5	(0.1)	0.001
N99_ICD10	3	(0.0)	0	(0.0)	0.52	1	(0.0)	1	(0.0)	0.1
NoD_ICD10	4	(0.0)	1	(0.0)	0.59	3	(0.0)	0	(0.0)	0.52
O00_ICD10	0	(0.0)	0	(0.0)	1	1	(0.0)	0	(0.0)	0.71
O01_ICD10	1	(0.0)	0	(0.0)	0.71	0	(0.0)	0	(0.0)	1
O02_ICD10	9	(0.0)	0	(0.0)	0.27	5	(0.0)	0	(0.0)	0.41
O03_ICD10	10	(0.0)	0	(0.0)	0.24	7	(0.0)	1	(0.0)	0.96
O04_ICD10	1	(0.0)	0	(0.0)	0.71	0	(0.0)	0	(0.0)	1
O08_ICD10	0	(0.0)	0	(0.0)	1	1	(0.0)	0	(0.0)	0.71
O10_ICD10	4	(0.0)	0	(0.0)	0.46	0	(0.0)	0	(0.0)	1
O13_ICD10	7	(0.0)	2	(0.0)	0.35	7	(0.0)	3	(0.1)	0.08
O14_ICD10	16	(0.0)	2	(0.0)	0.9	8	(0.0)	0	(0.0)	0.3
O20_ICD10	139	(0.3)	6	(0.1)	0.003	102	(0.4)	2	(0.1)	0.002

O21_ICD10	4	(0.0)	0	(0.0)	0.46	1	(0.0)	0	(0.0)	0.71
O22_ICD10	1	(0.0)	0	(0.0)	0.71	0	(0.0)	0	(0.0)	1
O23_ICD10	0	(0.0)	1	(0.0)	0.007	1	(0.0)	0	(0.0)	0.71
O24_ICD10	8	(0.0)	3	(0.1)	0.12	4	(0.0)	0	(0.0)	0.46
O26_ICD10	7	(0.0)	2	(0.0)	0.35	2	(0.0)	1	(0.0)	0.25
O30_ICD10	8	(0.0)	1	(0.0)	0.93	3	(0.0)	0	(0.0)	0.52
O31_ICD10	1	(0.0)	0	(0.0)	0.71	0	(0.0)	0	(0.0)	1
O32_ICD10	33	(0.1)	4	(0.1)	0.81	14	(0.0)	0	(0.0)	0.17
O33_ICD10	2	(0.0)	0	(0.0)	0.6	4	(0.0)	1	(0.0)	0.58
O34_ICD10	59	(0.1)	3	(0.1)	0.08	30	(0.1)	3	(0.1)	0.61
O35_ICD10	18	(0.0)	1	(0.0)	0.36	12	(0.0)	0	(0.0)	0.2
O36_ICD10	9	(0.0)	2	(0.0)	0.54	8	(0.0)	0	(0.0)	0.3
O41_ICD10	1	(0.0)	0	(0.0)	0.71	1	(0.0)	0	(0.0)	0.71
O42_ICD10	4	(0.0)	0	(0.0)	0.46	2	(0.0)	0	(0.0)	0.6
O43_ICD10	0	(0.0)	1	(0.0)	0.007	1	(0.0)	0	(0.0)	0.71
O44_ICD10	3	(0.0)	0	(0.0)	0.52	6	(0.0)	0	(0.0)	0.37
O45_ICD10	0	(0.0)	0	(0.0)	1	1	(0.0)	0	(0.0)	0.71
O46_ICD10	2	(0.0)	1	(0.0)	0.26	4	(0.0)	0	(0.0)	0.46
O47_ICD10	2	(0.0)	0	(0.0)	0.6	0	(0.0)	0	(0.0)	1
O48_ICD10	1	(0.0)	0	(0.0)	0.71	5	(0.0)	0	(0.0)	0.41
O60_ICD10	22	(0.1)	3	(0.1)	0.99	20	(0.1)	1	(0.0)	0.31
O61_ICD10	2	(0.0)	0	(0.0)	0.6	0	(0.0)	0	(0.0)	1
O62_ICD10	8	(0.0)	0	(0.0)	0.29	3	(0.0)	0	(0.0)	0.52
O63_ICD10	19	(0.0)	0	(0.0)	0.11	11	(0.0)	1	(0.0)	0.7
O66_ICD10	0	(0.0)	0	(0.0)	1	1	(0.0)	0	(0.0)	0.71
O68_ICD10	10	(0.0)	0	(0.0)	0.24	11	(0.0)	0	(0.0)	0.22
O70_ICD10	143	(0.3)	3	(0.1)	<0.001	96	(0.3)	1	(0.0)	<0.001
O72_ICD10	1	(0.0)	0	(0.0)	0.71	0	(0.0)	0	(0.0)	1
O75_ICD10	27	(0.1)	0	(0.0)	0.05	7	(0.0)	1	(0.0)	0.96
O80_ICD10	2	(0.0)	0	(0.0)	0.6	1	(0.0)	0	(0.0)	0.71
O82_ICD10	8	(0.0)	1	(0.0)	0.93	9	(0.0)	0	(0.0)	0.27
O86_ICD10	1	(0.0)	0	(0.0)	0.71	0	(0.0)	0	(0.0)	1

O87_ICD10	1	(0.0)	0	(0.0)	0.71	0	(0.0)	0	(0.0)	1
O91_ICD10	7	(0.0)	0	(0.0)	0.33	5	(0.0)	0	(0.0)	0.41
O92_ICD10	0	(0.0)	1	(0.0)	0.007	0	(0.0)	0	(0.0)	1
O98_ICD10	2	(0.0)	0	(0.0)	0.6	0	(0.0)	1	(0.0)	0.007
O99_ICD10	21	(0.0)	0	(0.0)	0.09	9	(0.0)	0	(0.0)	0.27
P00_ICD10	0	(0.0)	1	(0.0)	0.007	0	(0.0)	1	(0.0)	0.007
P02_ICD10	1	(0.0)	0	(0.0)	0.71	0	(0.0)	0	(0.0)	1
P07_ICD10	2	(0.0)	0	(0.0)	0.6	0	(0.0)	0	(0.0)	1
P10_ICD10	11	(0.0)	2	(0.0)	0.72	7	(0.0)	0	(0.0)	0.33
P11_ICD10	1	(0.0)	2	(0.0)	0.004	1	(0.0)	0	(0.0)	0.71
P13_ICD10	1	(0.0)	0	(0.0)	0.71	0	(0.0)	0	(0.0)	1
P23_ICD10	1	(0.0)	0	(0.0)	0.71	2	(0.0)	0	(0.0)	0.6
P25_ICD10	0	(0.0)	0	(0.0)	1	3	(0.0)	0	(0.0)	0.52
P26_ICD10	3	(0.0)	0	(0.0)	0.52	0	(0.0)	1	(0.0)	0.007
P28_ICD10	2	(0.0)	2	(0.0)	0.02	6	(0.0)	2	(0.1)	0.25
P35_ICD10	2	(0.0)	0	(0.0)	0.6	1	(0.0)	0	(0.0)	0.71
P38_ICD10	4	(0.0)	1	(0.0)	0.59	0	(0.0)	1	(0.0)	0.007
P54_ICD10	1	(0.0)	0	(0.0)	0.71	1	(0.0)	0	(0.0)	0.71
P55_ICD10	6	(0.0)	0	(0.0)	0.36	2	(0.0)	0	(0.0)	0.6
P58_ICD10	0	(0.0)	0	(0.0)	1	1	(0.0)	0	(0.0)	0.71
P59_ICD10	2	(0.0)	0	(0.0)	0.6	1	(0.0)	0	(0.0)	0.71
P70_ICD10	2	(0.0)	0	(0.0)	0.6	0	(0.0)	1	(0.0)	0.007
P71_ICD10	0	(0.0)	1	(0.0)	0.007	0	(0.0)	0	(0.0)	1
P78_ICD10	1	(0.0)	0	(0.0)	0.71	0	(0.0)	0	(0.0)	1
P80_ICD10	2	(0.0)	1	(0.0)	0.26	3	(0.0)	0	(0.0)	0.52
P91_ICD10	2	(0.0)	0	(0.0)	0.6	1	(0.0)	0	(0.0)	0.71
P95_ICD10	1	(0.0)	0	(0.0)	0.71	0	(0.0)	0	(0.0)	1
Q00_ICD10	0	(0.0)	0	(0.0)	1	2	(0.0)	0	(0.0)	0.6
Q01_ICD10	1	(0.0)	0	(0.0)	0.71	1	(0.0)	0	(0.0)	0.71
Q03_ICD10	8	(0.0)	0	(0.0)	0.29	3	(0.0)	0	(0.0)	0.52
Q04_ICD10	5	(0.0)	0	(0.0)	0.41	10	(0.0)	0	(0.0)	0.24
Q05_ICD10	3	(0.0)	1	(0.0)	0.43	0	(0.0)	1	(0.0)	0.007

Q06_ICD10	9	(0.0)	0	(0.0)	0.27	5	(0.0)	0	(0.0)	0.41
Q07_ICD10	3	(0.0)	0	(0.0)	0.52	1	(0.0)	0	(0.0)	0.71
Q10_ICD10	5	(0.0)	3	(0.1)	0.03	2	(0.0)	0	(0.0)	0.6
Q11_ICD10	1	(0.0)	0	(0.0)	0.71	1	(0.0)	0	(0.0)	0.71
Q12_ICD10	2	(0.0)	1	(0.0)	0.26	1	(0.0)	1	(0.0)	0.1
Q13_ICD10	1	(0.0)	0	(0.0)	0.71	0	(0.0)	0	(0.0)	1
Q14_ICD10	7	(0.0)	0	(0.0)	0.33	2	(0.0)	0	(0.0)	0.6
Q15_ICD10	5	(0.0)	0	(0.0)	0.41	3	(0.0)	0	(0.0)	0.52
Q16_ICD10	62	(0.1)	13	(0.2)	0.16	41	(0.1)	9	(0.2)	0.19
Q18_ICD10	5	(0.0)	1	(0.0)	0.73	4	(0.0)	2	(0.1)	0.11
Q20_ICD10	30	(0.1)	5	(0.1)	0.69	18	(0.1)	3	(0.1)	0.74
Q21_ICD10	57	(0.1)	9	(0.2)	0.7	41	(0.1)	7	(0.2)	0.57
Q22_ICD10	5	(0.0)	0	(0.0)	0.41	1	(0.0)	0	(0.0)	0.71
Q23_ICD10	16	(0.0)	3	(0.1)	0.62	6	(0.0)	3	(0.1)	0.05
Q24_ICD10	6	(0.0)	2	(0.0)	0.26	5	(0.0)	0	(0.0)	0.41
Q25_ICD10	20	(0.0)	7	(0.1)	0.03	14	(0.0)	0	(0.0)	0.17
Q26_ICD10	1	(0.0)	0	(0.0)	0.71	2	(0.0)	0	(0.0)	0.6
Q27_ICD10	9	(0.0)	1	(0.0)	0.84	4	(0.0)	1	(0.0)	0.58
Q28_ICD10	39	(0.1)	4	(0.1)	0.57	20	(0.1)	3	(0.1)	0.87
Q31_ICD10	2	(0.0)	0	(0.0)	0.6	0	(0.0)	0	(0.0)	1
Q33_ICD10	7	(0.0)	0	(0.0)	0.33	2	(0.0)	0	(0.0)	0.6
Q35_ICD10	1	(0.0)	1	(0.0)	0.1	2	(0.0)	1	(0.0)	0.25
Q38_ICD10	0	(0.0)	1	(0.0)	0.007	2	(0.0)	0	(0.0)	0.6
Q39_ICD10	6	(0.0)	0	(0.0)	0.36	2	(0.0)	0	(0.0)	0.6
Q40_ICD10	2	(0.0)	2	(0.0)	0.02	3	(0.0)	3	(0.1)	0.004
Q41_ICD10	1	(0.0)	2	(0.0)	0.004	2	(0.0)	4	(0.1)	<0.001
Q42_ICD10	7	(0.0)	2	(0.0)	0.35	4	(0.0)	3	(0.1)	0.01
Q43_ICD10	17	(0.0)	4	(0.1)	0.33	7	(0.0)	4	(0.1)	0.01
Q44_ICD10	59	(0.1)	16	(0.3)	0.01	36	(0.1)	11	(0.3)	0.02
Q45_ICD10	20	(0.0)	6	(0.1)	0.09	6	(0.0)	5	(0.1)	<0.001
Q51_ICD10	8	(0.0)	1	(0.0)	0.93	2	(0.0)	1	(0.0)	0.25
Q53_ICD10	0	(0.0)	0	(0.0)	1	4	(0.0)	0	(0.0)	0.46

Q54_ICD10	6	(0.0)	0	(0.0)	0.36	3	(0.0)	0	(0.0)	0.52
Q55_ICD10	3	(0.0)	0	(0.0)	0.52	4	(0.0)	1	(0.0)	0.58
Q60_ICD10	7	(0.0)	0	(0.0)	0.33	4	(0.0)	1	(0.0)	0.58
Q61_ICD10	94	(0.2)	22	(0.4)	0.02	65	(0.2)	18	(0.5)	0.006
Q62_ICD10	20	(0.0)	5	(0.1)	0.23	9	(0.0)	5	(0.1)	0.006
Q63_ICD10	8	(0.0)	0	(0.0)	0.29	7	(0.0)	1	(0.0)	0.96
Q64_ICD10	1	(0.0)	1	(0.0)	0.1	6	(0.0)	0	(0.0)	0.37
Q65_ICD10	1	(0.0)	0	(0.0)	0.71	2	(0.0)	0	(0.0)	0.6
Q66_ICD10	32	(0.1)	10	(0.2)	0.02	28	(0.1)	2	(0.1)	0.37
Q67_ICD10	10	(0.0)	0	(0.0)	0.24	5	(0.0)	0	(0.0)	0.41
Q68_ICD10	2	(0.0)	0	(0.0)	0.6	0	(0.0)	0	(0.0)	1
Q74_ICD10	15	(0.0)	3	(0.1)	0.55	6	(0.0)	2	(0.1)	0.25
Q75_ICD10	1	(0.0)	0	(0.0)	0.71	2	(0.0)	0	(0.0)	0.6
Q76_ICD10	55	(0.1)	11	(0.2)	0.25	33	(0.1)	8	(0.2)	0.14
Q78_ICD10	9	(0.0)	4	(0.1)	0.04	7	(0.0)	1	(0.0)	0.96
Q79_ICD10	7	(0.0)	0	(0.0)	0.33	5	(0.0)	0	(0.0)	0.41
Q80_ICD10	14	(0.0)	1	(0.0)	0.52	11	(0.0)	1	(0.0)	0.7
Q81_ICD10	14	(0.0)	3	(0.1)	0.48	14	(0.0)	3	(0.1)	0.47
Q82_ICD10	1	(0.0)	0	(0.0)	0.71	2	(0.0)	0	(0.0)	0.6
Q85_ICD10	30	(0.1)	7	(0.1)	0.2	14	(0.0)	6	(0.2)	0.01
Q87_ICD10	8	(0.0)	2	(0.0)	0.44	2	(0.0)	1	(0.0)	0.25
Q89_ICD10	5	(0.0)	0	(0.0)	0.41	2	(0.0)	0	(0.0)	0.6
Q90_ICD10	3	(0.0)	0	(0.0)	0.52	2	(0.0)	0	(0.0)	0.6
Q92_ICD10	5	(0.0)	0	(0.0)	0.41	2	(0.0)	0	(0.0)	0.6
Q96_ICD10	2	(0.0)	0	(0.0)	0.6	0	(0.0)	0	(0.0)	1
Q97_ICD10	1	(0.0)	0	(0.0)	0.71	0	(0.0)	0	(0.0)	1
Q98_ICD10	1	(0.0)	0	(0.0)	0.71	0	(0.0)	0	(0.0)	1
Q99_ICD10	6	(0.0)	0	(0.0)	0.36	8	(0.0)	0	(0.0)	0.3
R00_ICD10	563	(1.3)	79	(1.4)	0.87	411	(1.5)	53	(1.4)	0.73
R01_ICD10	74	(0.2)	12	(0.2)	0.6	54	(0.2)	8	(0.2)	0.82
R03_ICD10	72	(0.2)	5	(0.1)	0.13	35	(0.1)	6	(0.2)	0.6
R04_ICD10	396	(0.9)	47	(0.8)	0.33	273	(1.0)	44	(1.1)	0.29

R05_ICD10	2714	(6.4)	400	(6.9)	0.19	1786	(6.3)	242	(6.3)	0.98
R06_ICD10	1581	(3.7)	224	(3.8)	0.68	1067	(3.8)	175	(4.6)	0.02
R07_ICD10	2015	(4.8)	286	(4.9)	0.62	1323	(4.7)	200	(5.2)	0.14
R09_ICD10	22	(0.1)	2	(0.0)	0.57	12	(0.0)	2	(0.1)	0.79
R10_ICD10	3546	(8.4)	640	(11.0)	<0.001	2310	(8.2)	444	(11.6)	<0.001
R11_ICD10	497	(1.2)	95	(1.6)	0.003	313	(1.1)	53	(1.4)	0.13
R12_ICD10	5	(0.0)	0	(0.0)	0.41	0	(0.0)	0	(0.0)	1
R13_ICD10	115	(0.3)	21	(0.4)	0.23	91	(0.3)	7	(0.2)	0.14
R14_ICD10	100	(0.2)	16	(0.3)	0.58	65	(0.2)	8	(0.2)	0.79
R15_ICD10	2	(0.0)	0	(0.0)	0.6	2	(0.0)	2	(0.1)	0.02
R16_ICD10	141	(0.3)	37	(0.6)	<0.001	90	(0.3)	23	(0.6)	0.006
R17_ICD10	243	(0.6)	115	(2.0)	<0.001	187	(0.7)	68	(1.8)	<0.001
R18_ICD10	38	(0.1)	6	(0.1)	0.75	24	(0.1)	9	(0.2)	0.007
R19_ICD10	487	(1.2)	89	(1.5)	0.01	329	(1.2)	56	(1.5)	0.11
R20_ICD10	276	(0.7)	43	(0.7)	0.45	164	(0.6)	29	(0.8)	0.19
R21_ICD10	98	(0.2)	19	(0.3)	0.17	69	(0.2)	10	(0.3)	0.85
R22_ICD10	746	(1.8)	84	(1.4)	0.08	518	(1.8)	48	(1.3)	0.01
R23_ICD10	29	(0.1)	12	(0.2)	<0.001	25	(0.1)	4	(0.1)	0.76
R25_ICD10	297	(0.7)	38	(0.7)	0.67	198	(0.7)	46	(1.2)	<0.001
R26_ICD10	51	(0.1)	9	(0.2)	0.49	24	(0.1)	5	(0.1)	0.38
R27_ICD10	3	(0.0)	0	(0.0)	0.52	2	(0.0)	0	(0.0)	0.6
R29_ICD10	83	(0.2)	11	(0.2)	0.91	33	(0.1)	6	(0.2)	0.51
R30_ICD10	173	(0.4)	34	(0.6)	0.06	109	(0.4)	25	(0.7)	0.02
R31_ICD10	216	(0.5)	44	(0.8)	0.02	115	(0.4)	16	(0.4)	0.93
R32_ICD10	214	(0.5)	38	(0.7)	0.15	141	(0.5)	31	(0.8)	0.01
R33_ICD10	483	(1.1)	83	(1.4)	0.06	318	(1.1)	65	(1.7)	0.002
R34_ICD10	19	(0.0)	2	(0.0)	0.72	8	(0.0)	1	(0.0)	0.94
R35_ICD10	577	(1.4)	97	(1.7)	0.07	375	(1.3)	59	(1.5)	0.29
R36_ICD10	5	(0.0)	0	(0.0)	0.41	0	(0.0)	0	(0.0)	1
R39_ICD10	26	(0.1)	1	(0.0)	0.18	5	(0.0)	2	(0.1)	0.18
R40_ICD10	49	(0.1)	6	(0.1)	0.79	46	(0.2)	7	(0.2)	0.78
R41_ICD10	20	(0.0)	8	(0.1)	0.008	9	(0.0)	4	(0.1)	0.04

R42_ICD10	1915	(4.5)	349	(6.0)	<0.001	1310	(4.6)	215	(5.6)	0.008
R43_ICD10	27	(0.1)	4	(0.1)	0.89	15	(0.1)	4	(0.1)	0.22
R44_ICD10	8	(0.0)	2	(0.0)	0.44	5	(0.0)	0	(0.0)	0.41
R45_ICD10	3	(0.0)	0	(0.0)	0.52	1	(0.0)	1	(0.0)	0.1
R47_ICD10	79	(0.2)	7	(0.1)	0.26	50	(0.2)	6	(0.2)	0.78
R48_ICD10	3	(0.0)	0	(0.0)	0.52	2	(0.0)	0	(0.0)	0.6
R49_ICD10	169	(0.4)	25	(0.4)	0.74	115	(0.4)	14	(0.4)	0.7
R50_ICD10	1165	(2.8)	204	(3.5)	0.001	754	(2.7)	148	(3.9)	<0.001
R51_ICD10	160	(0.4)	19	(0.3)	0.54	90	(0.3)	10	(0.3)	0.55
R52_ICD10	5	(0.0)	2	(0.0)	0.18	3	(0.0)	0	(0.0)	0.52
R53_ICD10	109	(0.3)	18	(0.3)	0.47	88	(0.3)	13	(0.3)	0.77
R55_ICD10	374	(0.9)	79	(1.4)	<0.001	301	(1.1)	47	(1.2)	0.37
R56_ICD10	414	(1.0)	60	(1.0)	0.71	265	(0.9)	43	(1.1)	0.27
R57_ICD10	318	(0.8)	84	(1.4)	<0.001	242	(0.9)	63	(1.6)	<0.001
R58_ICD10	3	(0.0)	1	(0.0)	0.43	4	(0.0)	0	(0.0)	0.46
R59_ICD10	188	(0.4)	14	(0.2)	0.02	133	(0.5)	12	(0.3)	0.17
R60_ICD10	1215	(2.9)	251	(4.3)	<0.001	792	(2.8)	158	(4.1)	<0.001
R61_ICD10	11	(0.0)	1	(0.0)	0.69	6	(0.0)	1	(0.0)	0.85
R62_ICD10	12	(0.0)	1	(0.0)	0.63	9	(0.0)	1	(0.0)	0.85
R63_ICD10	622	(1.5)	131	(2.3)	<0.001	477	(1.7)	50	(1.3)	0.08
R64_ICD10	176	(0.4)	50	(0.9)	<0.001	133	(0.5)	35	(0.9)	<0.001
R65_ICD10	31	(0.1)	8	(0.1)	0.11	21	(0.1)	7	(0.2)	0.03
R68_ICD10	227	(0.5)	33	(0.6)	0.77	159	(0.6)	15	(0.4)	0.17
R69_ICD10	1	(0.0)	0	(0.0)	0.71	0	(0.0)	0	(0.0)	1
R73_ICD10	53	(0.1)	15	(0.3)	0.01	35	(0.1)	4	(0.1)	0.74
R74_ICD10	4	(0.0)	0	(0.0)	0.46	0	(0.0)	1	(0.0)	0.007
R75_ICD10	1	(0.0)	0	(0.0)	0.71	0	(0.0)	0	(0.0)	1
R76_ICD10	60	(0.1)	10	(0.2)	0.57	44	(0.2)	5	(0.1)	0.71
R78_ICD10	1353	(3.2)	288	(4.9)	<0.001	972	(3.4)	196	(5.1)	<0.001
R79_ICD10	4	(0.0)	4	(0.1)	0.001	4	(0.0)	1	(0.0)	0.58
R80_ICD10	360	(0.9)	60	(1.0)	0.17	237	(0.8)	37	(1.0)	0.43
R81_ICD10	3	(0.0)	0	(0.0)	0.52	0	(0.0)	0	(0.0)	1

R82_ICD10	51	(0.1)	10	(0.2)	0.3	29	(0.1)	4	(0.1)	0.98
R83_ICD10	1	(0.0)	0	(0.0)	0.71	0	(0.0)	0	(0.0)	1
R85_ICD10	2	(0.0)	1	(0.0)	0.26	2	(0.0)	0	(0.0)	0.6
R87_ICD10	3	(0.0)	2	(0.0)	0.06	3	(0.0)	0	(0.0)	0.52
R88_ICD10	0	(0.0)	0	(0.0)	1	1	(0.0)	0	(0.0)	0.71
R91_ICD10	3	(0.0)	0	(0.0)	0.52	6	(0.0)	1	(0.0)	0.85
R93_ICD10	107	(0.3)	6	(0.1)	0.03	42	(0.1)	5	(0.1)	0.78
R94_ICD10	819	(1.9)	120	(2.1)	0.52	478	(1.7)	72	(1.9)	0.4
R97_ICD10	376	(0.9)	92	(1.6)	<0.001	254	(0.9)	45	(1.2)	0.1
R99_ICD10	3	(0.0)	1	(0.0)	0.43	2	(0.0)	0	(0.0)	0.6
S00_ICD10	175	(0.4)	28	(0.5)	0.46	104	(0.4)	20	(0.5)	0.15
S01_ICD10	149	(0.4)	28	(0.5)	0.13	71	(0.3)	17	(0.4)	0.03
S02_ICD10	356	(0.8)	54	(0.9)	0.5	215	(0.8)	33	(0.9)	0.51
S03_ICD10	4	(0.0)	2	(0.0)	0.11	2	(0.0)	3	(0.1)	<0.001
S04_ICD10	5	(0.0)	2	(0.0)	0.18	2	(0.0)	0	(0.0)	0.6
S05_ICD10	33	(0.1)	9	(0.2)	0.06	14	(0.0)	5	(0.1)	0.05
S06_ICD10	262	(0.6)	31	(0.5)	0.42	172	(0.6)	31	(0.8)	0.14
S07_ICD10	1	(0.0)	0	(0.0)	0.71	1	(0.0)	0	(0.0)	0.71
S09_ICD10	833	(2.0)	135	(2.3)	0.08	502	(1.8)	74	(1.9)	0.5
S11_ICD10	2	(0.0)	0	(0.0)	0.6	2	(0.0)	0	(0.0)	0.6
S12_ICD10	224	(0.5)	31	(0.5)	0.98	151	(0.5)	23	(0.6)	0.6
S13_ICD10	130	(0.3)	9	(0.2)	0.04	69	(0.2)	7	(0.2)	0.46
S14_ICD10	80	(0.2)	11	(0.2)	1	54	(0.2)	8	(0.2)	0.82
S15_ICD10	1	(0.0)	1	(0.0)	0.1	0	(0.0)	0	(0.0)	1
S19_ICD10	1	(0.0)	0	(0.0)	0.71	1	(0.0)	0	(0.0)	0.71
S20_ICD10	181	(0.4)	28	(0.5)	0.56	127	(0.4)	20	(0.5)	0.53
S21_ICD10	29	(0.1)	12	(0.2)	<0.001	17	(0.1)	4	(0.1)	0.32
S22_ICD10	149	(0.4)	21	(0.4)	0.92	115	(0.4)	10	(0.3)	0.17
S23_ICD10	282	(0.7)	28	(0.5)	0.1	195	(0.7)	29	(0.8)	0.64
S24_ICD10	9	(0.0)	0	(0.0)	0.27	2	(0.0)	0	(0.0)	0.6
S25_ICD10	1	(0.0)	0	(0.0)	0.71	0	(0.0)	0	(0.0)	1
S26_ICD10	1	(0.0)	0	(0.0)	0.71	0	(0.0)	0	(0.0)	1

S27_ICD10	10	(0.0)	0	(0.0)	0.24	5	(0.0)	1	(0.0)	0.72
S30_ICD10	104	(0.2)	20	(0.3)	0.17	63	(0.2)	11	(0.3)	0.44
S31_ICD10	657	(1.6)	117	(2.0)	0.009	473	(1.7)	60	(1.6)	0.62
S32_ICD10	183	(0.4)	33	(0.6)	0.15	129	(0.5)	28	(0.7)	0.02
S33_ICD10	52	(0.1)	1	(0.0)	0.02	31	(0.1)	2	(0.1)	0.3
S34_ICD10	20	(0.0)	0	(0.0)	0.1	4	(0.0)	1	(0.0)	0.58
S35_ICD10	0	(0.0)	5	(0.1)	<0.001	5	(0.0)	2	(0.1)	0.18
S36_ICD10	7	(0.0)	3	(0.1)	0.08	4	(0.0)	0	(0.0)	0.46
S37_ICD10	6	(0.0)	3	(0.1)	0.05	11	(0.0)	3	(0.1)	0.27
S40_ICD10	85	(0.2)	14	(0.2)	0.53	57	(0.2)	11	(0.3)	0.28
S41_ICD10	29	(0.1)	5	(0.1)	0.64	30	(0.1)	5	(0.1)	0.67
S42_ICD10	252	(0.6)	53	(0.9)	0.005	159	(0.6)	36	(0.9)	0.005
S43_ICD10	306	(0.7)	65	(1.1)	0.001	178	(0.6)	31	(0.8)	0.2
S44_ICD10	3	(0.0)	0	(0.0)	0.52	2	(0.0)	1	(0.0)	0.25
S46_ICD10	2	(0.0)	0	(0.0)	0.6	0	(0.0)	0	(0.0)	1
S47_ICD10	0	(0.0)	1	(0.0)	0.007	0	(0.0)	0	(0.0)	1
S48_ICD10	2	(0.0)	0	(0.0)	0.6	1	(0.0)	0	(0.0)	0.71
S49_ICD10	24	(0.1)	7	(0.1)	0.07	21	(0.1)	8	(0.2)	0.009
S50_ICD10	49	(0.1)	13	(0.2)	0.03	34	(0.1)	5	(0.1)	0.87
S51_ICD10	21	(0.0)	1	(0.0)	0.28	17	(0.1)	2	(0.1)	0.85
S52_ICD10	234	(0.6)	43	(0.7)	0.08	170	(0.6)	33	(0.9)	0.06
S53_ICD10	21	(0.0)	1	(0.0)	0.28	15	(0.1)	1	(0.0)	0.48
S57_ICD10	0	(0.0)	1	(0.0)	0.007	0	(0.0)	0	(0.0)	1
S58_ICD10	1	(0.0)	0	(0.0)	0.71	0	(0.0)	0	(0.0)	1
S59_ICD10	6	(0.0)	1	(0.0)	0.86	8	(0.0)	4	(0.1)	0.02
S60_ICD10	97	(0.2)	14	(0.2)	0.87	54	(0.2)	12	(0.3)	0.12
S61_ICD10	152	(0.4)	17	(0.3)	0.42	90	(0.3)	13	(0.3)	0.83
S62_ICD10	87	(0.2)	15	(0.3)	0.42	56	(0.2)	2	(0.1)	0.05
S63_ICD10	89	(0.2)	6	(0.1)	0.08	68	(0.2)	6	(0.2)	0.31
S64_ICD10	1	(0.0)	0	(0.0)	0.71	0	(0.0)	0	(0.0)	1
S66_ICD10	0	(0.0)	0	(0.0)	1	1	(0.0)	0	(0.0)	0.71
S67_ICD10	3	(0.0)	3	(0.1)	0.004	0	(0.0)	1	(0.0)	0.007

S68_ICD10	5	(0.0)	1	(0.0)	0.73	3	(0.0)	0	(0.0)	0.52
S69_ICD10	100	(0.2)	7	(0.1)	0.08	61	(0.2)	9	(0.2)	0.81
S70_ICD10	177	(0.4)	46	(0.8)	<0.001	131	(0.5)	25	(0.7)	0.12
S71_ICD10	4	(0.0)	1	(0.0)	0.59	3	(0.0)	1	(0.0)	0.42
S72_ICD10	621	(1.5)	143	(2.5)	<0.001	426	(1.5)	83	(2.2)	0.002
S73_ICD10	124	(0.3)	24	(0.4)	0.12	73	(0.3)	19	(0.5)	0.01
S75_ICD10	2	(0.0)	0	(0.0)	0.6	0	(0.0)	0	(0.0)	1
S76_ICD10	0	(0.0)	0	(0.0)	1	1	(0.0)	0	(0.0)	0.71
S78_ICD10	18	(0.0)	4	(0.1)	0.38	16	(0.1)	1	(0.0)	0.44
S79_ICD10	49	(0.1)	17	(0.3)	<0.001	33	(0.1)	8	(0.2)	0.14
S80_ICD10	141	(0.3)	23	(0.4)	0.45	108	(0.4)	14	(0.4)	0.87
S81_ICD10	280	(0.7)	41	(0.7)	0.71	174	(0.6)	26	(0.7)	0.65
S82_ICD10	210	(0.5)	32	(0.5)	0.59	144	(0.5)	28	(0.7)	0.08
S83_ICD10	247	(0.6)	31	(0.5)	0.63	144	(0.5)	15	(0.4)	0.33
S86_ICD10	7	(0.0)	1	(0.0)	0.97	2	(0.0)	0	(0.0)	0.6
S87_ICD10	0	(0.0)	0	(0.0)	1	2	(0.0)	1	(0.0)	0.25
S88_ICD10	14	(0.0)	1	(0.0)	0.52	6	(0.0)	1	(0.0)	0.85
S89_ICD10	78	(0.2)	12	(0.2)	0.72	53	(0.2)	8	(0.2)	0.78
S90_ICD10	67	(0.2)	16	(0.3)	0.04	62	(0.2)	12	(0.3)	0.26
S91_ICD10	161	(0.4)	28	(0.5)	0.25	118	(0.4)	30	(0.8)	0.002
S92_ICD10	86	(0.2)	12	(0.2)	0.96	67	(0.2)	5	(0.1)	0.19
S93_ICD10	191	(0.5)	29	(0.5)	0.62	155	(0.5)	11	(0.3)	0.03
S97_ICD10	8	(0.0)	1	(0.0)	0.93	3	(0.0)	2	(0.1)	0.05
S98_ICD10	10	(0.0)	3	(0.1)	0.22	6	(0.0)	1	(0.0)	0.85
T07_ICD10	244	(0.6)	30	(0.5)	0.56	139	(0.5)	18	(0.5)	0.85
T14_ICD10	131	(0.3)	18	(0.3)	0.99	93	(0.3)	18	(0.5)	0.16
T15_ICD10	41	(0.1)	3	(0.1)	0.28	34	(0.1)	2	(0.1)	0.24
T16_ICD10	5	(0.0)	0	(0.0)	0.41	5	(0.0)	1	(0.0)	0.72
T17_ICD10	40	(0.1)	4	(0.1)	0.54	21	(0.1)	1	(0.0)	0.28
T18_ICD10	43	(0.1)	3	(0.1)	0.25	13	(0.0)	1	(0.0)	0.58
T19_ICD10	1	(0.0)	0	(0.0)	0.71	0	(0.0)	0	(0.0)	1
T20_ICD10	15	(0.0)	1	(0.0)	0.47	7	(0.0)	2	(0.1)	0.34

T21_ICD10	14	(0.0)	2	(0.0)	0.96	12	(0.0)	4	(0.1)	0.11
T22_ICD10	8	(0.0)	0	(0.0)	0.29	15	(0.1)	1	(0.0)	0.48
T23_ICD10	11	(0.0)	1	(0.0)	0.69	10	(0.0)	0	(0.0)	0.24
T24_ICD10	21	(0.0)	7	(0.1)	0.04	19	(0.1)	1	(0.0)	0.34
T25_ICD10	4	(0.0)	0	(0.0)	0.46	3	(0.0)	0	(0.0)	0.52
T26_ICD10	3	(0.0)	0	(0.0)	0.52	2	(0.0)	1	(0.0)	0.25
T28_ICD10	2	(0.0)	0	(0.0)	0.6	0	(0.0)	0	(0.0)	1
T30_ICD10	98	(0.2)	18	(0.3)	0.26	68	(0.2)	15	(0.4)	0.08
T31_ICD10	12	(0.0)	1	(0.0)	0.63	13	(0.0)	2	(0.1)	0.87
T33_ICD10	0	(0.0)	1	(0.0)	0.007	0	(0.0)	0	(0.0)	1
T42_ICD10	3	(0.0)	2	(0.0)	0.06	2	(0.0)	1	(0.0)	0.25
T43_ICD10	1	(0.0)	0	(0.0)	0.71	1	(0.0)	0	(0.0)	0.71
T45_ICD10	1	(0.0)	0	(0.0)	0.71	1	(0.0)	0	(0.0)	0.71
T46_ICD10	3	(0.0)	0	(0.0)	0.52	0	(0.0)	0	(0.0)	1
T48_ICD10	2	(0.0)	1	(0.0)	0.26	2	(0.0)	0	(0.0)	0.6
T50_ICD10	15	(0.0)	0	(0.0)	0.15	9	(0.0)	2	(0.1)	0.52
T56_ICD10	6	(0.0)	0	(0.0)	0.36	2	(0.0)	1	(0.0)	0.25
T57_ICD10	0	(0.0)	1	(0.0)	0.007	2	(0.0)	0	(0.0)	0.6
T58_ICD10	2	(0.0)	0	(0.0)	0.6	1	(0.0)	0	(0.0)	0.71
T60_ICD10	1	(0.0)	0	(0.0)	0.71	0	(0.0)	0	(0.0)	1
T63_ICD10	0	(0.0)	0	(0.0)	1	1	(0.0)	0	(0.0)	0.71
T65_ICD10	2	(0.0)	1	(0.0)	0.26	1	(0.0)	0	(0.0)	0.71
T66_ICD10	0	(0.0)	0	(0.0)	1	1	(0.0)	0	(0.0)	0.71
T67_ICD10	0	(0.0)	0	(0.0)	1	1	(0.0)	0	(0.0)	0.71
T68_ICD10	6	(0.0)	0	(0.0)	0.36	4	(0.0)	1	(0.0)	0.58
T69_ICD10	3	(0.0)	1	(0.0)	0.43	3	(0.0)	0	(0.0)	0.52
T70_ICD10	2	(0.0)	0	(0.0)	0.6	1	(0.0)	0	(0.0)	0.71
T75_ICD10	3	(0.0)	0	(0.0)	0.52	0	(0.0)	0	(0.0)	1
T78_ICD10	184	(0.4)	14	(0.2)	0.03	96	(0.3)	13	(0.3)	0.99
T79_ICD10	43	(0.1)	5	(0.1)	0.72	19	(0.1)	4	(0.1)	0.42
T80_ICD10	4	(0.0)	1	(0.0)	0.59	2	(0.0)	0	(0.0)	0.6
T81_ICD10	50	(0.1)	11	(0.2)	0.16	29	(0.1)	4	(0.1)	0.98

T82_ICD10	63	(0.1)	23	(0.4)	<0.001	33	(0.1)	8	(0.2)	0.14
T83_ICD10	8	(0.0)	0	(0.0)	0.29	3	(0.0)	2	(0.1)	0.05
T84_ICD10	22	(0.1)	3	(0.1)	0.99	11	(0.0)	4	(0.1)	0.08
T85_ICD10	82	(0.2)	17	(0.3)	0.12	48	(0.2)	6	(0.2)	0.85
T86_ICD10	103	(0.2)	12	(0.2)	0.58	67	(0.2)	10	(0.3)	0.78
T87_ICD10	1	(0.0)	0	(0.0)	0.71	0	(0.0)	0	(0.0)	1
V81_ICD10	1	(0.0)	0	(0.0)	0.71	1	(0.0)	0	(0.0)	0.71
V90_ICD10	2	(0.0)	1	(0.0)	0.26	1	(0.0)	0	(0.0)	0.71
V91_ICD10	4	(0.0)	1	(0.0)	0.59	2	(0.0)	0	(0.0)	0.6
V93_ICD10	30	(0.1)	9	(0.2)	0.04	19	(0.1)	5	(0.1)	0.18
W06_ICD10	0	(0.0)	0	(0.0)	1	0	(0.0)	1	(0.0)	0.007
W07_ICD10	1	(0.0)	0	(0.0)	0.71	0	(0.0)	0	(0.0)	1
W18_ICD10	2	(0.0)	1	(0.0)	0.26	0	(0.0)	0	(0.0)	1
W19_ICD10	7	(0.0)	2	(0.0)	0.35	4	(0.0)	1	(0.0)	0.58
W46_ICD10	1	(0.0)	0	(0.0)	0.71	0	(0.0)	0	(0.0)	1
W55_ICD10	2	(0.0)	0	(0.0)	0.6	0	(0.0)	0	(0.0)	1
W57_ICD10	0	(0.0)	0	(0.0)	1	1	(0.0)	0	(0.0)	0.71
X58_ICD10	5	(0.0)	0	(0.0)	0.41	5	(0.0)	1	(0.0)	0.72
Y83_ICD10	1	(0.0)	0	(0.0)	0.71	0	(0.0)	0	(0.0)	1
Y84_ICD10	0	(0.0)	1	(0.0)	0.007	0	(0.0)	0	(0.0)	1
Y93_ICD10	5	(0.0)	1	(0.0)	0.73	0	(0.0)	0	(0.0)	1
Z00_ICD10	5449	(12.9)	795	(13.7)	0.1	3531	(12.5)	508	(13.3)	0.19
Z01_ICD10	417	(1.0)	56	(1.0)	0.86	264	(0.9)	33	(0.9)	0.66
Z02_ICD10	636	(1.5)	86	(1.5)	0.88	413	(1.5)	44	(1.1)	0.12
Z03_ICD10	0	(0.0)	0	(0.0)	1	4	(0.0)	0	(0.0)	0.46
Z04_ICD10	15	(0.0)	2	(0.0)	0.97	11	(0.0)	1	(0.0)	0.7
Z08_ICD10	4	(0.0)	0	(0.0)	0.46	1	(0.0)	0	(0.0)	0.71
Z09_ICD10	1731	(4.1)	262	(4.5)	0.14	1143	(4.0)	180	(4.7)	0.06
Z11_ICD10	1	(0.0)	0	(0.0)	0.71	2	(0.0)	0	(0.0)	0.6
Z12_ICD10	2027	(4.8)	289	(5.0)	0.57	1283	(4.5)	176	(4.6)	0.89
Z13_ICD10	2	(0.0)	0	(0.0)	0.6	1	(0.0)	0	(0.0)	0.71
Z16_ICD10	3	(0.0)	0	(0.0)	0.52	3	(0.0)	0	(0.0)	0.52

Z18_ICD10	4	(0.0)	0	(0.0)	0.46	0	(0.0)	0	(0.0)	1
Z20_ICD10	119	(0.3)	15	(0.3)	0.75	84	(0.3)	8	(0.2)	0.34
Z21_ICD10	202	(0.5)	18	(0.3)	0.07	143	(0.5)	13	(0.3)	0.16
Z22_ICD10	894	(2.1)	157	(2.7)	0.004	557	(2.0)	107	(2.8)	<0.001
Z23_ICD10	984	(2.3)	113	(1.9)	0.06	605	(2.1)	71	(1.9)	0.24
Z30_ICD10	13	(0.0)	0	(0.0)	0.18	9	(0.0)	0	(0.0)	0.27
Z31_ICD10	7	(0.0)	2	(0.0)	0.35	13	(0.0)	0	(0.0)	0.18
Z32_ICD10	25	(0.1)	1	(0.0)	0.2	15	(0.1)	0	(0.0)	0.15
Z33_ICD10	21	(0.0)	0	(0.0)	0.09	13	(0.0)	0	(0.0)	0.18
Z34_ICD10	378	(0.9)	19	(0.3)	<0.001	262	(0.9)	9	(0.2)	<0.001
Z36_ICD10	82	(0.2)	1	(0.0)	0.002	38	(0.1)	3	(0.1)	0.36
Z39_ICD10	242	(0.6)	10	(0.2)	<0.001	160	(0.6)	8	(0.2)	0.004
Z41_ICD10	326	(0.8)	31	(0.5)	0.05	214	(0.8)	23	(0.6)	0.29
Z43_ICD10	28	(0.1)	5	(0.1)	0.59	32	(0.1)	1	(0.0)	0.11
Z44_ICD10	0	(0.0)	0	(0.0)	1	2	(0.0)	0	(0.0)	0.6
Z45_ICD10	13	(0.0)	2	(0.0)	0.88	7	(0.0)	1	(0.0)	0.96
Z46_ICD10	239	(0.6)	29	(0.5)	0.52	135	(0.5)	22	(0.6)	0.42
Z48_ICD10	80	(0.2)	12	(0.2)	0.78	47	(0.2)	8	(0.2)	0.55
Z49_ICD10	25	(0.1)	4	(0.1)	0.78	10	(0.0)	3	(0.1)	0.22
Z51_ICD10	37	(0.1)	6	(0.1)	0.71	38	(0.1)	5	(0.1)	0.95
Z52_ICD10	30	(0.1)	1	(0.0)	0.13	25	(0.1)	5	(0.1)	0.43
Z55_ICD10	1	(0.0)	0	(0.0)	0.71	0	(0.0)	0	(0.0)	1
Z57_ICD10	1	(0.0)	0	(0.0)	0.71	0	(0.0)	0	(0.0)	1
Z62_ICD10	2	(0.0)	0	(0.0)	0.6	1	(0.0)	0	(0.0)	0.71
Z63_ICD10	7	(0.0)	3	(0.1)	0.08	1	(0.0)	1	(0.0)	0.1
Z64_ICD10	7	(0.0)	2	(0.0)	0.35	10	(0.0)	1	(0.0)	0.77
Z65_ICD10	12	(0.0)	2	(0.0)	0.8	4	(0.0)	1	(0.0)	0.58
Z71_ICD10	2879	(6.8)	422	(7.2)	0.21	1832	(6.5)	274	(7.1)	0.12
Z72_ICD10	7	(0.0)	0	(0.0)	0.33	2	(0.0)	0	(0.0)	0.6
Z76_ICD10	0	(0.0)	0	(0.0)	1	2	(0.0)	0	(0.0)	0.6
Z77_ICD10	0	(0.0)	0	(0.0)	1	1	(0.0)	0	(0.0)	0.71
Z78_ICD10	2	(0.0)	0	(0.0)	0.6	0	(0.0)	0	(0.0)	1

Z79_ICD10	0	(0.0)	0	(0.0)	1	1	(0.0)	0	(0.0)	0.71
Z80_ICD10	45	(0.1)	7	(0.1)	0.76	22	(0.1)	1	(0.0)	0.26
Z82_ICD10	4	(0.0)	2	(0.0)	0.11	8	(0.0)	1	(0.0)	0.94
Z83_ICD10	19	(0.0)	0	(0.0)	0.11	7	(0.0)	0	(0.0)	0.33
Z84_ICD10	2	(0.0)	0	(0.0)	0.6	1	(0.0)	0	(0.0)	0.71
Z85_ICD10	20	(0.0)	1	(0.0)	0.3	7	(0.0)	1	(0.0)	0.96
Z86_ICD10	19	(0.0)	4	(0.1)	0.44	12	(0.0)	2	(0.1)	0.79
Z87_ICD10	17	(0.0)	1	(0.0)	0.39	12	(0.0)	0	(0.0)	0.2
Z88_ICD10	2	(0.0)	0	(0.0)	0.6	2	(0.0)	0	(0.0)	0.6
Z89_ICD10	4	(0.0)	1	(0.0)	0.59	10	(0.0)	1	(0.0)	0.77
Z90_ICD10	12	(0.0)	2	(0.0)	0.8	4	(0.0)	0	(0.0)	0.46
Z91_ICD10	1	(0.0)	0	(0.0)	0.71	1	(0.0)	0	(0.0)	0.71
Z92_ICD10	1	(0.0)	0	(0.0)	0.71	0	(0.0)	0	(0.0)	1
Z93_ICD10	26	(0.1)	1	(0.0)	0.18	12	(0.0)	2	(0.1)	0.79
Z94_ICD10	1208	(2.9)	137	(2.4)	0.03	827	(2.9)	96	(2.5)	0.14
Z95_ICD10	168	(0.4)	34	(0.6)	0.04	102	(0.4)	22	(0.6)	0.05
Z96_ICD10	1375	(3.3)	265	(4.6)	<0.001	919	(3.3)	190	(5.0)	<0.001
Z97_ICD10	7	(0.0)	2	(0.0)	0.35	4	(0.0)	0	(0.0)	0.46
Z98_ICD10	101	(0.2)	20	(0.3)	0.13	80	(0.3)	11	(0.3)	0.97
Z99_ICD10	72	(0.2)	12	(0.2)	0.54	50	(0.2)	5	(0.1)	0.51
Chills	3805	(9.0)	1098	(18.9)	<0.001	2543	(9.0)	744	(19.4)	<0.001
Headache	1874	(4.4)	109	(1.9)	<0.001	1244	(4.4)	80	(2.1)	<0.001
Myalgia	535	(1.3)	49	(0.8)	0.006	371	(1.3)	24	(0.6)	<0.001
Arthralgia	46	(0.1)	3	(0.1)	0.2	33	(0.1)	3	(0.1)	0.5
Pain	9713	(23.0)	1203	(20.7)	<0.001	6402	(22.7)	810	(21.1)	0.03
Weakness	3341	(7.9)	525	(9.0)	0.003	2326	(8.2)	341	(8.9)	0.16
AMS	1028	(2.4)	262	(4.5)	<0.001	669	(2.4)	196	(5.1)	<0.001
Syncope	129	(0.3)	27	(0.5)	0.05	88	(0.3)	8	(0.2)	0.27
Dizziness	1440	(3.4)	183	(3.1)	0.3	967	(3.4)	119	(3.1)	0.31
Seizures	185	(0.4)	22	(0.4)	0.52	118	(0.4)	13	(0.3)	0.47
Motor	97	(0.2)	9	(0.2)	0.25	67	(0.2)	12	(0.3)	0.37
Bulbar	52	(0.1)	8	(0.1)	0.77	34	(0.1)	1	(0.0)	0.1

Rhinorrhea	585	(1.4)	30	(0.5)	<0.001	384	(1.4)	22	(0.6)	<0.001
Cough	6572	(15.5)	323	(5.5)	<0.001	4335	(15.3)	221	(5.8)	<0.001
sputum	1958	(4.6)	137	(2.4)	<0.001	1318	(4.7)	66	(1.7)	<0.001
SOB	5130	(12.1)	685	(11.8)	0.43	3338	(11.8)	420	(11.0)	0.12
ChestPain	553	(1.3)	64	(1.1)	0.19	374	(1.3)	48	(1.3)	0.71
Hoarseness	32	(0.1)	1	(0.0)	0.11	15	(0.1)	1	(0.0)	0.48
SoreThroat	1572	(3.7)	38	(0.7)	<0.001	970	(3.4)	29	(0.8)	<0.001
Hemoptysis	131	(0.3)	6	(0.1)	0.006	103	(0.4)	4	(0.1)	0.009
Palpitations	197	(0.5)	20	(0.3)	0.19	121	(0.4)	13	(0.3)	0.42
Diaphoresis	111	(0.3)	28	(0.5)	0.004	70	(0.2)	17	(0.4)	0.03
Bradycardia	1	(0.0)	1	(0.0)	0.1	8	(0.0)	0	(0.0)	0.3
Choke	62	(0.1)	4	(0.1)	0.13	44	(0.2)	2	(0.1)	0.11
Cyanosis	14	(0.0)	4	(0.1)	0.19	8	(0.0)	0	(0.0)	0.3
Desat	31	(0.1)	5	(0.1)	0.74	25	(0.1)	4	(0.1)	0.76
Supp_O2	294	(0.7)	57	(1.0)	0.02	190	(0.7)	38	(1.0)	0.03
PPV	115	(0.3)	13	(0.2)	0.5	59	(0.2)	14	(0.4)	0.06
Erythema	1189	(2.8)	111	(1.9)	<0.001	829	(2.9)	83	(2.2)	0.007
Swelling	1241	(2.9)	106	(1.8)	<0.001	788	(2.8)	73	(1.9)	0.001
Discharge	139	(0.3)	9	(0.2)	0.02	93	(0.3)	10	(0.3)	0.48
Rash	528	(1.2)	19	(0.3)	<0.001	373	(1.3)	13	(0.3)	<0.001
LUTS	1365	(3.2)	242	(4.2)	<0.001	903	(3.2)	166	(4.3)	<0.001
FlankPain	930	(2.2)	193	(3.3)	<0.001	587	(2.1)	112	(2.9)	<0.001
Oliguria	51	(0.1)	11	(0.2)	0.17	33	(0.1)	9	(0.2)	0.06
anorexia	379	(0.9)	61	(1.0)	0.25	235	(0.8)	49	(1.3)	0.006
NauseaVomit	3716	(8.8)	719	(12.3)	<0.001	2523	(8.9)	477	(12.4)	<0.001
AbdomenDistention	141	(0.3)	28	(0.5)	0.07	104	(0.4)	16	(0.4)	0.64
AbdominalPain	3839	(9.1)	558	(9.6)	0.21	2594	(9.2)	377	(9.8)	0.19
Diarrhea	2369	(5.6)	204	(3.5)	<0.001	1557	(5.5)	138	(3.6)	<0.001
GIB	370	(0.9)	67	(1.2)	0.04	255	(0.9)	31	(0.8)	0.56
Constipation	189	(0.4)	21	(0.4)	0.35	112	(0.4)	18	(0.5)	0.5
Hematemesis	122	(0.3)	22	(0.4)	0.24	81	(0.3)	11	(0.3)	1
Jaundice	141	(0.3)	37	(0.6)	<0.001	83	(0.3)	32	(0.8)	<0.001

Hiccup	13	(0.0)	1	(0.0)	0.57	14	(0.0)	3	(0.1)	0.47
Hyperglycemia	44	(0.1)	9	(0.2)	0.28	21	(0.1)	4	(0.1)	0.53
Hypoglycemia	4	(0.0)	0	(0.0)	0.46	5	(0.0)	0	(0.0)	0.41
HTN	34	(0.1)	7	(0.1)	0.33	31	(0.1)	3	(0.1)	0.57
hoTN	46	(0.1)	20	(0.3)	<0.001	41	(0.1)	10	(0.3)	0.09
Fall	340	(0.8)	47	(0.8)	0.98	215	(0.8)	33	(0.9)	0.51
HTN_PHx	8481	(20.1)	1512	(26.0)	<0.001	5746	(20.3)	1017	(26.5)	<0.001
DM_PHx	5372	(12.7)	1105	(19.0)	<0.001	3669	(13.0)	725	(18.9)	<0.001
CAD_PHx	1684	(4.0)	279	(4.8)	0.003	1087	(3.8)	187	(4.9)	0.002
CVA_PHx	1469	(3.5)	220	(3.8)	0.23	938	(3.3)	137	(3.6)	0.41
BPH_PHx	1480	(3.5)	230	(4.0)	0.08	997	(3.5)	160	(4.2)	0.04
UTI_PHx	1245	(2.9)	232	(4.0)	<0.001	825	(2.9)	148	(3.9)	0.001
CKD_PHx	933	(2.2)	166	(2.9)	0.002	647	(2.3)	135	(3.5)	<0.001
heartDz_PHx	1074	(2.5)	201	(3.5)	<0.001	738	(2.6)	110	(2.9)	0.35
HBV_PHx	1171	(2.8)	190	(3.3)	0.03	732	(2.6)	114	(3.0)	0.17
COPD_PHx	875	(2.1)	54	(0.9)	<0.001	571	(2.0)	52	(1.4)	0.005
dementia_PHx	931	(2.2)	123	(2.1)	0.67	591	(2.1)	89	(2.3)	0.35
asthma_PHx	894	(2.1)	72	(1.2)	<0.001	595	(2.1)	46	(1.2)	<0.001
HLD_PHx	950	(2.2)	199	(3.4)	<0.001	604	(2.1)	110	(2.9)	0.004
pneumonia_PHx	1025	(2.4)	107	(1.8)	0.006	693	(2.5)	73	(1.9)	0.04
lungCA_PHx	1031	(2.4)	118	(2.0)	0.05	685	(2.4)	82	(2.1)	0.28
gout_PHx	797	(1.9)	112	(1.9)	0.84	529	(1.9)	80	(2.1)	0.36
anemia_PHx	902	(2.1)	166	(2.9)	<0.001	609	(2.2)	120	(3.1)	<0.001
CHF_PHx	607	(1.4)	95	(1.6)	0.24	426	(1.5)	74	(1.9)	0.05
liverCirrhosis_PHx	619	(1.5)	200	(3.4)	<0.001	428	(1.5)	126	(3.3)	<0.001
Parkinsonism_PHx	694	(1.6)	86	(1.5)	0.35	446	(1.6)	60	(1.6)	0.95
GU_PHx	727	(1.7)	147	(2.5)	<0.001	448	(1.6)	82	(2.1)	0.01
HCC_PHx	636	(1.5)	197	(3.4)	<0.001	469	(1.7)	110	(2.9)	<0.001
HCV_PHx	556	(1.3)	130	(2.2)	<0.001	389	(1.4)	81	(2.1)	<0.001
arrhythmia_PHx	514	(1.2)	71	(1.2)	0.98	361	(1.3)	47	(1.2)	0.79
lymphoma_PHx	727	(1.7)	98	(1.7)	0.84	496	(1.8)	56	(1.5)	0.19
gallstone_PHx	461	(1.1)	126	(2.2)	<0.001	336	(1.2)	95	(2.5)	<0.001

OA_PHx	409	(1.0)	85	(1.5)	<0.001	279	(1.0)	55	(1.4)	0.01
CVD_PHx	548	(1.3)	82	(1.4)	0.48	335	(1.2)	57	(1.5)	0.11
breastCA_PHx	1025	(2.4)	133	(2.3)	0.52	661	(2.3)	82	(2.1)	0.44
Afib_PHx	356	(0.8)	55	(0.9)	0.42	258	(0.9)	51	(1.3)	0.01
colonCA_PHx	521	(1.2)	124	(2.1)	<0.001	382	(1.4)	73	(1.9)	0.007
depression_PHx	339	(0.8)	48	(0.8)	0.85	255	(0.9)	40	(1.0)	0.39
cellulitis_PHx	329	(0.8)	49	(0.8)	0.61	206	(0.7)	36	(0.9)	0.16
nephrolithiasis_PHx	380	(0.9)	88	(1.5)	<0.001	272	(1.0)	51	(1.3)	0.03
SLE_PHx	351	(0.8)	40	(0.7)	0.26	265	(0.9)	25	(0.7)	0.08
constipation_PHx	223	(0.5)	38	(0.7)	0.22	157	(0.6)	26	(0.7)	0.34
insomnia_PHx	332	(0.8)	51	(0.9)	0.46	238	(0.8)	41	(1.1)	0.16
GERD_PHx	310	(0.7)	54	(0.9)	0.11	204	(0.7)	22	(0.6)	0.3
NPC_PHx	408	(1.0)	44	(0.8)	0.12	262	(0.9)	33	(0.9)	0.69
AML_PHx	372	(0.9)	48	(0.8)	0.67	281	(1.0)	28	(0.7)	0.12
ESRD_PHx	699	(1.7)	166	(2.9)	<0.001	513	(1.8)	117	(3.1)	<0.001
epilepsy_PHx	287	(0.7)	24	(0.4)	0.02	172	(0.6)	16	(0.4)	0.15
prostateCA_PHx	273	(0.6)	57	(1.0)	0.004	176	(0.6)	37	(1.0)	0.01
TB_PHx	391	(0.9)	33	(0.6)	0.006	281	(1.0)	24	(0.6)	0.03
kidneyTx_PHx	475	(1.1)	48	(0.8)	0.04	314	(1.1)	32	(0.8)	0.12
cataract_PHx	295	(0.7)	49	(0.8)	0.22	194	(0.7)	34	(0.9)	0.17
DU_PHx	210	(0.5)	56	(1.0)	<0.001	171	(0.6)	21	(0.5)	0.67
gastritis_PHx	205	(0.5)	40	(0.7)	0.04	159	(0.6)	25	(0.7)	0.49
anxiety_PHx	250	(0.6)	30	(0.5)	0.48	153	(0.5)	21	(0.5)	0.96
hemorrhoid_PHx	253	(0.6)	35	(0.6)	0.98	183	(0.6)	20	(0.5)	0.36
HIV_PHx	241	(0.6)	23	(0.4)	0.09	146	(0.5)	14	(0.4)	0.21
RA_PHx	217	(0.5)	19	(0.3)	0.06	103	(0.4)	23	(0.6)	0.03
hepatitis_PHx	1150	(2.7)	246	(4.2)	<0.001	708	(2.5)	141	(3.7)	<0.001
bronchitis_PHx	220	(0.5)	20	(0.3)	0.07	158	(0.6)	18	(0.5)	0.48
liverTumor_PHx	201	(0.5)	45	(0.8)	0.003	148	(0.5)	22	(0.6)	0.69
pancreaticCA_PHx	233	(0.6)	75	(1.3)	<0.001	149	(0.5)	56	(1.5)	<0.001
refluxEsophagitis_PHx	154	(0.4)	26	(0.4)	0.33	106	(0.4)	24	(0.6)	0.02
gastricCA_PHx	237	(0.6)	61	(1.0)	<0.001	180	(0.6)	42	(1.1)	0.001

URI_PHx	177	(0.4)	19	(0.3)	0.3	120	(0.4)	14	(0.4)	0.59
AMI_PHx	231	(0.5)	37	(0.6)	0.39	178	(0.6)	21	(0.5)	0.54
bladderCA_PHx	185	(0.4)	49	(0.8)	<0.001	135	(0.5)	42	(1.1)	<0.001
hypothyroidism_PHx	140	(0.3)	25	(0.4)	0.23	108	(0.4)	13	(0.3)	0.68
osteoporosis_PHx	148	(0.3)	37	(0.6)	<0.001	83	(0.3)	17	(0.4)	0.12
PAOD_PHx	130	(0.3)	33	(0.6)	0.001	92	(0.3)	25	(0.7)	0.002
neurogenicBladder_PHx	139	(0.3)	27	(0.5)	0.1	93	(0.3)	22	(0.6)	0.02
appendicitis_PHx	168	(0.4)	13	(0.2)	0.04	95	(0.3)	9	(0.2)	0.3
bronchiectasis_PHx	157	(0.4)	8	(0.1)	0.004	106	(0.4)	6	(0.2)	0.03
HZV_PHx	158	(0.4)	31	(0.5)	0.07	123	(0.4)	20	(0.5)	0.45
ICH_PHx	225	(0.5)	25	(0.4)	0.31	126	(0.4)	15	(0.4)	0.63
GIB_PHx	110	(0.3)	27	(0.5)	0.006	80	(0.3)	19	(0.5)	0.03
MM_PHx	162	(0.4)	28	(0.5)	0.26	122	(0.4)	17	(0.4)	0.92
lumbarSpondylosis_PHx	158	(0.4)	23	(0.4)	0.8	80	(0.3)	10	(0.3)	0.81
lungTumor_PHx	162	(0.4)	15	(0.3)	0.14	99	(0.4)	10	(0.3)	0.37
hydronephrosis_PHx	182	(0.4)	28	(0.5)	0.58	110	(0.4)	18	(0.5)	0.46
liverTx_PHx	202	(0.5)	25	(0.4)	0.62	146	(0.5)	20	(0.5)	0.97
APN_PHx	192	(0.5)	21	(0.4)	0.32	108	(0.4)	17	(0.4)	0.57
rectalCA_PHx	178	(0.4)	36	(0.6)	0.03	118	(0.4)	24	(0.6)	0.07
hyperthyroidism_PHx	175	(0.4)	19	(0.3)	0.32	115	(0.4)	5	(0.1)	0.008
chemoRx_PHx	1773	(4.2)	273	(4.7)	0.08	1140	(4.0)	178	(4.6)	0.08
BTI_PHx	137	(0.3)	50	(0.9)	<0.001	87	(0.3)	33	(0.9)	<0.001
goiter_PHx	157	(0.4)	21	(0.4)	0.9	104	(0.4)	13	(0.3)	0.78
glaucoma_PHx	154	(0.4)	29	(0.5)	0.12	103	(0.4)	16	(0.4)	0.61
MDS_PHx	156	(0.4)	21	(0.4)	0.92	118	(0.4)	17	(0.4)	0.82
hernia_PHx	201	(0.5)	28	(0.5)	0.95	134	(0.5)	13	(0.3)	0.24
urolithiasis_PHx	104	(0.2)	23	(0.4)	0.04	75	(0.3)	15	(0.4)	0.17
MVP_PHx	159	(0.4)	12	(0.2)	0.04	140	(0.5)	14	(0.4)	0.27
EV_PHx	82	(0.2)	36	(0.6)	<0.001	59	(0.2)	23	(0.6)	<0.001
uterineMyoma_PHx	181	(0.4)	23	(0.4)	0.72	139	(0.5)	16	(0.4)	0.53
pancreatitis_PHx	154	(0.4)	36	(0.6)	0.004	120	(0.4)	27	(0.7)	0.02
DVT_PHx	110	(0.3)	37	(0.6)	<0.001	79	(0.3)	9	(0.2)	0.62

seizure_PHx	59	(0.1)	13	(0.2)	0.12	55	(0.2)	5	(0.1)	0.39
hyperuricemia_PHx	113	(0.3)	13	(0.2)	0.54	75	(0.3)	15	(0.4)	0.17
cervicalCA_PHx	138	(0.3)	38	(0.7)	<0.001	91	(0.3)	36	(0.9)	<0.001
CesareanSection_PHx	78	(0.2)	8	(0.1)	0.43	64	(0.2)	5	(0.1)	0.23
ileus_PHx	93	(0.2)	14	(0.2)	0.75	75	(0.3)	6	(0.2)	0.21
boneMetastasis_PHx	184	(0.4)	30	(0.5)	0.39	118	(0.4)	24	(0.6)	0.07
jaundice_PHx	97	(0.2)	40	(0.7)	<0.001	41	(0.1)	16	(0.4)	<0.001
cholecystitis_PHx	95	(0.2)	29	(0.5)	<0.001	76	(0.3)	19	(0.5)	0.02
hematuria_PHx	74	(0.2)	19	(0.3)	0.01	64	(0.2)	8	(0.2)	0.83
compressionFx_PHx	90	(0.2)	18	(0.3)	0.15	57	(0.2)	16	(0.4)	0.009
PLE_PHx	109	(0.3)	16	(0.3)	0.81	65	(0.2)	9	(0.2)	0.95
esophCA_PHx	155	(0.4)	17	(0.3)	0.37	94	(0.3)	14	(0.4)	0.74
thrombocytopenia_PHx	106	(0.3)	27	(0.5)	0.004	69	(0.2)	11	(0.3)	0.62
chronicPancreatitis_PHx	75	(0.2)	20	(0.3)	0.007	53	(0.2)	13	(0.3)	0.05
arthritis_PHx	679	(1.6)	109	(1.9)	0.13	431	(1.5)	68	(1.8)	0.24
leukemia_PHx	567	(1.3)	79	(1.4)	0.92	394	(1.4)	38	(1.0)	0.04
chronicPeriodontitis_PHx	92	(0.2)	13	(0.2)	0.93	68	(0.2)	11	(0.3)	0.59
Sjogren_PHx	101	(0.2)	7	(0.1)	0.07	78	(0.3)	4	(0.1)	0.05
allergicRhinitis_PHx	101	(0.2)	9	(0.2)	0.21	58	(0.2)	6	(0.2)	0.53
thalassemia_PHx	105	(0.2)	18	(0.3)	0.39	76	(0.3)	9	(0.2)	0.7
chronicConjunctivitis_PHx	76	(0.2)	18	(0.3)	0.04	58	(0.2)	6	(0.2)	0.53
organicBrain_PHx	80	(0.2)	14	(0.2)	0.41	50	(0.2)	8	(0.2)	0.66
biliaryCA_PHx	56	(0.1)	37	(0.6)	<0.001	46	(0.2)	19	(0.5)	<0.001
colonPolyp_PHx	85	(0.2)	12	(0.2)	0.93	53	(0.2)	7	(0.2)	0.95
kidneyCA_PHx	113	(0.3)	20	(0.3)	0.3	82	(0.3)	14	(0.4)	0.43
AA_PHx	96	(0.2)	15	(0.3)	0.65	78	(0.3)	12	(0.3)	0.68
oralCA_PHx	101	(0.2)	18	(0.3)	0.31	89	(0.3)	12	(0.3)	0.98
choledocholithiasis_PHx	77	(0.2)	34	(0.6)	<0.001	51	(0.2)	14	(0.4)	0.02
respFailure_PHx	78	(0.2)	9	(0.2)	0.62	50	(0.2)	4	(0.1)	0.3
boneMarrowTx_PHx	193	(0.5)	24	(0.4)	0.64	147	(0.5)	17	(0.4)	0.53
dizziness_PHx	58	(0.1)	6	(0.1)	0.5	33	(0.1)	4	(0.1)	0.83
Sicca_PHx	77	(0.2)	11	(0.2)	0.91	53	(0.2)	8	(0.2)	0.78

Psy_PHx	44	(0.1)	3	(0.1)	0.23	39	(0.1)	1	(0.0)	0.07
UGIB_PHx	55	(0.1)	9	(0.2)	0.63	27	(0.1)	3	(0.1)	0.74
PVD_PHx	71	(0.2)	15	(0.3)	0.13	44	(0.2)	12	(0.3)	0.03
pancreaticTumor_PHx	52	(0.1)	28	(0.5)	<0.001	38	(0.1)	14	(0.4)	<0.001
bronchopneumonia_PHx	59	(0.1)	7	(0.1)	0.71	46	(0.2)	3	(0.1)	0.21
VHD_PHx	125	(0.3)	22	(0.4)	0.29	69	(0.2)	17	(0.4)	0.03
tongueCA_PHx	123	(0.3)	13	(0.2)	0.36	77	(0.3)	9	(0.2)	0.67
lungFibrosis_PHx	70	(0.2)	8	(0.1)	0.62	50	(0.2)	1	(0.0)	0.03
hoTN_PHx	65	(0.2)	15	(0.3)	0.07	38	(0.1)	8	(0.2)	0.25
brainTumor_PHx	127	(0.3)	23	(0.4)	0.22	102	(0.4)	10	(0.3)	0.32
HIVD_PHx	86	(0.2)	11	(0.2)	0.82	61	(0.2)	4	(0.1)	0.15
cardiomegaly_PHx	48	(0.1)	6	(0.1)	0.82	44	(0.2)	4	(0.1)	0.44
functionalGI_PHx	64	(0.2)	9	(0.2)	0.95	45	(0.2)	6	(0.2)	0.97
cholangioCA_PHx	86	(0.2)	44	(0.8)	<0.001	53	(0.2)	19	(0.5)	<0.001
pressureUlcer_PHx	73	(0.2)	10	(0.2)	0.99	48	(0.2)	5	(0.1)	0.57
brainMetastasis_PHx	113	(0.3)	13	(0.2)	0.54	66	(0.2)	13	(0.3)	0.22
buccalCA_PHx	71	(0.2)	9	(0.2)	0.82	64	(0.2)	9	(0.2)	0.92
vertigo_PHx	114	(0.3)	14	(0.2)	0.69	59	(0.2)	7	(0.2)	0.74
sinusitis_PHx	93	(0.2)	8	(0.1)	0.2	74	(0.3)	5	(0.1)	0.12
femoralFx_PHx	73	(0.2)	12	(0.2)	0.57	47	(0.2)	8	(0.2)	0.55
polyneuropathy_PHx	61	(0.1)	15	(0.3)	0.04	39	(0.1)	2	(0.1)	0.16
eczema_PHx	59	(0.1)	12	(0.2)	0.21	42	(0.1)	6	(0.2)	0.91
schizophrenia_PHx	104	(0.2)	7	(0.1)	0.06	65	(0.2)	5	(0.1)	0.21
fattyLiver_PHx	72	(0.2)	9	(0.2)	0.78	48	(0.2)	5	(0.1)	0.57
MDD_PHx	36	(0.1)	8	(0.1)	0.22	35	(0.1)	4	(0.1)	0.74
ascites_PHx	55	(0.1)	17	(0.3)	0.003	30	(0.1)	13	(0.3)	<0.001
liverAbscess_PHx	64	(0.2)	18	(0.3)	0.006	39	(0.1)	15	(0.4)	<0.001
AKI_PHx	42	(0.1)	10	(0.2)	0.11	26	(0.1)	9	(0.2)	0.01
vasculitis_PHx	69	(0.2)	9	(0.2)	0.88	53	(0.2)	6	(0.2)	0.67
TIA_PHx	46	(0.1)	12	(0.2)	0.04	35	(0.1)	5	(0.1)	0.91
SSS_PHx	73	(0.2)	7	(0.1)	0.36	47	(0.2)	12	(0.3)	0.05
ovarianCA_PHx	132	(0.3)	26	(0.4)	0.09	81	(0.3)	19	(0.5)	0.03

thyroidCA_PHx	61	(0.1)	8	(0.1)	0.9	32	(0.1)	9	(0.2)	0.05
MR_PHx	49	(0.1)	10	(0.2)	0.25	47	(0.2)	4	(0.1)	0.37
cervicalSpondylosis_PHx	57	(0.1)	12	(0.2)	0.18	30	(0.1)	4	(0.1)	0.97
cachexia_PHx	35	(0.1)	11	(0.2)	0.01	24	(0.1)	7	(0.2)	0.07
SDH_PHx	65	(0.2)	6	(0.1)	0.35	32	(0.1)	9	(0.2)	0.05
dermatitis_PHx	78	(0.2)	11	(0.2)	0.94	59	(0.2)	5	(0.1)	0.31
pacemaker_PHx	150	(0.4)	20	(0.3)	0.89	117	(0.4)	13	(0.3)	0.49
CML_PHx	64	(0.2)	9	(0.2)	0.95	39	(0.1)	1	(0.0)	0.07
autoimmune_PHx	62	(0.1)	11	(0.2)	0.44	50	(0.2)	7	(0.2)	0.94
aspirationPNA_PHx	52	(0.1)	4	(0.1)	0.26	43	(0.2)	1	(0.0)	0.05
radiculopathy_PHx	38	(0.1)	2	(0.0)	0.17	21	(0.1)	8	(0.2)	0.009
heartTx_PHx	65	(0.2)	0	(0.0)	0.003	51	(0.2)	3	(0.1)	0.15
pseudophakia_PHx	46	(0.1)	5	(0.1)	0.62	26	(0.1)	4	(0.1)	0.81
renalDz_PHx	57	(0.1)	7	(0.1)	0.78	37	(0.1)	6	(0.2)	0.68
Alzheimer_PHx	57	(0.1)	10	(0.2)	0.48	40	(0.1)	10	(0.3)	0.08
abdominalPain_PHx	36	(0.1)	9	(0.2)	0.1	19	(0.1)	4	(0.1)	0.42
hypopharynxCA_PHx	84	(0.2)	14	(0.2)	0.51	48	(0.2)	9	(0.2)	0.37
breastTumor_PHx	69	(0.2)	10	(0.2)	0.88	55	(0.2)	5	(0.1)	0.39
dysphagia_PHx	50	(0.1)	6	(0.1)	0.75	31	(0.1)	3	(0.1)	0.57
myofascialPain_PHx	57	(0.1)	8	(0.1)	0.96	28	(0.1)	7	(0.2)	0.14
MG_PHx	33	(0.1)	9	(0.2)	0.06	32	(0.1)	6	(0.2)	0.47
AFL_PHx	45	(0.1)	4	(0.1)	0.4	25	(0.1)	6	(0.2)	0.2
edema_PHx	29	(0.1)	5	(0.1)	0.64	14	(0.0)	4	(0.1)	0.18
spineOP_PHx	41	(0.1)	8	(0.1)	0.36	24	(0.1)	6	(0.2)	0.17
oropharynxCA_PHx	51	(0.1)	6	(0.1)	0.72	25	(0.1)	4	(0.1)	0.76
osteomyelitis_PHx	29	(0.1)	6	(0.1)	0.36	23	(0.1)	2	(0.1)	0.54
sleep_PHx	38	(0.1)	3	(0.1)	0.35	36	(0.1)	5	(0.1)	0.96
SNHL_PHx	42	(0.1)	5	(0.1)	0.76	31	(0.1)	2	(0.1)	0.3
pulmEdema_PHx	33	(0.1)	4	(0.1)	0.81	25	(0.1)	1	(0.0)	0.2
urticaria_PHx	43	(0.1)	6	(0.1)	0.97	47	(0.2)	3	(0.1)	0.19
radioRx_PHx	276	(0.7)	37	(0.6)	0.88	203	(0.7)	35	(0.9)	0.19
tinea_PHx	50	(0.1)	2	(0.0)	0.07	42	(0.1)	2	(0.1)	0.13

abnlLFTs_PHx	52	(0.1)	10	(0.2)	0.33	35	(0.1)	5	(0.1)	0.91
endometrialCA_PHx	65	(0.2)	16	(0.3)	0.03	57	(0.2)	13	(0.3)	0.09
liverMetastasis_PHx	104	(0.2)	21	(0.4)	0.11	73	(0.3)	15	(0.4)	0.14
labyrintheInsufficiency_PHx	46	(0.1)	4	(0.1)	0.37	28	(0.1)	7	(0.2)	0.14
pancytopenia_PHx	42	(0.1)	2	(0.0)	0.12	31	(0.1)	8	(0.2)	0.1
neckMass_PHx	40	(0.1)	6	(0.1)	0.84	23	(0.1)	3	(0.1)	0.95
hyperparathyroidism_PHx	17	(0.0)	5	(0.1)	0.13	18	(0.1)	2	(0.1)	0.79
hypoK_PHx	24	(0.1)	8	(0.1)	0.03	21	(0.1)	0	(0.0)	0.09
AoS_PHx	41	(0.1)	7	(0.1)	0.6	18	(0.1)	4	(0.1)	0.37
oralUlcer_PHx	36	(0.1)	4	(0.1)	0.68	17	(0.1)	6	(0.2)	0.04
bipolar_PHx	54	(0.1)	2	(0.0)	0.05	38	(0.1)	1	(0.0)	0.07
hydrocephalus_PHx	72	(0.2)	9	(0.2)	0.78	39	(0.1)	2	(0.1)	0.16
renalTumor_PHx	48	(0.1)	12	(0.2)	0.06	25	(0.1)	5	(0.1)	0.43
G6PD_PHx	53	(0.1)	3	(0.1)	0.12	32	(0.1)	2	(0.1)	0.28
adrenalInsufficiency_PHx	40	(0.1)	2	(0.0)	0.14	28	(0.1)	4	(0.1)	0.92
cholecystectomy_PHx	42	(0.1)	14	(0.2)	0.003	36	(0.1)	9	(0.2)	0.1
Fever_PHx	50	(0.1)	5	(0.1)	0.49	22	(0.1)	2	(0.1)	0.58
emphysema_PHx	29	(0.1)	0	(0.0)	0.05	27	(0.1)	1	(0.0)	0.17
urineRetention_PHx	27	(0.1)	5	(0.1)	0.54	19	(0.1)	4	(0.1)	0.42
delirium_PHx	38	(0.1)	6	(0.1)	0.75	28	(0.1)	4	(0.1)	0.92
liverCyst_PHx	43	(0.1)	7	(0.1)	0.68	20	(0.1)	3	(0.1)	0.87
CP_PHx	35	(0.1)	2	(0.0)	0.21	20	(0.1)	0	(0.0)	0.1
chronicGN_PHx	43	(0.1)	8	(0.1)	0.43	22	(0.1)	3	(0.1)	0.99
spinalStenosis_PHx	21	(0.0)	4	(0.1)	0.55	9	(0.0)	5	(0.1)	0.006
trachealCA_PHx	61	(0.1)	6	(0.1)	0.43	28	(0.1)	3	(0.1)	0.7
gallbladderCA_PHx	25	(0.1)	10	(0.2)	0.003	26	(0.1)	10	(0.3)	0.003
HSV_PHx	39	(0.1)	6	(0.1)	0.8	37	(0.1)	4	(0.1)	0.67
alcoholicLiverCirrhosis_PHx	26	(0.1)	11	(0.2)	0.001	15	(0.1)	7	(0.2)	0.004
carotidStenosis_PHx	36	(0.1)	4	(0.1)	0.68	27	(0.1)	5	(0.1)	0.52
pulmHTN_PHx	33	(0.1)	4	(0.1)	0.81	22	(0.1)	6	(0.2)	0.12
nephroticSyndrome_PHx	38	(0.1)	7	(0.1)	0.48	22	(0.1)	6	(0.2)	0.12
PSA_PHx	35	(0.1)	12	(0.2)	0.005	18	(0.1)	4	(0.1)	0.37

backPain_PHx	40	(0.1)	3	(0.1)	0.3	16	(0.1)	3	(0.1)	0.61
UC_PHx	41	(0.1)	4	(0.1)	0.51	26	(0.1)	3	(0.1)	0.79
AoA_PHx	33	(0.1)	14	(0.2)	<0.001	25	(0.1)	8	(0.2)	0.03
Fx_PHx	327	(0.8)	57	(1.0)	0.1	216	(0.8)	41	(1.1)	0.05
PSVT_PHx	44	(0.1)	4	(0.1)	0.42	29	(0.1)	5	(0.1)	0.62
psoriasis_PHx	34	(0.1)	3	(0.1)	0.46	30	(0.1)	7	(0.2)	0.19
chestPain_PHx	20	(0.0)	6	(0.1)	0.09	10	(0.0)	2	(0.1)	0.61
headache_PHx	28	(0.1)	5	(0.1)	0.59	14	(0.0)	2	(0.1)	0.95
larynxCA_PHx	39	(0.1)	5	(0.1)	0.88	32	(0.1)	3	(0.1)	0.54
splenomegaly_PHx	24	(0.1)	11	(0.2)	<0.001	19	(0.1)	6	(0.2)	0.06
colonTumor_PHx	38	(0.1)	5	(0.1)	0.92	24	(0.1)	6	(0.2)	0.17
hyperK_PHx	38	(0.1)	3	(0.1)	0.35	11	(0.0)	5	(0.1)	0.02
abscess_PHx	184	(0.4)	38	(0.7)	0.02	121	(0.4)	37	(1.0)	<0.001
PE_PHx	30	(0.1)	2	(0.0)	0.31	17	(0.1)	2	(0.1)	0.85
AGE_PHx	27	(0.1)	7	(0.1)	0.13	19	(0.1)	0	(0.0)	0.11
myeloproliferativeDz_PHx	28	(0.1)	0	(0.0)	0.05	18	(0.1)	4	(0.1)	0.37
palpitations_PHx	25	(0.1)	1	(0.0)	0.2	17	(0.1)	2	(0.1)	0.85
PID_PHx	983	(2.3)	205	(3.5)	<0.001	625	(2.2)	111	(2.9)	0.008
aneurysm_PHx	76	(0.2)	15	(0.3)	0.2	47	(0.2)	14	(0.4)	0.008
hysterectomy_PHx	39	(0.1)	3	(0.1)	0.32	20	(0.1)	3	(0.1)	0.87
Graves_PHx	53	(0.1)	5	(0.1)	0.42	29	(0.1)	3	(0.1)	0.65
dentalCaries_PHx	26	(0.1)	4	(0.1)	0.84	18	(0.1)	1	(0.0)	0.37
AoD_PHx	39	(0.1)	8	(0.1)	0.3	32	(0.1)	6	(0.2)	0.47
proteinuria_PHx	26	(0.1)	3	(0.1)	0.77	15	(0.1)	2	(0.1)	0.98
hepaticComa_PHx	9	(0.0)	11	(0.2)	<0.001	11	(0.0)	4	(0.1)	0.08
hemoptysis_PHx	22	(0.1)	0	(0.0)	0.08	13	(0.0)	2	(0.1)	0.87
AS_PHx	36	(0.1)	2	(0.0)	0.2	12	(0.0)	4	(0.1)	0.11
biliaryAtresia_PHx	21	(0.0)	5	(0.1)	0.26	12	(0.0)	3	(0.1)	0.34
tonsillitis_PHx	47	(0.1)	2	(0.0)	0.09	31	(0.1)	2	(0.1)	0.3
biliaryCirrhosis_PHx	21	(0.0)	10	(0.2)	<0.001	19	(0.1)	2	(0.1)	0.73
hypoNa_PHx	36	(0.1)	4	(0.1)	0.68	15	(0.1)	4	(0.1)	0.22
TKR_PHx	55	(0.1)	15	(0.3)	0.02	48	(0.2)	12	(0.3)	0.05

NTM_PHx	33	(0.1)	0	(0.0)	0.03	19	(0.1)	2	(0.1)	0.73
Crohn_PHx	33	(0.1)	8	(0.1)	0.15	28	(0.1)	1	(0.0)	0.16
dryEye_PHx	27	(0.1)	5	(0.1)	0.54	25	(0.1)	2	(0.1)	0.47
bacteremia_PHx	19	(0.0)	11	(0.2)	<0.001	8	(0.0)	4	(0.1)	0.02
transplant_PHx	906	(2.1)	97	(1.7)	0.02	649	(2.3)	68	(1.8)	0.04
hemolyticAnemia_PHx	34	(0.1)	6	(0.1)	0.57	39	(0.1)	6	(0.2)	0.77
neuropathy_PHx	104	(0.2)	26	(0.4)	0.006	75	(0.3)	11	(0.3)	0.81
AVshunt_PHx	344	(0.8)	84	(1.4)	<0.001	278	(1.0)	47	(1.2)	0.16
meningitis_PHx	42	(0.1)	6	(0.1)	0.93	32	(0.1)	2	(0.1)	0.28
thyroidTumor_PHx	32	(0.1)	5	(0.1)	0.79	19	(0.1)	2	(0.1)	0.73
sepsis_PHx	38	(0.1)	11	(0.2)	0.03	28	(0.1)	9	(0.2)	0.02
MS_PHx	21	(0.0)	5	(0.1)	0.26	17	(0.1)	1	(0.0)	0.4
diarrhea_PHx	23	(0.1)	3	(0.1)	0.93	21	(0.1)	5	(0.1)	0.25
AVB_PHx	25	(0.1)	6	(0.1)	0.22	13	(0.0)	4	(0.1)	0.14
PCKD_PHx	42	(0.1)	10	(0.2)	0.11	17	(0.1)	8	(0.2)	0.002
PTX_PHx	51	(0.1)	5	(0.1)	0.47	33	(0.1)	2	(0.1)	0.26
conjunctivitis_PHx	100	(0.2)	21	(0.4)	0.08	84	(0.3)	8	(0.2)	0.34
adenomyosis_PHx	30	(0.1)	3	(0.1)	0.6	23	(0.1)	2	(0.1)	0.54
ureterCA_PHx	25	(0.1)	2	(0.0)	0.45	25	(0.1)	2	(0.1)	0.47
pelvicTumor_PHx	25	(0.1)	7	(0.1)	0.09	15	(0.1)	1	(0.0)	0.48
chronicGingivitis_PHx	19	(0.0)	2	(0.0)	0.72	17	(0.1)	1	(0.0)	0.4
ITP_PHx	33	(0.1)	7	(0.1)	0.29	26	(0.1)	6	(0.2)	0.24
MSA_PHx	37	(0.1)	3	(0.1)	0.37	14	(0.0)	0	(0.0)	0.17
CervicalCIN_PHx	8	(0.0)	2	(0.0)	0.44	14	(0.0)	1	(0.0)	0.53
thyroiditis_PHx	36	(0.1)	4	(0.1)	0.68	18	(0.1)	4	(0.1)	0.37
pneumoconiosis_PHx	28	(0.1)	4	(0.1)	0.94	14	(0.0)	0	(0.0)	0.17
tonsillarCA_PHx	41	(0.1)	7	(0.1)	0.6	22	(0.1)	1	(0.0)	0.26
dermatomyositis_PHx	30	(0.1)	3	(0.1)	0.6	13	(0.0)	1	(0.0)	0.58
macularLesion_PHx	28	(0.1)	11	(0.2)	0.002	25	(0.1)	5	(0.1)	0.43
ovarianTumor_PHx	48	(0.1)	6	(0.1)	0.82	25	(0.1)	4	(0.1)	0.76
GvHD_PHx	24	(0.1)	4	(0.1)	0.72	18	(0.1)	1	(0.0)	0.37
leukopenia_PHx	20	(0.0)	5	(0.1)	0.23	10	(0.0)	0	(0.0)	0.24

ovarianCyst_PHx	28	(0.1)	4	(0.1)	0.94	17	(0.1)	2	(0.1)	0.85
LAP_PHx	26	(0.1)	2	(0.0)	0.42	17	(0.1)	1	(0.0)	0.4
ventilator_PHx	34	(0.1)	4	(0.1)	0.77	17	(0.1)	1	(0.0)	0.4
bladderTumor_PHx	24	(0.1)	4	(0.1)	0.72	26	(0.1)	3	(0.1)	0.79
paronychia_PHx	21	(0.0)	1	(0.0)	0.28	12	(0.0)	2	(0.1)	0.79
folliculitis_PHx	23	(0.1)	1	(0.0)	0.23	12	(0.0)	1	(0.0)	0.64
meningioma_PHx	18	(0.0)	6	(0.1)	0.05	12	(0.0)	2	(0.1)	0.79
AIDS_PHx	32	(0.1)	2	(0.0)	0.27	35	(0.1)	3	(0.1)	0.44
esophagitis_PHx	178	(0.4)	28	(0.5)	0.51	119	(0.4)	22	(0.6)	0.18
panic_PHx	24	(0.1)	1	(0.0)	0.21	22	(0.1)	2	(0.1)	0.58
carpelTunnel_PHx	16	(0.0)	1	(0.0)	0.43	13	(0.0)	0	(0.0)	0.18
retainedDentRoot_PHx	14	(0.0)	3	(0.1)	0.48	11	(0.0)	1	(0.0)	0.7
leukocytosis_PHx	14	(0.0)	2	(0.0)	0.96	8	(0.0)	3	(0.1)	0.12
spondylolisthesis_PHx	20	(0.0)	3	(0.1)	0.89	13	(0.0)	3	(0.1)	0.4
tracheostomy_PHx	118	(0.3)	15	(0.3)	0.77	67	(0.2)	7	(0.2)	0.51
gingivaCA_PHx	19	(0.0)	1	(0.0)	0.33	17	(0.1)	1	(0.0)	0.4
VBI_PHx	14	(0.0)	0	(0.0)	0.17	14	(0.0)	3	(0.1)	0.47
OAB_PHx	21	(0.0)	1	(0.0)	0.28	14	(0.0)	3	(0.1)	0.47
adrenalTumor_PHx	25	(0.1)	1	(0.0)	0.2	13	(0.0)	3	(0.1)	0.4
perianalAbscess_PHx	12	(0.0)	0	(0.0)	0.2	13	(0.0)	5	(0.1)	0.04
Flu_PHx	24	(0.1)	3	(0.1)	0.87	16	(0.1)	3	(0.1)	0.61
BWL_PHx	20	(0.0)	2	(0.0)	0.67	9	(0.0)	1	(0.0)	0.85
cough_PHx	15	(0.0)	3	(0.1)	0.55	15	(0.1)	1	(0.0)	0.48
neuralgia_PHx	29	(0.1)	7	(0.1)	0.18	16	(0.1)	3	(0.1)	0.61
gallbladderPolyp_PHx	24	(0.1)	4	(0.1)	0.72	15	(0.1)	2	(0.1)	0.98
vaginitis_PHx	23	(0.1)	5	(0.1)	0.35	19	(0.1)	4	(0.1)	0.42
spondylosis_PHx	220	(0.5)	38	(0.7)	0.19	113	(0.4)	17	(0.4)	0.69
pruritus_PHx	21	(0.0)	2	(0.0)	0.62	14	(0.0)	1	(0.0)	0.53
encephalopathy_PHx	19	(0.0)	11	(0.2)	<0.001	31	(0.1)	4	(0.1)	0.92
hemangioma_PHx	33	(0.1)	3	(0.1)	0.49	26	(0.1)	0	(0.0)	0.06
acuteBronchiolitis_PHx	17	(0.0)	0	(0.0)	0.13	5	(0.0)	0	(0.0)	0.41
syphilis_PHx	21	(0.0)	2	(0.0)	0.62	17	(0.1)	0	(0.0)	0.13

softTissueTumor_PHx	19	(0.0)	1	(0.0)	0.33	5	(0.0)	1	(0.0)	0.72
boneTumor_PHx	13	(0.0)	5	(0.1)	0.04	10	(0.0)	0	(0.0)	0.24
ILD_PHx	13	(0.0)	3	(0.1)	0.41	9	(0.0)	0	(0.0)	0.27
HPylori_PHx	13	(0.0)	2	(0.0)	0.88	15	(0.1)	3	(0.1)	0.54
hyperCa_PHx	9	(0.0)	0	(0.0)	0.27	8	(0.0)	1	(0.0)	0.94
ureteralStricture_PHx	21	(0.0)	1	(0.0)	0.28	4	(0.0)	3	(0.1)	0.01
SCI_PHx	38	(0.1)	3	(0.1)	0.35	21	(0.1)	0	(0.0)	0.09
seronegativeArthritis_PHx	14	(0.0)	1	(0.0)	0.52	10	(0.0)	2	(0.1)	0.61
menopause_PHx	24	(0.1)	1	(0.0)	0.21	17	(0.1)	1	(0.0)	0.4
vocalCordPalsy_PHx	7	(0.0)	1	(0.0)	0.97	9	(0.0)	1	(0.0)	0.85
eyeStrain_PHx	10	(0.0)	6	(0.1)	0.002	6	(0.0)	2	(0.1)	0.25
epididymitis_PHx	11	(0.0)	2	(0.0)	0.72	11	(0.0)	1	(0.0)	0.7
chronicPharyngitis_PHx	18	(0.0)	0	(0.0)	0.12	11	(0.0)	0	(0.0)	0.22
headInjury_PHx	13	(0.0)	3	(0.1)	0.41	12	(0.0)	2	(0.1)	0.79
dyspnea_PHx	13	(0.0)	1	(0.0)	0.57	7	(0.0)	0	(0.0)	0.33
osteopenia_PHx	9	(0.0)	3	(0.1)	0.17	10	(0.0)	1	(0.0)	0.77
polymyositis_PHx	15	(0.0)	1	(0.0)	0.47	8	(0.0)	2	(0.1)	0.43
pituitaryTumor_PHx	19	(0.0)	2	(0.0)	0.72	14	(0.0)	0	(0.0)	0.17
lungAbscess_PHx	21	(0.0)	2	(0.0)	0.62	9	(0.0)	1	(0.0)	0.85
DNI_PHx	9	(0.0)	2	(0.0)	0.54	7	(0.0)	1	(0.0)	0.96
VH_PHx	12	(0.0)	4	(0.1)	0.11	6	(0.0)	1	(0.0)	0.85
majorTrauma_PHx	23	(0.1)	1	(0.0)	0.23	11	(0.0)	1	(0.0)	0.7
oralCandidiasis_PHx	9	(0.0)	3	(0.1)	0.17	11	(0.0)	2	(0.1)	0.7
obesity_PHx	19	(0.0)	4	(0.1)	0.44	11	(0.0)	2	(0.1)	0.7
renalCyst_PHx	24	(0.1)	3	(0.1)	0.87	12	(0.0)	3	(0.1)	0.34
THR_PHx	8	(0.0)	3	(0.1)	0.12	6	(0.0)	1	(0.0)	0.85
thymicCA_PHx	20	(0.0)	0	(0.0)	0.1	5	(0.0)	4	(0.1)	0.003
humerusFx_PHx	17	(0.0)	1	(0.0)	0.39	6	(0.0)	0	(0.0)	0.37
hypopituitarism_PHx	17	(0.0)	0	(0.0)	0.13	14	(0.0)	0	(0.0)	0.17
essentialTremor_PHx	8	(0.0)	1	(0.0)	0.93	9	(0.0)	1	(0.0)	0.85
peritonitis_PHx	40	(0.1)	8	(0.1)	0.33	28	(0.1)	4	(0.1)	0.92
PBSCT_PHx	21	(0.0)	3	(0.1)	0.95	17	(0.1)	0	(0.0)	0.13

scoliosis_PHx	20	(0.0)	0	(0.0)	0.1	6	(0.0)	1	(0.0)	0.85
PPU_PHx	40	(0.1)	12	(0.2)	0.02	28	(0.1)	4	(0.1)	0.92
DCM_PHx	19	(0.0)	3	(0.1)	0.82	10	(0.0)	0	(0.0)	0.24
scabies_PHx	9	(0.0)	2	(0.0)	0.54	12	(0.0)	1	(0.0)	0.64
AR_PHx	22	(0.1)	2	(0.0)	0.57	13	(0.0)	1	(0.0)	0.58
cornealErosion_PHx	9	(0.0)	1	(0.0)	0.84	7	(0.0)	0	(0.0)	0.33
pemphigoid_PHx	13	(0.0)	1	(0.0)	0.57	10	(0.0)	2	(0.1)	0.61
gastricTumor_PHx	11	(0.0)	3	(0.1)	0.28	13	(0.0)	2	(0.1)	0.87
OSA_PHx	9	(0.0)	0	(0.0)	0.27	10	(0.0)	3	(0.1)	0.22
candidiasis_PHx	35	(0.1)	11	(0.2)	0.01	28	(0.1)	5	(0.1)	0.57
GV_PHx	14	(0.0)	8	(0.1)	<0.001	8	(0.0)	2	(0.1)	0.43
NF_PHx	11	(0.0)	0	(0.0)	0.22	6	(0.0)	1	(0.0)	0.85
macrocyticAnemia_PHx	14	(0.0)	1	(0.0)	0.52	9	(0.0)	2	(0.1)	0.52
septicArthritis_PHx	10	(0.0)	3	(0.1)	0.22	4	(0.0)	0	(0.0)	0.46
flankPain_PHx	5	(0.0)	1	(0.0)	0.73	7	(0.0)	0	(0.0)	0.33
CLL_PHx	19	(0.0)	3	(0.1)	0.82	13	(0.0)	2	(0.1)	0.87
migraine_PHx	15	(0.0)	1	(0.0)	0.47	11	(0.0)	1	(0.0)	0.7
infertility_PHx	16	(0.0)	0	(0.0)	0.14	10	(0.0)	0	(0.0)	0.24
hemodialysis_PHx	648	(1.5)	173	(3.0)	<0.001	488	(1.7)	120	(3.1)	<0.001
brainCA_PHx	9	(0.0)	3	(0.1)	0.17	6	(0.0)	0	(0.0)	0.37
empyema_PHx	11	(0.0)	2	(0.0)	0.72	10	(0.0)	1	(0.0)	0.77
polio_PHx	16	(0.0)	1	(0.0)	0.43	9	(0.0)	0	(0.0)	0.27
vulvitis_PHx	10	(0.0)	3	(0.1)	0.22	8	(0.0)	3	(0.1)	0.12
MUP_PHx	10	(0.0)	2	(0.0)	0.63	4	(0.0)	1	(0.0)	0.58
IE_PHx	16	(0.0)	6	(0.1)	0.03	7	(0.0)	5	(0.1)	0.002
skinTumor_PHx	6	(0.0)	4	(0.1)	0.007	3	(0.0)	2	(0.1)	0.05
lungMetastasis_PHx	82	(0.2)	8	(0.1)	0.35	56	(0.2)	13	(0.3)	0.08
thymicTumor_PHx	22	(0.1)	2	(0.0)	0.57	13	(0.0)	1	(0.0)	0.58
incontinence_PHx	30	(0.1)	3	(0.1)	0.6	20	(0.1)	2	(0.1)	0.68
IBS_PHx	13	(0.0)	3	(0.1)	0.41	10	(0.0)	1	(0.0)	0.77
urineFrequency_PHx	7	(0.0)	1	(0.0)	0.97	3	(0.0)	0	(0.0)	0.52
dermatophytosis_PHx	13	(0.0)	0	(0.0)	0.18	3	(0.0)	1	(0.0)	0.42

analFistula_PHx	33	(0.1)	3	(0.1)	0.49	14	(0.0)	0	(0.0)	0.17
SAH_PHx	33	(0.1)	3	(0.1)	0.49	12	(0.0)	4	(0.1)	0.11
CMV_PHx	12	(0.0)	3	(0.1)	0.35	10	(0.0)	0	(0.0)	0.24
VSD_PHx	11	(0.0)	2	(0.0)	0.72	11	(0.0)	3	(0.1)	0.27
prostateTumor_PHx	6	(0.0)	2	(0.0)	0.26	11	(0.0)	2	(0.1)	0.7
floater_PHx	10	(0.0)	2	(0.0)	0.63	6	(0.0)	1	(0.0)	0.85
frozenShoulder_PHx	10	(0.0)	1	(0.0)	0.76	5	(0.0)	0	(0.0)	0.41
bedRidden_PHx	24	(0.1)	0	(0.0)	0.07	15	(0.1)	3	(0.1)	0.54
liverDz_PHx	18	(0.0)	7	(0.1)	0.01	16	(0.1)	4	(0.1)	0.27
fibromyalgia_PHx	9	(0.0)	2	(0.0)	0.54	5	(0.0)	0	(0.0)	0.41
GIST_PHx	16	(0.0)	2	(0.0)	0.9	5	(0.0)	4	(0.1)	0.003
otitis_PHx	27	(0.1)	6	(0.1)	0.28	18	(0.1)	0	(0.0)	0.12
parotidTumor_PHx	9	(0.0)	3	(0.1)	0.17	3	(0.0)	0	(0.0)	0.52
UterineCA_PHx	15	(0.0)	0	(0.0)	0.15	5	(0.0)	2	(0.1)	0.18
spineFx_PHx	7	(0.0)	3	(0.1)	0.08	6	(0.0)	1	(0.0)	0.85
coagulopathy_PHx	7	(0.0)	0	(0.0)	0.33	6	(0.0)	0	(0.0)	0.37
duodenumCA_PHx	10	(0.0)	2	(0.0)	0.63	7	(0.0)	5	(0.1)	0.002
hemochromatosis_PHx	5	(0.0)	1	(0.0)	0.73	3	(0.0)	1	(0.0)	0.42
PD_PHx	159	(0.4)	22	(0.4)	0.98	116	(0.4)	12	(0.3)	0.37
endometriosis_PHx	24	(0.1)	1	(0.0)	0.21	16	(0.1)	0	(0.0)	0.14
neutropenia_PHx	8	(0.0)	0	(0.0)	0.29	7	(0.0)	1	(0.0)	0.96
ocularHTN_PHx	7	(0.0)	3	(0.1)	0.08	6	(0.0)	1	(0.0)	0.85
alcoholism_PHx	57	(0.1)	20	(0.3)	<0.001	26	(0.1)	12	(0.3)	<0.001
ALL_PHx	12	(0.0)	2	(0.0)	0.8	6	(0.0)	3	(0.1)	0.05
cystocele_PHx	6	(0.0)	4	(0.1)	0.007	6	(0.0)	2	(0.1)	0.25
PVC_PHx	11	(0.0)	0	(0.0)	0.22	3	(0.0)	1	(0.0)	0.42
renalAbscess_PHx	12	(0.0)	0	(0.0)	0.2	8	(0.0)	1	(0.0)	0.94
dystonia_PHx	12	(0.0)	2	(0.0)	0.8	4	(0.0)	0	(0.0)	0.46
enteritis_PHx	56	(0.1)	10	(0.2)	0.45	33	(0.1)	1	(0.0)	0.11
ALS_PHx	9	(0.0)	2	(0.0)	0.54	7	(0.0)	0	(0.0)	0.33
thrombocytosis_PHx	11	(0.0)	1	(0.0)	0.69	3	(0.0)	1	(0.0)	0.42
syncope_PHx	8	(0.0)	3	(0.1)	0.12	8	(0.0)	2	(0.1)	0.43

pleurisy_PHx	8	(0.0)	0	(0.0)	0.29	6	(0.0)	0	(0.0)	0.37
adjustmentDz_PHx	9	(0.0)	0	(0.0)	0.27	7	(0.0)	2	(0.1)	0.34
tinnitus_PHx	11	(0.0)	0	(0.0)	0.22	9	(0.0)	0	(0.0)	0.27
pregnancy_PHx	23	(0.1)	5	(0.1)	0.35	20	(0.1)	0	(0.0)	0.1
aphasia_PHx	11	(0.0)	0	(0.0)	0.22	2	(0.0)	0	(0.0)	0.6
ankleFx_PHx	10	(0.0)	2	(0.0)	0.63	4	(0.0)	1	(0.0)	0.58
hyperglycemia_PHx	7	(0.0)	2	(0.0)	0.35	7	(0.0)	1	(0.0)	0.96
CCRT_PHx	80	(0.2)	14	(0.2)	0.41	47	(0.2)	5	(0.1)	0.6

Abbreviations: ICD-10, International Statistical Classification of Diseases and Related Health Problems 10th Revision; PHx, past history; EMS, Emergency medical service; BP, Blood pressure; BMI, Body Mass Index; GCS, Glasgow Coma Scale; AMS, Altered mental status; SOB, Short of breath; Desat. Desaturation; Supp_O2; Oxygen supplement; PPV, Positive pressure ventilation; GIB, Gastrointestinal bleeding; HTN, Hypertension; HoTN, Hypotension; DM, Diabetes mellitus; CAD, Coronary artery disease; CVA, Cerebrovascular disease; BPH, Benign prostatic hyperplasia; UTI, Urinary tract infection; CKD, Chronic kidney disease; HeartDz, Heaert Disease; HBV, Hepatitis B virus; COPD, Chronic Obstruction Pulmonary Disease; HLD, Hyperlipidemia; LungCA, Lung cancer; GU, Gastric ulcer; HCC, Hepatocellular carcinoma; HCV, Hepatitis C virus; OA, Osteoarthritis; CVD, Cardiovascular disease; breastCA, Breast cancer; Afib, Atrial fibrilattion; ColonCA, Colon cancer; SLE, Systemic Lupus Erythematosus; GERD, Gastroesophageal reflux disease; NPC, Nasopharyngeal carcinoma; AML, Acute myeloid leukemia; ESRD, End-Stage Renal Disease; prostateCA, Prostate cancer; TB, Tuberculosis; kidneyTx, Kidney transplant; DU, Duodenal ulcer; HIV, Human Immunodeficiency Virus; RA, Rheumatoid arthritis; PancreaticCA, Pancreatic cancer; gastricCA, Gastric cancer; URI, Upper respiratory tract infection; AMI, Acute myocardial infarction; bladderCA, Bladder cancer; PAOD, Peripheral Arterial Occlusion Disease; HZV, Herpes zoster virus; ICH, Intracranial hemorrhage; GIB, Gastrointestinal bleeding; MM, Multiple myeloma; liverTx, Liver transplant; APN, Acute pyelonephritis; rectalCA, Rectal cancer; chemoRx, chemotherapy; BTI, Biliary tract infection; MDS, Myelodysplastic Syndromes; MVP, Mitral valve prolapse; EV, Esophageal varices; DVT, Deep vein thrombosis; cervicalCA, Cervical cancer; compressionFx, Compression fracture; PLE, pleural effusion; esophCA, Esophageal cancer; biliaryCA, Billiary cancer; KidneyCA, Kidney cancer; AA, Aplastic anemia; respFailure, Respiratory failure; boneMarrowTx, Bone marrow transplant; Psy, Psychiatric disease; UGIB, Upper gastrointestinal bleeding; PVD, Peripheral vascular disease; VHD, Valvular heart disease; tongueCA, Tongue cancer; HIVD, Herniation of intervertebral disc; functionalGI, Functional gastrointestinal disorders; cholangioCA, Cholangiocarcinoma; buccalCA, Buccal cancer; femoralFx, Femoral fracture; MDD, Major depressive disorder; AKI, Acute kidney injury; TIA, Transient ischemic attack; SSS, Sick sinus syndrome; ovarianCA, Ovarian cancer; thyroidCA, Thyroid cancer; MR, Mitral regurgitation; SDH, Subdural hematoma; CML, chronic myeloid leukemia; Autoimmune, Autoimmune disease; aspirationPNA, Aspiration pneumonia; heartTx, Heaert Transplant; renalDz, Renal disease; hypopharynxCA, Hypopharyngeal carcinoma; MG, Myasthenia Gravis; AFL, Atrial flutter; spineOP, Spine operation; oropharynxCA,

Oropharyngeal cancer; SNHL, Sensorineural hearing loss; pulmEdema, Pulmonary edema; radioRx, Radiotherapy; abnlLFTs, Abnormal liver function; endometrialCA, Endometrial cancer; hypoK, Hypokalemia; AoS, Aortic stenosis; CP, Cerebral palsy; Chronic GN, Chronic Glomerulonephritis; trachealCA, Tracheal cancer; gallbladderCA, Gallbladder cancer; HSV, herpes simplex virus; pulmHTN, Pulmonary hypertension; PSA, Elevated prostate specific antigen; UC, Ulcerative colitis; AoA, Aortic aneurysm; Fx, Fracture; PSVT, Paroxysmal supraventricular tachycardia; larynxCA, Laryngeal cancer; HyperK, Hyperkalemia; PE, Pulmoanry embolism; AGE, Acute gastroenteritis; myeloproliferativeDz, Myeloproliferative disease; PID, Pelvic inflammatory disease; AoD, Dissection of aorta; AsS, Aoric stenosis; hypoNa, Hyponatremia; TKR, Total knee replacement; NTM, Non-tuberculosis mycobacterium; AVshunt, Arteriovenous shunt; MS, Multiple sclerosis; AVB, Atrioventricular block; PCKD, Polycystic kidney disease; PTX, Pneumothorax; ureterCA, Ureter Cancer; ITP, Immune Thrombocytopenia Purpura; MSA, multiple system atrophy; CervicalCIN, Cervical intraepithelial neoplasia; tonsillarCA, Tonsillar cancer; GVHD, Graft-versus-host disease; LAP, Lymphadenopathy; AIDS, Acquired Immunodeficiency Syndrome; gingivaCA,Gingival cancer; VBI, Vertebrobasilar insufficiency; OAB, Overactive bladder; Flu, Influenza; BWL, Body weight loss; ILD, Interstitial lung disease; Hpylori, Helicobacter pylori; hyperCa, Hypercalcemia; SCI, Spinal cord injury; DNI, Deep neck infection; VH, Vitreous hemorrhage; THR, Total hip replacement; thymicCA, Thymic cancer; humerusFx, Humerus fracture; PBSCT, Peripheral Blood Stem Cell Transplantation; PPU, Perforated peptic ulcer; DCM, Dilated cardiomyopathy; AR, Aortic regurgitation; OSA, Obstructive sleep apnea; GV, Gastric varices; NF, Necrotizing fasciitis; CLL, Chronic lymphocytic leukemia; brainCA, Brain cancer; MUP, Metastatic cancer of unknown primary; IE, Infective endocarditis; IBS, Irritable bowel syndrome; SAH, Subarachnoid hemorrhage; CMV, Cytomegalovirus; VSD, Ventricular Septal Defect; GIST, Gastrointestinal stromal tumor; UterineCA, Uterine cancer; spineFx, Spine fracture; duodenumCA, Duodenal cancer; PD, Peritoneal dialysis; ocularHTN, Ocular Hypertension; ALL, Acute lymphoblastic leukemia; PVC, Premature ventricular complex; ALS, Amyotrophic lateral sclerosis; adjustmentDz, Adjustment disorder; ankleFx, Ankle fracture; CCRT, Concurrent chemoradiotherapy
