# Supplementary figures and images for: Developing Machine-Learning Models to Predict Bacteremia in Febrile Adults Presenting to the Emergency Department: A Retrospective Cohort Study from a Large Center
Source: West J Emerg Med. 2025 May 30;26(3):617–26. doi: 10.5811/westjem.35866 (PMC12208070; doi:10.5811/westjem.35866)

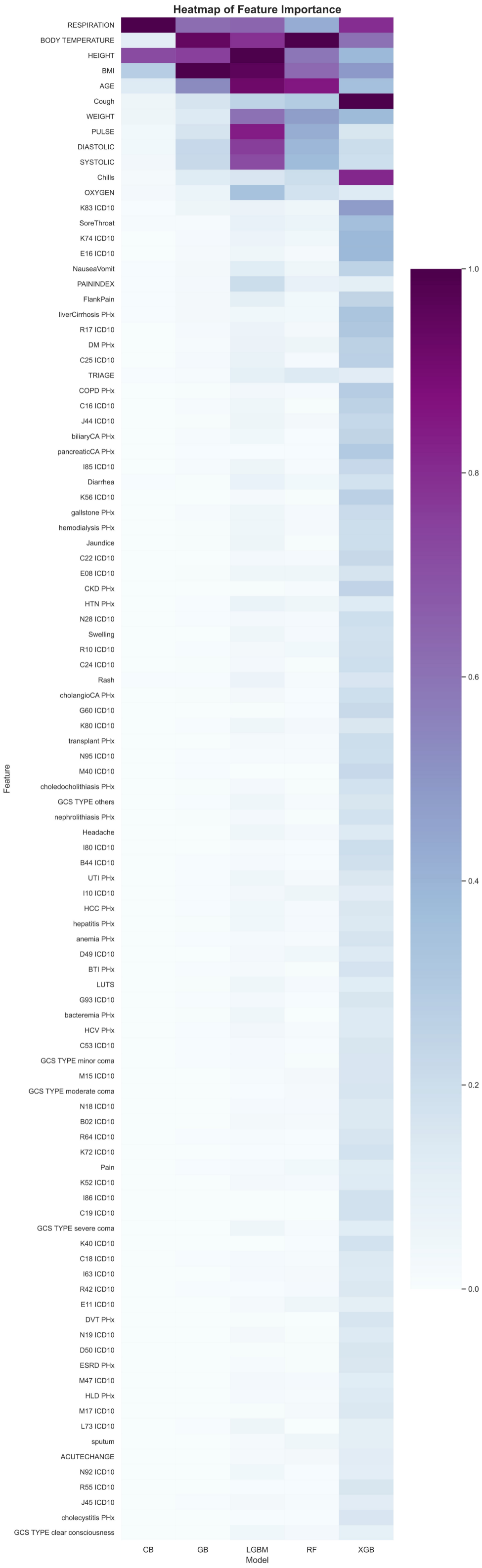

Supplement: Supplementary file 3 [file wjem-26-617-supplementary_fig_s1.pdf]
