# Supplementary material for: Developing Machine-Learning Models to Predict Bacteremia in Febrile Adults Presenting to the Emergency Department: A Retrospective Cohort Study from a Large Center
Source: West J Emerg Med. 2025 May 30;26(3):617–26. doi: 10.5811/westjem.35866 (PMC12208070; doi:10.5811/westjem.35866)

SUPPLEMENTARY FIGURE S2

The SHapley Additive exPlanations (SHAP) values of the top 100 important features as a way to explain the output of the constructed machine learning models by selecting 8-fold cross validation using XGBoost, Gradient boosting, LightGBM, and Random Forest.

XGBoost

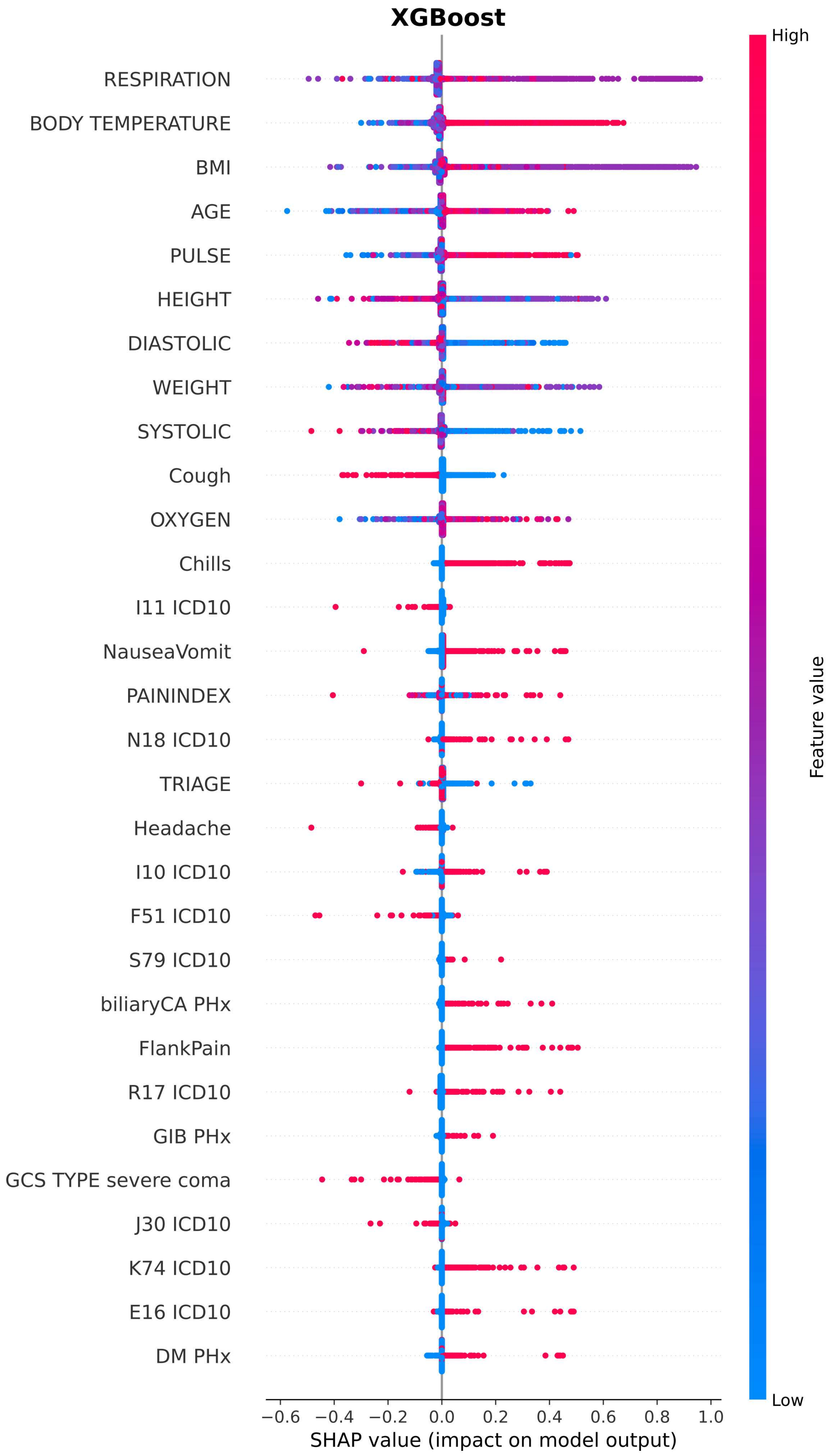
XGBoost
RESPIRATION
BODY TEMPERATURE
BMI
AGE
PULSE
HEIGHT
DIASTOLIC
WEIGHT
SYSTOLIC
Cough
OXYGEN
Chills
I11 ICD10
NauseaVomit
PAININDEX
N18 ICD10
TRIAGE
Headache
I10 ICD10
F51 ICD10
S79 ICD10
biliaryCA PHx
FlankPain
R17 ICD10
GIB PHx
GCS TYPE severe coma
J30 ICD10
K74 ICD10
E16 ICD10
DM PHx
−0.6 −0.4 −0.2 0.0 0.2 0.4 0.6 0.8 1.0
SHAP value (impact on model output)
High
Feature value
Low

Gradient boosting

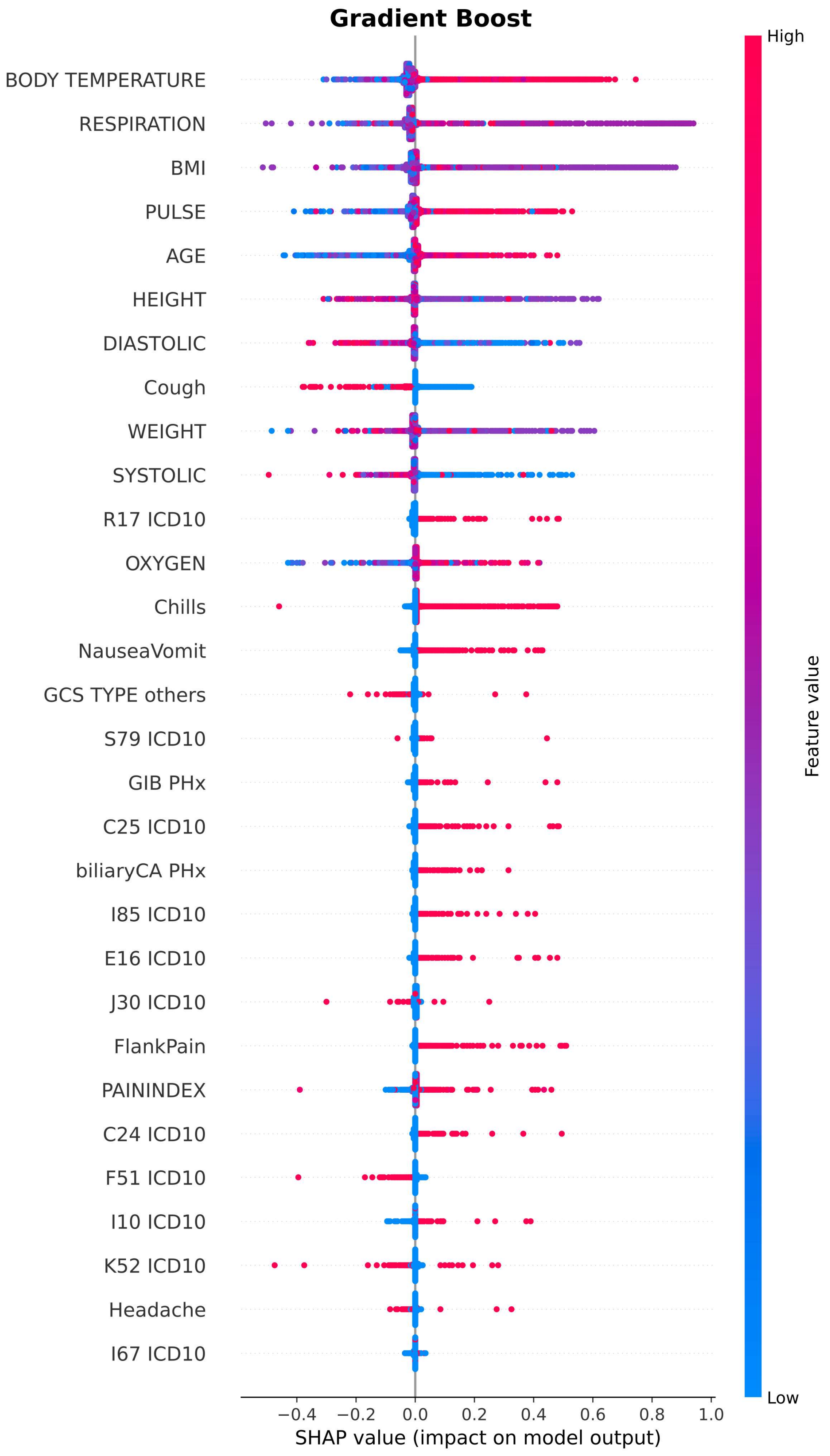

Light GBM

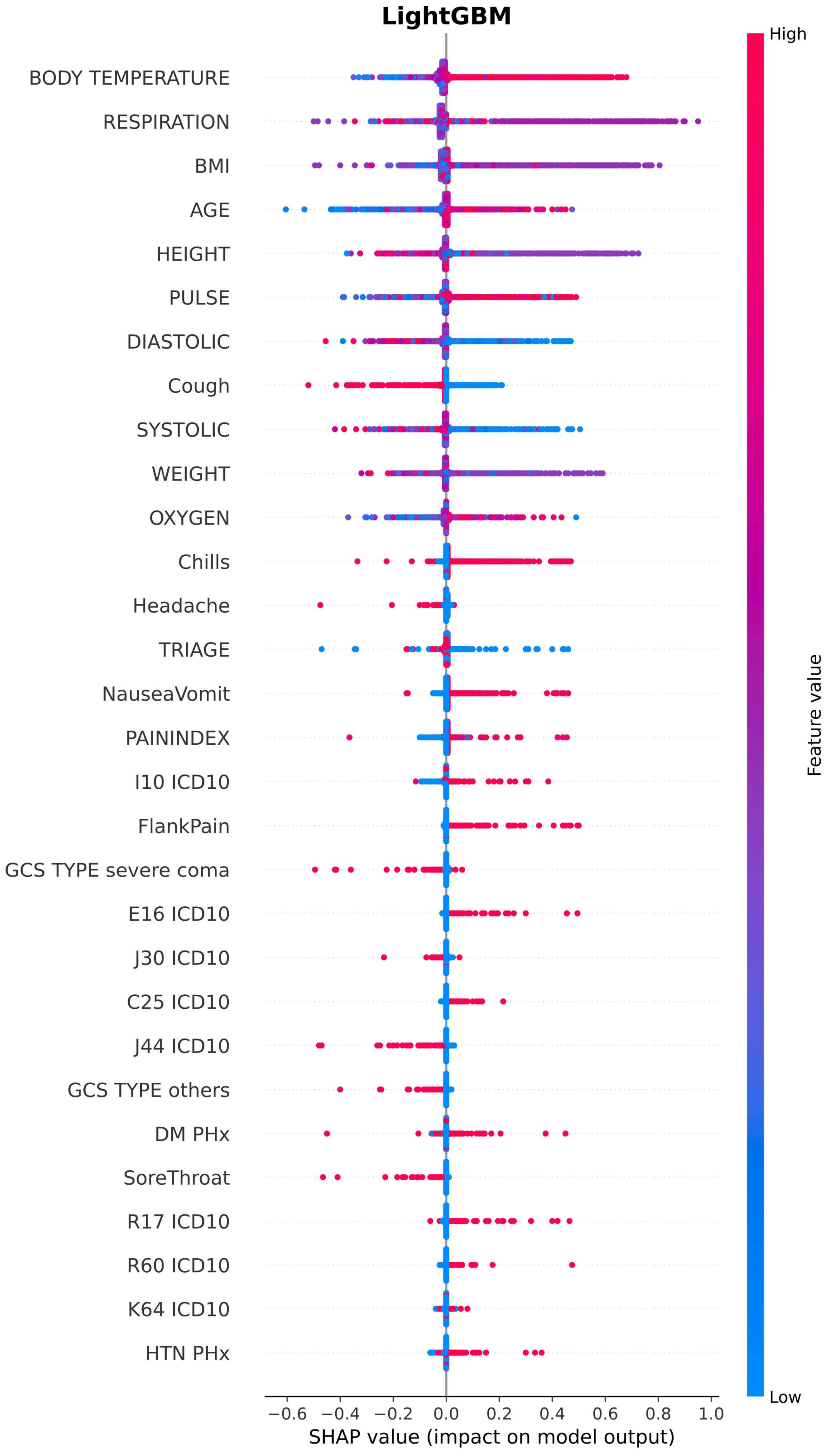
LightGBM
BODY TEMPERATURE
RESPIRATION
BMI
AGE
HEIGHT
PULSE
DIASTOLIC
Cough
SYSTOLIC
WEIGHT
OXYGEN
Chills
Headache
TRIAGE
NauseaVomit
PAININDEX
I10 ICD10
FlankPain
GCS TYPE severe coma
E16 ICD10
J30 ICD10
C25 ICD10
J44 ICD10
GCS TYPE others
DM PHx
SoreThroat
R17 ICD10
R60 ICD10
K64 ICD10
HTN PHx
−0.6 −0.4 −0.2 0.0 0.2 0.4 0.6 0.8 1.0
SHAP value (impact on model output)
High
Low
Feature value

Random Forest

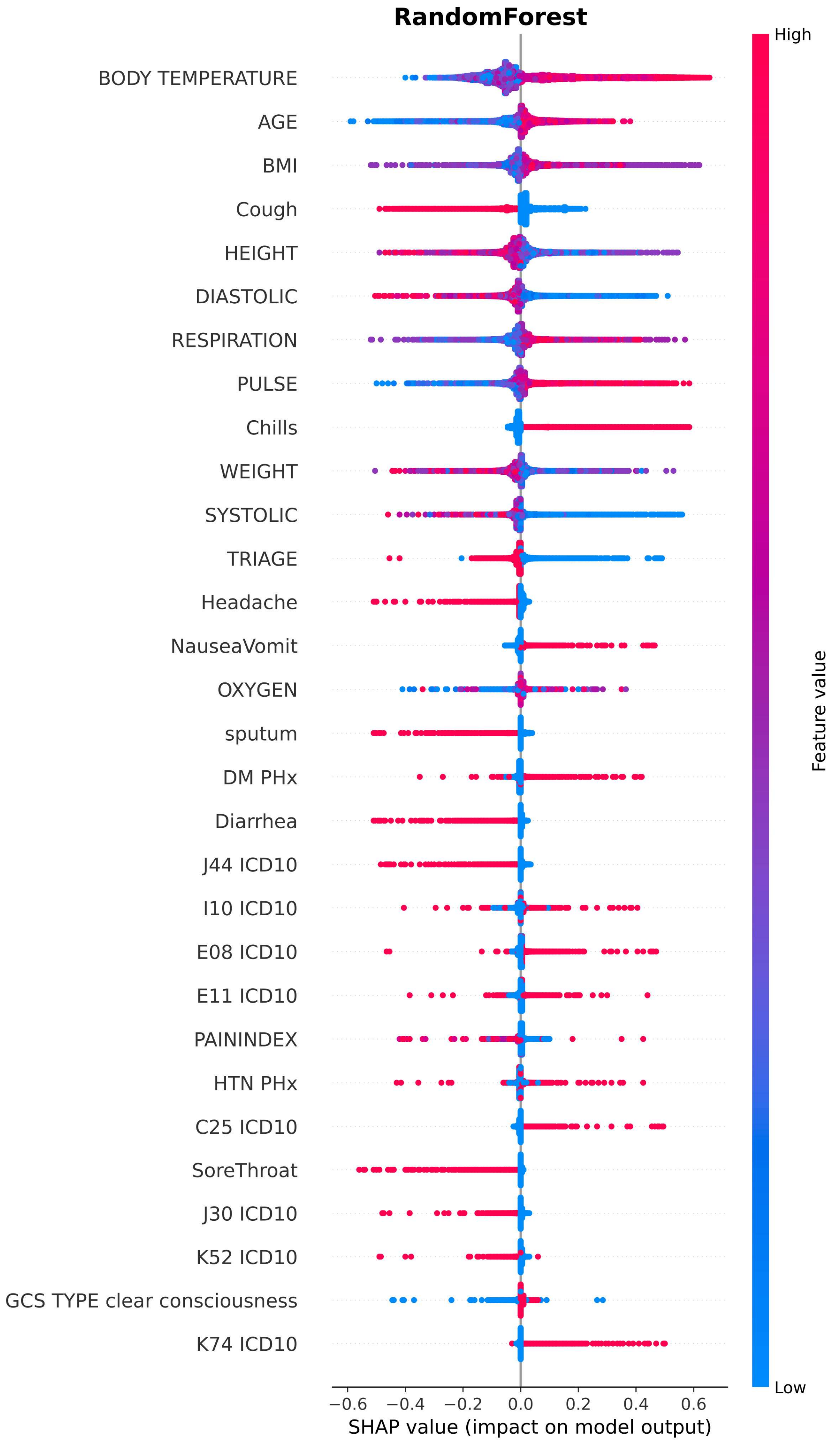

Supplement: Supplementary file 4 [file wjem-26-617-supplementaryfigs2.pdf]
